# Olefin Metathesis Catalyzed by a Hoveyda–Grubbs-like Complex Chelated to Bis(2-mercaptoimidazolyl) Methane: A Predictive DFT Study

**DOI:** 10.1021/acs.jpca.1c09336

**Published:** 2022-01-26

**Authors:** J. Pablo Martínez, Bartosz Trzaskowski

**Affiliations:** Centre of New Technologies, University of Warsaw, 02-097 Warszawa, Poland

## Abstract

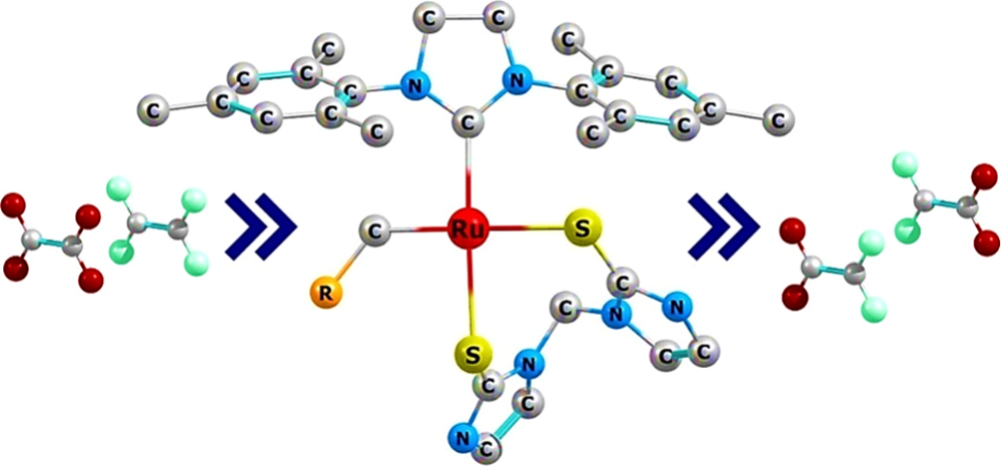

Although highly selective complexes for the cross-metathesis of olefins, particularly oriented toward the productive metathesis of *Z*-olefins, have been reported in recent years, there is a constant need to design and prepare new and improved catalysts for this challenging reaction. In this work, guided by density functional theory (DFT) calculations, the performance of a Ru-based catalyst chelated to a sulfurated pincer in the olefin metathesis was computationally assessed. The catalyst was designed based on the Hoveyda–Grubbs catalyst (SIMes)Cl_2_Ru(=CH–o–O*i*PrC_6_H_4_) through the substitution of chlorides with the chelator bis(2-mercaptoimidazolyl)methane. The obtained thermodynamic and kinetic data of the initiation phase through side- and bottom-bound mechanisms suggest that this system is a versatile catalyst for olefin metathesis, as DFT predicts the highest energy barrier of the catalytic cycle of ca. 20 kcal/mol, which is comparable to those corresponding to the Hoveyda–Grubbs-type catalysts. Moreover, in terms of the stereoselectivity evaluated through the propagation phase in the metathesis of propene–propene to 2-butene, our study reveals that the *Z* isomer can be formed under a kinetic control. We believe that this is an interesting outcome in the context of future exploration of Ru-based catalysts with sulfurated chelates in the search for high stereoselectivity in selected reactions.

## 1 Introduction

**Scheme 1:**
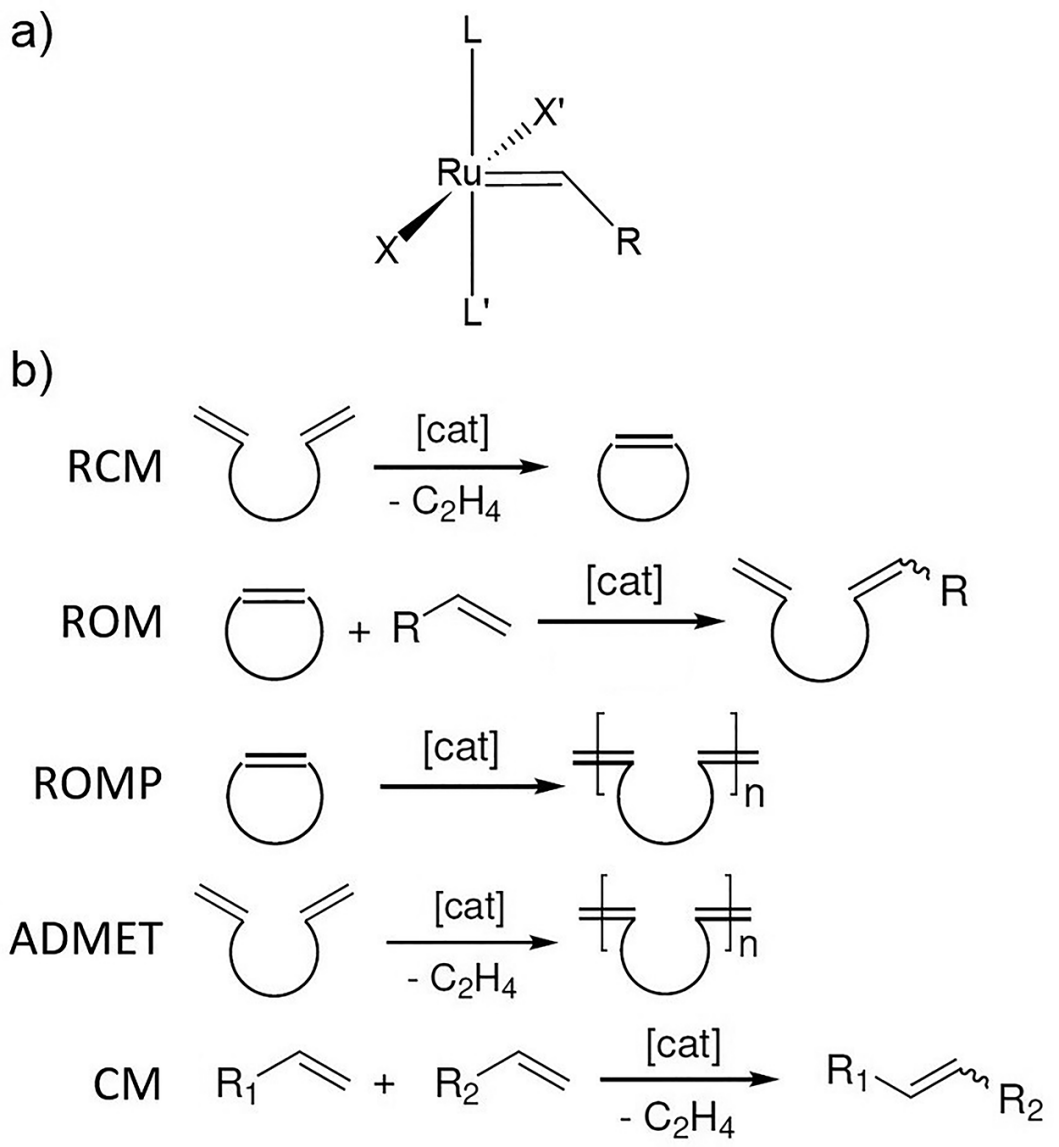
(a) General Ru(II)-Based Catalyst Structure and (b) Olefin Metathesis Applications

In the beginning of olefin metathesis, such reactions were performed employing undefined mixtures of molybdenum and tungsten salts adsorbed on alumina under harsh conditions and additives.^1,2^ Therefore, subsequent investigations focused on detailed descriptions of metathesis catalysts to obtain high control over the reaction, which led to the first well-defined Schrock catalysts.^3,4^ This discovery encouraged the development of a family of catalysts with early transition metals.^5,6^ Unfortunately, these species showed some operational issues related to oxophilicity, solvents, as well as limited tolerance to moisture or a number of different functional groups, even though some air-stable and user-friendly complexes were obtained.^7^ These drawbacks were overcome by Ru-based olefin metathesis, thus leading to a completely new group of catalysts. In this regard, the aqueous ring-opening metathesis polymerization of strained olefins, initially catalyzed by ruthenium salts,^8^ allowed the determination of the general structure of Ru-based catalysts (Scheme 1a).^9,10^ The importance of this well-defined structure is reflected in the reaction control as structural modifications can be envisaged to enhance the initiation rate, turnover number, lifetime, or stereoselectivity. In fact, the addition of carbenes to the phosphine–ruthenium complexes, RuCl_2_(PR_3_)_3_, resulted in stable catalysts with improved tolerance to air, moisture, and a wide spectrum of functional groups. This family of species is today known as the Grubbs first-generation catalysts (**GI**) and are commercially available.^11^

Ligands characterized by σ-donor capabilities, such as phosphines, induce a high electron density in Ru, and this idea was realized by introducing NHCs as L-spectators.^12^ First synthesized by Arduengo,^13^ NHCs have been widely utilized in the field of organometallic chemistry, probably due to their ability to coordinate several elements,^14^ which makes them useful in both homogeneous and heterogeneous catalysis reactions.^15^ The newly developed set of complexes bearing NHCs, known as the Grubbs second-generation catalysts (**GII**), are also commercially accessible.^16^ Interestingly, the incorporation of a chelating ligand, such as 2-isopropoxystyrene, in Hoveyda–Grubbs (**HG**) catalysts^17^ revealed new opportunities in tuning catalytic properties through substituent variation in the styrene fragment.^18,19^ For example, the substitution of NO_2_ prepared by Grela made possible an efficient low-temperature metathesis, which is attributed to the electron-withdrawing effect of the NO_2_ moiety, that weakens the Ru–O bond strength.^20^ Other examples encompass Ru–N chelating agents like amidobenzylidene that shows catalytic activity in acidic medium^21^ or carbamate and acetamide that induce metathesis more effectively in the presence of RuCl_2_···H weak bonds compared to similar catalysts lacking such H bonds.^22^

**Scheme 2:**
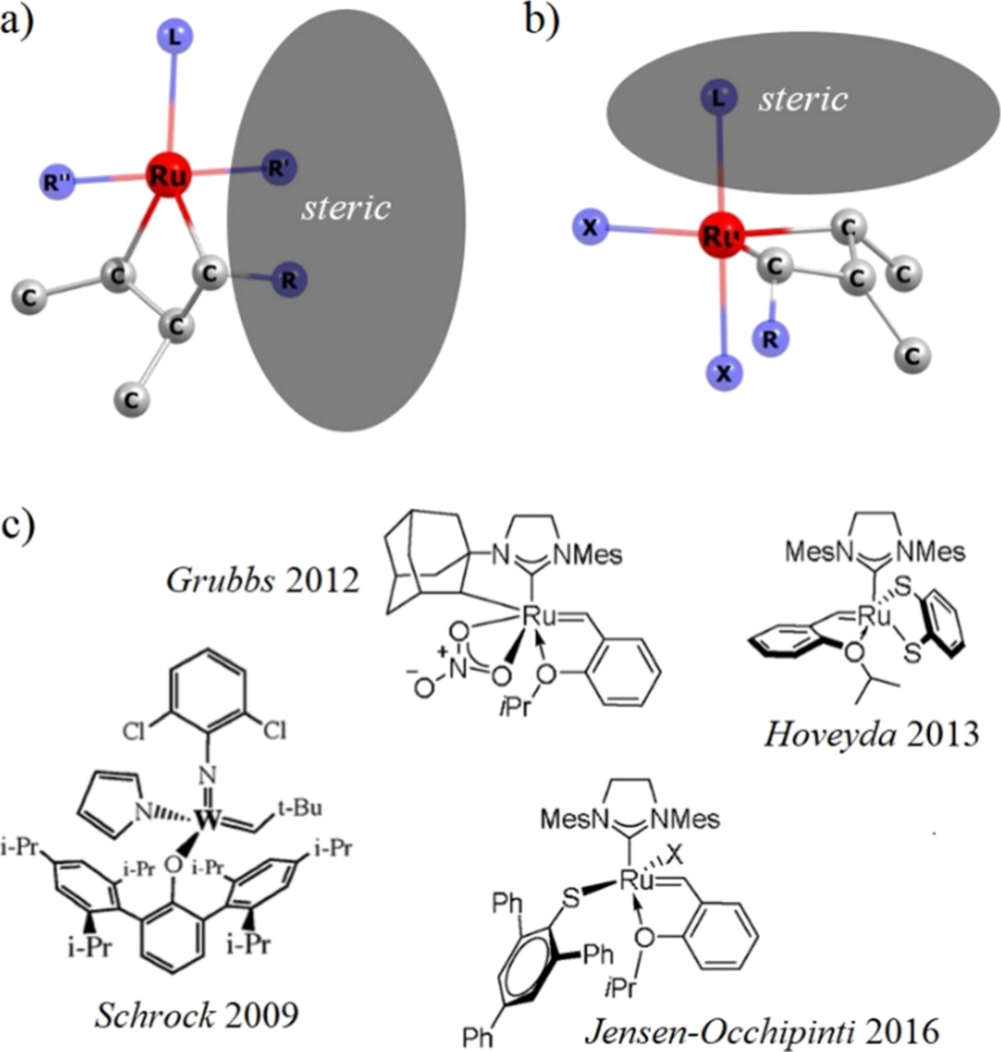
Graphical Representation of the Design of *Z*-Selective Catalysts via (a) Bottom-Bound and (b) Side-Bound Mechanisms, and (c) Examples of *Z*-selective Catalysts

Olefin metathesis is technically considered a single reaction, yet it can be categorized into several types, such as (i) ring-closing metathesis, (ii) ring-opening metathesis (ROM), (iii) ROM polymerization (ROMP), (iv) acyclic diene metathesis, and (v) homocoupling self-metathesis or cross-metathesis (CM) (see Scheme 1b). Today, all these processes have reached an industrial scale,^11^ particularly in the pharmaceutical field.^23,24^ In the case of CM, it has become an important tool in synthetic chemistry, but the ability to selectively form the desired product is still a significant challenge because two stereoisomers are normally produced: the *E-* and *Z-*olefins. This reaction mixture represents operational difficulties, as component separation is often problematic and costly.^25^ For benchmark CM reactions and reaction yields of 60% or less using **GI** and **GII** catalysts, the resulting *E*/*Z* ratios are between 3:1 and 5:1, yet the preference for the *E*-olefin can be significantly increased for reaction yields above 60%, which may be explained in terms of the higher thermodynamic stability alluded to the *E* isomer.^26,27^ In this regard, the incorporation of bulky ligands into the catalyst backbone prevents sterically the formation of the *E* conformer of the olefin in metallacyclobutanes (MCBs), this latter originally proposed by Hérisson and Chauvin,^28^ resulting in an increased yield of *Z* olefin under a kinetic control. In olefin metathesis, *Z*-selective catalysts were first introduced by Schrock and Hoveyda (Mo- and W-based), and some of them showed ratios above 98% for *Z*-olefins with yields above 50% (for a general picture, see Scheme 2).^29^ For Ru-based catalysts, the first selective CM catalysts were based on **GII** catalysts.^30^ Recently, however, guided by the predictive density functional theory (DFT) calculations, Jensen et al. synthesized a phosphine-based **HG** catalyst by replacing one chloride with the bulky 2,4,6-triphenylbenzenethiolate, which showed 70–95% selectivity with respect to *Z*-olefins.^31^

The stereoselectivity of Ru-based metathesis catalysts may be understood based on two experimentally validated reaction mechanisms: bottom-bound^32^ and side-bound^33^ (see Scheme 2). Accordingly, Grubbs et al. developed a series of *Z*-selective catalysts by modifying **HG** catalysts, particularly by replacing chloride ligands with pivalate or nitrate moieties;^34,35^ the latter resulted in greater stability and higher efficiency for CM reactions.^36^ Based on DFT calculations, the authors demonstrated that the side-bound mechanism is mainly induced so that the *Z* isomer is kinetically favored due to reduced steric compression, more favorable van der Waals interactions, and stronger d-orbital backdonation between the catalyst and the reacting olefin.^37^

Motivated by these findings and the fact that the catalytic activity correlates with the molecular structure, we decided to explore in this work the design of a catalyst for an efficient and selective CM by incorporating a sulfurated pincer into the molecular structure of **HG**. This strategy was previously implemented by Hoveyda et al. via the substitution of chlorides by a catechothiolate ligand (see Scheme 2c), which resulted in a highly *Z*-selective catalyst and high yields for ROMP and ROCM.^38^ It is observed that this approach focused on stereoselectivity differs from other sulfur chelates previously reported, in which the ether R_2_O→Ru in **HG** catalysts is replaced by a thioether R_2_S→Ru, resulting in enhancements in the catalytic activity.^39−44^ It should be clarified, however, that we aimed at the formation of an active species that resembles the structure depicted in Scheme 2b. In this regard, several authors have previously reported predictive catalysis based on DFT calculations verified by experimental evidence.^45−55^

## 2 Computational Details

Geometry optimizations were carried out without constraints, and the characterization of stationary points was performed by analytical frequency calculations at the B3LYP-D3/LACVP** level of theory.^56−59^ All transition states and the connecting local minima were searched using linear transit calculations using the same DFT method; specifically, the progress of the reactions was monitored by varying a dihedral angle in the case of styrene rotation or a carbon–carbon distance for steps regarding MCBs. Gibbs free energies used throughout this report are the sum of electronic energy (M06-D3/LACV3P++**//B3LYP-D3/LACVP**),^60^ solvation energy (single-point Poisson–Boltzmann self-consistent polarizable continuum method in toluene),^61,62^ zero-point energy correction, thermal correction to enthalpy, and the negative product of temperature and entropy, all at 298 K. The M06 functional already includes medium-range dispersion so that M06-D3 may overestimate the effect of dispersion due to the double-counting of these effects.^63^ On the other hand, the addition of D3 correction to M06 was shown to improve the results for many organic reactions, particularly in the treatment of weak interactions.^64^ Moreover, the computational approach we selected has been previously validated against experimental data.^65−68^ In all instances, standard convergence criteria and a fine grid for DFT calculations were used, as implemented in Jaguar ver. 9.5.^69^ The buried volume, % *V*_bur_, to determine steric maps around the first coordination of Ru was calculated with the SambVca package ver. 2.1 developed by Cavallo et al.^70,71^ % *V*_bur_ is defined as the amount of the first coordination sphere of the metal occupied by a given ligand. The radius of the sphere around the metal center was set to 3.5 Å, whereas for the atoms we selected the Bondi radii scaled by 1.17, and a mesh of 0.1 Å was used to scan the sphere for buried voxels.

## 3 Results and Discussion

**Scheme 3:**
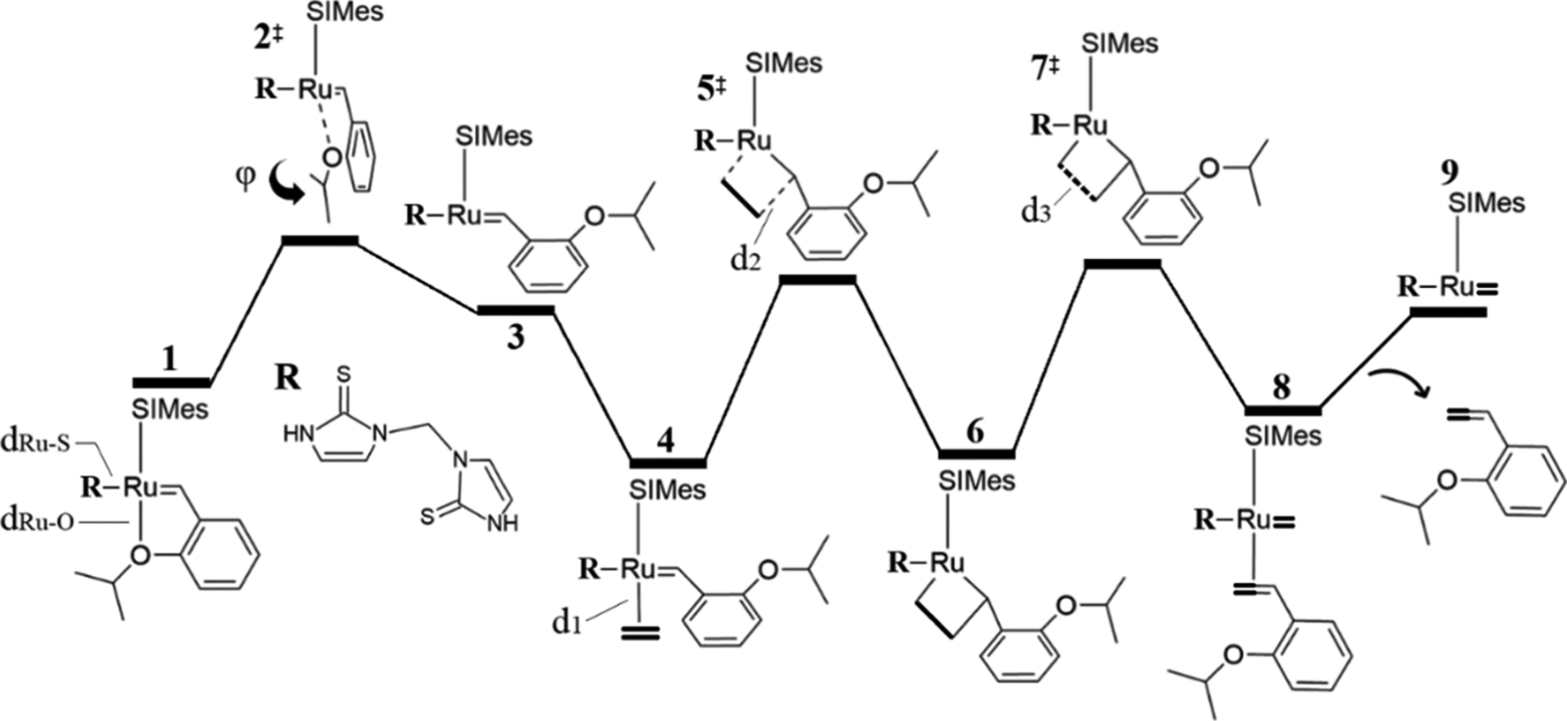
Initiation Phase for the Metathesis Catalytic Cycle and the Labeling Scheme along with Structural Parameters Used throughout This Work

The chemical structures and the general reaction mechanism studied in this work are depicted in Scheme 3. 1,3-Bis(2,4,6-trimethylphenyl)-4,5-dihydroimidazol-2-ylidene (SIMes) was used as an auxiliary ligand as it affords a higher activity than the 1,3-bis(2,4,6-trimethylphenyl)imidazole-2-ylidene (IMes) ligand.^16^ The derivatives of the **HG** catalyst in this work have both chlorides replaced by the chelating agent bis(2-mercaptoimidazolyl)methane, NHC=S, a chelate previously used to form rhenium complexes that exhibited agostic interactions.^72,73^ As ruthenium and rhenium share some of chemical properties (e.g., a well-defined Re(VII) complex or also the oxide Re_2_O_7_ have been demonstrated to be active catalysts for olefin metathesis^74,75^), we hypothesize that HNC=S can also be relatively strongly chelated to ruthenium. In terms of synthetic protocols, NHC=S replaced two bromines in the case of the species [Re(CO_3_)Br_3_]^−^ under reflux for 3 h;^73^ the ability of NHC=S to replace halogens may also be observed in the replacement of chlorines in **HG,** following a similar synthetic strategy.

**Figure 1:**
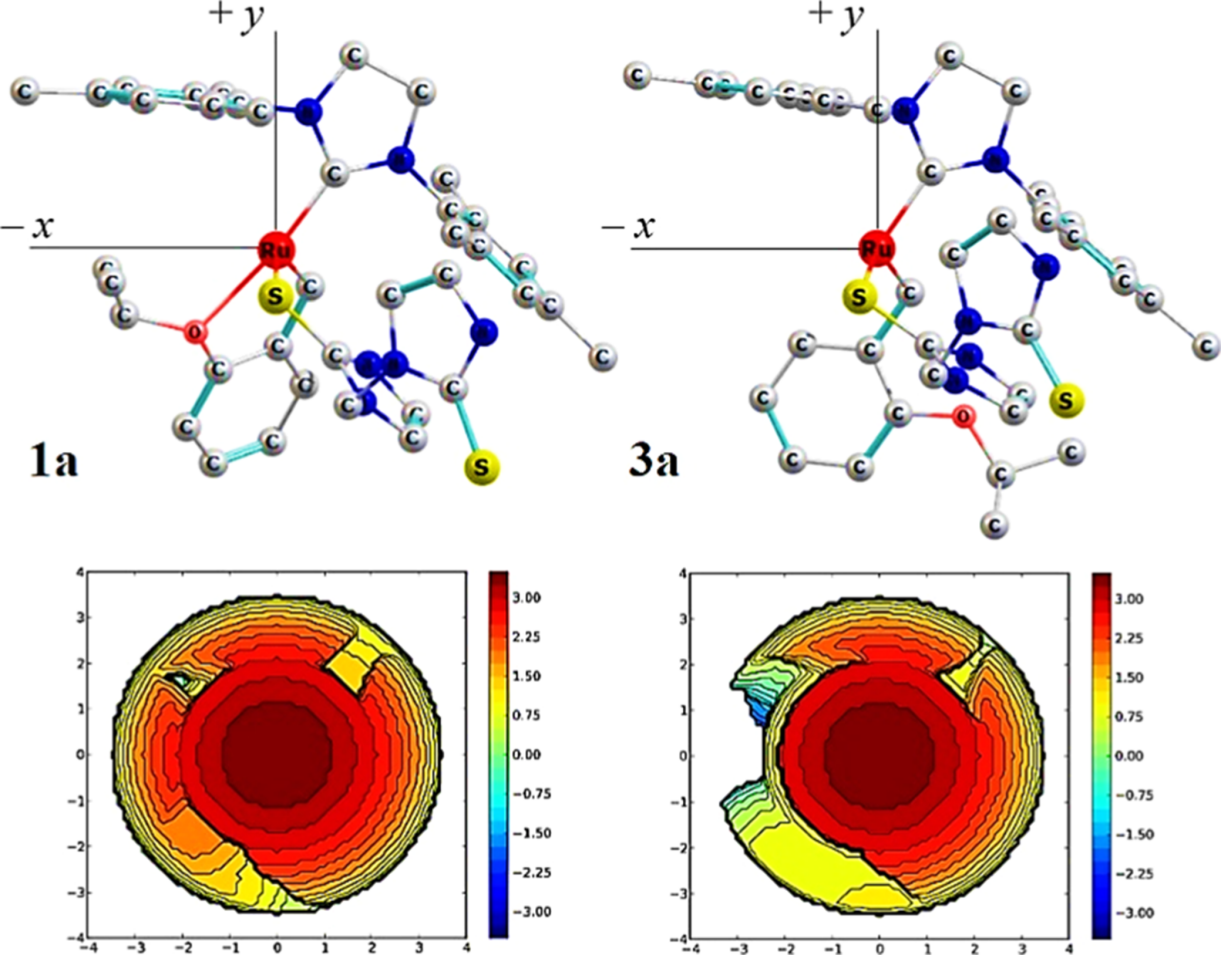
Topographic steric maps in the *xy* plane of the precatalyst (left) and active catalyst (right) studied in this work. The Ru atom is at the origin, the S atom is on the *z* axis, and the NHC of the SIMes ligand is on the *xy* plane. The contour curves are given in Å.

The main target of the current work is the thermodynamic and kinetic evaluation of the catalytic activity of complex **1** for the metathesis of olefins. As such, reactions were monitored by the dihedral angle φ defined in Scheme 3, which accounts for the styrene rotation in the first step of the pathway, and the bond distances *d*_n_ related to the bond formation and rupture of the reacting olefin carbon atoms, particularly useful in the description of the 2,2-cycloaddition and 2,2-cycloreversion steps. In this regard, it has been shown that **HG** catalysts preferably initiate via an interchange mechanism for small olefins and a dissociative mechanism for larger substrates; moreover, the initiation phase was also demonstrated to be the rate-limiting step for the entire catalytic cycle.^76^ The preference for the initiation mechanism may be rationalized in terms of steric hindrance; for instance, the initiation phase exclusively occurs via a dissociative pathway for the bulky olefin diethyl-diallyl malonate, yet both mechanisms compete in the case of the less bulky butyl-vinyl ether.^77^ For our study, we selected ethylene as the substrate to describe the initiation phase catalyzed by complex **1**. However, even though the chosen substrate is small so that the interchange mechanism may be considered, we focus on the dissociative path because the NHC=S fragments already add a steric factor to the catalyst. In fact, we quantify the steric properties of precatalyst **1** and active catalyst **3** through topographic steric maps (see Figure 1). The active catalyst formed after Ru–O ether dissociation shows a depletion in % *V*_bur_ that allows olefin coordination. In contrast, Ru is sterically hindered in the precatalyst so that the interchange mechanism becomes less competitive.

**Figure 2:**
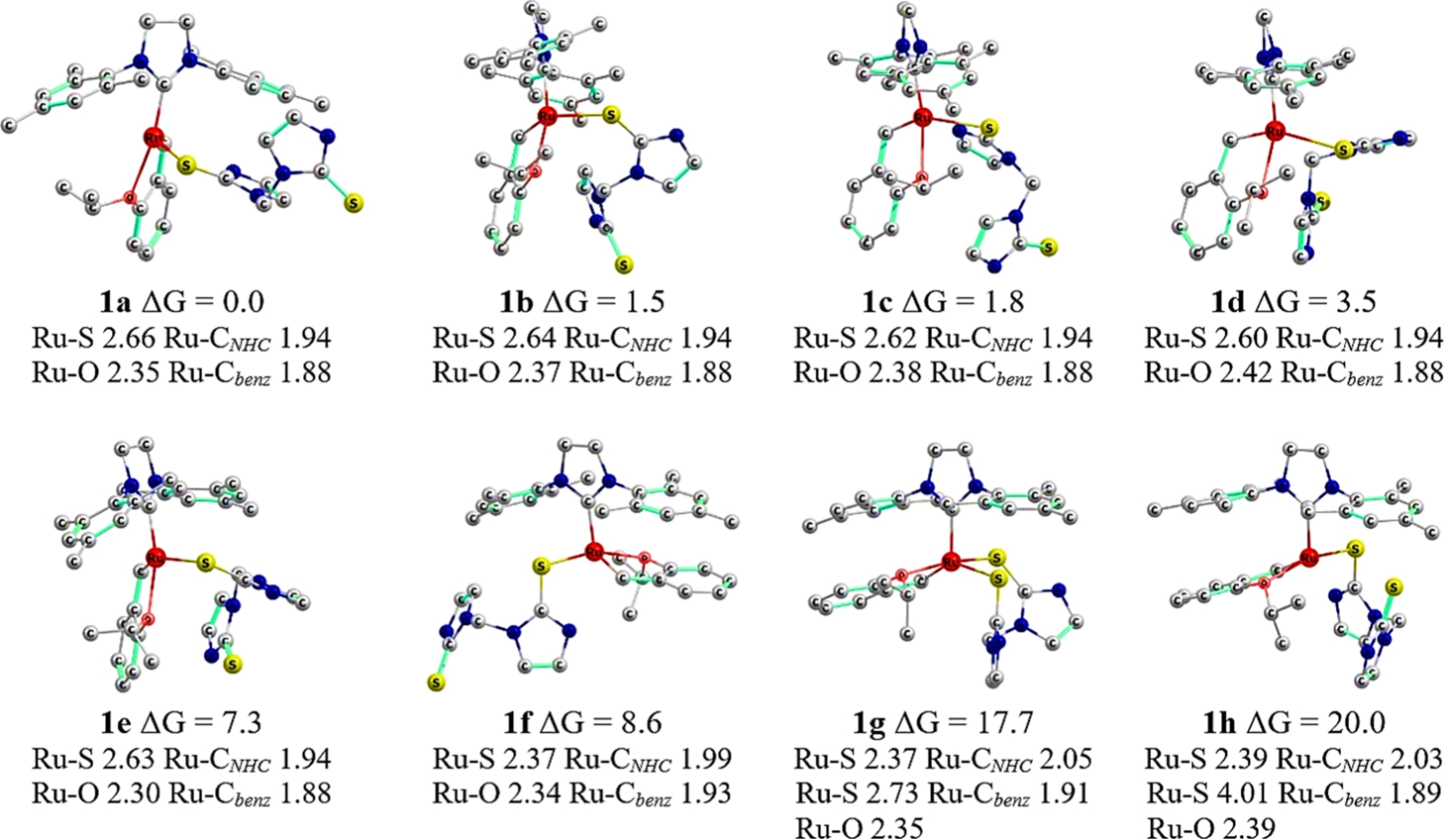
Three-dimensional representation of DFT-optimized geometries of precatalyst **1** and Gibbs energy comparisons in kcal/mol. Hydrogen atoms are hidden for the sake of clarity. Main bond lengths around the metal center are given in Å.

Our study began with the investigation of 20 preliminary structures that were manually prepared by varying the positions and orientations of thiones and styrene. After geometry optimizations, eight complexes were obtained (details in Figure 2), with **1a** being the most thermodynamically stable structure. The styrene and SIMes fragments in all complexes show the typical configuration of an **HG-**type catalyst. Even though the starting structures of all systems had ruthenium bonds to both thiones, we observed that one of the Ru–S bonds was dissociated in several complexes after the geometry optimization procedure. For the other shorter Ru–S bond, the respective distance in **1a** of 2.66 Å significantly differs from the previously reported values; for instance, the crystal structure of a sulfur-chelated **HG-**type catalyst revealed a Ru–S distance of ca. 2.30 Å.^78^ The increased bond distance may be attributed to a dative bond between Ru and a sulfur lone pair. The structures of **1b–e** differ in the NHC=S orientation, and their Ru–S bond distance is similar to that of **1a** as it ranges from 2.60 to 2.64 Å. The bonding scheme is different for **1f–h**, where the styrene fragment is side-oriented, and the Ru–S bond distance ranges from 2.37 to 2.39 Å. However, despite this decrease in bond distance, the side orientation of styrene causes destabilization of these precatalysts. This outcome is particularly evident for structures **1g** and **1h** that are destabilized by more than 17 kcal/mol with respect to **1a**.

### 3.1 Catalytic Performance of the Most Stable Complex

**Figure 3:**
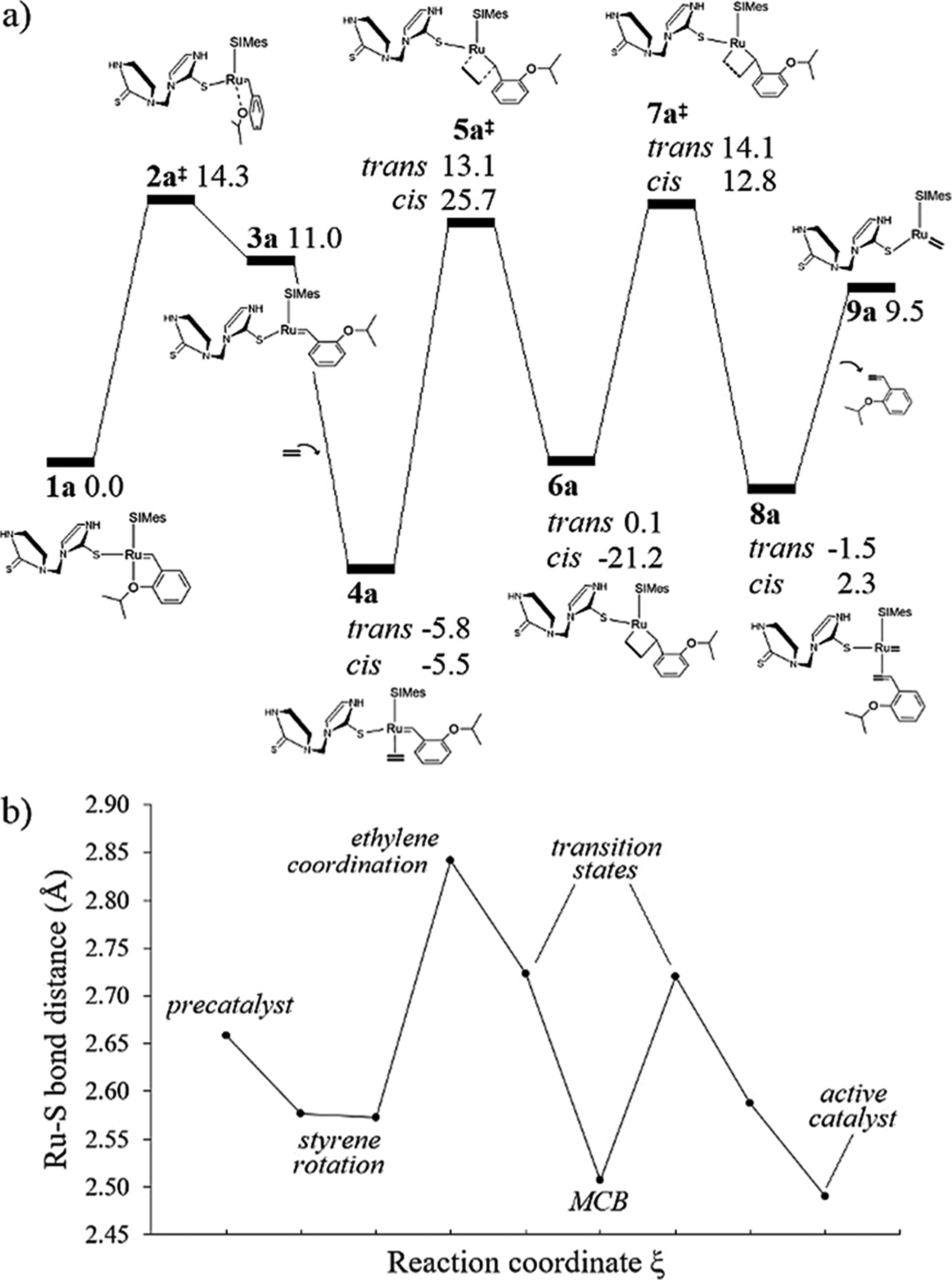
(a) Relative Gibbs free energy profile (kcal/mol) of the initiation phase for precatalyst **1a**. The bottom- and side-bound mechanisms are compared, in which the olefin binding can be respectively *trans* or *cis* to the SIMes ligand. (b) Ru–S bond distance variation through the bottom-bound catalytic cycle.

The energy profiles associated with the catalytic performance of **1a** are shown in Figure 3a, and the structural parameters used to monitor the reaction are reported in Table 1. Within the dissociative pathway, styrene is rotated by increasing or decreasing the starting value of φ = 8.9°, thus causing a rupture of the bond formed between the Ru and O atoms, initially at *d*_Ru–O_ = 2.35 Å. In the case of φ < 0, we localized a transition state **2a**^‡^ characterized by a small imaginary frequency, for which the vibrational mode suggests styrene rotation. The calculated energy barrier associated with this structure is 14.3 kcal/mol, and it occurs at φ = −68.6° and *d*_Ru–O_ = 3.42 Å. After overcoming such an energy barrier, the dissociative pathway progresses to the stationary point **3a**, φ = −125.0° and *d*_Ru–O_ = 4.22 Å. On the contrary, the rotation of styrene for φ > 0 leads to the analogous structure **3a′**, φ = 111.2° and *d*_Ru–O_ = 4.05 Å, which is formed at 17.3 kcal/mol. These results suggest that the styrene rotation in the dissociative mechanism preferably occurs in the direction φ < 0.

**Table 1:** Evolution of Structural Parameters as Defined in Scheme 3 through the Initiation Phase for Complex **1a**^a^

ξ	φ	*d*_Ru–O_	*d*_1_	*d*_2_	*d*_3_
**1a**	8.9	2.346			
**2a**^‡^	–68.6	3.424			
**3a′**	111.2	4.046			
**3a**	–125.0	4.219			
**4a**_*trans*_	–107.4	4.099	2.288	2.977	1.393
**5a**_*trans*_^‡^	155.3	4.618	2.339	2.181	1.449
**6a**_*trans*_	101.6	4.280	2.540	1.553	1.535
**7a**^‡b^	130.6	4.707	2.267	1.465	2.223
**8a**^b^	122.6	4.655	2.152	1.461	3.180
**9a**		4.402	5.792	1.340	6.138
**9a′**		3.555	5.802	1.341	7.293
Side-Bound Mechanism					
**4a**_*cis*_	–150.0	4.580	2.150	3.283	1.418
**5a**_*cis*_^‡^	–155.2	4.764	2.333	2.091	1.447
**6a**_*cis*_	–108.7	3.880	2.714	1.532	1.533
**7a**^‡c^	–118.7	4.420	2.313	1.459	2.232
**8a**^c^	–105.2	4.212	2.202	1.426	2.843

a Torsional angle, φ, in degrees and bond distances, *d*_n_, in Å.

b Originated from olefin *trans* binding.

c Originated from olefin *cis* binding.

The olefin η^2^ coordination to Ru can occur through a bottom-bound mechanism, in which ethylene is bonded *trans* to the SIMes ligand, or through *cis* binding, which takes place through the side-bound case (we will use the *cis*/*trans* subscripts to denote this characteristic). Species **3a** acquires a structural conformation that allows the coordination of the olefin with the metal center, with *d*_1_ = 2.29, leading to the stabilized complex **4a**_*trans*_ (−5.8 kcal/mol with respect to the precatalyst, which represents a favorable driving force that promotes catalytic activity toward the metathesis reaction). At this point, we also examined the η^2^ coordination with a C=C bond belonging to one of the NHC=S rings, which resulted in an endergonic process that requires 9.1 kcal/mol (see Figure S1 in Supporting Information for structural details). As a result, such a C=C bond could not compete with the stabilizing η^2^ coordination of ethylene. The energy cost associated with the 2,2-cycloaddition step is 18.9 kcal/mol, which is estimated via the **5a**_*trans*_^‡^ transition state. For this transition state, *d*_1_ increases to 2.34 Å as the olefin carbon approaches benzylidene, *d*_2_ = 2.18 Å, also resulting in an increase in the distance of the ethylene C=C bond *d*_3_ from 1.39 to 1.45 Å. In this regard, the efficiency and rate of the olefin metathesis reaction may be described in terms of the thermodynamic descriptors of the MCB. For example, a highly stabilized MCB that is originated from Schrock catalysts may be associated with reduced catalytic performance.^79^ Furthermore, a previous study revealed that **GI** MCBs were destabilized by 8.4 kcal/mol as compared to those corresponding to **GII**, which explains to some extent the improved catalytic activity of **GII** catalysts.^80^ Therefore, to induce the progress of the reaction, the MCB should only be moderately stabilized.^45^ Our results show that the MCB **6a**_*trans*_ is energetically comparable to the precatalyst so that it is neither highly stabilized nor destabilized. In **6a**_*trans*_, the reacting olefin carbon is not coordinated to the metal center, *d*_1_ = 2.54 Å, and it forms single C–C bonds, which are reflected in the value of ca. 1.5 Å for *d*_2_ and *d*_3_.

The 2,2-cycloreversion of **6a**_*trans*_ required 14.0 kcal/mol to proceed to rupture the MCB through **7a**^‡^, which occurs at *d*_3_ = 2.22 Å, and it is a slightly exergonic process that leads to **8a**. In this step, we were unable to explicitly differentiate between *trans* and *cis* binding as a result of the rupture of the olefin. In **8a**, the reacting olefin carbon is again coordinated to Ru, *d*_1_ = 2.15 Å, forms a new double bond in the produced olefin, *d*_2_ = 1.46 Å, and it is completely separated from the other ethylene carbon, *d*_3_ = 3.18 Å. The formation of the 14e species **9a** requires 11.0 kcal/mol, and this stationary point is characterized as a complex formed between the released *ortho*-isopropoxy vinylbenzene and the active catalyst, *d*_Ru–O_ = 4.40 Å. We also located a similar structure, **9a′**, which differs in the styrene orientation, *d*_Ru–O_ = 3.56 Å, although it is destabilized by 8.2 kcal/mol as compared to **9a**. On the other hand, styrene release occurs at an energy cost of 11.1 kcal/mol if isolated species are considered, suggesting negligible interactions between the active catalyst and the styrene counterpart when forming complex **9a**, probably due to its distant separation (*d*_1_ = 5.79 and *d*_3_ = 6.14 Å).

The highest energy transition state in this part of the catalytic cycle occurs in the dissociative pathway, **2a**^‡^, leading to the 14e species **3a**, in agreement with previous experimental and computational data obtained for the **HG** catalyst.^76^ On the other hand, even though the energy of **2a**^‡^ is the largest in terms of the initiation phase, to determine the kinetic bottleneck of the reaction, it is necessary to examine all energy barriers with respect to the lowest energy intermediate, a conceptualization made employing the energetic span model.^81−84^ Accordingly, the crucial states that determine the course of the reaction are **4a**_*trans*_ and **7a**^‡^, which represent an overall cost of 19.9 kcal/mol. Previous kinetic studies in **HG** catalysts demonstrated that even olefin release may become the limiting step of the entire reaction,^85^ although the formation or rupture of the MCB may also be rate-determining.^86^ It is also worth mentioning that predictions based on thermodynamic arguments rarely reflect the product composition, as the stability of the isomers is often comparable.^25^ Furthermore, according to a computational study by Cavallo et al.,^87^ the origin of selectivity is best explained by considering the end of the reaction at the olefin release step. On the basis of DFT Gibbs free-energy profiles, they demonstrated that *Z* isomers are less prone to be released from the catalyst, and, as a result, these return to the reaction medium until the *E*-olefin is formed. Their study also shows that the activation energy related to the 2,2-cycloaddition step to form the *Z*-olefin could be similar or even lower than that corresponding to the *E* isomer, and, consequently, stereoselectivity may not be fully elucidated considering only the initial steps of the catalytic cycle.

The performance of **1a** as a metathesis catalyst may be driven by the modulation of the Ru–S bond throughout the catalytic cycle, as depicted in Figure 3b. Interestingly, we noted that the Ru–S bond is shortened for 14e species (MCB and the active catalyst). On the other hand, the addition of η^2^ electrons related to ethylene lengthens the Ru–S bond despite of energy release, which highlights the importance of thione in the bonding scheme of the catalyst. This is an important result, as the saturation of the coordination sphere around the metal can reduce the turnover number or even preclude the activity of the catalyst, as demonstrated for 18e Ru–vinylidene complexes with a hindered vacant site for olefin coordination.^88^ However, the incorporation of chelating phosphine sulfonates leading to 18e Ru alkylidenes showed catalytic activities that exceeded the performance achieved via comparable nonchelating phosphine catalysts.^89^

**Figure 4:**
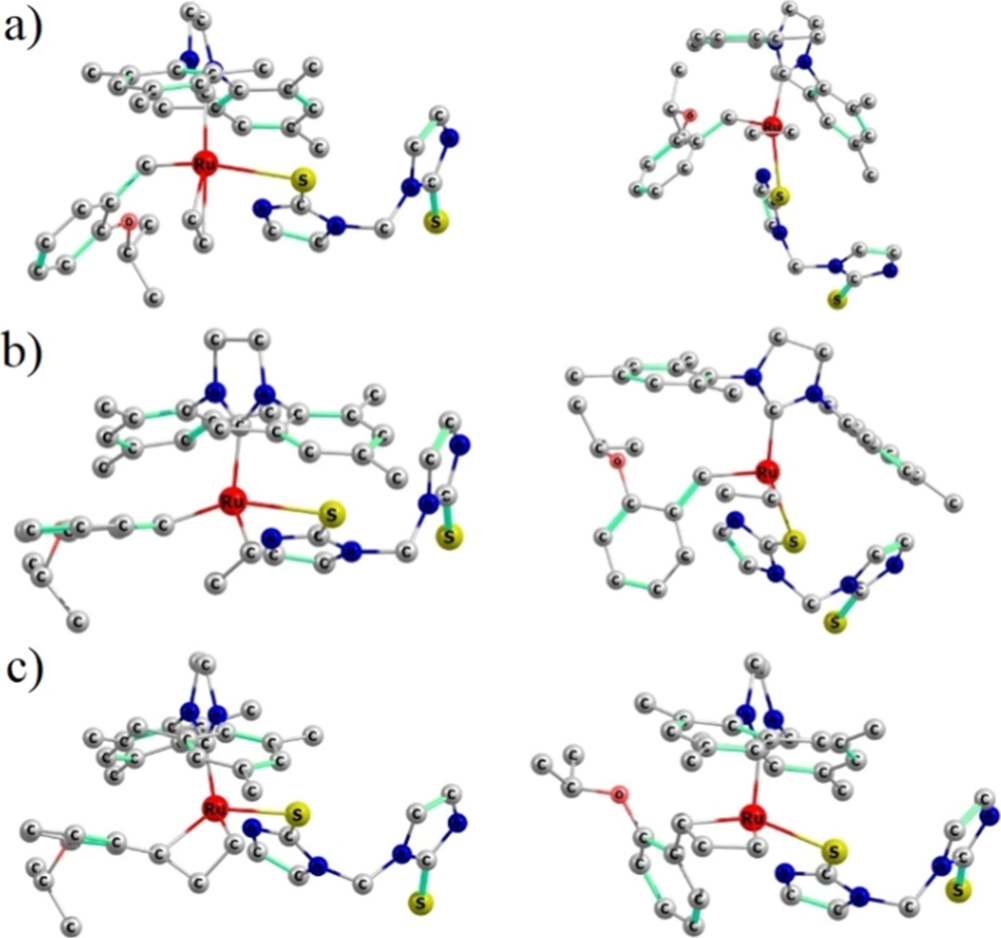
Comparison between bottom-bound (left) and side-bound structures (right) for (a) olefin coordination **4a**, (b) transition state **5a**^‡^, and (c) MCB **6a**.

The reaction described above corresponds to the bottom-bound mechanism, but the side-bound pathway was also studied. Structures related to the 2,2-cycloaddition step for both mechanisms are compared in Figure 4. Both the **4a**_*trans*_ and **4a**_*cis*_ intermediates result in a similar thermodynamic stabilization (ca. −6 kcal/mol), but the energy cost of 31.2 kcal/mol to reach the MCB via **5a**_*cis*_^‡^ is significantly higher than that corresponding to **5a**_*trans*_^‡^ (18.9 kcal/mol). Furthermore, the MCB of the side-bound mechanism, **6a**_*cis*_, is also highly stabilized (−21.2 kcal/mol); so, it likely impedes the course of the reaction, if it is formed. Therefore, even though the subsequent 2,2-cycloreversion step originated from **6a**_*cis*_ reveals stationary points **7a**^‡^ and **8a** that are energetically comparable to those derived from **6a**_*trans*_, it is unlikely that the side-bound mechanism will be observed, as the formation of the MCB **6a**_*cis*_ is kinetically hindered.

### 3.2 Catalytic Activity through the Side-Bound Mechanism

The catalytic performance of **1a** indicates that the incorporation of a sulfurated chelate into an **HG**-type catalyst may be an efficient alternative for the olefin metathesis, and, as a result, we were prompted to explore other structural conformations of the precatalyst. Therefore, all reported Δ*G* values are relative to the energy of **1a**. As a highly stabilized MCB is undesired in olefin metathesis, we performed a comprehensive investigation of the side-bound mechanism, olefin *cis* binding, to evaluate the versatility of the catalyst in question.

**Figure 5:**
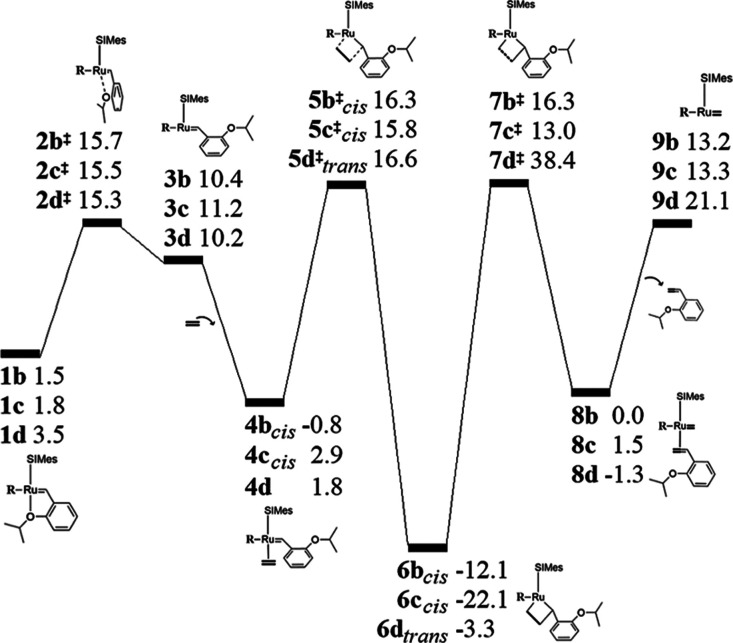
Gibbs free-energy profiles (kcal/mol) of the initiation phase via side-bound pathways **b** and **c** and bottom-bound path **d**. Energy differences are relative to **1a**.

Complex **1b** (φ = 10.9°, *d*_Ru–O_ = 2.37 Å) differs from **1a** mainly in the orientation of the chelate, but both complexes are energetically equivalent. However, for the dissociative step, we only explored the energy surface through φ > 0 because NHC=S located *trans* to the SIMes ligand hampers the rotation in the other direction. The Gibbs energy profiles for **1b–d** are shown in Figure 5, and the structural details for **1b–f** are reported in Table S1 (see the Supporting Information). The energy barrier associated with the dissociative path **b** is 14.2 kcal/mol, calculated using **2b**^‡^ (φ = 35.1° and *d*_Ru–O_ = 3.24 Å). Structure **3b′** is formed at 13.3 kcal/mol and is located at φ = 106.8° and *d*_Ru–O_ = 3.89 Å (not shown in Figure 5); furthermore, continued styrene rotation in the same direction leads to an analogous intermediate **3b** (φ = −144.5° and *d*_Ru–O_ = 4.49 Å), located at 10.4 kcal/mol. Olefin coordination is an exergonic process that releases 2.3 kcal/mol through **4b**_*cis*_, and, after overcoming an energy barrier of 17.1 kcal/mol (**5b**_*cis*_^‡^), **6b**_*cis*_ is formed at −12.1 kcal/mol. Due to the stabilization of the MCB, the energy cost for the 2,2-cycloreversion requires 28.4 kcal/mol via **7b**^‡^. Styrene release occurs at a lower cost, 13.2 kcal/mol, so that a productive olefin metathesis through path **b** is primarily hampered at the rupture of the MCB.

Precatalyst **1c** is structurally and energetically comparable to **1b**, with the main difference being the inversion of methylene that connects both NHC=S units. The description of the side-bound mechanism for **1c** is nearly identical to that of **1b**, both structurally and energetically (see also Table S1). The main difference is the formation of **6c**_*cis*_, which is even more stabilized than **6b**_*cis*_ and, in turn, creates a potential well as the progress or reversion of the reaction requires at least 35 kcal/mol. For this precatalyst conformation, we additionally investigated the bottom-bound path. Consequently, the energy of **4c**_*trans*_ and **5a**_*trans*_^‡^ is similar to that corresponding to *cis*-bound structures, 0.8 and 16.7 kcal/mol, respectively (not shown in Figure 5), yet the MCB **6c**_*trans*_ is located at −3.2 kcal/mol, and the energy barrier via **7c**^‡^ is 20.9 kcal/mol, which can be overcome toward productive metathesis. Although the initiation phase can occur via both side- and bottom-bound, the latter is slightly favored in the olefin coordination step **4c**_*trans*_ by 2.1 kcal/mol. As a result, we infer that the potential well for **6c**_*cis*_ may not be formed.

Complex **1d** is structurally and energetically comparable to **1b** with small differences in the orientation of the NHC=S units. The determining states through path **d** are the MCB **6d**_*trans*_ and **7d**^‡^, which result in a high energy barrier of 41.7 kcal/mol (see Figure 5). Additional pathways are reported in Figure S2 and discussed in the Supporting Information, which are the side-bound mechanism (a pathway where the Ru–O bond is kept during the MCB formation) and two more alternative pathways. However, the four pathways converged to **7d**^‡^ so that the productive olefin metathesis is hindered by path **d**. In this regard, a thermodynamic control in the initiation phase should prevent the formation of **1d** to induce metathesis through stable species.

The structural conformations of **1e** (in this case, the active catalyst **3e** forms two Ru–S bonds) and **1f** were intentionally envisaged to avoid an olefin *trans* binding, although reactions via these complexes can proceed only under a kinetic control due to their relative destabilization, as compared to that of **1a**. Based on the results shown in Table S1 and Figure S3, and a detailed discussion reported in the Supporting Information, we found that the olefin metathesis across complexes **1e** and **1f** is not viable, as the reaction will probably reverse before reaching the respective MCB. In summary, the energy cost associated with the formation of the MCB through an olefin *cis* binding with respect to **1a–f** is in the range of 13 (path **d**) to 31 (path **a**) kcal/mol, which is the energy barrier from **4** to **5**^‡^ (except for path **c**, which is **1** to **2**^‡^). The overall energy cost for the bottom-bound path **a** is 20 kcal/mol, and **6b–f** could also be formed at a lower or similar cost, although the resulting side-bound MCBs must evolve toward affordable paths to achieve productive *Z*-selective metathesis. In this regard, according to the study by Cavallo et al. previously discussed,^87^ the release of the product at the end of the catalytic cycle is the crucial step for stereoselectivity, in agreement with the 2,2-cycloreversions described here. On the contrary, Houk et al.^37^ focused on the formation of MCB and concluded that the side-bound mechanism is faster than the bottom-bound counterpart, which also agrees with the 2,2-cycloaddition analyzed here. However, based on the energetic span model, the energy costs related to Houk’s study,^37^ calculated from the rate-determining states, were 18.7 kcal/mol for the side-bound 2,2-cycloreversion and 17.4 kcal/mol for the bottom-bound 2,2-cycloaddition; these energy barriers are nearly identical so that product release becomes important, in agreement with Cavallo’s analysis.^87^

### 3.3 Analysis of Electronic Properties

**Figure 6:**
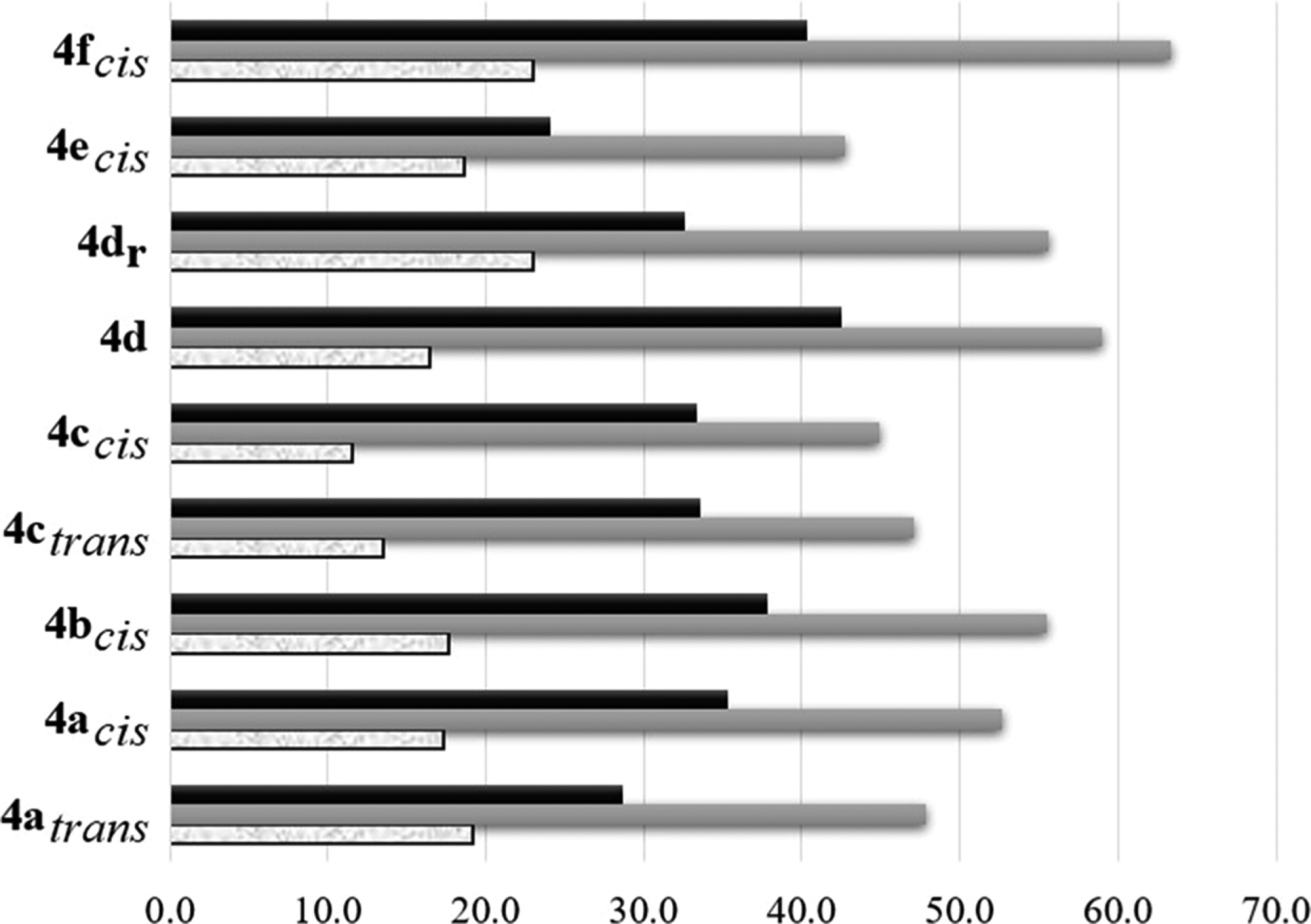
Strain (Δ*E*_strain_, black-filled), interaction (|Δ*E*_int_|, gray-filled), and binding energies (|BE|, textured white) in kcal/mol for the active catalyst coordinated to ethylene.

One of the determining states of the CM corresponds to the olefin coordination to the metal center. In this regard, the activation strain model (ASM)^90−93^ is implemented to reveal interactions between reactants
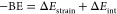
1where Δ*E*_strain_ is the strain energy related to the change in the geometry of reactants to the geometry they acquire in the complex under consideration, and Δ*E*_int_ is the energy gain associated with placing the distorted fragments together. Because of the formation and rupture of covalent bonds associated with the formation and breaking of the MCB, the ASM cannot be applied to the entire catalytic cycle. Consequently, we focus on intermediate **4** to evaluate the balance between the steric and electronic features during the course of the reaction. Applying eq 1 and considering the previous strain interaction scheme, species **4** is divided into the Ru fragment, *f*_1_, and the ethylene moiety, *f*_2_. The olefin η^2^ coordination to the active catalyst causes structural deformation in both fragments; in Figure 6 we report the total Δ*E*_strain_, while detailed values of all parameters are given in Table S2 (see the Supporting Information). For a comprehensive description of the interacting fragments, we identify the complexes that precede an MCB stabilized by at least 10 kcal/mol compared to **1a**; these are the side-bound intermediates **4a–c**_*cis*_, **4d**_**r**_, and **4f**_*cis*_. Except for **4c**_*cis*_, all these complexes show the highest |Δ*E*_int_| and |BE| (above 50 and 17 kcal/mol, respectively), while Δ*E*_strain_ is also relatively high (above 33 kcal/mol). This outcome suggests stronger interactions between the active catalyst and ethylene so that the formation of an MCB is strongly induced, in agreement with the results discussed previously. On the other hand, a moderately stabilized MCB was calculated for the bottom-bound intermediates **4a**_*trans*_, **4c**_*trans*_, **4d**, and side-bound **4e**_*cis*_. Apart from **4d**, these complexes have both |Δ*E*_int_| and Δ*E*_strain_ values below 50 and ca. 33 kcal/mol, respectively, indicating that highly stabilized MCBs may be prevented by moderate electronic interactions and reduced structural distortion of the reactants. Furthermore, the MCB **6d**_*trans*_ formed from **4d** is also moderately stabilized, but this complex shows high values of Δ*E*_int_ and Δ*E*_strain_. Here, we can hypothesize that strain is the main cause of complex destabilization through the **d** pathway, specifically for **7d**^‡^ or similar structures in which structural rearrangements of styrene are to some extent hindered. The lowest |BE| is calculated for both complexes **4c** and **4d**, which is a consequence of the decreased electronic interactions (**c**) or the increased strain (**d**).

As a final remark, during the determination of potential energy surfaces of the dissociative mechanisms reported in this work, we found a structure that can be formed in the decomposition of the catalyst (see Figure S4 in Supporting Information for details), in addition to the potential formation of ruthenium hydrides.^94^ Such a structure involves the η^2^ coordination of the thione C=S bond to Ru, which occurs at an energy cost of only 0.4 kcal/mol as compared to the energy of **1a**. However, we observed that Ru could break the C=S bond to form new NHC=Ru and S(atomic)=Ru bonds. This process is still unfavorable with respect to the exergonic η^2^ coordination of the olefin, but the C=S bond rupture may lead to catalyst decomposition during the initial dissociative step, according to our linear transit calculations. Therefore, we decided to include an additional analysis based on conceptual DFT to evaluate the feasibility of **1** and the active catalyst **3** to be chemically modified
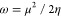
2
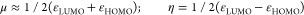
3where ω is the Parr electrophilicity index, and μ and η, respectively, stand for the chemical potential and the molecular hardness, which are defined as the first and second derivatives of the energy with respect to N at a fixed external potential, given an N electron system with total electronic energy *E*.^95−100^ To solve eq 3, Koopmans’ approximation was adopted. These values are reported in Table 2 and are compared to those corresponding to the **HG** catalyst, calculated at the same level of theory. For precatalysts **1a–h** and active catalyst **3a–f**, the variation for ε_H_ and ε_L_ is calculated in the range of −3.93 to −3.22 and −1.42 to −1.21 eV, which are higher values as compared to the respective ε_H_ and ε_L_ in the **HG** catalyst.

**Table 2:** Electronic Properties (in eV) Derived from Conceptual DFT for the Precatalysts and Active Catalysts under Study^a^

species	ε_H_	ε_L_	μ	Η	ω
**1a**	–3.87	–1.33	–2.60	1.27	2.66
**1b**	–3.73	–1.33	–2.53	1.20	2.67
**1c**	–3.92	–1.42	–2.67	1.25	2.85
**1d**	–3.73	–1.36	–2.54	1.19	2.72
**1e**	–3.53	–1.25	–2.39	1.14	2.50
**1f**	–3.88	–1.25	–2.57	1.31	2.50
**1g**	–3.22	–1.30	–2.26	0.96	2.66
**1h**	–3.41	–1.25	–2.33	1.08	2.51
**1**_**HG**_	–5.59	–2.18	–3.88	1.71	4.42
**3a**	–3.86	–1.29	–2.58	1.29	2.58
**3b**	–3.81	–1.42	–2.62	1.19	2.87
**3c**	–3.84	–1.23	–2.54	1.30	2.47
**3d**	–3.66	–1.31	–2.48	1.18	2.62
**3d**_**r**_	–3.65	–1.21	–2.43	1.22	2.42
**3e**	–3.40	–1.33	–2.37	1.04	2.70
**3f**	–3.93	–1.23	–2.58	1.35	2.47
**3**_**HG**_	–5.81	–2.29	–4.05	1.76	4.65

a A comparison with the Hoveyda–Grubbs catalyst is also provided.

The resistance of a system to the change in the number of electrons is evaluated via η, and the energy released when the number of electrons increases due to a more accessible LUMO is assessed with μ. The average value for μ is −2.50 eV, the arithmetic mean validated by the relatively small variation of ε_H_ and ε_L_ and calculated from μ of complexes **1a–h** and **3a–f** reported in Table 2 (excluding μ of **HG**); this is also reflected in the standard deviation σ = 0.12 eV. The arithmetic means for η and ω are 1.20 and 2.61, respectively, and σ < 0.15 eV. Therefore, it is more illustrative to compare these values with those of **HG**. In general, **HG** LUMO is notoriously more accessible due to the decreased value of μ, ca. −4 eV. Complex **1** shows a similar hardness compared to the **HG** catalyst, yet the latter is significantly more electrophilic. The reduced ω in **1** may have an impact on reactions with electron-deficient olefins, although it would also be expected to possess less affinity to other functional groups, preventing catalyst decomposition.^101−103^ However, the results reported in the previous subsection of this work suggest potential catalytic activity of the **HG** complex chelated to a sulfurated pincer in CM.

### 3.4 Evaluation of Stereoselectivity

**Figure 7:**
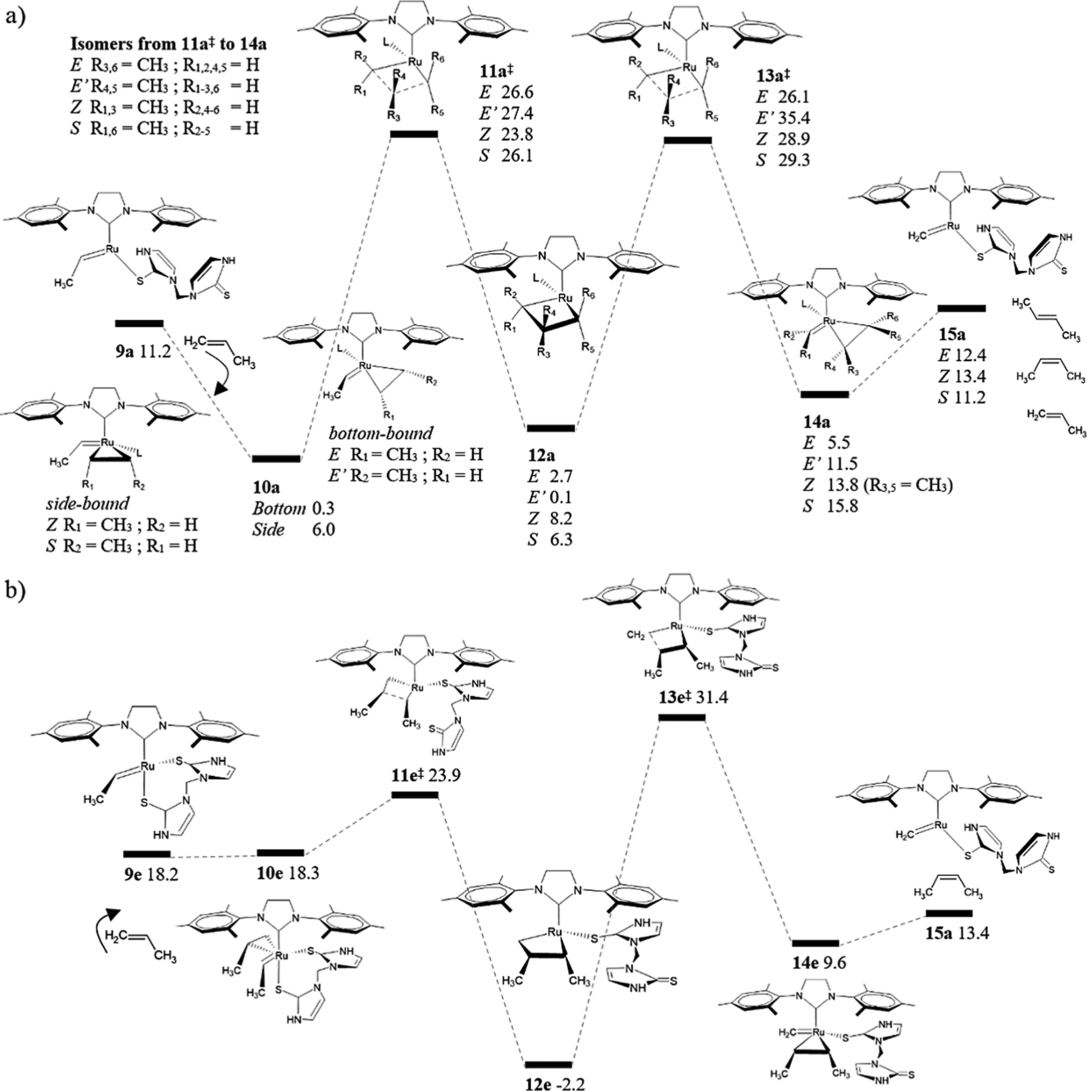
Gibbs free-energy profiles (kcal/mol) of the propagation phase in the metathesis of propene–propene to 2-butene. Energy differences are relative to the precatalyst **1a**.

Our study is concluded with an examination of the propagation phase related to the metathesis of propene–propene to 2-butene and ethylene through five different pathways reported in Figure 7: two of them leading to the *E* isomer, two corresponding to the *Z* isomer, and one for self-metathesis. After the initiation phase, our previous results show that the most stable active catalyst is **9a**, and our analysis continues from here for the case of propene. We assume that the initiation phase with propylene should be energetically similar to that with ethylene; indeed, the energy difference between both the **9a** species is less than 2 kcal/mol. Furthermore, we made additional modifications to **9a** (see Figure S5a in Supporting Information) to confirm the preferred structural conformation of the active catalyst. For the next step, the propene coordination to **9a** leading to **10a**, we optimized 12 conformations and selected two structures based on energy stabilization and proper structural conformation to evaluate the stereoselectivity of the reaction (see Figure S5b in Supporting Information). In the most stable structure for the olefin coordination, propene is coordinated *trans* to the NHC ligand so that it follows a bottom-bound mechanism, **10a**_*trans*_. From this species, we identify two pathways leading to *E* and the *E*′ 2-butene product, which are differentiated by the carbon atom of the olefin that reacts first. The determining states in the path related to *E-*olefin are **10a**_*trans*_ and the 2,2-cycloaddition transition state, **11a**^‡^, with an overall cost of 26.3 kcal/mol. We could not differentiate the transition states and MCBs as *trans* and *cis* structures because the tetracoordinated Ru metal acquires a triangular pyramidal configuration. For the second alternative, the energy barrier that determines the formation of *E*′ 2-butene is 35.3 kcal/mol, which is calculated from the MCB, **12a**, to the 2,2-cycloreversion transition state, **13a**^‡^. The pathway for the formation of the *Z* isomer was investigated from the propene coordination *cis* to the NHC ligand, **10a**_*cis*_, following a side-bound mechanism. The determining states in this case are **10a**_*cis*_ and **13a**^‡^, thus resulting in an energy barrier of 22.9 kcal/mol, which is lower than the respective *E* isomer counterpart. However, **10a**_*cis*_ is destabilized by 5.7 kcal/mol compared to **10a**_*trans*_ so that the formation of *Z-*olefin should be induced under a kinetic control; otherwise, the energy barrier increases from 22.9 to 28.6 kcal/mol (i.e., considering the additional cost to form **10a**_*cis*_ from **10a**_*trans*_). Self-metathesis was also determined from **10a**_*cis*_, and an estimate analogous to the *Z* isomer case reveals an energy cost of 29.0 kcal/mol. The continued metathesis from **15a** through the coordination of styrene to form ethylene, and to recover the catalyst **1a**, is an exergonic process that releases ca. 11 kcal/mol, a favorable driving force that accounts for a proper catalytic activity.

Our results suggest that, without kinetic control, the *E* isomer is formed at a lower energy cost compared to that of the *Z* isomer (26.3 vs 28.6 kcal/mol, respectively). In fact, an energy difference of 2.3 kcal/mol represents an *E*/*Z* ratio of 49:1 at room temperature. We decided to examine another pathway similar to path **e** previously discussed, which is mainly characterized by two Ru–S bonds in the initial species of the catalytic cycle. The two Ru–S bonds induce the formation of *Z-*olefin as a result of stereoselective restrictions. In this case, the active catalyst **9e** and the propene-coordinated structure **10e** are clearly destabilized compared to the respective species in path **a**, but 2,2-cycloaddition through **11e**^‡^ is faster (only 5.7 kcal/mol from the active catalyst **9e**). Furthermore, one of the two Ru–S bonds was dissociated in the species related to MCB **12e**. Consistent with the previous discussion related to Figure S3 in the Supporting Information, the intermediates investigated from the Grubbs carboxylate catalyst depicted in Scheme 2c (analogous to nitrate) resulted in only one Ru–O bond instead of two,^37^ suggesting that the chelating agent links the metal center depending on the electronic environment. The release of *Z* 2-butene via path **e** is kinetically hampered by the stabilization of **12e** and the high energy of the 2,2-cycloreversion transition state, **13e**^‡^. It is observed that the side-bound pathway **a** is also faster at the 2,2-cycloaddition step due to the lowest energy transition state **11a**^‡^, so that the *Z* isomer release is rate-limited at **13a**^‡^ as well. In this regard, we emphasize here that the design of *Z*-selective catalysts is not guaranteed by increasing the rate of 2,2-cycloadditions; as a matter of fact, catalysts reported in Scheme 2 are *Z*-selective probably because of the restrictions of the stereo space related to bulky multiphenyl and adamantyl groups, a steric factor that is not included in the active catalyst **9** that we report here.

## 4 Conclusions

Predictive DFT calculations have been implemented to evaluate the catalytic performance of a Hoveyda–Grubbs catalyst chelated to bis(2-mercaptoimidazolyl)methane (**1**). A detailed description of the thermodynamics and kinetics of the formulated mechanisms revealed the potential applications of this system in CM. We showed that such a new catalyst should allow for an efficient formation of active catalysts as the energy barriers for all the dissociative pathways are lower than 15 kcal/mol. Olefin metathesis through the thermodynamically most stable precatalyst resulted in an overall cost of 20 kcal/mol, obtained considering a bottom-bound pathway in which the olefin binding is *trans* to the SIMes ligand. Under such an energy gap, side-bound 2,2-cycloaddition is not only possible but even kinetically favored compared to the bottom counterpart. However, side-bound MCBs were found to be highly stabilized, precluding the olefin product release, although the olefin *cis* binding will probably be reversed before reaching the respective MCB. Additionally, on the basis of the activation strain model, highly stabilized MCBs may be induced by improved electron interactions between the active catalyst and the olefin, and a reduced strain of the reactants is required in each case. The reduced electrophilicity of **1**, as compared to the Hoveyda–Grubbs catalyst, may represent a disadvantage, but it also indicates a lower affinity for other functional groups, which could prevent decomposition. Finally, the propagation phase in the metathesis of propene–propene to the *Z* isomer of 2-butene resulted in higher rates for the 2,2-cycloaddition step, but the respective olefin release is slower than that of the *E* isomer. However, although the *Z* isomer can be formed under kinetic control, the design of *Z*-selective catalysts should be examined throughout the entire catalytic cycle, as faster 2,2-cycloadditions do not ensure the expected stereoselectivity. We hope that the results of this study may encourage future experimental studies of this new candidate for a catalyst, as well as guide computational studies for the design of new *Z*-selective catalysts.

## Supporting Information Available

Detailed structural information, energies, and Cartesian coordinates for all studied systems (PDF)
