# Supplementary material for: Olefin Metathesis Catalyzed by a Hoveyda–Grubbs-like Complex Chelated to Bis(2-mercaptoimidazolyl) Methane: A Predictive DFT Study
Source: J Phys Chem A. 2022 Jan 26;126(5):720–32. doi: 10.1021/acs.jpca.1c09336 (PMC8842278; doi:10.1021/acs.jpca.1c09336)

Supporting Information

Olefin metathesis catalyzed by a Hoveyda-Grubbs-like complex chelated to bis(2-mercaptoimidazolyl) methane: a predictive DFT study

J. Pablo Martínez[§]*

orcid.org/0000-0002-6589-790X; email: p.martinez@cent.uw.edu.pl

Bartosz Trzaskowski[§]*

orcid.org/0000-0003-2385-1476; email: b.trzaskowski@cent.uw.edu.pl

[§] *Centre of New Technologies, University of Warsaw, 02-097 Warszawa, Poland*

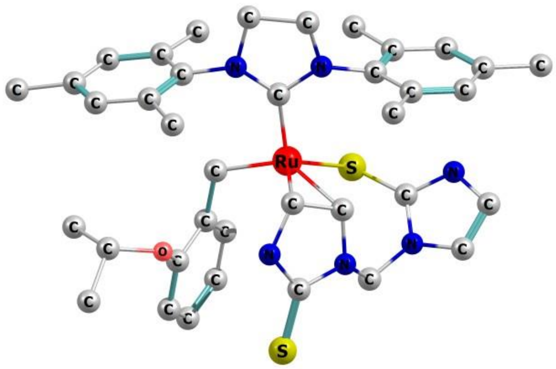

Fig. S1 Alternative C=C coordination to the metal.

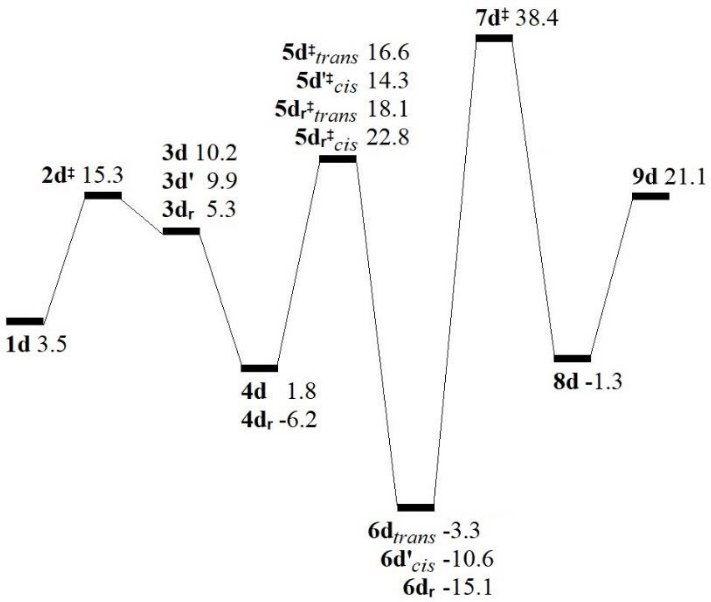

Fig. S2 Relative Gibbs free energies profiles (kcal/mol) of the initiation phase for precatalyst **1d**. Energy differences are relative to complex **1a**. Both paths via **d$_{r}$** converged to the same MCB **6d$_{r}$**.

The dissociative path reported in Figure 5 in the main manuscript corresponds to φ < 0, which may be counterintuitive because the isopropoxy group hinders the styrene rotation in this direction. However, the energy barrier associated with this step is only 11.8 kcal/mol, which is calculated via **2d**‡ (φ = -69.6°, $d_{Ru\text{-}O}$ = 3.75 Å). A continued styrene rotation leads to the structure **3d'** localized at 9.9 kcal/mol (φ = -113.5°, $d_{Ru\text{-}O}$ = 4.21 Å), followed by **3d** (φ = 176.8°, $d_{Ru\text{-}O}$ = 4.66 Å). In the case of incomplete rotation of the styrene (these are not shown in Figure 5 and are denoted as **xd'**, *x* = 3 to 6), the isopropoxy group is located below the ruthenium atom (in standard orientation with NHC located above the ruthenium atom) in **4d** and, based on linear transit calculations, we observed that the side- and bottom-bound mechanisms depend on the ethylene carbon that reacts first. In the case of **5d'**$^{‡}_{cis}$, the isopropoxy group was spontaneously linked to ruthenium again (φ = 15.9°, $d_{Ru\text{-}O}$ = 2.48 Å). On the other hand, the analogous transition state **5d**$^{‡}_{trans}$ prevents the styrene back-rotation (φ = 167.9°, $d_{Ru\text{-}O}$ = 4.70 Å). The structural descriptors d_1, d_2, and d_3 are similar for both transition states, but the energy barrier from **4d** to **5d'**$^{‡}_{cis}$ is slightly lower (12.5 compared to 14.8 kcal/mol with **5d**$^{‡}_{trans}$, see Figure S2). On the other hand, **6d'**$_{cis}$ is highly stabilized by 14.1 kcal/mol compared to **1d**. Attempts to localize **7d'**‡ from **6d'**$_{cis}$ were unsuccessful, since the Ru-O bond dissociates during the linear-transit calculations. The high stabilization of **6d'**$_{cis}$ represents an important drawback from a practical point of view, since the course of the reaction may fall into a potential well: reversion via side **5d'**$^{‡}_{cis}$ requires 24.9 kcal/mol, and progression to active catalyst **9d** would require at least 31 kcal/mol. In contrast, even though MCB **6d**$_{trans}$ is moderately stabilized by 6.8 kcal/mol compared to **1d**, the 2,2-cycloreversion is blocked by a large energy barrier of 41.7 kcal/mol, according to **7d**‡. Furthermore, we investigated an alternative pathway starting from the species **3d**$_{\mathbf{r}}$, where the subscript "r" stands for rotated styrene at φ = -140.8° and $d_{Ru\text{-}O}$ = 4.38 Å. Although **5d**$_{\mathbf{r}}^{‡}{}_{trans}$ is 4.7 kcal/mol lower than the analogous **5d**$_{\mathbf{r}}^{‡}{}_{cis}$ (details in Figure S2), the resulting MCB **6d**$_{\mathbf{r}}$ is even more stabilized by 18.6 kcal/mol as compared to **1d**. Therefore, these results suggest that productive olefin metathesis is hindered by path **d**.

Table S1 Evolution of structural parameters as defined in Scheme 3 through the initiation phase for complex **1b-f**. Torsional angle, φ, in degrees and bond distances, d_n, in Å.

ξ	φ	d_{Ru-O}	d_1	d_2	d_3
**1b**	10.9	2.375			
**2b**‡	35.1	3.236			
**3b'**	106.8	3.888			
**3b**	-144.5	4.495			
**4b***cis*	170.1	4.687	2.168	3.059	1.422
**5b**‡*cis*	-153.9	4.737	2.340	2.182	1.432
**6b***cis*	-112.4	4.066	2.709	1.535	1.537
**7b**‡	-123.6	4.438	2.311	1.454	2.249
**8b**	-114.3	4.392	2.228	1.418	2.883
**9b**	-	3.711	5.143	1.340	5.031

ξ	φ	d_{Ru-O}	d_1	d_2	d_3
**1c**	8.1	2.376			
**2c**‡	94.9	3.841			
**3c'**	120.6	4.114			
**3c**	-124.9	4.216			
**4c***cis*	126.1	4.227	2.213	2.924	1.399
**4c***trans*	-119.9	4.231	2.197	3.150	1.394
**5c**‡*cis*	-148.1	4.595	2.335	2.110	1.442
**5c**‡*trans*	161.9	4.642	2.361	2.183	1.443
**6c***cis*	-109.9	4.016	2.710	1.534	1.537
**6c***trans*	101.4	4.272	2.586	1.553	1.535
**7c**‡*cis*	-119.6	4.307	2.320	1.460	2.229
**7c**‡*trans*	116.0	4.525	2.309	1.461	2.203
**8c***cis*	-101.6	4.103	2.205	1.424	2.795
**8c***trans*	152.4	4.879	2.212	1.429	2.785
**9c**	-	6.164	4.908	1.340	5.555

Table S1 *Continued…*

ξ	φ	$d_{Ru\text{-}O}$	d_1	d_2	d_3
**1d**	12.7	2.425			
**2d‡**	-69.6	3.748			
**3d'**	-113.5	4.213			
**3d**	176.8	4.659			
**4d**	-99.2	4.083	2.170	3.002	1.426
**5d‡***trans*	167.9	4.703	2.319	2.141	1.466
**5d'‡***cis*	15.9	2.484	2.354	2.151	1.435
**6d***trans*	80.9	3.932	2.704	1.524	1.534
**6d'***cis*	32.7	2.422	2.661	1.565	1.532
**7d‡**	102.0	4.209	2.976	1.407	2.515
**8d**	103.6	4.104	2.131	1.443	2.840
**9d**	-	2.500	6.038	1.340	5.059

**3d$_r$**	-140.8	4.378			
**4d$_r$**	-147.5	4.459	2.226	3.620	1.419
**5d$_r$‡***cis*	-136.1	4.414	2.319	2.122	1.443
**5d$_r$‡***trans*	-138.5	4.440	2.308	2.142	1.456
**6d$_r$**	-114.2	4.125	2.723	1.536	1.540

Subscript *r* stands for an alternative rotation of the styrene fragment.

Table S1 *Continued…*

ξ	φ	$d_{Ru\text{-}O}$	d_1	d_2	d_3
**1e**	2.7	2.302			
**2e**	69.7	3.688			
**3e'**	126.7	4.485			
**3e**	-125.4	4.367			
**4e**$_{cis}$	-131.4	4.460	2.228	3.329	1.404
**5e**$^{‡}_{cis}$	-148.6	4.569	2.301	2.138	1.443
**6e**$_{cis}$	-98.5	4.223	2.702	1.549	1.532
**7e**‡	-136.9	4.598	2.298	1.453	2.211
**8e**					
**9e**	-	9.082	7.740	1.342	8.871

ξ	φ	$d_{Ru\text{-}O}$	d_1	d_2	d_3
**1f**	-14.1	2.341			
**2f**	-67.4	3.116			
**3f'**	-118.0	4.130			
**3f**	121.8	4.142			
**4f**‡	144.2	4.494	3.360	3.785	1.337
**4f**$_{cis}$	-159.3	4.573	2.147	2.973	1.438
**5f**$^{‡}_{cis}$	177.9	4.742	2.260	1.930	1.491
**6f**$_{cis}$	112.9	4.258	2.683	1.548	1.530
**7f**‡	87.7	4.032	2.276	1.476	1.994
**8f**	102.0	4.173	2.142	1.442	2.820
**9f**	-	4.470	6.492	1.342	5.764

Pathways via 1e and 1f

In the case of complex **1e**, styrene rotation occurs via **2e** (φ = 69.7°, $d_{Ru\text{-}O}$ = 3.69 Å), which is characterized as a local minimum at an energy cost of 10.3 kcal/mol (see Fig. S3). During styrene rotation towards **3e**, both thiones were bonded to Ru and both Ru-S bonds were kept until the MCB. Ethylene coordination releases 1.3 kcal/mol via **4e**$_{cis}$, and the energy barrier calculated with **5e**$^{‡}_{cis}$ to form the MCB is 17.2 kcal/mol. Even though **6e**$_{cis}$ is less stabilized than side-bound **6a-c**, the product release is hampered by 31.7 kcal/mol at the 2,2-cycloreversion step, despite **7e**‡ is only 16.1 kcal/mol above the precatalyst **1e**. Geometry optimizations of **7e**‡ resulted in the rupture of one Ru-S bond. We assumed the reaction from **6e**$_{cis}$ may proceed with only one Ru-S bond; but it would be probably reverted through the dissociative step before MCB formation. Additionally, considering the reaction mechanisms formulated by Houk et al. (*J. Am. Chem. Soc.* 2012, 134, 1464) for the Grubbs-carboxylate catalyst shown in Scheme 2c (analogous to nitrate), some intermediate species resulted in only one Ru-O bond, which suggests the chelating agent links the metal centre depending on the electronic environment.

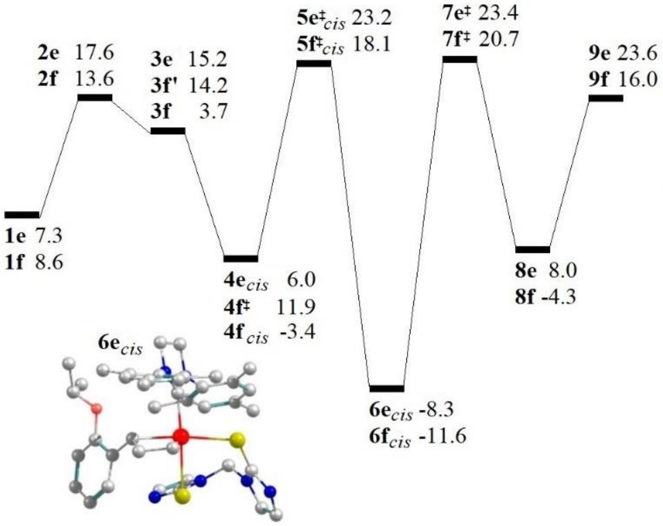

Fig. S3 Gibbs free energies profiles (kcal/mol) of the initiation phase for complexes **1e** and **1f**. Energy differences are relative to **1a**.

In the case of **1f**, the higher energy structure though the dissociative path is **3f'** (φ = -118.0°, $d_{Ru\text{-}O}$ = 4.13 Å) instead of **2f** (φ = -67.4°, $d_{Ru\text{-}O}$ = 3.12 Å), both are local minima, and the resulting energy barrier is therefore only 5.6 kcal/mol. Continued rotation is an exergonic process leading to intermediate **3f** (φ = 121.8°, $d_{Ru\text{-}O}$ = 4.14 Å), and releasing 4.9 kcal/mol. We additionally localized a transition state related to the η^2-coordination of ethylene to form **4f**$_{cis}$, which adds a second barrier of 8.2 kcal/mol via **4f**‡ as compared to **3f**. The formation of **4f**$_{cis}$ releases 12.0 kcal/mol and the energy cost associated to the formation of a MCB is 21.5 by means of **5f**$^{‡}_{cis}$, yet the MCB **6f**$_{cis}$ is highly stabilized by 20.2 kcal/mol. Nonetheless, we observed that reversion of the reaction from the olefin coordination step towards **1f** occurs at a lower energy cost. We conclude therefore that olefin metathesis across complex **1f** is not viable since the reaction will be probably reverted before reaching the respective MCB.

Table S2 Strain (ΔE_{strain}),* interaction (ΔE_{int}), and binding energies (*BE*) in kcal/mol for the active catalysts (fragment f_1) under study coordinated to ethylene (f_2). Electronic energies calculated at (PB-SC-PCM:*toluene*)M06-D3/LACV3P++**//B3LYP-D3/LACVP**.

Species	$\Delta E_{str\text{-}f1}$	$\Delta E_{str\text{-}f2}$	ΔE_{strain}	ΔE_{int}	|BE|
**4a**$_{trans}$	21.02	7.69	28.71	-47.87	19.16
**4a**$_{cis}$	22.53	12.84	35.37	-52.69	17.32
**4b**$_{cis}$	24.68	13.13	37.81	-55.44	17.63
**4c**$_{trans}$	25.95	7.62	33.57	-47.06	13.49
**4c**$_{cis}$	24.99	8.31	33.30	-44.86	11.56
**4d**	27.16	15.30	42.46	-58.94	16.48
**4d**$_{r}$	20.38	12.20	32.58	-55.61	23.03
**4e**$_{cis}$	14.41	9.68	24.08	-42.75	18.67
**4f**$_{cis}$	20.73	19.55	40.28	-63.29	23.01

* Strain is evaluated considering distortion of precatalyst **1**.

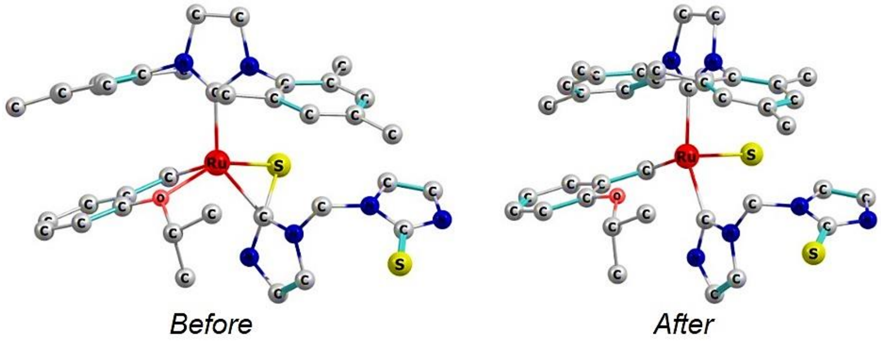

Fig. S4 Coordination of the thione C=S bond to Ru followed by catalyst decomposition.

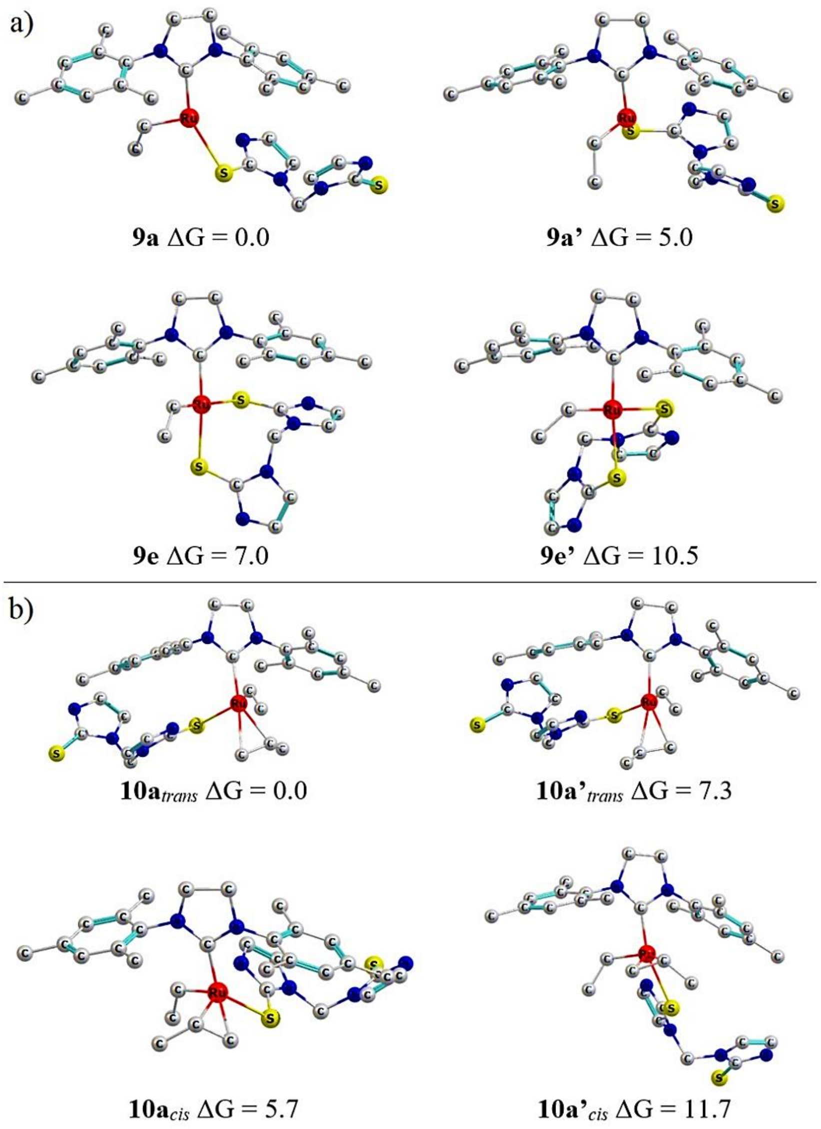

Fig. S5 3D representation of DFT-optimized geometries of a) active catalyst **9**, and b) olefin coordination corresponding to the propagation phase. Gibbs energy comparisons are given in kcal/mol for each case. Structures to the left are the ones discussed in the main manuscript. Hydrogen atoms are hidden for the sake of clarity.

Table S3 Total energy values (E) and Gibbs free energy values (G; as defined in the manuscript) for structures reported in Table S1 in the same order, along with Cartesian coordinates and its corresponding 3D view of optimized geometries. Species used for the analysis of stereoselectivity are also included consecutively.

Species	*gas-phase* B3LYP-D3/LACVP**			*solvated* M06-D3/LACV3P++**		M06//B3LYP
	ZPE (kcal/mol)	*S* (cal/mol)	*H* (kcal/mol)	E_{gas} (a.u.)	E_{solv} (kcal/mol)	G_{solv} (kcal/mol)
**1a**	487.695	282.000	31.097	-2768.860014	-1.7939	-1737054.427
**2a**‡	487.414	287.006	31.334	-2768.834193	-2.1463	-1737040.113
**3a'**	486.715	287.199	31.122	-2768.827696	-2.3037	-1737037.163
**3a**	487.812	284.998	31.264	-2768.840739	-2.2677	-1737043.416
**4a**$_{trans}$	522.232	297.407	33.437	-2847.425848	-2.4487	-1786323.645
**5a**$^{‡}_{trans}$	521.323	296.682	32.686	-2847.394068	-2.0104	-1786304.709
**6a**$_{trans}$	523.543	287.474	32.021	-2847.421393	-2.2068	-1786317.752
**7a**‡	521.325	294.878	32.624	-2847.392770	-2.3032	-1786303.709
**8a**	521.412	297.471	33.085	-2847.417505	-2.1249	-1786319.278
**9a**	520.867	302.795	33.740	-2847.397570	-2.2028	-1786308.323
**9a'**	520.139	306.563	34.154	-2847.382516	-1.9649	-1786300.077
**4a**$_{cis}$	522.167	302.577	33.581	-2847.422651	-2.6113	-1786323.265
**5a**$^{‡}_{cis}$	522.550	295.490	33.042	-2847.376893	-2.1328	-1786292.115
**6a**$_{cis}$	524.358	290.171	32.745	-2847.456383	-2.2213	-1786338.988
**7a**‡	521.425	299.061	33.203	-2847.394757	-1.7459	-1786304.967
**8a**	521.936	302.549	33.546	-2847.409937	-2.6022	-1786315.535
**1b**	488.155	282.289	31.219	-2768.856583	-2.9845	-1737052.969
**2b**‡	487.753	287.711	31.426	-2768.831133	-2.9158	-1737038.742
**3b'**	487.647	294.834	32.047	-2768.833584	-2.1687	-1737041.142
**3b**	488.175	289.539	31.816	-2768.842569	-1.3112	-1737044.047
**4b**$_{cis}$	523.267	290.474	32.900	-2847.421610	-2.6124	-1786318.585
**5b**$^{‡}_{cis}$	522.475	282.350	32.082	-2847.397051	-1.8021	-1786301.551
**6b**$_{cis}$	524.728	281.122	31.883	-2847.445660	-2.1124	-1786329.944
**7b**‡	522.373	291.782	32.617	-2847.393338	-1.6721	-1786301.470
**8b**	522.206	298.568	33.375	-2847.415400	-2.7399	-1786317.814
**9b**	520.627	304.415	33.688	-2847.390989	-1.8064	-1786304.572

Species	*gas-phase* B3LYP-D3/LACVP**			*solvated* M06-D3/LACV3P++**		M06//B3LYP
	ZPE (kcal/mol)	*S* (cal/mol)	*H* (kcal/mol)	E_{gas} (a.u.)	E_{solv} (kcal/mol)	G_{solv} (kcal/mol)
**1c**	488.028	286.896	31.575	-2768.856717	-1.4085	-1737052.622
**2c**‡	486.760	293.093	31.627	-2768.829413	-1.7545	-1737038.898
**3c'**	487.319	297.853	32.031	-2768.834388	-2.1130	-1737042.835
**3c**	487.743	293.362	31.883	-2768.837608	-2.1088	-1737043.236
$\mathbf{4c}_{cis}$	521.739	299.701	33.296	-2847.409932	-2.3820	-1786314.909
$\mathbf{4c}_{trans}$	521.517	299.322	33.228	-2847.413130	-2.3031	-1786317.014
$\mathbf{5c}^{‡}_{cis}$	521.423	295.576	32.811	-2847.391214	-1.6913	-1786302.044
$\mathbf{5c}^{‡}_{trans}$	521.150	298.190	32.836	-2847.388470	-1.4064	-1786301.064
$\mathbf{6c}_{cis}$	524.068	295.040	32.929	-2847.456129	-1.7160	-1786339.881
$\mathbf{6c}_{trans}$	523.624	293.043	32.554	-2847.425150	-2.0559	-1786321.004
$\mathbf{7c}^{‡}_{cis}$	521.856	297.136	33.102	-2847.395533	-2.0368	-1786304.841
$\mathbf{7c}^{‡}_{trans}$	521.394	294.436	32.725	-2847.387992	-1.9542	-1786300.060
$\mathbf{8c}_{cis}$	522.384	292.099	32.875	-2847.415927	-2.3089	-1786316.107
$\mathbf{8c}_{trans}$	522.124	299.810	33.423	-2847.413783	-1.8253	-1786316.290
**9c**	520.797	305.347	33.801	-2847.390454	-2.1005	-1786304.526
**1d**	488.680	281.213	31.303	-2768.854235	-3.3944	-1737050.976
**2d**‡	487.822	284.083	31.328	-2768.832857	-3.2290	-1737039.085
**3d'**	487.989	287.611	31.700	-2768.840018	-3.6653	-1737044.527
**3d**	488.447	289.990	31.805	-2768.840223	-3.1271	-1737044.264
**4d**	522.656	286.287	32.611	-2847.416357	-3.7022	-1786316.030
$\mathbf{5d}^{‡}_{trans}$	522.755	290.966	32.649	-2847.391720	-3.0751	-1786301.201
$\mathbf{5d}'^{‡}_{cis}$	522.641	292.248	32.891	-2847.394983	-3.0954	-1786303.523
$\mathbf{6d}_{trans}$	524.123	291.023	32.448	-2847.425415	-2.9512	-1786321.071
$\mathbf{6d}'_{cis}$	524.103	296.376	32.996	-2847.434807	-3.2793	-1786328.361
**7d**‡	520.723	296.644	33.292	-2847.352882	-2.5899	-1786279.426
**8d**	522.619	297.369	33.218	-2847.418198	-2.8699	-1786319.087
**9d**	521.707	301.325	33.778	-2847.379815	-3.0672	-1786296.730

Species	gas-phase B3LYP-D3/LACVP**			solvated M06-D3/LACV3P++**		M06//B3LYP
	ZPE (kcal/mol)	*S* (cal/mol)	*H* (kcal/mol)	E_{gas} (a.u.)	E_{solv} (kcal/mol)	G_{solv} (kcal/mol)
**3d$_r$**	487.958	285.942	31.376	-2768.849732	-2.3530	-1737049.168
**4d$_r$**	522.458	291.773	33.023	-2847.427024	-3.5521	-1786323.995
**5d$_r$$^{‡}_{cis}$**	522.155	288.380	32.595	-2847.382408	-2.8461	-1786295.011
**5d$_r$$^{‡}_{trans}$**	522.632	289.486	32.686	-2847.391190	-2.3054	-1786299.743
**6d$_r$**	524.587	294.807	32.833	-2847.444241	-2.7323	-1786332.945
**1e**	487.641	285.935	31.728	-2768.846434	-2.4436	-1737047.152
**2e**	487.314	282.630	31.422	-2768.829605	-3.0231	-1737036.819
**3e'**	487.023	285.324	31.605	-2768.843506	-4.1103	-1737047.540
**3e**	487.776	273.300	30.739	-2768.837643	-2.9506	-1737039.229
**4e**$_{cis}$	523.360	287.135	32.754	-2847.410849	-3.4955	-1786311.772
**5e**$^{‡}_{cis}$	522.400	285.173	32.652	-2847.384191	-2.5603	-1786294.586
**6e**$_{cis}$	524.707	285.353	32.572	-2847.437299	-2.9052	-1786326.083
**7e**‡	521.293	300.371	33.411	-2847.374702	-3.4864	-1786294.437
**8e**	522.646	292.837	33.248	-2847.406811	-4.503	-1786312.166
**9e**	520.921	292.587	32.563	-2847.377536	-2.5462	-1786294.175
**1f**	487.899	274.076	30.636	-2768.849583	-1.8400	-1737045.822
**2f**	486.362	280.737	31.157	-2768.836631	-2.0110	-1737040.868
**3f'**	487.135	292.714	31.977	-2768.832700	-1.8193	-1737040.188
**3f**	487.678	289.331	31.909	-2768.849084	-3.5391	-1737050.705
**4f**‡	521.079	308.440	34.197	-2847.391269	-2.7072	-1786305.888
**4f**$_{cis}$	522.421	299.102	33.510	-2847.422623	-1.8267	-1786321.243
**5f**$^{‡}_{cis}$	521.798	290.501	32.593	-2847.390721	-1.2874	-1786299.661
**6f**$_{cis}$	524.850	289.066	32.647	-2847.442797	-1.8712	-1786329.389
**7f**‡	521.936	292.032	32.718	-2847.386417	-1.1959	-1786297.062
**8f**	521.635	288.310	32.413	-2847.426166	-1.7489	-1786322.054
**9f**	520.529	303.017	33.767	-2847.388166	-1.1778	-1786301.775
**1g**	487.305	279.773	31.451	-2768.830174	-3.4267	-1737036.707
**1h**	487.657	281.702	31.475	-2768.827055	-2.8689	-1737034.392

Stereoselectivity

Species	*gas-phase* B3LYP-D3/LACVP**			*solvated* M06-D3/LACV3P++**		M06//B3LYP
	ZPE (kcal/mol)	*S* (cal/mol)	*H* (kcal/mol)	E_{gas} (a.u.)	E_{solv} (kcal/mol)	G_{solv} (kcal/mol)
**9a**	397.323	254.836	26.899	-2384.112912	-2.1888	-1495708.639
**9a'**	397.806	249.568	26.723	-2384.106657	-3.0125	-1495703.660
**10a**$_{trans}$	450.030	268.015	29.362	-2501.989940	-1.8389	-1569626.063
**10a'**$_{trans}$	449.879	261.958	28.867	-2501.980252	-1.8217	-1569618.806
**10a**$_{cis}$	450.336	266.529	29.196	-2501.981431	-2.0743	-1569620.375
**10a'**$_{cis}$	449.913	270.192	29.316	-2501.969593	-2.0500	-1569614.318
**11a**$^{‡}_{path\ E}$	449.828	256.285	27.875	-2501.950972	-1.7915	-1569599.755
**12a**$_{path\ E}$	452.275	266.436	28.651	-2501.989299	-1.7844	-1569623.601
**13a**$^{‡}_{path\ E}$	449.708	265.379	28.529	-2501.948865	-1.4108	-1569600.229
**14a**$_{path\ E}$	449.934	270.537	29.377	-2501.979440	-2.3655	-1569620.833
**11a**$^{‡}_{path\ E'}$	449.896	263.226	28.461	-2501.948027	-1.4327	-1569598.963
**12a**$_{path\ E'}$	452.094	261.113	28.277	-2501.994751	-2.0553	-1569626.261
**13a**$^{‡}_{path\ E'}$	449.994	254.843	27.791	-2501.938013	-1.6394	-1569590.958
**14a**$_{path\ E'}$	450.339	265.913	29.076	-2501.972613	-2.1832	-1569614.885
**11a**$^{‡}_{path\ Z}$	450.052	265.339	28.872	-2501.953562	-1.5015	-1569602.568
**12a**$_{path\ Z}$	452.870	251.807	27.671	-2501.986201	-2.1992	-1569618.096
**13a**$^{‡}_{path\ Z}$	450.680	255.046	28.324	-2501.950525	-1.4084	-1569597.420
**14a**$_{path\ Z}$	451.102	258.630	28.690	-2501.973315	-1.9582	-1569612.552
**11a**$^{‡}_{path\ S}$	450.224	258.305	28.265	-2501.952196	-1.6555	-1569600.203
**12a**$_{path\ S}$	452.489	259.157	28.085	-2501.986320	-1.8746	-1569620.004
**13a**$^{‡}_{path\ S}$	450.569	254.738	27.968	-2501.948775	-1.7070	-1569596.996
**14a**$_{path\ S}$	450.799	265.179	29.027	-2501.965091	-3.1459	-1569610.497
**15a**	*This structure corresponds to **9a** generated with ethylene in the initiation phase, already reported above					

Species	*gas-phase* B3LYP-D3/LACVP**			*solvated* M06-D3/LACV3P++**		M06//B3LYP
	ZPE (kcal/mol)	*S* (cal/mol)	*H* (kcal/mol)	E_{gas} (a.u.)	E_{solv} (kcal/mol)	G_{solv} (kcal/mol)
**9e**	397.292	246.258	26.452	-2384.101623	-4.3780	-1495701.665
**9e'**	396.961	243.902	26.033	-2384.095200	-4.8549	-1495698.159
**10e**	450.261	261.593	29.023	-2501.959780	-4.5137	-1569608.005
**11e**‡	449.794	263.665	28.890	-2501.949723	-3.9996	-1569602.398
**12e**	452.466	261.522	28.537	-2501.997317	-3.1968	-1569628.503
**13e**‡	450.476	262.486	28.699	-2501.943445	-1.3160	-1569594.932
**14e**	450.195	266.293	29.201	-2501.975318	-2.2508	-1569616.782

ethene	32.065	52.323	2.501	-78.539005	1.6695	-49263.376
styrene	140.059	104.702	7.933	-502.556887	1.6512	-315241.046
propene	50.103	63.151	3.145	-117.835434	2.0143	-73906.479
E 2-butene	67.867	71.054	4.011	-157.131053	2.3523	-98548.261
Z 2-butene	67.970	71.837	4.017	-157.129541	2.4379	-98547.351

1a

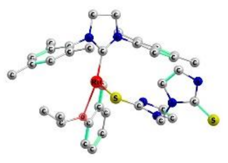

94
RuC38N6H46S2O

Ru	-0.802345681	1.154985115	1.150715737
C	-0.870892640	0.896005701	-0.715521966
H	-0.828546873	1.635591386	-1.520990074
C	-0.717617009	-0.470429896	-1.217544383
C	-0.716013327	-1.550073964	-0.303522028
C	-0.544584755	-2.867352697	-0.712008872
C	-0.386718083	-3.147805571	-2.073390391
C	-0.389784716	-2.107771812	-3.005853657
C	-0.545136390	-0.790013888	-2.579716835
H	-0.527362691	-3.671524277	0.016463771
H	-0.254539862	-4.176484894	-2.396861023
H	-0.263570925	-2.325968559	-4.063190865
H	-0.538139357	0.023644173	-3.301944044
O	-0.846946053	-1.187250637	1.031687492
C	-1.787633470	-1.946669028	1.853696051
C	-3.210273048	-1.744281882	1.343344024
C	-1.595536917	-1.478000313	3.287481344
H	-1.505453227	-3.004160915	1.784572133
H	-3.301999845	-2.069916220	0.303315324
H	-3.913171536	-2.323012752	1.952588929
H	-3.471742348	-0.684443949	1.405163013
H	-0.544679736	-1.545830594	3.578090761
H	-1.923669876	-0.440812879	3.407400555
H	-2.195933817	-2.102620704	3.958152127
C	-1.162410202	3.062003779	1.121223122
N	-0.799648911	4.135137667	0.335559579
C	-1.271767123	5.424314072	0.864136441
C	-2.378082138	4.990942372	1.836970630
N	-2.018405757	3.592228161	2.071820145
H	-0.457272746	5.950789684	1.382846985
H	-1.637272319	6.072553740	0.061144078
H	-2.379011194	5.569327793	2.767136703
H	-3.376426041	5.075539174	1.382641477
C	0.243321269	4.118716048	-0.638887615
C	-0.118327566	4.113101335	-1.998953763
C	-1.575379762	4.116118233	-2.397613089
C	0.893000282	4.098783912	-2.960985183
C	2.245806565	4.107077070	-2.601776819
C	3.320120943	4.104425732	-3.665092726
C	2.573482780	4.141034352	-1.244126090
C	1.590869248	4.154454395	-0.246539128
C	1.978643838	4.208776855	1.210885587
H	-2.048519766	5.078163167	-2.163854990
H	-2.133340846	3.346491775	-1.856757175
H	-1.689529349	3.942305679	-3.471338953
H	0.621086617	4.074770667	-4.014540843
H	3.435609373	5.099392061	-4.113294819
H	3.074996238	3.412769227	-4.478447509
H	4.292408592	3.814774577	-3.255032669
H	3.620267804	4.145738217	-0.947600194
H	1.832211587	5.216225234	1.621736761
H	1.378949386	3.515884018	1.805294043
H	3.033032184	3.951204670	1.346173326
C	-2.751565163	2.733479246	2.947289317
C	-2.270459565	2.554514861	4.256773782
C	-1.036532028	3.292336632	4.718750237
C	-2.956554977	1.684286104	5.111094275
C	-4.102029318	1.002943960	4.692974477
C	-4.784694341	0.005473015	5.599171898
C	-4.577059210	1.226332999	3.395102953
C	-3.922341023	2.081981840	2.506473543
C	-4.425188728	2.268390490	1.096012946
H	-0.756238753	2.990515663	5.731928720
H	-0.192655787	3.094401181	4.051628791
H	-1.201640197	4.376658519	4.722399545
H	-2.581271637	1.529376330	6.120544149
H	-4.401813489	-1.007003476	5.415512445
H	-4.610584707	0.233224417	6.655395909
H	-5.865873641	-0.020766054	5.428838657
H	-5.470492069	0.704076549	3.058965332
H	-4.655982874	3.317833949	0.880319499
H	-3.654439494	1.941827693	0.388237250
H	-5.332998646	1.683158915	0.923783732
S	1.176994381	0.872508239	2.902073856
C	2.406136884	0.866410406	1.709056701
N	2.212583047	1.024164573	0.385035042
C	3.416496223	1.015140701	-0.294560317
C	4.395865136	0.833428583	0.626400221
N	3.761485192	0.725023463	1.869418563
H	1.224413069	1.184742985	0.064627716
H	3.464953613	1.144047339	-1.362978006
H	5.471250693	0.789847505	0.550542385
C	4.437166373	0.687491851	3.150204979
H	5.349588171	0.096616239	3.059805891
H	3.756975590	0.248205489	3.881054205
S	7.399896721	1.904051533	2.604669741
C	6.045644020	2.590323440	3.329553612
N	4.816681293	2.016772160	3.588589790
C	3.947596397	2.945241383	4.165315983
C	4.636950963	4.100964430	4.301442267
N	5.911525922	3.869223575	3.796147055
H	2.923675424	2.684773133	4.384952935
H	4.339574571	5.054903595	4.705443902
H	6.673043773	4.527712088	3.745671448

2a‡ (16.61*i* cm^{-1})

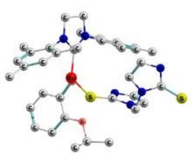

94
RuC38N6H46S2O

Ru	-0.957151164	1.354459756	1.043142187
C	-1.077801728	1.038212576	-0.776057654
H	-0.866803166	1.689183280	-1.632131086
C	-1.665644884	-0.281019377	-1.138644646
C	-1.044004465	-1.534652106	-0.912289980
C	-1.720118191	-2.725041819	-1.219255796
C	-2.997003481	-2.694601493	-1.780550477
C	-3.610544515	-1.471105985	-2.048606906
C	-2.945041568	-0.291989341	-1.725940429
H	-1.257894160	-3.685739154	-1.027756395
H	-3.502324060	-3.629662250	-2.006742340
H	-4.602165065	-1.435129145	-2.491588828
H	-3.424089453	0.668088047	-1.904929091
O	0.242421515	-1.496221134	-0.425632161
C	0.887533797	-2.719580351	-0.031212892
C	0.350440812	-3.195856068	1.319084095
C	2.383362949	-2.432095873	0.012828493
H	0.714625543	-3.481759194	-0.804004296
H	-0.725548017	-3.384112397	1.276502811
H	0.851364654	-4.120683943	1.626212093
H	0.531980567	-2.423214381	2.072376995
H	2.721354928	-2.001471040	-0.933295962
H	2.621916723	-1.734428110	0.817542933
H	2.935360462	-3.360618539	0.191608980
C	-1.187143570	3.271723373	0.956217111
N	-0.752542905	4.284947149	0.127248633
C	-1.087791731	5.626381432	0.636175259
C	-2.158524684	5.323135110	1.695281400
N	-1.936023343	3.897401138	1.931314050
H	-0.202851659	6.105875527	1.077208741
H	-1.454031496	6.271461714	-0.169165959
H	-2.029235274	5.909051175	2.611884455
H	-3.174371634	5.507067444	1.315868995
C	0.323104738	4.163069758	-0.804525146
C	0.017605197	4.163269002	-2.177766881
C	-1.419978886	4.244062376	-2.633591474
C	1.065049494	4.084295084	-3.097459618
C	2.400780674	4.029915179	-2.683230940
C	3.515693744	3.970239194	-3.702222550
C	2.673811908	4.050351261	-1.312819439
C	1.653896600	4.115057866	-0.355412237
C	1.983665420	4.113114562	1.118577342
H	-1.848861359	5.230688882	-2.418344819
H	-2.037332046	3.507467965	-2.111446002
H	-1.501608476	4.072145540	-3.710456370
H	0.835160877	4.064376220	-4.160949001
H	3.669102352	4.946678524	-4.178502214
H	3.287556762	3.256390676	-4.501359221
H	4.465293491	3.675528131	-3.245171457
H	3.706734481	4.003768429	-0.974220846
H	1.785305161	5.088283684	1.580669385
H	1.376551504	3.372053404	1.645812705
H	3.038873648	3.876703429	1.283100009
C	-2.595925657	3.155355775	2.956980069
C	-1.946731179	3.022454605	4.197991380
C	-0.590183458	3.649741320	4.415787243
C	-2.578586690	2.292528164	5.208845895
C	-3.827383784	1.698545723	5.007988483
C	-4.478130300	0.875609421	6.095488990
C	-4.453842906	1.857468669	3.766350022
C	-3.859918830	2.577858282	2.727890871
C	-4.527933361	2.710337881	1.381964682
H	-0.229845601	3.457529536	5.430937371
H	0.136033302	3.242434704	3.705669767
H	-0.617812420	4.735504218	4.266561318
H	-2.082744747	2.182189238	6.171080157
H	-4.284941868	-0.193797092	5.943932954
H	-4.094580864	1.141255601	7.085387790
H	-5.564884670	1.009969578	6.104210598
H	-5.427185270	1.400566236	3.599789367
H	-4.702471504	3.760034105	1.118364581
H	-3.883612750	2.276831912	0.608767710
H	-5.491456583	2.193783143	1.369225460
S	0.800795338	0.498364757	2.721057628
C	2.143656194	0.664964038	1.665918850
N	2.079352728	0.943949196	0.350934663
C	3.342662730	1.005257883	-0.203174512
C	4.230462849	0.750842947	0.791665690
N	3.478059408	0.522351295	1.948938222
H	1.132106232	1.122438551	-0.053077444
H	3.492835804	1.233740249	-1.244786821
H	5.308658993	0.731852318	0.820901699
C	4.020478630	0.393699102	3.288212287
H	4.972411363	-0.136340527	3.239592048
H	3.298809100	-0.150624413	3.898430959
S	6.935988412	1.810319350	3.159770790
C	5.478264937	2.354268090	3.799035525
N	4.269353750	1.691335231	3.883986339
C	3.289066502	2.518946011	4.435705908
C	3.884645150	3.695879985	4.733501106
N	5.217444276	3.577443458	4.352874534
H	2.267922766	2.185366016	4.532592639
H	3.487741253	4.592703526	5.180422976
H	5.935992281	4.280003373	4.432584619

3a’

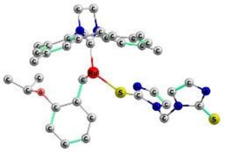

94
RuC38N6H46S2O

Ru	-0.231794822	1.333725407	1.721758418
C	-0.725814804	0.572050917	0.115285589
H	-1.261010378	0.979034318	-0.748022703
C	-0.420832446	-0.877407099	0.044044356
C	-1.458084127	-1.839208412	0.028853018
C	-1.178937993	-3.206078884	0.014011728
C	0.143632885	-3.656168280	-0.002695632
C	1.184968248	-2.728633790	0.015687043
C	0.901409208	-1.362845898	0.046028646
H	-2.003243473	-3.913265029	0.044451816
H	0.353318495	-4.722214546	-0.012170219
H	2.218566947	-3.064803299	0.007201997
H	1.712951191	-0.642745687	0.053742908
O	-2.759964054	-1.392968596	0.125774545
C	-3.652648204	-1.709555716	-0.959117063
C	-3.437649934	-0.717620911	-2.100579370
C	-5.060701200	-1.661794075	-0.381666137
H	-3.439502162	-2.727001234	-1.316920429
H	-2.397526302	-0.748013472	-2.439616181
H	-4.088815287	-0.948820568	-2.950648248
H	-3.654848640	0.298335541	-1.755853603
H	-5.157658735	-2.373679941	0.443151775
H	-5.274750697	-0.660266402	0.004094670
H	-5.801795397	-1.906991112	-1.149194580
C	-1.284954038	2.914561584	1.393373050
N	-1.334331926	3.819361637	0.350472506
C	-2.186052485	4.987323698	0.631659354
C	-2.959986799	4.548206754	1.886473091
N	-2.196710419	3.375410849	2.315127945
H	-1.569196854	5.876803432	0.820795857
H	-2.841839840	5.209594213	-0.217044765
H	-2.979397134	5.320350835	2.664372572
H	-4.000783346	4.278355335	1.657901573
C	-0.369636066	3.918924668	-0.695247277
C	-0.756860003	3.586453685	-2.006683236
C	-2.165723740	3.130720379	-2.305171404
C	0.181053471	3.706897027	-3.035263357
C	1.477108692	4.173549087	-2.796627647
C	2.464790135	4.316395147	-3.932518743
C	1.822785181	4.531800207	-1.489481568
C	0.918523395	4.419518225	-0.427599302
C	1.325439401	4.809069671	0.972587047
H	-2.826110416	3.991236925	-2.478598704
H	-2.584930056	2.564023164	-1.470763579
H	-2.198329673	2.508741422	-3.204592207
H	-0.107516334	3.429649160	-4.047436794
H	2.255945776	5.213738758	-4.528703231
H	2.417092510	3.459958664	-4.613813604
H	3.492624880	4.401040780	-3.565592472
H	2.823417868	4.909085117	-1.287107863
H	0.661012084	5.576408854	1.385959687
H	1.268408012	3.945012081	1.644220751
H	2.346583626	5.200186281	0.989102223
C	-2.547660790	2.624025637	3.476715521
C	-1.904607640	2.935788056	4.686923725
C	-0.831839653	3.999889829	4.723198738
C	-2.262773696	2.225548464	5.836190653
C	-3.236015414	1.223140556	5.799288636
C	-3.591749801	0.442127908	7.043133178
C	-3.853399391	0.932245818	4.577456083
C	-3.522002254	1.611178488	3.402979876
C	-4.137193554	1.223403963	2.081357114
H	-0.420764271	4.108223490	5.731084319
H	-0.011322139	3.747222065	4.042193011
H	-1.218866605	4.976447479	4.408759604
H	-1.770164295	2.458542308	6.778137416
H	-3.036587920	-0.503208602	7.082882732
H	-3.351706791	1.001712978	7.952553716
H	-4.657689557	0.192448753	7.068748567
H	-4.604874775	0.146201827	4.535700111
H	-4.455845131	2.096767429	1.502274142
H	-3.401065400	0.681768394	1.475842300
H	-5.006785046	0.576298435	2.229662229
S	2.009649173	1.053522948	3.023888891
C	3.034883110	1.372806118	1.686168883
N	2.629083253	1.787720262	0.469605485
C	3.710566087	1.951661200	-0.379027589
C	4.824513037	1.626538341	0.326007474
N	4.395526249	1.253678635	1.603778416
H	1.589816937	1.878776854	0.327783326
H	3.584067110	2.291733326	-1.393283457
H	5.875295958	1.640442536	0.080833623
C	5.251497404	0.859231274	2.707039126
H	6.123525322	0.338278333	2.309558474
H	4.673999830	0.208030042	3.364875436
S	8.101226645	2.261136231	2.073125732
C	6.918510438	2.645055597	3.206591285
N	5.726562327	1.997827513	3.463877561
C	5.007237010	2.659578720	4.461111863
C	5.758222687	3.712291889	4.856974188
N	6.918766734	3.687777936	4.091198854
H	4.028626340	2.318217279	4.760836501
H	5.574779320	4.466324535	5.604991476
H	7.687404265	4.338504374	4.134451766

3a

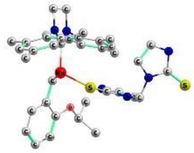

94
RuC38N6H46S2O

Ru	-1.501940470	1.468736378	1.443740306
C	-1.297619217	0.724340526	-0.251230757
H	-0.765910784	1.085200422	-1.136076035
C	-1.608895218	-0.719800163	-0.282846695
C	-0.665163892	-1.662963640	-0.773916966
C	-0.960603377	-3.027955155	-0.791079554
C	-2.185831366	-3.484372682	-0.292065680
C	-3.117232619	-2.585277961	0.217946094
C	-2.827532430	-1.219506568	0.210502456
H	-0.248851737	-3.748728260	-1.175560465
H	-2.398891185	-4.550141166	-0.305486151
H	-4.069366693	-2.938638452	0.603663247
H	-3.557916801	-0.496262558	0.564870392
O	0.523382967	-1.122803805	-1.197847468
C	1.470367709	-1.928777225	-1.916142731
C	2.347185779	-2.735592213	-0.954625617
C	2.281219452	-0.949812286	-2.757879357
H	0.924071451	-2.608996393	-2.584904174
H	1.746626328	-3.371759178	-0.300456334
H	3.040365837	-3.372493368	-1.514855161
H	2.931276065	-2.064334667	-0.318877960
H	1.627565870	-0.394819292	-3.436090113
H	2.782556258	-0.221430603	-2.113744384
H	3.038627181	-1.479856421	-3.344355179
C	-1.392072971	3.314617243	0.916748421
N	-0.642029457	4.024240260	-0.000844024
C	-0.822193302	5.481106402	0.112624805
C	-2.108138908	5.591202747	0.945090766
N	-2.178192213	4.260241735	1.546031058
H	0.035781609	5.940995183	0.624107617
H	-0.912258941	5.944189287	-0.875541095
H	-2.056427811	6.375150503	1.708642064
H	-2.987420759	5.788972500	0.314312664
C	0.533029808	3.539222567	-0.651675083
C	0.462060769	3.251136796	-2.026395251
C	-0.853106887	3.378195509	-2.757297265
C	1.621844833	2.839486796	-2.686926516
C	2.844475900	2.723697593	-2.017290790
C	4.095331964	2.319984708	-2.764556325
C	2.884586224	3.017449044	-0.650944606
C	1.745433722	3.428694529	0.051610022
C	1.819449022	3.730177816	1.529619159
H	-1.164310642	4.427132218	-2.837408363
H	-1.646942247	2.849182114	-2.221958721
H	-0.780702966	2.972603893	-3.770362694
H	1.570172805	2.601481850	-3.747555218
H	4.560041902	3.186180323	-3.252873529
H	3.874712737	1.586804599	-3.547177229
H	4.843871354	1.885091880	-2.094231010
H	3.826035261	2.924001897	-0.113197041
H	1.683520604	4.799688732	1.732081108
H	1.033004589	3.195335643	2.069596378
H	2.788003151	3.433901491	1.942600121
C	-3.120597802	3.910059987	2.559223298
C	-2.711745955	4.009966646	3.902447395
C	-1.306638400	4.452646608	4.235686246
C	-3.627188816	3.686055294	4.906591660
C	-4.926749560	3.269757221	4.602558131
C	-5.891860257	2.895110701	5.703446556
C	-5.305216284	3.182335082	3.258276723
C	-4.421839252	3.494438979	2.221256106
C	-4.830141051	3.366513573	0.774522072
H	-1.153749789	4.483997925	5.318783093
H	-0.576262409	3.763061697	3.800750660
H	-1.091427903	5.449470394	3.833379558
H	-3.320309610	3.761314655	5.947890037
H	-5.809508727	1.829224898	5.949522489
H	-5.691241848	3.457381481	6.621212777
H	-6.929498416	3.082513254	5.409555560
H	-6.313955255	2.858382025	3.010262877
H	-4.791728802	4.331984275	0.256266107
H	-4.145090539	2.685292910	0.257213670
H	-5.848865945	2.978758030	0.686722567
S	-0.233377699	0.794230985	3.577547333
C	1.275468783	0.484702224	2.817938537
N	1.507827312	0.469618210	1.490595661
C	2.844660342	0.240919944	1.226663523
C	3.469288677	0.087283158	2.421294233
N	2.485198158	0.221658479	3.406768221
H	0.722929356	0.712373704	0.851643223
H	3.226003144	0.231133739	0.220669596
H	4.502128337	-0.069104969	2.690778557
C	2.746610819	0.356291939	4.827993372
H	3.565679589	-0.307586119	5.106698367
H	1.835174188	0.103628859	5.370271372
S	5.862458677	1.242167753	4.968036521
C	4.455030765	2.142973499	5.165219306
N	3.143048815	1.711086228	5.160437606
C	2.266867421	2.789119914	5.309883632
C	3.025696991	3.900464800	5.443937508
N	4.352680119	3.492562875	5.361132113
H	1.198065969	2.647907131	5.279018183
H	2.744947295	4.930767971	5.590043720
H	5.171812432	4.077335024	5.418878575

4a

4a$_{trans}$

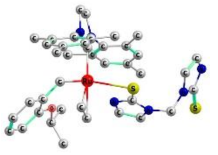

100

RuC40N6H50S2O

Ru	-1.421849155	1.024392077	2.046799771
C	-2.113660605	0.599044266	0.354953953
H	-2.047004123	1.319203829	-0.472669015
C	-2.669890703	-0.680198083	-0.151373749
C	-1.964371367	-1.489319629	-1.074613194
C	-2.525360326	-2.674063908	-1.561979653
C	-3.807837731	-3.060649848	-1.159374048
C	-4.527057840	-2.269921151	-0.268390068
C	-3.951315933	-1.098980639	0.230794743
H	-1.979763604	-3.303998112	-2.254865348
H	-4.231796601	-3.982373198	-1.548490024
H	-5.525210649	-2.562678361	0.045511734
H	-4.498821002	-0.471156060	0.928156353
O	-0.722870167	-1.016514267	-1.438358701
C	0.154416571	-1.831190776	-2.232089981
C	0.787125056	-2.924497438	-1.369612674
C	1.181143472	-0.876753364	-2.832640310
H	-0.412576628	-2.286175291	-3.056112917
H	0.022464422	-3.566417310	-0.924947091
H	1.458292228	-3.549178431	-1.968849252
H	1.361850676	-2.475364113	-0.554132747
H	0.699214671	-0.168541087	-3.511997517
H	1.672658201	-0.297523283	-2.045661644
H	1.942549391	-1.432708087	-3.388981508
C	-1.505930814	2.996896071	1.398415709
N	-0.711826151	3.715758426	0.560139858
C	-1.282371314	5.026218471	0.180678955
C	-2.520246490	5.126998840	1.089293243
N	-2.592913738	3.769614530	1.655698902
H	-0.558906646	5.828371318	0.353675137
H	-1.543162645	5.032598749	-0.884984900
H	-2.399788219	5.870226723	1.888882231
H	-3.433695612	5.371607837	0.538671437
C	0.648394425	3.398754540	0.244330660
C	0.977885120	2.793510771	-0.975807798
C	-0.068204061	2.424162229	-1.998615967
C	2.326766601	2.502806872	-1.224941794
C	3.331862805	2.796905938	-0.302166171
C	4.777494626	2.457290202	-0.576175557
C	2.972611985	3.438917308	0.889973410
C	1.646067197	3.764785611	1.170903867
C	1.274775856	4.501648992	2.434199904
H	-0.956612623	3.055546243	-1.926926655
H	-0.394078111	1.389866189	-1.853790562
H	0.335704837	2.511791149	-3.012388724
H	2.593284442	2.031540649	-2.169093720
H	5.354266020	3.357061820	-0.825110187
H	4.873647306	1.762600140	-1.416197862
H	5.252330519	2.001211207	0.299546255
H	3.745006936	3.690719637	1.613538983
H	0.772785847	5.450745973	2.210289905
H	0.596804228	3.901719722	3.045523196
H	2.163778841	4.720521463	3.029277119
C	-3.667455060	3.365648287	2.509222685
C	-3.520647217	3.472444272	3.901718511
C	-2.234442247	3.985292936	4.505944669
C	-4.578650912	3.055262729	4.714791784
C	-5.762628066	2.545175135	4.175104144
C	-6.876443270	2.052360502	5.069745764
C	-5.883915358	2.472283051	2.783567909
C	-4.852365935	2.875551528	1.933743632
C	-4.979566394	2.732212585	0.437969571
H	-2.341064988	4.137312874	5.583728227
H	-1.415360272	3.276576322	4.341718935
H	-1.923271104	4.935140109	4.057153934
H	-4.472446682	3.125221614	5.795494002
H	-6.857335416	2.544812873	6.046961867
H	-7.859689096	2.225846626	4.620109903
H	-6.785892610	0.973239166	5.245443188
H	-6.801215056	2.079496449	2.349235683
H	-4.847025587	3.687573798	-0.082912269
H	-4.211648152	2.047065994	0.065833153
H	-5.960442533	2.334962292	0.162937795
S	0.694684895	1.240407275	3.929710905
C	1.788115070	0.585335062	2.789016141
N	1.503052810	0.244593743	1.514540167
C	2.619434703	-0.264219931	0.878244739
C	3.636438828	-0.252698628	1.773945302
N	3.114205235	0.265800767	2.962189411
H	0.508730094	0.366376450	1.183605561
H	2.600413231	-0.566009075	-0.153704702
H	4.679771094	-0.511255412	1.694527759
C	3.865679219	0.517414522	4.171077617
H	4.686035513	-0.198906158	4.231282940
H	3.193037628	0.411881992	5.023657532
S	6.777700260	1.163023761	2.893515658
C	5.650075309	2.175837515	3.624229229
N	4.436370522	1.852072923	4.193890212
C	3.795796572	2.992636618	4.676222785
C	4.616092069	4.041351236	4.435896805
N	5.739453866	3.530171396	3.794339739
H	2.805060253	2.936063937	5.098152666
H	4.499756364	5.090454225	4.653140910
H	6.548410551	4.045282851	3.483728508
C	-1.170811447	-1.196780105	2.534185130
C	-2.308970076	-0.668489579	3.140249995
H	-0.253207489	-1.322160444	3.102062861
H	-1.257748997	-1.811760055	1.643349064
H	-2.270408937	-0.358652264	4.186384083
H	-3.301100474	-0.896924680	2.766184078

5a$^{‡}_{trans}$ (275.02*i* cm^{-1})

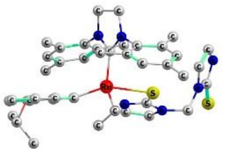

100

RuC40N6H50S2O

Ru	-0.930738987	0.803294447	1.466130486
C	-1.045795888	-0.291611643	-0.105137014
H	-0.020830591	-0.498024690	-0.449183914
C	-2.025382748	-0.787889214	-1.084491958
C	-1.607483147	-1.163138423	-2.383971599
C	-2.521408226	-1.586900238	-3.350927621
C	-3.876764585	-1.680434368	-3.042095056
C	-4.316457241	-1.327746463	-1.762592199
C	-3.405912271	-0.877630704	-0.813321080
H	-2.157881560	-1.832858133	-4.344767446
H	-4.583751375	-2.014304817	-3.796681979
H	-5.371315475	-1.395530678	-1.509744420
H	-3.752921438	-0.592891659	0.172498844
O	-0.272461540	-1.031798969	-2.720090965
C	0.450064546	-2.247583827	-3.004783217
C	0.522924302	-3.145326612	-1.770615854
C	1.823596925	-1.799641955	-3.484085315
H	-0.065241104	-2.788610903	-3.813338941
H	-0.479355974	-3.399885741	-1.414540474
H	1.052000513	-4.074796242	-2.006303369
H	1.053266626	-2.635328199	-0.959941601
H	1.732013023	-1.153488091	-4.362404216
H	2.322098446	-1.233092391	-2.691532105
H	2.444066354	-2.662944004	-3.744831018
C	-1.282189333	2.737361832	1.147542550
N	-0.630687182	3.488903960	0.198469705
C	-1.310560778	4.753627159	-0.105789707
C	-2.212720889	4.922171617	1.129994814
N	-2.245318262	3.555320400	1.672621927
H	-0.591390747	5.569808728	-0.226183191
H	-1.898792406	4.673098582	-1.030721398
H	-1.782304781	5.623158155	1.858332547
H	-3.220589959	5.266958944	0.878003538
C	0.478340041	2.985776900	-0.546026898
C	0.284250495	2.400284511	-1.807486296
C	-1.093493778	2.306103138	-2.414376641
C	1.397705287	1.880332330	-2.475973836
C	2.678991481	1.934006785	-1.922033846
C	3.877269350	1.409727487	-2.679863557
C	2.839879350	2.518053621	-0.659200674
C	1.757163427	3.054131806	0.041985300
C	1.943869572	3.694588589	1.397507508
H	-1.832740388	2.049901950	-1.651414676
H	-1.123317335	1.533697888	-3.185755671
H	-1.391905130	3.256565243	-2.875992056
H	1.248722739	1.394326762	-3.436274425
H	4.383980231	2.219629668	-3.220302571
H	3.588606612	0.656132589	-3.417947847
H	4.616041824	0.961157308	-2.006647300
H	3.832397798	2.565638909	-0.214216482
H	1.699731854	4.763523162	1.363323632
H	1.286032457	3.238451337	2.142513693
H	2.977549708	3.593730352	1.740814535
C	-3.090143343	3.187222797	2.762770603
C	-2.757927747	3.564699341	4.072515389
C	-1.532834642	4.400257612	4.360089948
C	-3.597898375	3.155503391	5.115148165
C	-4.742778788	2.391519074	4.880597859
C	-5.606549719	1.908677183	6.022753752
C	-5.060462213	2.054630106	3.559426803
C	-4.253482982	2.444818103	2.490921658
C	-4.597815873	2.079243835	1.069540392
H	-1.215358022	4.275410484	5.399963461
H	-0.701044228	4.114682071	3.711243599
H	-1.734225414	5.468771512	4.206575389
H	-3.341919626	3.431835462	6.136302369
H	-5.476590144	2.526265818	6.916783155
H	-6.668455595	1.919007768	5.754434883
H	-5.352159333	0.876667891	6.295842579
H	-5.956445646	1.470156956	3.359081266
H	-4.781869801	2.973935492	0.461993760
H	-3.764358027	1.542938475	0.607192385
H	-5.492873282	1.452014975	1.026676134
S	0.092995648	1.274000602	3.944805399
C	1.692970110	0.848042461	3.561658051
N	2.144488555	0.508058205	2.331440342
C	3.514052790	0.292956353	2.342710856
C	3.932956505	0.481763651	3.618580996
N	2.800666411	0.805685804	4.372600793
H	1.459531416	0.484456654	1.558712338
H	4.058694694	0.030766508	1.450565134
H	4.908987855	0.446662371	4.077187656
C	2.794710664	1.187559170	5.770318207
H	3.564655220	0.624202446	6.299098242
H	1.804176045	0.971744690	6.173768262
S	5.791635767	2.253452141	6.389315581
C	4.350419196	3.095714469	6.175395105
N	3.084082496	2.596404216	5.947566602
C	2.164157325	3.633557790	5.777940807
C	2.844087094	4.792551920	5.931252222
N	4.169113872	4.451002132	6.179782340
H	1.132717550	3.427895600	5.537681914
H	2.508802802	5.815934011	5.887144903
H	4.939405852	5.082288554	6.334300825
C	-1.418389195	-1.473508064	1.689411000
C	-2.216682072	-0.542145536	2.460723067
H	-0.525737146	-1.897288418	2.149203421
H	-1.900117564	-2.151426113	0.995439139
H	-2.017138702	-0.486042295	3.529101547
H	-3.275564679	-0.450766693	2.218162906

6a$_{trans}$

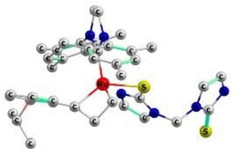

100
RuC40N6H50S2O

Ru1	-0.585609138	1.149699966	1.761451018
C2	-1.347890758	-0.673988949	0.938474663
H3	-0.420883782	-1.029200233	0.474241885
C4	-2.484328059	-0.953324110	0.041453398
C5	-2.283458326	-1.202437032	-1.338352116
C6	-3.344341632	-1.485604177	-2.199121061
C7	-4.648843879	-1.573227075	-1.711010359
C8	-4.873638116	-1.357409524	-0.349598330
C9	-3.814642030	-1.047228128	0.500762148
H10	-3.133003120	-1.636630199	-3.254610232
H11	-5.471047367	-1.802046930	-2.383043259
H12	-5.881417700	-1.423639205	0.054128903
H13	-4.020277318	-0.856550252	1.549294641
O14	-0.997317603	-1.106438169	-1.852734374
C15	-0.408492246	-2.317559824	-2.360276661
C16	-0.056346583	-3.277255353	-1.224118012
C17	0.812928604	-1.880637058	-3.159717462
H18	-1.126826716	-2.811140636	-3.032236513
H19	-0.936727051	-3.480905239	-0.607854074
H20	0.314593594	-4.227086368	-1.625292924
H21	0.717512522	-2.843256137	-0.582458554
H22	0.516880125	-1.233370955	-3.991097278
H23	1.494805295	-1.317783340	-2.513210521
H24	1.345179720	-2.748253090	-3.563140866
C25	-1.445171420	2.716767723	1.095675821
N26	-0.854174220	3.503399762	0.110191232
C27	-1.396807781	4.859950655	0.048457864
C28	-2.751144703	4.674428696	0.747760646
N29	-2.536637212	3.446833219	1.526040813
H30	-0.759590330	5.575305470	0.589217074
H31	-1.493823425	5.201264014	-0.987454915
H32	-3.009896134	5.510895519	1.405159169
H33	-3.563257276	4.546629149	0.020652755
C34	0.277622998	3.075897696	-0.638956130
C35	0.059309548	2.236825829	-1.753147829
C36	-1.346470347	1.875003554	-2.149899675
C37	1.162994851	1.786025464	-2.477912992
C38	2.467182773	2.175743400	-2.147296204
C39	3.638568414	1.685567849	-2.966267225
C40	2.652405076	3.043285008	-1.066684487
C41	1.572760951	3.505416673	-0.296896250
C42	1.798220741	4.441775747	0.866734632
H43	-1.850530517	1.361382500	-1.329732630
H44	-1.357092955	1.211620409	-3.015151725
H45	-1.924736701	2.777936427	-2.380467613
H46	0.999538309	1.123393651	-3.323013262
H47	3.651447899	2.157459712	-3.956347732
H48	3.583942859	0.603335851	-3.128292230
H49	4.594039056	1.910347219	-2.482230218
H50	3.658327067	3.371213498	-0.809044532
H51	1.590980556	5.480797817	0.582049984
H52	1.147638226	4.184857195	1.706882931
H53	2.835904131	4.398339829	1.210755340
C54	-3.593142290	2.914632944	2.325326704
C55	-3.529731072	3.065481107	3.720064420
C56	-2.354211711	3.763156594	4.355513942
C57	-4.548947629	2.514840523	4.499366746
C58	-5.619760171	1.823653076	3.924107577
C59	-6.675720880	1.178254761	4.791387659
C60	-5.668431895	1.712078619	2.532003778
C61	-4.671122616	2.252155230	1.713076301
C62	-4.756611023	2.115087970	0.212384287
H63	-2.501061888	3.885234317	5.432695985
H64	-1.442687548	3.181660628	4.189647689
H65	-2.185239795	4.751542427	3.913692341
H66	-4.498842059	2.614459357	5.581912373
H67	-6.833052101	1.738980223	5.718623097
H68	-7.635918721	1.104195803	4.271090642
H69	-6.378265387	0.160571133	5.073867550
H70	-6.496668425	1.180270110	2.067812008
H71	-5.105469156	3.046137624	-0.252244070
H72	-3.783668898	1.866491546	-0.217075711
H73	-5.449882912	1.322200169	-0.071935240
S74	0.954922792	1.921549542	3.582631280
C75	2.502227954	1.455971025	3.025131319
N76	2.934833497	1.395765166	1.739669611
C77	4.253061857	0.968289718	1.673404668
C78	4.659431185	0.740823840	2.944885215
N79	3.571531074	1.028305597	3.773009401
H80	2.356582982	1.668138320	0.951304742
H81	4.771551094	0.868597789	0.733907884
H82	5.605395630	0.435568883	3.364130284
C83	3.577711624	0.952495106	5.222087050
H84	4.239175792	0.140954937	5.528465016
H85	2.554205639	0.771918706	5.553914244
S86	6.704576273	1.356007044	5.948182230
C87	5.396243906	2.400022848	6.121824390
N88	4.066316194	2.172736435	5.828292289
C89	3.300230649	3.312066377	6.082918346
C90	4.142274012	4.254618284	6.564503034
N91	5.410803983	3.685375645	6.587491105
H92	2.242278799	3.330214271	5.872554020
H93	3.956187404	5.265321064	6.889598486
H94	6.266466567	4.124505444	6.889778789
C95	-1.510441096	-1.123793053	2.416442682
C96	-1.824556734	0.202310963	3.122312976
H97	-0.568704764	-1.552469111	2.789844399
H98	-2.269092448	-1.911528823	2.554022290
H99	-1.500546781	0.279025579	4.167624312
H100	-2.875407404	0.475790958	3.030376505

7a‡ (280.19i cm^{-1}) *from* **6a**$_{trans}$

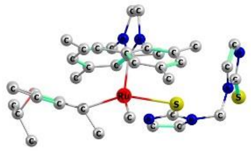

100
RuC40N6H50S2O

Ru	-0.925816824	0.588126568	1.935389642
C	-1.275994137	-0.766525255	0.310686105
H	-0.394865266	-0.567304182	-0.306510774
C	-2.485324881	-0.969673758	-0.522914113
C	-2.397957598	-0.995372347	-1.932681203
C	-3.527065410	-1.162604864	-2.737561944
C	-4.782538090	-1.356191820	-2.163764920
C	-4.891674305	-1.373552541	-0.770578126
C	-3.765381143	-1.177656676	0.024653425
H	-3.400283172	-1.144575246	-3.816946208
H	-5.658395746	-1.489075837	-2.792541959
H	-5.859826341	-1.528364044	-0.300555553
H	-3.875727079	-1.164422511	1.103846628
O	-1.162493233	-0.833371725	-2.545699354
C	-0.606330007	-2.026535910	-3.138748580
C	-0.195837846	-3.039765225	-2.070595190
C	0.570444589	-1.567260100	-3.988265969
H	-1.370531202	-2.481041484	-3.787988511
H	-1.046776192	-3.306084974	-1.438066492
H	0.187609821	-3.952644541	-2.540194358
H	0.585095161	-2.620539977	-1.428201080
H	0.246278526	-0.826135376	-4.725159576
H	1.334829301	-1.110337395	-3.351961245
H	1.017861439	-2.415212739	-4.516475254
C	-1.249746973	2.546347880	1.502781822
N	-0.554754714	3.305823467	0.603380580
C	-0.890787921	4.735030578	0.646182350
C	-2.188235919	4.724603440	1.472777204
N	-2.150342735	3.388952531	2.088807779
H	-0.091278500	5.306648074	1.138616326
H	-1.028024287	5.138508594	-0.362259909
H	-2.215284883	5.508354401	2.236220806
H	-3.081467360	4.832019985	0.841605914
C	0.502207700	2.784015031	-0.201045718
C	0.167400569	2.222730705	-1.448253764
C	-1.267799073	2.207959381	-1.909254309
C	1.190872450	1.690616320	-2.231604739
C	2.525217733	1.711514705	-1.807152942
C	3.605345202	1.095912494	-2.667065417
C	2.828398980	2.293632816	-0.573393950
C	1.832257548	2.840761550	0.246288573
C	2.185825190	3.490864300	1.562001535
H	-1.897294324	1.694115750	-1.178014274
H	-1.367798910	1.689103972	-2.863300961
H	-1.657200755	3.228126854	-2.016207681
H	0.937843757	1.247015383	-3.189797653
H	3.491313098	1.385497980	-3.717318575
H	3.560787309	-0.000224372	-2.632995385
H	4.604928963	1.397779055	-2.339970486
H	3.862002089	2.324625437	-0.234020620
H	2.276081592	4.579685516	1.453397061
H	1.422015930	3.295395674	2.317410683
H	3.144828096	3.121553036	1.937857260
C	-3.141881709	2.974231974	3.027818172
C	-2.952268193	3.262145058	4.386977397
C	-1.743458584	4.040145111	4.850565404
C	-3.913802753	2.818837414	5.301741615
C	-5.043599020	2.109325856	4.889783222
C	-6.046424948	1.588206914	5.893158975
C	-5.217732315	1.865170966	3.522253922
C	-4.284926789	2.290048126	2.575602222
C	-4.476632761	2.008748815	1.106922752
H	-1.557979338	3.878350658	5.916729265
H	-0.851808353	3.740607252	4.293858264
H	-1.884820144	5.118956935	4.700501281
H	-3.769635600	3.025090556	6.360461335
H	-5.988051733	2.130620828	6.841710854
H	-7.071416839	1.672221020	5.516288968
H	-5.867875296	0.527277413	6.109572753
H	-6.101132511	1.325880314	3.185721442
H	-4.492967848	2.934078417	0.518730805
H	-3.654689421	1.395764303	0.727321286
H	-5.412417896	1.475528109	0.922245147
S	0.501860329	1.231105564	4.159416668
C	2.036633118	0.821526104	3.561902856
N	2.291076187	0.238525721	2.366014804
C	3.653682964	0.121830522	2.141756303
C	4.276494004	0.630016132	3.232921287
N	3.273628568	1.045212045	4.115729043
H	1.494283434	-0.001285506	1.766475656
H	4.051395759	-0.296515660	1.232033200
H	5.317747426	0.774598402	3.473014721
C	3.484995805	1.750859714	5.364356215
H	4.426575090	1.416866505	5.802121344
H	2.646030835	1.530556443	6.025712852
S	6.304957653	3.232936157	4.791086983
C	4.743521397	3.851347830	4.891798714
N	3.567692211	3.185770896	5.175125065
C	2.481447038	4.063524332	5.143308494
C	2.973466266	5.291117355	4.860595164
N	4.348877384	5.147949026	4.712541956
H	1.474513372	3.708288714	5.293656646
H	2.474898686	6.240068865	4.748710635
H	5.014727291	5.873805469	4.498480318
C	-0.990405728	-1.628083997	1.460624960
C	-1.957747619	-0.453058206	3.080545083
H	0.008712646	-2.045506348	1.580705609
H	-1.750933040	-2.350633927	1.747100261
H	-1.642588704	-0.670978213	4.109448813
H	-2.958930128	-0.830314297	2.849705528

8a

from **6a***trans*

100

RuC40N6H50S2O

Ru	-0.522734051	1.063447039	1.630030848
C	-0.572606544	-0.381258473	-0.015512207
H	-0.395461324	0.139343102	-0.951280905
C	-1.654601329	-1.380270500	-0.119726305
C	-2.475950673	-1.465872684	-1.267363685
C	-3.536707682	-2.370153179	-1.351210973
C	-3.797426705	-3.260376270	-0.310532810
C	-2.973072378	-3.233078386	0.816362006
C	-1.931544232	-2.312797003	0.903753361
H	-4.147996110	-2.364070868	-2.249892172
H	-4.624514715	-3.961067673	-0.380610627
H	-3.145100048	-3.926536208	1.635980912
H	-1.313871003	-2.298554737	1.796274191
O	-2.241006361	-0.629791981	-2.351567708
C	-1.670725074	-1.281876010	-3.509454968
C	-0.180518533	-1.550094433	-3.302674725
C	-1.950541691	-0.381385999	-4.704853534
H	-2.190577304	-2.240523863	-3.652431749
H	-0.020836136	-2.154831494	-2.406091030
H	0.234644343	-2.086353133	-4.163874523
H	0.362829564	-0.608001149	-3.178464921
H	-3.022631027	-0.182940579	-4.791738524
H	-1.432175404	0.575000571	-4.593006124
H	-1.602957505	-0.855307836	-5.628750133
C	-1.297382974	2.861084353	0.994454007
N	-0.663307108	3.678590040	0.101254459
C	-1.291282344	5.006148526	-0.019830337
C	-2.647096189	4.777453747	0.663094193
N	-2.408117547	3.529794090	1.414479553
H	-0.688471255	5.762207602	0.501245227
H	-1.380326650	5.304325513	-1.068478776
H	-2.929878250	5.591305854	1.336574579
H	-3.462473651	4.635041958	-0.059850920
C	0.335308935	3.220342101	-0.815745647
C	-0.083969149	2.695184088	-2.054427464
C	-1.545911622	2.589289580	-2.413307471
C	0.889789073	2.232665237	-2.943267699
C	2.252335151	2.280305669	-2.630678011
C	3.274801746	1.723440438	-3.593144168
C	2.637273206	2.839300401	-1.409050286
C	1.698372094	3.326086478	-0.492194495
C	2.140700323	3.950163243	0.809418647
H	-1.955994451	1.638529256	-2.060052305
H	-1.680265651	2.619671193	-3.498461045
H	-2.145481839	3.391487501	-1.978235172
H	0.577490939	1.828538044	-3.903726172
H	3.036157393	1.989127407	-4.628627781
H	3.300778403	0.628255549	-3.538989179
H	4.281613221	2.089633421	-3.371599935
H	3.695316223	2.906424280	-1.164113842
H	1.662429691	4.923362561	0.966613197
H	1.870046812	3.321754165	1.663915383
H	3.225184017	4.096760380	0.820233127
C	-3.460408121	2.973456217	2.208677224
C	-3.584320500	3.375670089	3.546881233
C	-2.577982838	4.315276869	4.164996509
C	-4.629114507	2.842012446	4.308522546
C	-5.531612185	1.921641601	3.768677779
C	-6.625604942	1.316162911	4.617450384
C	-5.381909154	1.542766143	2.430022787
C	-4.357425679	2.054789346	1.629865946
C	-4.181994939	1.586340350	0.206765658
H	-2.869754840	4.588061358	5.183318982
H	-1.591807539	3.841029393	4.207904285
H	-2.466313745	5.239074216	3.587123266
H	-4.730732938	3.143146343	5.349029939
H	-6.841981224	1.931715005	5.495894426
H	-7.554097787	1.196349679	4.049173331
H	-6.335850974	0.320420675	4.976205715
H	-6.073888071	0.822418938	1.998854878
H	-4.109315491	2.423595708	-0.496066671
H	-3.256326256	1.009342802	0.111428247
H	-5.015423600	0.951816461	-0.105597921
S	0.586514035	2.145511524	3.702307393
C	1.496076586	0.852803971	4.355516274
N	1.584449534	-0.390340616	3.838104455
C	2.437013655	-1.180162132	4.592253513
C	2.886714951	-0.416104545	5.618254689
N	2.287050203	0.838702460	5.475626576
H	1.080153312	-0.593499344	2.961526109
H	2.643010242	-2.207151838	4.337742791
H	3.581890686	-0.609170213	6.420452802
C	2.576027399	2.007499016	6.284061769
H	2.759371552	1.694171397	7.312700085
H	1.718023120	2.678685099	6.225581251
S	5.498000722	1.454782754	7.571145527
C	5.028343614	2.465264383	6.310736190
N	3.759361290	2.702781137	5.819973856
C	3.804401564	3.578777124	4.732675443
C	5.100477690	3.921568935	4.555106468
N	5.831525713	3.243909190	5.524392778
H	2.908794591	3.839946022	4.190882869
H	5.562628452	4.578330291	3.836243805
H	6.829592860	3.273554249	5.662041362
C	0.590889168	-0.599487434	0.840143803
C	-1.835303791	0.349594053	2.663876552
H	1.548526454	-0.222588023	0.464565710
H	0.708314879	-1.586871872	1.296689486
H	-1.917062900	0.610391048	3.727566421
H	-2.597674889	-0.338612136	2.282284871

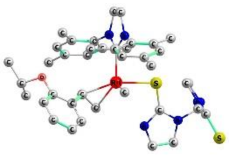

9a

100

RuC40N6H50S2O

Ru	-1.623808650	2.581763650	2.120952093
C	-0.904834389	-2.762495836	1.997091912
H	-1.951079577	-2.478959854	1.917869476
C	-0.163212630	-2.827922001	0.726779164
C	-0.555336405	-2.009262430	-0.360419643
C	0.193912132	-1.998036087	-1.544261851
C	1.324965672	-2.807101896	-1.664884420
C	1.711279850	-3.638225180	-0.612708355
C	0.964602640	-3.644189121	0.563663711
H	-0.099101587	-1.356658724	-2.367484300
H	1.896196933	-2.789452832	-2.589192216
H	2.580022687	-4.282211252	-0.711021267
H	1.251328555	-4.299184215	1.380542529
O	-1.629227174	-1.195418783	-0.139157886
C	-2.415619685	-0.702793232	-1.254035736
C	-3.425240523	0.254315982	-0.643350575
C	-3.073166000	-1.863866120	-1.998762125
H	-1.756729273	-0.141012619	-1.929286760
H	-2.924179757	1.032795844	-0.057769702
H	-4.019789019	0.724277422	-1.433768697
H	-4.102951284	-0.286322671	0.025646898
H	-2.336833998	-2.555559490	-2.417020807
H	-3.719835919	-2.421885187	-1.313823353
H	-3.687784723	-1.479640616	-2.819107027
C	-1.643657039	4.090318233	0.877897054
N	-0.493455445	4.422878044	0.180117321
C	-0.671046566	5.527884837	-0.765239806
C	-1.981544318	6.149172494	-0.264050842
N	-2.559748478	5.054478729	0.530063226
H	0.174458358	6.223226085	-0.727015441
H	-0.763404573	5.160513245	-1.798345066
H	-1.802051500	7.033969208	0.363997604
H	-2.654647595	6.443816297	-1.075575414
C	0.708611332	3.656110481	0.234852477
C	0.954255393	2.643290742	-0.711139704
C	-0.098379961	2.284235247	-1.731354415
C	2.176077558	1.966087077	-0.662113043
C	3.140227878	2.253575351	0.310607426
C	4.464345624	1.527400583	0.337318689
C	2.854712411	3.241021799	1.259521540
C	1.656083147	3.960135969	1.232649069
C	1.362196291	5.037619325	2.248433568
H	-0.193822876	3.051948203	-2.509326085
H	-1.073291017	2.184323120	-1.243284028
H	0.148697147	1.340784234	-2.227761763
H	2.372076160	1.180060348	-1.389182439
H	5.251810171	2.134266932	-0.128399903
H	4.413267099	0.581478932	-0.210854040
H	4.784855508	1.305607914	1.360588567
H	3.585248944	3.460104727	2.034859509
H	1.157180788	5.997883158	1.761150844
H	0.470869394	4.778158888	2.832058668
H	2.203642924	5.167977151	2.933353889
C	-3.846238074	5.226663486	1.122048361
C	-3.971421138	5.808046009	2.395053225
C	-2.746887203	6.164825621	3.199115740
C	-5.255585690	5.980131166	2.920391656
C	-6.399665629	5.588825000	2.217431714
C	-7.773872388	5.750091852	2.826753147
C	-6.240254547	5.010058498	0.954379587
C	-4.974784757	4.816449376	0.394724167
C	-4.803034517	4.129476736	-0.937804508
H	-3.018873149	6.667916474	4.131705056
H	-2.188807003	5.248222839	3.433176754
H	-2.065767449	6.818996427	2.643058038
H	-5.364514740	6.423478015	3.908392015
H	-7.820540746	6.621251885	3.488387194
H	-8.543661550	5.866800635	2.057040962
H	-8.043318980	4.871346101	3.426814777
H	-7.119686363	4.689410736	0.399214804
H	-4.321323209	4.781686400	-1.676178854
H	-4.164090602	3.247762007	-0.822897350
H	-5.767403994	3.813729629	-1.346120815
S	-0.302230826	1.634584649	4.006110319
C	0.801711233	0.642031761	3.139880563
N	0.683586309	0.205674304	1.865311798
C	1.802398078	-0.514650580	1.481726960
C	2.634012024	-0.554515373	2.548441417
N	2.005461183	0.157680554	3.575699790
H	-0.140275304	0.449026657	1.307456630
H	1.899370355	-0.937111424	0.497594302
H	3.618873798	-0.969987939	2.685286269
C	2.591446407	0.468031905	4.863959147
H	3.259226723	-0.345904839	5.149196148
H	1.787138030	0.583685647	5.590912857
S	5.751662114	0.459953752	4.151911999
C	4.712609692	1.733767321	4.512764821
N	3.371684690	1.690757913	4.834088740
C	2.871959425	2.976364343	5.046712915
C	3.904614230	3.834746056	4.885013244
N	5.016910886	3.065983119	4.558445092
H	1.825561528	3.140768895	5.248574993
H	3.945350301	4.908689230	4.963936214
H	5.951830984	3.397431642	4.378875993
C	-3.404906677	2.296364443	2.332437198
C	-0.370297232	-2.967044693	3.208432877
H	-3.707950951	1.235051163	2.416044095
H	-4.225620860	3.006550214	2.486314789
H	-0.976285536	-2.902644164	4.106728408
H	0.687567388	-3.176644134	3.344990032

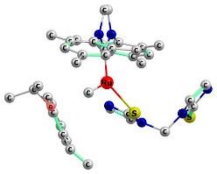

9a’

100

RuC40N6H50S2O

Ru	-0.906905256	0.751720755	1.861786697
C	0.444378016	-1.602104182	-2.257640213
H	-0.458237400	-2.206743726	-2.237005785
C	1.164068909	-1.524021796	-0.976913626
C	0.513293492	-1.924225610	0.217548327
C	1.189531538	-1.864792762	1.444688870
C	2.498482912	-1.390950340	1.501270839
C	3.150644392	-0.983876079	0.338114986
C	2.487298591	-1.063688167	-0.881799713
H	0.695261006	-2.163577198	2.358298817
H	3.000652090	-1.330057757	2.462379194
H	4.170097057	-0.614385680	0.384101203
H	3.005435354	-0.776406893	-1.792175135
O	-0.786000812	-2.323582529	0.082876105
C	-1.490720971	-2.936248824	1.186190872
C	-0.997014633	-4.366751556	1.397293413
C	-2.963902557	-2.867264725	0.810358890
H	-1.336585173	-2.326956719	2.085765374
H	0.075557811	-4.394129489	1.608484444
H	-1.524659895	-4.830874954	2.237501314
H	-1.183850063	-4.963426305	0.498130064
H	-3.277005166	-1.830608724	0.670453419
H	-3.147262645	-3.415143892	-0.120552575
H	-3.575549386	-3.311201434	1.602681455
C	-1.277987659	2.382919360	0.762281835
N	-0.523682658	3.497113821	0.477010770
C	-1.320075896	4.633440837	-0.016082686
C	-2.645164190	3.953063437	-0.394256131
N	-2.548240478	2.678750518	0.326765222
H	-1.462532404	5.383511098	0.773275969
H	-0.827711651	5.117620370	-0.864921206
H	-3.521924925	4.525762995	-0.074985316
H	-2.731142830	3.780132756	-1.477588874
C	0.895938757	3.561216147	0.576352859
C	1.674410957	2.843663299	-0.350710575
C	1.021079932	2.025652160	-1.434021600
C	3.068379176	2.918623798	-0.257297658
C	3.697172364	3.712231893	0.705531021
C	5.203636682	3.817606513	0.774418675
C	2.897088533	4.415876594	1.613757251
C	1.501926154	4.349513022	1.572435599
C	0.663302259	5.077818304	2.594845019
H	0.519445220	1.145768246	-1.022893033
H	1.757023143	1.682611248	-2.164639428
H	0.256987070	2.608362630	-1.960432106
H	3.672627843	2.360687214	-0.969197255
H	5.538416496	4.846447673	0.597233027
H	5.684421694	3.179606963	0.025914489
H	5.578646218	3.520225412	1.760355644
H	3.372850007	5.021858161	2.383053990
H	0.272264921	6.023878273	2.198815896
H	-0.188717746	4.463882863	2.900761013
H	1.252100798	5.314209749	3.485687972
C	-3.606395163	1.722099283	0.265464441
C	-4.620777967	1.781806565	1.238015304
C	-4.521424048	2.774513232	2.368367115
C	-5.669902658	0.864227915	1.168039765
C	-5.730440411	-0.103765672	0.159466899
C	-6.843275145	-1.125827128	0.131576713
C	-4.710352683	-0.136598542	-0.794202651
C	-3.632835192	0.755980951	-0.756058709
C	-2.504746175	0.620975321	-1.749350930
H	-5.370308249	2.680768104	3.051431651
H	-3.597073601	2.593505186	2.927208063
H	-4.489393380	3.808257231	2.005528111
H	-6.450225145	0.895112235	1.925724041
H	-7.745618248	-0.753544343	0.626622590
H	-7.107377302	-1.405936284	-0.893458351
H	-6.540243241	-2.044244255	0.651106391
H	-4.740284363	-0.889225051	-1.579792200
H	-2.062110081	1.584263275	-2.013454844
H	-1.701232224	0.008833664	-1.325942805
H	-2.845478930	0.132844417	-2.667138123
S	0.780062659	1.547631332	3.250055459
C	1.069713116	0.297937582	4.400282455
N	0.284837559	-0.774683544	4.616085855
C	0.822969641	-1.571961400	5.614279667
C	1.977398034	-0.987842662	6.024061218
N	2.118688331	0.184526838	5.273986347
H	-0.630508564	-0.790037862	4.073844965
H	0.349212531	-2.485592285	5.936031546
H	2.734978839	-1.285210702	6.732390236
C	3.216722944	1.128435414	5.358507430
H	3.625775064	1.095698060	6.369550723
H	2.832554292	2.123824935	5.129153160
S	5.694408618	-0.758652708	6.220030024
C	5.362581775	0.011112982	4.761663341
N	4.292050808	0.822558887	4.438314960
C	4.377429516	1.256071899	3.113554081
C	5.516956044	0.734510355	2.603388231
N	6.104582383	-0.019478797	3.613946681
H	3.605622133	1.859745579	2.661670566
H	5.950378345	0.829921954	1.621778340
H	6.963833480	-0.543643692	3.558262260
C	0.780806649	-0.996929579	-3.406267430
C	-2.127341621	0.182288451	3.176592013
H	0.182893383	-1.132030609	-4.302212710
H	1.647146939	-0.348352137	-3.499633799
H	-2.430981028	0.669522370	4.116269261
H	-2.955866881	-0.406520384	2.732567226

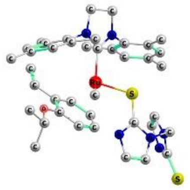

4a$_{cis}$

100

RuC40N6H50S2O

Ru	-1.109013052	1.921788153	2.313634499
C	-1.576879105	0.793296519	0.791835238
H	-1.991206306	1.211537073	-0.132860552
C	-1.668884188	-0.673288772	0.745026504
C	-1.392743242	-1.367729117	-0.467282651
C	-1.399914201	-2.762372602	-0.524862045
C	-1.704275738	-3.507981652	0.618828484
C	-1.981032481	-2.856932866	1.817826510
C	-1.948959428	-1.464329834	1.874626749
H	-1.165143464	-3.283387766	-1.445750245
H	-1.711649096	-4.593172364	0.561870416
H	-2.215036171	-3.428825352	2.711902348
H	-2.153495359	-0.953226309	2.806301152
O	-1.078351384	-0.556019209	-1.538588342
C	-0.898455981	-1.121834832	-2.844541577
C	0.542276515	-1.608732839	-3.015373379
C	-1.264978919	-0.026211205	-3.839712559
H	-1.596127424	-1.960052939	-2.973470143
H	0.824111848	-2.294187846	-2.211692568
H	0.670013971	-2.124127001	-3.973315061
H	1.226029911	-0.752520102	-2.997103997
H	-2.293505154	0.306844910	-3.675783138
H	-0.600932335	0.835221623	-3.721177793
H	-1.173622990	-0.396070705	-4.866348345
C	-1.885328315	3.454550392	1.339474569
N	-1.036697612	4.146601371	0.500496990
C	-1.619961395	5.381686086	-0.038308161
C	-3.114614719	5.165937223	0.248555880
N	-3.094611429	4.077111756	1.241341403
H	-1.221960334	6.263532584	0.483237889
H	-1.401979072	5.495409988	-1.104839418
H	-3.605075173	6.056492166	0.652347364
H	-3.667989658	4.847521256	-0.645235612
C	0.378788007	3.922826892	0.548699805
C	1.002940204	3.256360448	-0.523644127
C	0.208046030	2.789329117	-1.717604061
C	2.362379165	2.954784612	-0.423181050
C	3.107036744	3.274789698	0.716811322
C	4.539982244	2.816005064	0.847411364
C	2.477564177	3.990532553	1.737034622
C	1.124616827	4.341173931	1.669879855
C	0.493266982	5.118917412	2.805354924
H	-0.515759997	3.536403311	-2.055664371
H	-0.357236142	1.885919981	-1.464850298
H	0.871115665	2.554188301	-2.556445743
H	2.844774023	2.418796491	-1.238314928
H	5.043817291	3.299924650	1.688772234
H	5.115452350	3.022569902	-0.061759862
H	4.582272734	1.732458759	1.014885279
H	3.048310222	4.271514901	2.619729185
H	-0.202776896	5.880317467	2.440341579
H	-0.075154320	4.459794307	3.469494036
H	1.262110671	5.622412870	3.398965730
C	-4.280138925	3.765061061	1.977177843
C	-4.597515223	4.556656335	3.097497817
C	-3.730304891	5.724801468	3.507649383
C	-5.752661378	4.258227163	3.824208745
C	-6.598782463	3.208925309	3.454928372
C	-7.815936027	2.869739595	4.283578257
C	-6.274389165	2.462701610	2.318730649
C	-5.125582879	2.721328020	1.564146810
C	-4.792363431	1.854915718	0.377525751
H	-3.792992951	5.897397109	4.586058159
H	-2.681993925	5.561557543	3.248298143
H	-4.054734438	6.649038493	3.011583979
H	-5.992717251	4.854673714	4.702005792
H	-8.233987462	3.758276306	4.767608241
H	-8.601841782	2.410792974	3.675541609
H	-7.558796329	2.156957975	5.076919193
H	-6.928780491	1.649088343	2.012364211
H	-4.277297626	2.413796344	-0.408809970
H	-4.116765484	1.049515288	0.681521116
H	-5.696415610	1.407285109	-0.046297218
S	0.616406052	0.452883410	3.514690476
C	1.592851020	-0.085516723	2.212136508
N	1.345959091	0.088334789	0.900671760
C	2.343022542	-0.474586263	0.129274823
C	3.252840580	-1.013461515	0.979460913
N	2.774611763	-0.781018493	2.272050439
H	0.465355153	0.568953302	0.623769882
H	2.312121046	-0.446250769	-0.946250086
H	4.197391343	-1.509762289	0.821446276
C	3.498296619	-1.059291143	3.495670436
H	4.083469471	-1.970467410	3.365781926
H	2.774571672	-1.171562663	4.303836684
S	6.592080274	-1.096078057	2.527548410
C	5.729185636	0.056198240	3.399006747
N	4.417411882	0.013133352	3.826885542
C	4.070201385	1.196843037	4.480757449
C	5.174457651	1.977875820	4.497810940
N	6.176684727	1.269452134	3.843625912
H	3.064227566	1.362722997	4.833505160
H	5.336461841	2.958862888	4.913754190
H	7.122941987	1.572817902	3.674735090
C	-1.840387217	2.633739461	4.324511523
C	-2.590200334	1.538893721	3.824712841
H	-2.272696103	3.628528496	4.324296942
H	-1.082127481	2.466310696	5.089233674
H	-3.605737529	1.684874019	3.471345317
H	-2.407140943	0.546996008	4.238735064

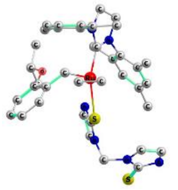

5a‡$_{cis}$ (419.84i cm^{-1})

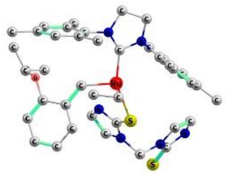

100
RuC40N6H50S2O

Ru	-0.557038077	2.244548871	2.355795827
C	-1.960494089	0.867717869	1.924205916
H	-2.826278214	1.202147025	1.360560752
C	-2.025287463	-0.574261920	2.188188150
C	-2.821006065	-1.432877260	1.374429556
C	-2.924097545	-2.800045325	1.638400617
C	-2.220267250	-3.387484645	2.691223075
C	-1.424485110	-2.574720792	3.500507903
C	-1.346475636	-1.207986613	3.259165562
H	-3.574706999	-3.409056275	1.018061857
H	-2.309708754	-4.453431236	2.879847854
H	-0.871835163	-3.002980997	4.333324165
H	-0.725853249	-0.593056129	3.900418835
O	-3.521975499	-0.875298598	0.314176777
C	-3.355317848	-1.507918124	-0.966714539
C	-1.942289083	-1.256926066	-1.493828769
C	-4.442895474	-0.961308710	-1.880080881
H	-3.506139381	-2.591129336	-0.866380682
H	-1.215561749	-1.677396864	-0.792557493
H	-1.794368848	-1.729365282	-2.471152351
H	-1.759085952	-0.182150425	-1.585867048
H	-5.424061372	-1.081868709	-1.411676546
H	-4.282728468	0.100327260	-2.079724459
H	-4.440462923	-1.495626261	-2.835683448
C	-1.346463118	3.823417689	1.450307745
N	-0.409422957	4.810430812	1.193116250
C	-0.996625141	6.108593484	0.837620594
C	-2.426130412	5.694620117	0.457196588
N	-2.546765620	4.364849257	1.082545314
H	-0.986890987	6.805520731	1.686865804
H	-0.450704928	6.574015950	0.010465771
H	-3.189325685	6.377298354	0.842754348
H	-2.559440107	5.618769319	-0.630769028
C	0.903595185	4.691116325	1.753184915
C	1.941980653	4.173444694	0.945758090
C	1.642174815	3.681204932	-0.448069174
C	3.235558669	4.135800548	1.462520034
C	3.525517170	4.602543175	2.754146899
C	4.941846452	4.578383566	3.282458786
C	2.481411066	5.091740929	3.535599165
C	1.163466106	5.147931004	3.060458022
C	0.095164929	5.768712634	3.929407437
H	1.184125954	4.466860915	-1.059563773
H	0.928850304	2.850407450	-0.401079821
H	2.551637658	3.338001100	-0.949440790
H	4.037444090	3.742496952	0.842002220
H	5.221002350	3.584755753	3.652516213
H	5.071925150	5.282271184	4.109559397
H	5.660850694	4.846144844	2.499625813
H	2.691381862	5.457428123	4.538882848
H	0.166290205	6.864383743	3.910744013
H	-0.908539326	5.489234175	3.610282450
H	0.213902224	5.452492973	4.970195476
C	-3.798115858	3.682147023	1.026029469
C	-4.736396583	3.901469699	2.047104940
C	-4.420262813	4.824730859	3.199986121
C	-5.961512246	3.230908318	1.979380305
C	-6.267926474	2.371911274	0.920762281
C	-7.568216667	1.603624621	0.891052748
C	-5.326090281	2.204838881	-0.098971151
C	-4.087001929	2.847630975	-0.066631004
C	-3.048536033	2.616273339	-1.136191861
H	-5.202363190	4.781035088	3.962671358
H	-3.468929568	4.559732549	3.670023371
H	-4.334784566	5.867529819	2.870718154
H	-6.687637828	3.376107840	2.776586518
H	-8.314259850	2.043685135	1.559586988
H	-7.992508994	1.570889768	-0.118256321
H	-7.408394796	0.565991725	1.209400311
H	-5.558534639	1.543645905	-0.928299751
H	-2.626322307	3.555411900	-1.508031532
H	-2.217354835	2.032478763	-0.723684205
H	-3.470102140	2.077179530	-1.987434394
S	1.398283303	0.900739361	3.266573459
C	1.706450585	-0.304489168	2.098496047
N	0.928884087	-0.618148314	1.037069530
C	1.492691502	-1.643951239	0.295212879
C	2.652415456	-1.987778261	0.906141144
N	2.770014093	-1.169912794	2.031295748
H	0.044579598	-0.123599379	0.880355022
H	1.019699921	-2.038022562	-0.588770741
H	3.420819050	-2.704027978	0.659245952
C	3.929512982	-1.101178366	2.896719744
H	4.351244089	-2.100078604	3.016142925
H	3.611333334	-0.693110661	3.857171839
S	6.465766632	-2.327714174	1.285984373
C	6.036843404	-0.733119961	1.607581862
N	4.962044954	-0.255971305	2.331399427
C	4.923951759	1.139155006	2.305211263
C	6.001679252	1.549240790	1.597418934
N	6.674547399	0.402688458	1.189085078
H	4.109080352	1.685550787	2.754939527
H	6.346014281	2.541508469	1.357689544
H	7.513956752	0.357605868	0.632949462
C	-1.308086225	2.614803926	4.309786666
C	-2.350140978	1.768074180	3.770633068
H	-1.554571986	3.660598883	4.484833079
H	-0.682587630	2.195649060	5.102102645
H	-3.286639345	2.230223193	3.473777685
H	-2.486632412	0.793328395	4.232700868

6a$_{cis}$

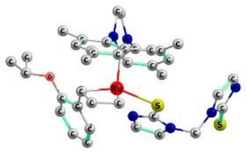

100
RuC40N6H50S2O

Ru	-1.200692426	1.163289459	2.027170172
C	-3.025895728	0.365470145	1.208134964
H	-3.564304564	0.818903990	0.386189548
C	-2.111265988	-0.687709026	0.830450235
C	-1.718314654	-0.886425416	-0.545503594
C	-0.745445018	-1.806021426	-0.892608007
C	-0.127438838	-2.611354928	0.092729316
C	-0.502965489	-2.474497821	1.418384871
C	-1.472600516	-1.524560008	1.796454653
H	-0.462301478	-1.923173516	-1.934097482
H	0.618983855	-3.343330234	-0.203479899
H	-0.052381578	-3.102983272	2.182268065
H	-1.830734009	-1.524647794	2.819756172
O	-2.367217985	-0.089472262	-1.454928133
C	-2.682712865	-0.595528542	-2.764893082
C	-1.697844078	-0.047711016	-3.797520366
C	-4.118446253	-0.162188528	-3.048902460
H	-2.628922329	-1.692333782	-2.741316209
H	-0.674585183	-0.370747814	-3.589492603
H	-1.967513697	-0.386939359	-4.803686327
H	-1.715261950	1.046974170	-3.784235275
H	-4.793929896	-0.572331924	-2.293157988
H	-4.192635283	0.929886907	-3.017314655
H	-4.441948097	-0.505617107	-4.037192478
C	-1.428210199	2.974135500	1.363191559
N	-0.381323971	3.600709563	0.695916674
C	-0.823107449	4.700177509	-0.166770099
C	-2.160821561	5.061641764	0.481011686
N	-2.509248805	3.806154935	1.158898619
H	-0.100336824	5.522140211	-0.159201636
H	-0.951976715	4.361413829	-1.206383361
H	-2.057551965	5.881346123	1.208112453
H	-2.929137961	5.342765673	-0.244768668
C	0.944921912	3.081974028	0.587310809
C	1.299673858	2.209441253	-0.453732740
C	0.296300382	1.768982539	-1.492514971
C	2.628679610	1.767259167	-0.525565656
C	3.593089374	2.171180070	0.398669268
C	5.022516139	1.690666838	0.311044748
C	3.210389569	3.056991301	1.416016436
C	1.903479121	3.530490032	1.517714778
C	1.491441361	4.497895839	2.598130164
H	0.316451959	2.436914948	-2.364416870
H	-0.724665924	1.750222930	-1.108921342
H	0.526421749	0.761726662	-1.848794669
H	2.909564685	1.082393498	-1.324044333
H	5.680778344	2.485174272	-0.063070741
H	5.117936103	0.837161688	-0.366680076
H	5.406215226	1.387230602	1.291141141
H	3.950031992	3.380100286	2.144941599
H	1.076019963	5.417338198	2.169489155
H	0.713348086	4.053508315	3.225954544
H	2.341426438	4.764210700	3.230354740
C	-3.795424868	3.664083360	1.751410141
C	-3.972001238	3.955870961	3.116098947
C	-2.806057592	4.431059867	3.950055267
C	-5.242228730	3.794277644	3.672076679
C	-6.335070212	3.378593424	2.902507471
C	-7.683457715	3.144613944	3.544091364
C	-6.139342635	3.150925124	1.538334386
C	-4.881092156	3.288237171	0.942912116
C	-4.706209420	3.041636534	-0.537179950
H	-3.099277475	4.562049269	4.995388431
H	-1.978842130	3.715765078	3.903055847
H	-2.424691825	5.391682242	3.583171473
H	-5.383446970	3.992642541	4.732727846
H	-7.873135730	3.856204168	4.354613235
H	-8.497056984	3.231846670	2.817176074
H	-7.738173211	2.137679475	3.977407416
H	-6.981052791	2.836595087	0.924072953
H	-4.841466413	3.969028945	-1.109548229
H	-3.710519487	2.652543594	-0.761509890
H	-5.445701577	2.323321057	-0.904051793
S	0.647041836	1.189489854	3.732048315
C	1.875659323	0.232653064	3.036449961
N	1.835016973	-0.392519006	1.837178156
C	3.005098271	-1.089424784	1.590300655
C	3.812191261	-0.898152485	2.659770540
N	3.107326975	-0.088523254	3.555927411
H	0.997407522	-0.396196619	1.265911913
H	3.153940664	-1.643958584	0.679087433
H	4.822510534	-1.207429899	2.871525314
C	3.580815678	0.345741906	4.852595403
H	4.328828037	-0.367863920	5.201415207
H	2.731800919	0.380530150	5.537225045
S	6.696119167	0.748587911	4.078703457
C	5.515137360	1.875639476	4.485253278
N	4.195445688	1.658427531	4.821593199
C	3.547733395	2.863306384	5.090076957
C	4.462885189	3.848127515	4.941143608
N	5.654021252	3.233553378	4.568871916
H	2.492932894	2.890890312	5.310133688
H	4.371474352	4.916093902	5.052044055
H	6.537726710	3.683191814	4.386501864
C	-2.648372943	0.895692674	3.503760580
C	-3.724151299	0.326636178	2.571197627
H	-2.965213818	1.786606686	4.044368009
H	-2.235231744	0.176716076	4.225666882
H	-4.612143346	0.964363191	2.547343242
H	-4.058352687	-0.687941352	2.847116402

7a‡ (292.02i cm^{-1})
from **6a**$_{cis}$

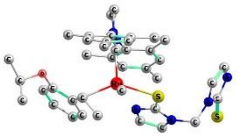

100
RuC40N6H50S2O

Ru	-1.367390437	1.128092027	2.068549397
C	-2.544847792	-0.317271944	0.935009980
H	-3.498230554	0.009040797	0.530603278
C	-1.622909914	-0.866325102	-0.073792673
C	-1.873126996	-0.716012290	-1.461883307
C	-0.998607088	-1.220411702	-2.424303839
C	0.164580417	-1.900246917	-2.054650250
C	0.428884037	-2.083081056	-0.697034686
C	-0.452512665	-1.589247816	0.265906547
H	-1.236176330	-1.056801166	-3.472049633
H	0.841320531	-2.282185850	-2.813406714
H	1.308568052	-2.639557536	-0.380761597
H	-0.262625546	-1.811343317	1.311002977
O	-3.004265178	-0.033831410	-1.869827583
C	-4.019872685	-0.829030256	-2.523945405
C	-4.913417624	0.145308561	-3.278158121
C	-4.784013395	-1.669403510	-1.502216414
H	-3.526822896	-1.500914404	-3.241832748
H	-4.324047769	0.742327822	-3.979928702
H	-5.683988895	-0.397021507	-3.835871260
H	-5.407431978	0.825466014	-2.577966770
H	-4.097460435	-2.301039608	-0.931082000
H	-5.317464780	-1.021846411	-0.799104707
H	-5.512801815	-2.315045547	-2.005142633
C	-1.073267166	2.818658553	1.027817849
N	-0.004212426	3.216792128	0.250307591
C	-0.273709065	4.433674230	-0.535266885
C	-1.527884858	4.990204815	0.154417638
N	-2.001593086	3.822906076	0.909216184
H	0.577153339	5.120582581	-0.495246137
H	-0.461962119	4.179620516	-1.587501680
H	-1.297421434	5.827603447	0.830150241
H	-2.287817561	5.331030378	-0.555486134
C	1.330004880	2.735739226	0.418130100
C	1.886541748	1.865078667	-0.530248932
C	1.082939529	1.443911574	-1.732688154
C	3.204311870	1.427641846	-0.341654124
C	3.971335366	1.848052755	0.748370629
C	5.394819401	1.378478326	0.937377755
C	3.399082240	2.745450900	1.659244068
C	2.093158303	3.213364606	1.503268948
C	1.510362534	4.236633679	2.444965301
H	1.043578670	2.251810154	-2.475166613
H	0.053634744	1.204737433	-1.458853047
H	1.516586507	0.566596910	-2.215749753
H	3.638336659	0.742244960	-1.067370270
H	6.107657009	2.138663421	0.592278088
H	5.594571859	0.463981519	0.369968194
H	5.613648831	1.179890440	1.991787627
H	3.987551949	3.091385840	2.506824078
H	1.234030381	5.151744654	1.906671361
H	0.607475500	3.850601923	2.922511775
H	2.227133615	4.501046638	3.225328011
C	-3.290431524	3.825687718	1.519596287
C	-3.437882478	4.271115986	2.843569868
C	-2.234285435	4.687057248	3.652116199
C	-4.713309936	4.252427735	3.413702326
C	-5.829754930	3.800441128	2.702500738
C	-7.190306707	3.737460404	3.358326721
C	-5.650694370	3.375215016	1.382902255
C	-4.392061058	3.381630788	0.773381633
C	-4.206304463	2.889058829	-0.638825475
H	-2.533176906	5.124832301	4.609192662
H	-1.606093965	3.811317037	3.848980324
H	-1.613077619	5.416054911	3.121309306
H	-4.836583540	4.586386967	4.442277681
H	-7.313007968	4.525227504	4.108737832
H	-7.996478937	3.838218925	2.624588291
H	-7.333779081	2.777045720	3.869482663
H	-6.510266696	3.024070375	0.814910860
H	-3.773025050	3.660704498	-1.286571578
H	-3.535333304	2.026350067	-0.672769702
H	-5.165425709	2.595957963	-1.074462452
S	0.296666037	1.282593252	4.055745955
C	1.602062002	0.347885598	3.506345433
N	1.753625425	-0.230029744	2.287465748
C	2.947103059	-0.929820357	2.202614978
C	3.569471266	-0.795119793	3.395208766
N	2.735860378	-0.012213041	4.200053617
H	1.084945119	-0.120067350	1.535588553
H	3.245248920	-1.432525355	1.298126889
H	4.530491984	-1.126920613	3.751353035
C	3.057121562	0.458937362	5.529684291
H	3.749884263	-0.246612385	5.990269996
H	2.133500369	0.529870918	6.106294429
S	6.255193484	0.809463912	5.117885915
C	5.040190090	1.958611696	5.303451617
N	3.690285946	1.765281037	5.514001926
C	3.024722879	2.987935495	5.606004396
C	3.956828636	3.959414528	5.475002327
N	5.177533566	3.319051857	5.289505196
H	1.952920520	3.031467384	5.710470829
H	3.861125645	5.032740891	5.488885852
H	6.078683696	3.754298796	5.167823422
C	-2.799222821	1.147319245	3.258261669
C	-2.546409538	-0.849552459	2.293570961
H	-3.851931835	1.152735639	2.955189119
H	-2.667583151	1.232195949	4.347402377
H	-3.509038090	-0.999221045	2.772454196
H	-1.843031546	-1.627076517	2.582712908

8a
from **6a**$_{cis}$

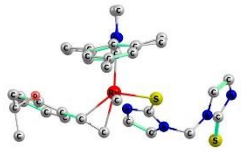

100
RuC40N6H50S2O

Ru	-1.302873495	1.355196524	1.978864757
C	-1.880103184	-0.589737163	1.066869150
H	-2.957101147	-0.588254735	0.947111600
C	-1.171936333	-0.931280903	-0.177797445
C	-1.832097011	-0.828803276	-1.428421414
C	-1.205906102	-1.196026541	-2.618293478
C	0.105523474	-1.678382133	-2.611592576
C	0.779342893	-1.793152130	-1.395506926
C	0.146250389	-1.430696003	-0.207319904
H	-1.745963670	-1.081835022	-3.553806516
H	0.588072025	-1.959014446	-3.543686587
H	1.793795275	-2.184724015	-1.368461610
H	0.662211587	-1.590466060	0.733368224
O	-3.106840553	-0.303336488	-1.446546338
C	-4.160519653	-1.137028061	-1.971213782
C	-5.386294873	-0.239880927	-2.066678567
C	-4.380910103	-2.353653139	-1.074291525
H	-3.881644528	-1.478916973	-2.979314419
H	-5.188181653	0.611144222	-2.724432699
H	-6.241103267	-0.799020476	-2.460404377
H	-5.643417906	0.145597049	-1.074818854
H	-3.455048026	-2.926501084	-0.969898900
H	-4.702908860	-2.037881844	-0.077007828
H	-5.147243355	-3.010341473	-1.500125023
C	-1.161295265	3.275124991	1.159291505
N	0.015763049	3.790189941	0.652810311
C	-0.142417457	5.072564419	-0.052413332
C	-1.532266393	5.516197986	0.413824685
N	-2.080032472	4.271850642	0.979090321
H	0.649826481	5.774357982	0.227829967
H	-0.096493699	4.924016841	-1.140377951
H	-1.480039051	6.300962994	1.180087926
H	-2.165049519	5.880698335	-0.401922443
C	1.205656812	3.035014314	0.427291650
C	1.264937922	2.124189007	-0.643737563
C	0.095361156	1.944654294	-1.578395111
C	2.464370870	1.429342163	-0.862606491
C	3.600255975	1.657978473	-0.081852751
C	4.891047127	0.915629369	-0.339226603
C	3.519940076	2.595771264	0.954229644
C	2.339623186	3.289446342	1.226600882
C	2.289877234	4.311780043	2.335122034
H	-0.023606287	2.833606018	-2.211084251
H	-0.839246635	1.798969304	-1.033233689
H	0.248089366	1.084297726	-2.229756340
H	2.501813777	0.701516608	-1.669383197
H	5.656371129	1.584135640	-0.751942296
H	4.749272514	0.096868112	-1.050986638
H	5.297577324	0.496548341	0.588245899
H	4.393226378	2.775610110	1.578405614
H	2.517865308	5.313964974	1.948568252
H	1.299664855	4.341684786	2.791759804
H	3.023353923	4.080647747	3.113635689
C	-3.460853364	4.219826309	1.328517002
C	-3.892487561	4.779058131	2.542311028
C	-2.892907061	5.360528921	3.514572179
C	-5.251906689	4.712236638	2.860789377
C	-6.176319638	4.098970400	2.008161215
C	-7.629505652	3.973330325	2.404004761
C	-5.714313244	3.564613043	0.801138607
C	-4.364247189	3.613211693	0.440560693
C	-3.876442167	2.974907392	-0.833805114
H	-3.343168537	5.502474535	4.500987717
H	-2.030999421	4.692917661	3.620895199
H	-2.513047687	6.335150037	3.183011378
H	-5.592964769	5.124441480	3.808555904
H	-7.942678857	4.794803545	3.056310337
H	-8.285876134	3.964741331	1.527852891
H	-7.804976711	3.037914052	2.950931825
H	-6.420760857	3.085837751	0.125557115
H	-3.145975231	3.606739526	-1.350660434
H	-3.379269898	2.024008262	-0.611292621
H	-4.706230778	2.775482734	-1.517756644
S	0.397695549	1.693406257	3.821844831
C	1.638766251	0.583556944	3.487061041
N	1.996929724	0.086157996	2.274970519
C	3.085908143	-0.765575616	2.380223549
C	3.421068958	-0.818333765	3.690547173
N	2.517657032	0.001311250	4.371772898
H	1.572614332	0.383214424	1.404619120
H	3.514049531	-1.248374236	1.517604732
H	4.213508634	-1.325663950	4.217634213
C	2.571861629	0.312798370	5.785621697
H	2.943408043	-0.557677192	6.327680677
H	1.565303300	0.578351953	6.112642604
S	5.686885679	-0.159354241	6.555517453
C	4.812251378	1.261581653	6.344709269
N	3.469694677	1.418550551	6.059208623
C	3.147720798	2.770549475	5.923289712
C	4.281221980	3.472946701	6.151791999
N	5.282330444	2.544213187	6.412663306
H	2.150181742	3.082285722	5.657294304
H	4.461262193	4.535646273	6.158858107
H	6.249496302	2.737685032	6.621288151
C	-2.998216343	1.478727892	2.648934079
C	-1.337291819	-0.811925276	2.366636314
H	-3.657500806	0.599089974	2.743997554
H	-3.410748004	2.380886630	3.107852358
H	-2.018672542	-1.060401899	3.177733023
H	-0.355948589	-1.267633077	2.486922042

1b

94

RuC38N6H46S2O

Ru	-0.898873593	1.749398473	1.647281630
C	-1.038533022	0.805430303	0.025297400
H	-0.826517189	1.140178607	-0.996167551
C	-1.209743261	-0.648081277	0.094490867
C	-1.496132218	-1.261242102	1.336009296
C	-1.584541883	-2.640887911	1.486279588
C	-1.412262918	-3.465241751	0.369474793
C	-1.141858567	-2.895007253	-0.877238323
C	-1.038494664	-1.511751341	-1.007710894
H	-1.769744948	-3.074992926	2.463725895
H	-1.472534684	-4.543491692	0.482069506
H	-0.998435896	-3.533461656	-1.745046357
H	-0.805053954	-1.069742475	-1.973549944
O	-1.583839450	-0.389206439	2.421247141
C	-2.741857900	-0.534481863	3.304902590
C	-4.019813647	-0.202725088	2.544800685
C	-2.504210393	0.373374533	4.500236119
H	-2.762516509	-1.577324479	3.643808096
H	-4.143952726	-0.864147493	1.682754846
H	-4.888182242	-0.320303966	3.202027188
H	-3.973253426	0.829736980	2.190363526
H	-1.530794867	0.173941679	4.953924670
H	-2.534330043	1.425334481	4.202972209
H	-3.289508736	0.204432029	5.245329401
C	-0.770821302	3.510787126	0.837859036
N	-0.212286479	4.046149807	-0.305641075
C	-0.370997980	5.507315391	-0.404192099
C	-1.485699479	5.785081715	0.611059567
N	-1.435367743	4.574056752	1.429730318
H	0.566748049	6.014359322	-0.133080836
H	-0.633964917	5.810179346	-1.422758320
H	-1.303329236	6.683661180	1.210213603
H	-2.465829998	5.894270219	0.123341901
C	0.782007280	3.436199207	-1.125624546
C	0.423850388	3.048466098	-2.428566124
C	-1.003898989	3.188903103	-2.901182905
C	1.411529202	2.525752531	-3.268916459
C	2.738937748	2.399828572	-2.848833159
C	3.803749692	1.865130143	-3.780158750
C	3.066145452	2.797347070	-1.548240162
C	2.108995707	3.319300411	-0.673397658
C	2.476326639	3.701595577	0.737735636
H	-1.260450474	4.238498822	-3.094058955
H	-1.704037368	2.821925590	-2.145525009
H	-1.166275929	2.634308115	-3.829851943
H	1.136904113	2.209991907	-4.273562861
H	4.551064467	1.275111840	-3.237869401
H	4.340938276	2.681661748	-4.279486098
H	3.373071975	1.233357692	-4.563918953
H	4.091576980	2.688052033	-1.200733753
H	2.206335576	4.740225417	0.960462822
H	1.925320975	3.079593320	1.450581340
H	3.549993553	3.583820040	0.910934525
C	-2.286591717	4.382863477	2.559603233
C	-1.813828300	4.777126564	3.824643779
C	-0.480306836	5.471932241	3.962451706
C	-2.617151531	4.536286579	4.944827270
C	-3.873596174	3.938149950	4.828507263
C	-4.695246917	3.612839513	6.053548863
C	-4.342649325	3.610623920	3.550574134
C	-3.574157693	3.826323024	2.404781895
C	-4.104408029	3.468032967	1.036919546
H	-0.148463610	5.480204706	5.004524895
H	0.285213578	4.976840892	3.361623042
H	-0.547983231	6.514781464	3.624829435
H	-2.247505347	4.816642572	5.928989208
H	-4.525131115	2.574603657	6.366052326
H	-4.432669881	4.255710702	6.899421031
H	-5.767674337	3.722029419	5.861085814
H	-5.329245409	3.163937016	3.445168894
H	-4.133147503	4.342824450	0.376799662
H	-3.454816371	2.718527345	0.570880391
H	-5.119336139	3.065620181	1.104995162
S	0.582422892	2.132488873	3.801208484
C	2.070666792	1.313157716	3.750279160
N	3.182270998	1.731951629	4.424642992
C	4.238313705	0.845471227	4.263201917
C	3.787078315	-0.149640741	3.465104357
N	2.460514840	0.145016367	3.148977796
H	3.182122594	2.587204309	4.958138255
H	5.199476077	1.000233285	4.725312676
H	4.252494881	-1.051751254	3.095275498
C	1.615077134	-0.713084979	2.322467656
H	0.674922865	-0.182406361	2.178601238
H	1.464577897	-1.673390490	2.818904322
S	3.405849448	-3.365456412	1.722164727
C	2.956936160	-2.083270382	0.721889423
N	2.217408233	-0.962893508	1.035868569
C	2.118699154	-0.106286119	-0.061394287
C	2.783101356	-0.695375547	-1.080404947
N	3.282444651	-1.900143338	-0.590910923
H	1.539950826	0.803072425	0.001572761
H	2.928800756	-0.370827380	-2.096681300
H	3.829851594	-2.580471657	-1.093589455

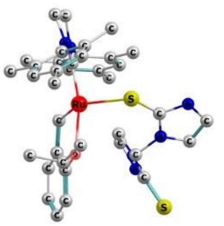

2b‡ (5.05i cm^{-1})

94

RuC38N6H46S2O

Ru	-0.969596381	1.692644795	1.296883734
C	-0.317486066	0.731483001	-0.138556553
H	0.565557905	1.037639669	-0.710450895
C	-0.681751908	-0.648646637	-0.519893893
C	-1.960708951	-1.250780516	-0.385958463
C	-2.177492931	-2.556779683	-0.841371635
C	-1.135196842	-3.322655102	-1.364534596
C	0.137669542	-2.761891758	-1.482192614
C	0.341608774	-1.444130381	-1.087453082
H	-3.182848771	-2.958499671	-0.809875796
H	-1.326188376	-4.338996658	-1.698120232
H	0.959661302	-3.340408401	-1.895447747
H	1.323735196	-0.991674230	-1.208306678
O	-3.015362668	-0.499947422	0.081782894
C	-3.610916791	-0.838439938	1.360841384
C	-4.572896545	-2.025648876	1.282658216
C	-4.327958372	0.429349032	1.812027912
H	-2.799789731	-1.072998211	2.060423211
H	-4.050274093	-2.969248948	1.111911820
H	-5.117123824	-2.116925954	2.229238184
H	-5.300468001	-1.871598263	0.478512768
H	-3.631975946	1.273438507	1.800406646
H	-5.156772590	0.656839962	1.132418511
H	-4.723608528	0.307674222	2.825482577
C	-0.640446914	3.493904191	0.633480944
N	-0.068689945	4.110314204	-0.460494297
C	-0.098951913	5.579984484	-0.384191196
C	-1.172438701	5.820124602	0.685263047
N	-1.149804477	4.540613344	1.393877471
H	0.875640251	5.983816540	-0.075350429
H	-0.355418124	6.013982476	-1.355756768
H	-0.932957730	6.651469819	1.356476854
H	-2.157399447	6.013110957	0.233808965
C	0.793321415	3.470464922	-1.398528914
C	0.231659952	2.902818141	-2.557364135
C	-1.263554364	2.913033490	-2.749338896
C	1.081204087	2.289956563	-3.480300671
C	2.466099044	2.236674612	-3.281639905
C	3.357811864	1.538484115	-4.281982405
C	2.995297663	2.813296083	-2.124202498
C	2.178139324	3.426486689	-1.165716383
C	2.778685909	4.014125400	0.090627770
H	-1.665426164	3.931517592	-2.700362419
H	-1.740086010	2.340272398	-1.945400513
H	-1.542447744	2.475343844	-3.711950539
H	0.653023848	1.836927059	-4.371869607
H	3.330057107	0.450928560	-4.138719809
H	4.400227122	1.858280694	-4.186336405
H	3.036701373	1.735883536	-5.310166596
H	4.073008385	2.797990127	-1.967408512
H	2.965794826	5.090269162	-0.015692374
H	2.110237496	3.873547839	0.944780117
H	3.735848837	3.540816250	0.328143533
C	-1.829792201	4.320273608	2.628971985
C	-1.065013180	4.312440990	3.813050961
C	0.433845475	4.466729985	3.742542740
C	-1.717976313	4.105595480	5.029539460
C	-3.100330735	3.892410898	5.093410387
C	-3.792963227	3.690254100	6.420953180
C	-3.830760367	3.893845904	3.902239412
C	-3.221038415	4.112746818	2.662186840
C	-4.033654803	4.124140416	1.389138861
H	0.876555884	4.533414538	4.741656417
H	0.866066555	3.604848728	3.218006037
H	0.721921550	5.359363902	3.177584175
H	-1.139271485	4.122758677	5.953341116
H	-4.620859031	2.979463832	6.334030890
H	-3.102581273	3.312977029	7.182041424
H	-4.210685026	4.633645663	6.795897849
H	-4.903224816	3.713170647	3.936015027
H	-4.239448913	5.151326064	1.061482078
H	-3.501507733	3.611142461	0.582988993
H	-4.995524576	3.625438153	1.535500586
S	-1.189566526	0.572496219	3.498074792
C	0.381454584	0.580842455	4.168215484
N	0.763598857	1.301316612	5.257358870
C	2.093934849	1.058664265	5.574002706
C	2.547375437	0.154342691	4.672382847
N	1.489988655	-0.133234285	3.811895623
H	0.134134675	1.960282471	5.692548698
H	2.587202456	1.533134252	6.406355008
H	3.504278046	-0.331998116	4.556719985
C	1.599312408	-0.977116589	2.622024036
H	0.687148732	-0.846252517	2.038677963
H	1.736104559	-2.019555661	2.916572148
S	4.419646640	-2.582030005	2.768905019
C	3.953667831	-1.256905937	1.840649939
N	2.745316452	-0.588525526	1.835475579
C	2.790330538	0.521215299	0.987201070
C	4.018732134	0.529351565	0.422225272
N	4.711377964	-0.561024553	0.941960881
H	1.935512763	1.178754502	0.897239235
H	4.442963824	1.191413833	-0.312762945
H	5.661096222	-0.829891187	0.738806856

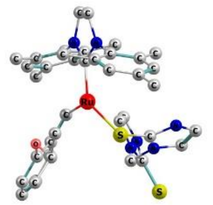

3b’

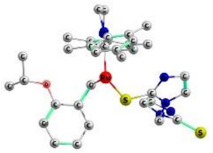

94

RuC38N6H46S2O

Ru	-0.830702672	1.315347550	1.380404763
C	-0.748211287	0.564653918	-0.294353730
H	-0.403223373	0.910858557	-1.271738955
C	-1.434361116	-0.747889242	-0.268280384
C	-2.706222942	-0.902780060	-0.884867853
C	-3.398917584	-2.110906724	-0.790528167
C	-2.842434051	-3.185088162	-0.083535408
C	-1.598614073	-3.059392382	0.524308512
C	-0.908568552	-1.848373857	0.425844582
H	-4.370891343	-2.233052900	-1.253802155
H	-3.394143984	-4.119314768	-0.019439002
H	-1.162989740	-3.892482510	1.068759887
H	0.067054541	-1.739561116	0.889121974
O	-3.175535809	0.215215398	-1.519063725
C	-4.538854170	0.279415648	-1.962648783
C	-4.670831692	-0.334950622	-3.356770608
C	-4.920463329	1.755707078	-1.934492402
H	-5.172476863	-0.262493740	-1.246547494
H	-4.334545750	-1.374996267	-3.366764730
H	-5.711473342	-0.303682451	-3.697144935
H	-4.054826621	0.227002686	-4.066315714
H	-4.783907185	2.162615360	-0.929252568
H	-4.289680163	2.325040111	-2.624594715
H	-5.966340005	1.885199875	-2.232204389
C	-0.829978386	3.146040153	0.724653230
N	-0.374378839	3.778201019	-0.416280215
C	-0.578848269	5.236951355	-0.391752049
C	-1.646200493	5.393087667	0.694823257
N	-1.479223409	4.143492973	1.437581019
H	0.355977854	5.754726146	-0.128866702
H	-0.904082615	5.605076672	-1.369544672
H	-1.485648786	6.267714414	1.333282611
H	-2.657668878	5.464960283	0.267125434
C	0.602312928	3.274256121	-1.328376689
C	0.192832068	2.963381377	-2.638806887
C	-1.270354290	3.013479272	-3.008989512
C	1.157091966	2.556057919	-3.562613450
C	2.509400622	2.458944988	-3.216261142
C	3.528925034	1.990875299	-4.229537665
C	2.884627861	2.776017847	-1.908145250
C	1.950545514	3.179636848	-0.946261745
C	2.378447948	3.473169301	0.469560019
H	-1.693725506	4.015031434	-2.871977658
H	-1.846194094	2.330579864	-2.375561202
H	-1.419066704	2.725279414	-4.053734287
H	0.846098732	2.303286530	-4.574326375
H	3.520505301	0.897072596	-4.322792978
H	4.543445047	2.290827791	-3.949144005
H	3.319992513	2.397021013	-5.224848247
H	3.933623077	2.714884802	-1.624635916
H	2.078715688	4.479615567	0.783604336
H	1.894411060	2.766854296	1.155468550
H	3.463106736	3.389488839	0.580289754
C	-1.967874395	3.964992541	2.763857451
C	-1.151068590	4.395311662	3.827257571
C	0.201091380	5.005595979	3.540004893
C	-1.604134165	4.215387072	5.136813803
C	-2.839103310	3.609432730	5.404560010
C	-3.286111552	3.366589562	6.826553576
C	-3.633675157	3.210349333	4.327696592
C	-3.224699833	3.383413969	3.000963644
C	-4.087168114	2.931992750	1.847798390
H	0.731642476	5.248977179	4.466547547
H	0.815528526	4.314587846	2.953265106
H	0.117837815	5.930321067	2.958347711
H	-0.990883802	4.565341200	5.966970822
H	-2.976498941	2.369447366	7.162589804
H	-2.850836032	4.096150281	7.516966296
H	-4.375375373	3.417225382	6.920013386
H	-4.597922361	2.746674064	4.523713230
H	-4.274888363	3.754146911	1.147434311
H	-3.572311821	2.141297008	1.285360995
H	-5.050060573	2.551177063	2.199595262
S	-0.581689506	0.346757891	3.698891669
C	0.969691762	0.941599081	4.090526609
N	1.235884474	2.082082232	4.782055419
C	2.604653105	2.272167918	4.921485229
C	3.209277405	1.207882768	4.339566420
N	2.197609170	0.387972248	3.841981580
H	0.491101883	2.713847784	5.047263443
H	3.019242859	3.129904626	5.425356677
H	4.247481657	0.931133334	4.229827044
C	2.412920247	-0.844629558	3.097579932
H	1.426855083	-1.246980242	2.862577043
H	2.986296287	-1.544594072	3.709193294
S	5.603213446	-1.382642449	2.936071569
C	4.513620511	-0.863572733	1.758402547
N	3.160413594	-0.618703236	1.882604004
C	2.626387643	-0.138368869	0.682269658
C	3.640743262	-0.113381155	-0.211282289
N	4.778964268	-0.562166590	0.453575096
H	1.591756721	0.190911952	0.609967746
H	3.650417945	0.204957504	-1.240209632
H	5.707353851	-0.656875719	0.072885623

3b

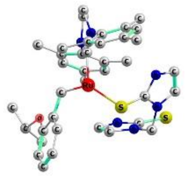

94

RuC38N6H46S2O

Ru	-0.820337796	1.226284062	1.381696702
C	-0.949580951	0.618362615	-0.370687655
H	-0.787262767	1.102866328	-1.334337052
C	-1.180004260	-0.830740723	-0.451064465
C	-0.616292243	-1.630105195	-1.485120140
C	-0.714881642	-3.023785415	-1.451572337
C	-1.403386100	-3.655899290	-0.411570999
C	-2.002324369	-2.898327846	0.592826314
C	-1.895779606	-1.508979136	0.559705226
H	-0.251811436	-3.629601921	-2.221910154
H	-1.470128661	-4.740296356	-0.399596529
H	-2.559462448	-3.382385325	1.389852789
H	-2.398141150	-0.907705176	1.317095349
O	0.056649590	-0.935753080	-2.460260787
C	0.451518613	-1.587799099	-3.679155565
C	1.868635563	-2.142590942	-3.531637621
C	0.349200372	-0.531951586	-4.772374101
H	-0.253102317	-2.399898878	-3.900415046
H	1.936061220	-2.837805179	-2.689980671
H	2.179707029	-2.668091117	-4.440599881
H	2.568425631	-1.318032152	-3.354206997
H	-0.677021311	-0.161805497	-4.847878911
H	1.003994321	0.309796606	-4.533343640
H	0.646772079	-0.948550414	-5.740115602
C	-0.678405585	3.049126886	0.784294442
N	-0.011438748	3.597039467	-0.305333917
C	-0.117643038	5.065830385	-0.366148586
C	-1.310053982	5.339396904	0.550151806
N	-1.319387138	4.132195251	1.379283312
H	0.806448987	5.532275215	0.007773258
H	-0.280165732	5.407586035	-1.392647611
H	-1.193373579	6.246780292	1.149195162
H	-2.248996025	5.425587206	-0.017150324
C	1.094521426	3.023192984	-1.010725863
C	0.978802222	2.849910709	-2.404799112
C	-0.320105601	3.127961135	-3.125098167
C	2.098002175	2.432508958	-3.129675213
C	3.328096124	2.189208894	-2.512252124
C	4.516360790	1.724585190	-3.324311916
C	3.421205139	2.386840248	-1.132371622
C	2.326660668	2.800781512	-0.364203764
C	2.489566499	3.006730556	1.119178403
H	-0.328759805	4.139814437	-3.552143794
H	-1.173642059	3.045267971	-2.448780752
H	-0.463223967	2.425226125	-3.951676180
H	2.009999519	2.312302194	-4.208043846
H	4.353357929	0.716590624	-3.728077350
H	5.431450443	1.700013785	-2.724337046
H	4.697254467	2.382610637	-4.182214146
H	4.376392788	2.239222580	-0.631116010
H	2.216089265	4.027666865	1.411564549
H	1.820164014	2.328371427	1.660708518
H	3.517598007	2.817427456	1.437765390
C	-1.932447409	4.103606081	2.665368903
C	-1.239730796	4.696147652	3.742752981
C	0.101731876	5.361000258	3.528172391
C	-1.812053546	4.653778135	5.018791140
C	-3.051970801	4.043714512	5.244325887
C	-3.628581105	3.960798254	6.638075632
C	-3.730318408	3.498633284	4.151846101
C	-3.200591943	3.525522537	2.856980895
C	-3.956214319	2.936196342	1.694356737
H	0.588614130	5.583034459	4.483566290
H	0.765773991	4.726888009	2.934529293
H	0.003523940	6.312846929	2.992935183
H	-1.283736695	5.113733124	5.852587917
H	-3.315571302	3.033582718	7.133739736
H	-3.293861276	4.795112824	7.262891146
H	-4.722857870	3.966275885	6.620827213
H	-4.703910590	3.039216560	4.308639280
H	-4.041341101	3.657298068	0.873537720
H	-3.404150782	2.071374656	1.296914043
H	-4.961410891	2.624266664	1.992035313
S	-0.575447761	0.264182549	3.803610429
C	0.857392216	1.107874501	4.177462029
N	0.932154543	2.378466145	4.652765135
C	2.252063955	2.764750308	4.834825484
C	3.022590244	1.703142065	4.497515453
N	2.158403000	0.677991725	4.109053180
H	0.104033303	2.955998604	4.748737185
H	2.521182795	3.751129439	5.174574370
H	4.091287371	1.572752303	4.440704385
C	2.573441990	-0.641802809	3.645492059
H	1.802388241	-1.356186611	3.935486048
H	3.525132985	-0.878463156	4.124013879
S	5.342002099	0.280262195	2.231254065
C	3.896672757	-0.289931076	1.566952068
N	2.752441832	-0.710277521	2.214811647
C	1.772996554	-1.085365746	1.288299616
C	2.306483952	-0.913103938	0.058586876
N	3.599807657	-0.446147418	0.248250224
H	0.793881444	-1.411778889	1.594569840
H	1.861948123	-0.999767151	-0.917356437
H	4.227888606	-0.138117551	-0.476471725

4b$_{cis}$

100
RuC40N6H50S2O

Ru	-1.109248823	1.090309965	1.573902397
C	-0.462037952	0.281659911	0.032989978
H	-0.181022678	0.866202112	-0.839908113
C	-0.188065799	-1.144731704	-0.191793979
C	0.105249147	-1.614505197	-1.499656872
C	0.505133331	-2.932082454	-1.732341078
C	0.603267778	-3.839345244	-0.677271515
C	0.280115199	-3.415726097	0.617002008
C	-0.108682694	-2.099499751	0.846627332
H	0.737470729	-3.225443385	-2.752622580
H	0.916924657	-4.862827556	-0.863750367
H	0.336700397	-4.113638882	1.448523120
H	-0.351828133	-1.766576577	1.849647175
O	0.026001698	-0.758678181	-2.580563268
C	-1.184073328	-0.867683295	-3.368177268
C	-0.901444456	-0.142939838	-4.676336439
C	-2.385891175	-0.286810594	-2.624453609
H	-1.364295425	-1.935224016	-3.570101086
H	-0.021075690	-0.565771525	-5.169406076
H	-1.758333954	-0.226533144	-5.352575615
H	-0.711788284	0.916380071	-4.481110554
H	-2.554232583	-0.806657274	-1.677954664
H	-2.218983195	0.771258753	-2.399889140
H	-3.289719451	-0.381044951	-3.236546560
C	-0.346031271	2.910565472	1.074615476
N	0.642924325	3.419880660	0.273169392
C	1.038036486	4.803048921	0.584916855
C	-0.147190497	5.288687152	1.418279368
N	-0.718248810	4.012975167	1.907825783
H	1.981116929	4.811214318	1.152180155
H	1.194399504	5.386160472	-0.327543679
H	0.141802920	5.936088385	2.252586262
H	-0.878674482	5.833276602	0.806959416
C	1.323272825	2.734692783	-0.788265021
C	0.862485300	2.928203470	-2.103886647
C	-0.368100706	3.755188532	-2.386130090
C	1.507373869	2.257777503	-3.143344209
C	2.566908771	1.379252518	-2.904870657
C	3.134739042	0.545149692	-4.025917142
C	3.022112096	1.233067693	-1.593435704
C	2.424686398	1.906524601	-0.523745221
C	2.906217499	1.678613424	0.882103491
H	-0.319477781	4.755124711	-1.941984176
H	-1.253401727	3.265112499	-1.968574480
H	-0.521874426	3.873645834	-3.462553216
H	1.146845322	2.391215677	-4.160963090
H	3.103725456	1.074704858	-4.983956805
H	2.535427401	-0.367900846	-4.132496474
H	4.170019515	0.247419642	-3.831950100
H	3.852452165	0.560019710	-1.388656823
H	3.072321035	2.617504239	1.415157259
H	2.154983635	1.108720518	1.436046609
H	3.843501853	1.117671415	0.894025556
C	-2.057776562	4.089940591	2.456860333
C	-2.231645568	4.237178789	3.845688335
C	-1.075380541	4.279861132	4.815275492
C	-3.529092293	4.333495948	4.362175746
C	-4.656287200	4.298291030	3.544088318
C	-6.049368246	4.342561312	4.124804479
C	-4.457254791	4.204448209	2.163980448
C	-3.183743880	4.110106240	1.599551623
C	-3.064699601	3.988991331	0.100845513
H	-0.689706444	3.272766710	5.005152586
H	-0.247861980	4.898203900	4.453827107
H	-1.396045333	4.695763602	5.774532001
H	-3.655535722	4.437041740	5.437900687
H	-6.464553850	3.330952456	4.216197736
H	-6.056548392	4.791888913	5.122619296
H	-6.730691599	4.915126404	3.487157527
H	-5.321227686	4.203242690	1.502487971
H	-2.277198851	4.626682204	-0.307610289
H	-2.810336992	2.957617327	-0.164304563
H	-4.006185265	4.263725773	-0.383280608
S	0.026682724	0.565528075	3.719578339
C	1.399159580	1.580245616	3.900208613
N	1.475723506	2.903210069	3.629082473
C	2.735479265	3.398788449	3.919615678
C	3.473794251	2.360386353	4.382779306
N	2.642065190	1.240392741	4.375618105
H	0.701596760	3.392354702	3.167159716
H	2.995157366	4.433676263	3.766396867
H	4.509160198	2.279376371	4.672180110
C	3.077963353	-0.123193952	4.644189249
H	2.237622023	-0.675637414	5.065083721
H	3.911903829	-0.084965435	5.346160945
S	6.146712268	0.062108256	3.620212204
C	4.807206765	-0.698817511	2.940337093
N	3.525236087	-0.793312651	3.443390420
C	2.700399074	-1.498476864	2.564076837
C	3.460154851	-1.867049691	1.510144353
N	4.740721696	-1.376636706	1.754312491
H	1.651415497	-1.626521805	2.759370279
H	3.189661018	-2.407806961	0.617270475
H	5.552491334	-1.484625824	1.166357496
C	-3.212287222	0.625334365	1.328362781
C	-2.717707686	-0.360270932	2.226840194
H	-3.461889857	0.360751521	0.304014153
H	-3.786735319	1.466869059	1.717480505
H	-2.936295551	-0.277345087	3.290914270
H	-2.559478322	-1.376996188	1.874635925

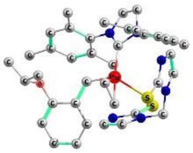

5b$^{‡}_{cis}$ (272.32i cm^{-1})

100
RuC40N6H50S2O

Ru	-0.787457101	1.073331313	1.662072867
C	-0.960937378	0.313010361	-0.132511796
H	-1.392471308	0.793579146	-1.012940090
C	-0.397193242	-0.998860816	-0.439293956
C	-0.013253480	-1.342054327	-1.764458041
C	0.653372344	-2.533875673	-2.046308920
C	0.902616311	-3.470210486	-1.038723787
C	0.447005372	-3.204066948	0.257308908
C	-0.193281740	-2.001330213	0.542126922
H	0.965101533	-2.721621285	-3.070704871
H	1.409980972	-4.402351057	-1.270454891
H	0.586860262	-3.938632156	1.046271806
H	-0.563333175	-1.817242888	1.548614882
O	-0.272469074	-0.455568525	-2.792142619
C	-1.277651832	-0.870485472	-3.741164949
C	-1.186070593	0.115695022	-4.897209955
C	-2.664169108	-0.898075468	-3.097711979
H	-1.030117203	-1.881012067	-4.101082770
H	-0.182269749	0.105123622	-5.331626635
H	-1.911058684	-0.136125461	-5.677649931
H	-1.395892224	1.128990120	-4.539487480
H	-2.674402389	-1.557756470	-2.225133150
H	-2.953513158	0.106148224	-2.771084846
H	-3.409932776	-1.261422717	-3.813181134
C	-0.245818657	2.892841939	1.261572274
N	0.615365452	3.393257493	0.307036308
C	0.975017782	4.800521151	0.512830483
C	-0.158420633	5.287258775	1.415666821
N	-0.631698224	4.028898622	2.042894344
H	1.958460142	4.883824921	1.000758797
H	1.029176024	5.340477012	-0.437756200
H	0.168643061	6.003382539	2.176444381
H	-0.961756890	5.758263826	0.834836729
C	1.311223423	2.631668680	-0.691234231
C	0.825599799	2.661715119	-2.010747569
C	-0.445671933	3.396149083	-2.358150765
C	1.503635097	1.937062301	-2.990204452
C	2.626105177	1.162739384	-2.689270877
C	3.243107303	0.277555435	-3.743243354
C	3.106688354	1.179973926	-1.378335149
C	2.477198250	1.918379443	-0.370995978
C	3.015954119	1.918729360	1.032271937
H	-0.445825738	4.431629301	-2.000980089
H	-1.307265503	2.903303326	-1.897371943
H	-0.604141574	3.412964990	-3.440403571
H	1.112965501	1.932814965	-4.004386663
H	3.250944412	0.762155098	-4.725660316
H	2.656145390	-0.644885151	-3.838950374
H	4.271136136	-0.002884984	-3.493282553
H	3.992735624	0.600718081	-1.125092554
H	3.203205038	2.933798170	1.394551700
H	2.285494561	1.460193399	1.704802914
H	3.953545801	1.363186506	1.103187499
C	-1.969459288	4.098975714	2.600319451
C	-2.136948937	4.365418988	3.969508128
C	-0.973079041	4.543276126	4.912887085
C	-3.433135179	4.452520342	4.492517669
C	-4.565451995	4.293470337	3.696854468
C	-5.954617549	4.331726941	4.288361728
C	-4.374614317	4.083947314	2.328099420
C	-3.102707278	3.998616425	1.758251290
C	-2.989722312	3.783002994	0.268120120
H	-0.556435866	3.571690631	5.196013509
H	-0.169851783	5.145345513	4.476546325
H	-1.295460369	5.045460979	5.829339331
H	-3.553829280	4.647429075	5.556187399
H	-6.321588735	3.316841197	4.486168902
H	-5.974300701	4.877284489	5.236799414
H	-6.668861258	4.806405631	3.607211738
H	-5.243827838	3.988556276	1.680179904
H	-2.337818864	4.519048188	-0.211243269
H	-2.555538705	2.801773289	0.060395806
H	-3.972749785	3.849608885	-0.206111437
S	-0.032657994	0.849564647	4.054182449
C	1.438755382	1.711139263	4.162547100
N	1.622754691	3.021621126	3.869004959
C	2.943464066	3.391587804	4.066920126
C	3.604738288	2.292297764	4.503158459
N	2.668330522	1.257644096	4.569648225
H	0.883061638	3.551301918	3.400112471
H	3.292887985	4.394342112	3.882088609
H	4.643146672	2.114219148	4.732828347
C	2.999527169	-0.153514093	4.717874267
H	2.118874231	-0.670097558	5.098801840
H	3.840429076	-0.247237549	5.405908002
S	6.088911412	-0.241139851	3.742503543
C	4.678671636	-0.751292951	2.973642508
N	3.384165490	-0.739819568	3.451841567
C	2.496661931	-1.227336909	2.485981247
C	3.232251400	-1.570862872	1.405666858
N	4.556802200	-1.279626061	1.718845482
H	1.433134433	-1.232470113	2.650065232
H	2.920607887	-1.970862108	0.454202044
H	5.362304904	-1.424631382	1.130519705
C	-2.809959470	0.096384344	1.005546492
C	-2.738670866	0.501143153	2.377611888
H	-2.838993539	-0.968268413	0.777084929
H	-3.379603556	0.709183074	0.312041272
H	-3.260374559	1.406050473	2.677349504
H	-2.705149516	-0.273079635	3.148543240

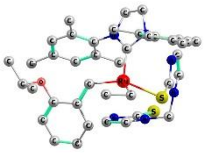

6b$_{cis}$

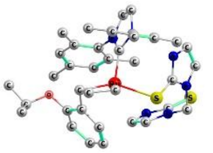

100
RuC40N6H50S2O

Ru1	-0.621791629	0.918119452	1.410152631
C2	-2.060917477	0.387457860	-0.118091942
H3	-2.173240373	0.887321296	-1.075166113
C4	-1.211538834	-0.783897984	-0.153281010
C5	-0.385379322	-1.080156336	-1.295593875
C6	0.589936862	-2.053508892	-1.252900357
C7	0.785721074	-2.835638332	-0.092325518
C8	-0.046561969	-2.638877482	0.997166106
C9	-1.041298942	-1.639078798	0.983394242
H10	1.191645287	-2.218968175	-2.143620016
H11	1.545225867	-3.612589299	-0.079257437
H12	0.044997180	-3.274063137	1.875441875
H13	-1.763948072	-1.615626097	1.791152097
O14	-0.587774321	-0.351791468	-2.452445384
C15	-1.254749009	-1.051015821	-3.525290380
C16	-1.115406302	-0.161029648	-4.753146933
C17	-2.712940403	-1.343775778	-3.175078410
H18	-0.729861854	-2.003048083	-3.700347253
H19	-0.061042133	0.000233495	-4.994878537
H20	-1.608823176	-0.616752982	-5.617790250
H21	-1.578808576	0.812774095	-4.561516155
H22	-2.781504508	-1.939561987	-2.260746917
H23	-3.261623748	-0.410252275	-3.015263457
H24	-3.194648598	-1.899909407	-3.986712101
C25	-0.293728730	2.822434975	1.289549201
N26	0.693784478	3.317896336	0.457422016
C27	1.082647187	4.702094723	0.735061871
C28	-0.081752529	5.190703247	1.605167861
N29	-0.684703200	3.925196742	2.087856108
H30	2.044379518	4.745681730	1.268291553
H31	1.192101202	5.272247545	-0.193558881
H32	0.239110148	5.812840256	2.447510084
H33	-0.808056505	5.769857624	1.022376089
C34	1.357738598	2.564227531	-0.570868842
C35	0.812762520	2.568244770	-1.870144918
C36	-0.478508901	3.275972637	-2.184862874
C37	1.470196594	1.862096532	-2.876779146
C38	2.639333691	1.142253025	-2.630313141
C39	3.261443148	0.303791974	-3.720013441
C40	3.182459070	1.186820570	-1.345459655
C41	2.570195965	1.899610940	-0.309589601
C42	3.206701592	1.930878923	1.053979303
H43	-0.554922733	4.244924871	-1.684428445
H44	-1.322693255	2.668801094	-1.847592891
H45	-0.584318096	3.434402073	-3.262200307
H46	1.038014986	1.851028155	-3.873755744
H47	3.197734175	0.797779180	-4.695701002
H48	2.731648905	-0.653730574	-3.807982891
H49	4.314532198	0.084220581	-3.517469571
H50	4.109769499	0.656721965	-1.136932578
H51	3.288435189	2.946130847	1.449323609
H52	2.611184444	1.349847924	1.758436851
H53	4.211411972	1.503364342	1.031816257
C54	-2.033498531	4.044384816	2.598256812
C55	-2.249527189	4.099037480	3.983809750
C56	-1.118405048	3.959543604	4.970758117
C57	-3.558485964	4.233177758	4.459684720
C58	-4.654192647	4.311855261	3.599535492
C59	-6.063149015	4.393828622	4.138111414
C60	-4.411559276	4.275508275	2.224249095
C61	-3.121346643	4.147052518	1.703897662
C62	-2.933042939	4.057297755	0.210648863
H63	-0.743992367	2.930047491	4.975415373
H64	-0.281926929	4.627849030	4.737916265
H65	-1.453671999	4.197435379	5.983950013
H66	-3.723921634	4.263176066	5.534704982
H67	-6.486099324	3.390103020	4.271630068
H68	-6.094065088	4.892643514	5.112250724
H69	-6.724370616	4.937097677	3.455129035
H70	-5.250519494	4.335267776	1.534071812
H71	-2.281473707	4.846874802	-0.178170564
H72	-2.472202380	3.102827444	-0.051099691
H73	-3.892508685	4.136002447	-0.307962364
S74	0.334937023	0.418693297	3.704213077
C75	1.649876871	1.444848326	4.025828820
N76	1.689279550	2.792714653	3.876016768
C77	2.944024819	3.289476002	4.195045745
C78	3.708835768	2.232103132	4.562438510
N79	2.902730632	1.096790597	4.468135238
H80	0.899745896	3.294805984	3.470693549
H81	3.180591195	4.339160382	4.132254062
H82	4.747502558	2.153593065	4.840361608
C83	3.369500756	-0.282732640	4.547612383
H84	2.543876552	-0.901712658	4.897272290
H85	4.212675646	-0.322497452	5.238009753
S86	6.430589539	0.066380986	3.569852815
C87	5.100488851	-0.608468783	2.786975666
N88	3.814986197	-0.777652864	3.261664056
C89	2.999186000	-1.358500640	2.283148078
C90	3.775337127	-1.575531162	1.199103559
N91	5.050529012	-1.119159278	1.520005505
H92	1.943887143	-1.512994166	2.434123661
H93	3.515737932	-1.977880545	0.234256230
H94	5.868810940	-1.143609701	0.931835334
C95	-3.236598198	0.462651875	0.866360998
C96	-2.554655280	0.802121145	2.201145263
H97	-3.824446696	-0.470895557	0.890520272
H98	-3.922026044	1.260054572	0.556984818
H99	-2.933878337	1.704287693	2.675613949
H100	-2.588555294	-0.012418046	2.939451754

7b‡ (273.29*i* cm^{-1})

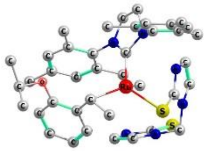

100
RuC40N6H50S2O

Ru	-0.998319925	0.988202384	1.692793057
C	-1.888745106	-0.182678465	0.098048689
H	-2.544021485	0.295123279	-0.625604107
C	-0.743210227	-0.898191471	-0.507768158
C	-0.448352515	-0.778336708	-1.885534262
C	0.596486868	-1.489619694	-2.474526319
C	1.387385088	-2.352501194	-1.716124105
C	1.125180101	-2.488265188	-0.352817865
C	0.084531423	-1.771970575	0.235147019
H	0.791747242	-1.351390423	-3.533519196
H	2.190568229	-2.912043695	-2.188854608
H	1.708301166	-3.177130540	0.253971534
H	-0.115877405	-1.912934064	1.292420756
O	-1.213596348	0.082801498	-2.646599543
C	-1.952754250	-0.493013701	-3.743562866
C	-2.544838749	0.685185450	-4.502850346
C	-3.013796140	-1.464956562	-3.231863029
H	-1.256564653	-1.034493593	-4.401580798
H	-1.753358872	1.356101916	-4.848963069
H	-3.114956307	0.336495046	-5.369678917
H	-3.214255035	1.252658891	-3.848306104
H	-2.552138046	-2.264093737	-2.645368011
H	-3.730792982	-0.941461006	-2.591533390
H	-3.554378272	-1.918610903	-4.069450832
C	-0.220147566	2.754087361	1.192599152
N	0.805890190	3.141323168	0.362518991
C	1.193738448	4.551974390	0.491816149
C	-0.024520894	5.146611566	1.193375589
N	-0.627544593	3.959390808	1.836704913
H	2.109450279	4.650591597	1.094463185
H	1.388474158	4.997454621	-0.488627125
H	0.229046364	5.907160474	1.938485257
H	-0.723561643	5.598560260	0.476176765
C	1.500292036	2.325538388	-0.588618921
C	1.036834611	2.307204286	-1.915876962
C	-0.241980800	3.007264636	-2.297742292
C	1.775988744	1.604028722	-2.870185191
C	2.946028065	0.919971717	-2.536714408
C	3.689222021	0.103727689	-3.566180006
C	3.386282072	0.963120808	-1.211003330
C	2.686509438	1.665322816	-0.227257085
C	3.189817747	1.717065033	1.190454783
H	-0.248175944	4.054305453	-1.975093576
H	-1.091169298	2.505758202	-1.829126409
H	-0.393477363	2.981202724	-3.379736085
H	1.412352340	1.573056181	-3.894767164
H	3.477703289	0.445614018	-4.584126564
H	3.389249342	-0.950132177	-3.502067854
H	4.772858741	0.145281809	-3.412123116
H	4.301486599	0.442956801	-0.934590088
H	3.310184403	2.745094086	1.543725716
H	2.480688646	1.226452985	1.859690539
H	4.154795270	1.216074675	1.290730736
C	-1.964621178	4.170475419	2.342560070
C	-2.139799166	4.526660862	3.690234799
C	-0.982691958	4.628796729	4.655208103
C	-3.433863728	4.759448854	4.170759907
C	-4.555928910	4.652967618	3.350550486
C	-5.951153488	4.835691043	3.900260677
C	-4.352757648	4.342588020	2.003158072
C	-3.081050398	4.109549372	1.476439010
C	-2.946175326	3.734685955	0.023481544
H	-0.703929271	3.638954797	5.032836538
H	-0.094637718	5.074448498	4.196406504
H	-1.251673221	5.243561059	5.518979558
H	-3.563282699	5.022067622	5.218855286
H	-6.398624444	3.866694168	4.156566179
H	-5.950790174	5.445422198	4.809046178
H	-6.612309383	5.313210535	3.169478582
H	-5.212347691	4.272034813	1.339313815
H	-2.182728624	4.323986156	-0.491304954
H	-2.638419681	2.686188442	-0.057245275
H	-3.893564211	3.876324486	-0.504359385
S	0.121709273	0.284629096	3.872044903
C	1.426922172	1.333204230	4.164875470
N	1.482484562	2.667369637	3.918815753
C	2.725109009	3.182488410	4.257347728
C	3.469428142	2.154644888	4.730734035
N	2.662692164	1.014820780	4.682116572
H	0.742648359	3.142805060	3.403938581
H	2.966052675	4.225695292	4.132616228
H	4.493645745	2.094077167	5.063000870
C	3.132345934	-0.349014551	4.866289934
H	2.278808999	-0.967956550	5.142633738
H	3.895125074	-0.358430790	5.645580658
S	6.307469500	-0.100799602	4.291713955
C	5.068417322	-0.737619528	3.345730068
N	3.729415099	-0.876018176	3.654038816
C	3.028276428	-1.422317041	2.573343083
C	3.928813159	-1.655820285	1.592873233
N	5.164758600	-1.240378740	2.077663680
H	1.956919853	-1.534400654	2.596838874
H	3.785853171	-2.027517674	0.592039426
H	6.049567900	-1.282699935	1.596468452
C	-2.489042372	-0.733835812	1.301947061
C	-2.676184250	1.176018744	2.474120312
H	-2.105738353	-1.655344730	1.733491159
H	-3.568196235	-0.670259289	1.397521904
H	-3.617330431	1.395886673	1.957385526
H	-2.797316313	1.131903934	3.566309957

8b

100

RuC40N6H50S2O

Ru	-0.607385394	0.663762032	1.306045624
C	-1.872771135	-0.559792853	-0.099988800
H	-2.879284136	-0.164466099	-0.045971501
C	-1.359787559	-0.658295874	-1.478094275
C	-2.078941198	-0.078062174	-2.550538796
C	-1.661705793	-0.224814238	-3.873180227
C	-0.512084141	-0.957909183	-4.172533417
C	0.226815676	-1.522720512	-3.131285527
C	-0.188449610	-1.365569801	-1.811375080
H	-2.236841719	0.248974380	-4.663458278
H	-0.193948856	-1.073502754	-5.205089769
H	1.130699128	-2.086949661	-3.346422540
H	0.403975178	-1.804791911	-1.015292599
O	-3.186016755	0.688687837	-2.249263110
C	-4.453254038	0.284808222	-2.803973678
C	-5.426489210	1.398322965	-2.442018346
C	-4.875247133	-1.076975820	-2.256089411
H	-4.364576156	0.221654814	-3.899151090
H	-5.084067282	2.353568847	-2.850657133
H	-6.424057460	1.182644291	-2.837796463
H	-5.492950430	1.494761373	-1.353292138
H	-4.118630029	-1.834415982	-2.480397348
H	-4.997880698	-1.027443117	-1.169461458
H	-5.823820696	-1.392698354	-2.703471877
C	0.033473920	2.625439023	1.075350063
N	1.240141294	2.943358058	0.488305130
C	1.513323943	4.379624556	0.349688082
C	0.193392112	5.006215275	0.813045970
N	-0.537563029	3.852314817	1.371528596
H	2.363619278	4.674467730	0.979466212
H	1.762874220	4.630971726	-0.687544699
H	0.328056583	5.785267286	1.571832067
H	-0.374327054	5.447752181	-0.016475228
C	2.150316759	1.999710976	-0.077129750
C	1.872402585	1.480575120	-1.358049835
C	0.673385188	1.965274870	-2.134155106
C	2.764538231	0.560907828	-1.912789466
C	3.937982498	0.187892235	-1.248199878
C	4.874444024	-0.827552544	-1.860811443
C	4.216417293	0.767918733	-0.009331783
C	3.339116312	1.674518340	0.594926933
C	3.707792585	2.310078760	1.913486756
H	0.792385741	3.025492566	-2.394514290
H	-0.243563930	1.873924795	-1.550357352
H	0.545976856	1.398159546	-3.057187752
H	2.540404921	0.139077655	-2.888719236
H	4.972106409	-0.680869818	-2.941803421
H	4.503149716	-1.848652007	-1.706848799
H	5.875776288	-0.769951679	-1.421639564
H	5.136965243	0.509911538	0.508736149
H	2.836603015	2.733166291	2.412517882
H	4.167749553	1.581649769	2.587270123
H	4.435458931	3.118349387	1.765577109
C	-1.896678484	4.092733712	1.747552242
C	-2.179125901	4.527053421	3.051713557
C	-1.100529664	4.615815777	4.107140771
C	-3.506178737	4.803014070	3.398916597
C	-4.547801631	4.650749842	2.482716251
C	-5.983937028	4.895779895	2.883713417
C	-4.233381405	4.238906846	1.182742257
C	-2.922619543	3.963896278	0.788793523
C	-2.632548568	3.487640762	-0.609878084
H	-1.058235589	3.681938079	4.683587813
H	-0.111144990	4.782076951	3.673432500
H	-1.306597804	5.422431834	4.817690929
H	-3.729512745	5.129938933	4.412843012
H	-6.495232800	3.949416117	3.101272464
H	-6.052403430	5.519559989	3.780147000
H	-6.545593403	5.388471443	2.082862724
H	-5.032958384	4.127455663	0.452631134
H	-1.738235620	3.956553595	-1.032153775
H	-2.454264311	2.408390617	-0.607550313
H	-3.473599783	3.691215492	-1.278073144
S	1.150141800	-0.510755995	2.637801340
C	1.569501749	0.608895520	3.850657898
N	1.052056755	1.847421854	4.016978382
C	1.634326823	2.485357703	5.103294025
C	2.517168383	1.613679536	5.648963822
N	2.460805434	0.448051433	4.880514431
H	0.383894439	2.199251337	3.325226870
H	1.362049112	3.486666147	5.392070832
H	3.198057167	1.693267411	6.481861331
C	3.251577420	-0.750039257	5.091563105
H	2.674781321	-1.603119344	4.730975900
H	3.461513370	-0.846046131	6.157828106
S	5.931158399	0.369679170	6.511030157
C	5.687899852	-0.238853411	4.962387569
N	4.520958868	-0.713949366	4.395545281
C	4.735810281	-1.107532725	3.072764010
C	6.046722704	-0.902739235	2.808885290
N	6.613081203	-0.377993370	3.965262080
H	3.923492141	-1.447523647	2.449842845
H	6.617323209	-1.076870671	1.911380153
H	7.575542544	-0.110655939	4.101160412
C	-1.405552258	-1.386384975	0.953155132
C	-2.099110786	1.074914098	2.284106567
H	-0.650830203	-2.147711414	0.764692698
H	-2.082892199	-1.641560291	1.764884072
H	-2.979898095	0.411016281	2.299633942
H	-2.192512182	1.912619269	2.982861567

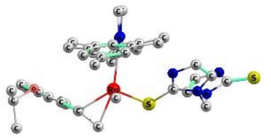

9b

100

RuC40N6H50S2O

Ru	-0.693321122	0.693632768	0.582784568
C	-2.296263631	-3.507965564	1.415213687
H	-3.383328198	-3.487366082	1.424310203
C	-1.650880506	-2.938208450	0.222488553
C	-2.296035416	-1.908896209	-0.506598790
C	-1.625107329	-1.271976775	-1.564194203
C	-0.348494941	-1.702506892	-1.941343453
C	0.269907249	-2.755263126	-1.272391021
C	-0.383926986	-3.361418486	-0.202727197
H	-2.100279896	-0.472678595	-2.115445130
H	0.155920272	-1.203951429	-2.761299487
H	1.255789658	-3.094542614	-1.576194872
H	0.091239274	-4.180081051	0.327723456
O	-3.550612890	-1.578645636	-0.084364240
C	-4.310067125	-0.562397247	-0.773100030
C	-5.398681561	-0.121976972	0.197626214
C	-4.887749220	-1.130503731	-2.069560318
H	-3.649456800	0.290276317	-0.966148499
H	-4.959698069	0.339621775	1.084249411
H	-6.055218470	0.609484907	-0.285025961
H	-6.003238882	-0.981304815	0.506185789
H	-4.103189101	-1.512548760	-2.727557458
H	-5.571657119	-1.954681464	-1.841710666
H	-5.443901476	-0.357322559	-2.610077499
C	-0.299496248	2.543681217	0.156302211
N	0.815056638	2.861895988	-0.622071227
C	1.135094571	4.291046313	-0.633211251
C	-0.207483221	4.900399291	-0.221357620
N	-0.849111896	3.776815128	0.472323023
H	1.927190210	4.533071336	0.091413651
H	1.469220255	4.615454945	-1.624461842
H	-0.101778378	5.766393307	0.440498519
H	-0.800123011	5.211092364	-1.095146866
C	1.791827221	1.890580447	-0.994482586
C	1.658216525	1.260713964	-2.246878430
C	0.478683737	1.590025456	-3.130369372
C	2.625369488	0.333809372	-2.639344389
C	3.719059085	0.028298271	-1.821502725
C	4.732686740	-1.006479725	-2.250851346
C	3.826290483	0.669348823	-0.586141437
C	2.870779536	1.590111628	-0.145536635
C	2.999177036	2.226887385	1.216538849
H	0.470100721	2.651761980	-3.401419683
H	-0.460020430	1.391382074	-2.602967198
H	0.498011004	1.001213278	-4.052295597
H	2.526999162	-0.158750594	-3.604959895
H	4.942563004	-0.943977847	-3.323888827
H	4.362967987	-2.020181951	-2.052089843
H	5.679666327	-0.890636897	-1.713971611
H	4.665908707	0.439134502	0.065394969
H	2.015249069	2.410427994	1.652529764
H	3.556824913	1.574973896	1.895237092
H	3.533828552	3.183866242	1.164982137
C	-2.111427248	3.982391158	1.097631373
C	-2.131649365	4.377650854	2.444090246
C	-0.839691840	4.573374436	3.202360765
C	-3.367246075	4.569791734	3.071173395
C	-4.570783951	4.374088989	2.388685020
C	-5.899244961	4.529111655	3.093006145
C	-4.518522614	3.995781888	1.042012523
C	-3.306040035	3.798773734	0.377912116
C	-3.268254833	3.355111940	-1.062423046
H	-1.020019549	4.622631161	4.280647161
H	-0.135190578	3.762724776	2.995359239
H	-0.337197819	5.504715932	2.911077811
H	-3.389849315	4.866719628	4.118078704
H	-6.284722760	3.555119442	3.420719408
H	-5.813176634	5.162728078	3.981212700
H	-6.654427551	4.970012562	2.433585884
H	-5.448372785	3.841078868	0.497670029
H	-2.758581939	4.086890093	-1.700245218
H	-2.713480370	2.413680408	-1.137243828
H	-4.278392058	3.210380815	-1.456656591
S	0.971255484	-0.940485358	1.649929537
C	1.132456504	-0.131097103	3.149135150
N	0.500331487	1.009736158	3.490328700
C	0.874537932	1.418980968	4.759178000
C	1.757625663	0.502463419	5.231331809
N	1.899441818	-0.467443669	4.233508847
H	-0.141985809	1.401729416	2.759358118
H	0.481479318	2.315786166	5.209636585
H	2.320554375	0.436602002	6.149499085
C	2.796184280	-1.605243185	4.274229623
H	2.379204713	-2.386841892	3.637297155
H	2.888465487	-1.949129160	5.305338638
S	5.159610430	-0.733732504	6.310719937
C	5.150374670	-0.861994772	4.633895780
N	4.125018127	-1.269999461	3.803354301
C	4.509664097	-1.191475525	2.463328784
C	5.792731216	-0.761948480	2.446269493
N	6.171586695	-0.573353395	3.770797038
H	3.816150920	-1.415433151	1.667486872
H	6.462424022	-0.581021982	1.621267880
H	7.065095794	-0.248392543	4.105828720
C	-1.639643636	-3.992303388	2.478171722
C	-2.234333900	0.916117172	1.546375738
H	-0.555187867	-3.966036355	2.542802031
H	-2.173482465	-4.406438813	3.328395053
H	-2.832499639	-0.009951065	1.597777502
H	-2.619850965	1.714604389	2.191123902

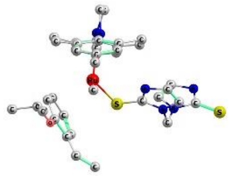

1c

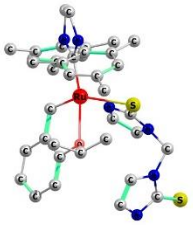

94

RuC38N6H46S2O

Ru	-0.534796323	1.542400239	0.923474035
C	-0.571168965	1.093029674	-0.904450910
H	-0.644339207	1.747254144	-1.779042938
C	-0.224809760	-0.278580209	-1.278839343
C	-0.044866854	-1.261995020	-0.277845708
C	0.259774929	-2.584299492	-0.582251203
C	0.397336494	-2.971056198	-1.919915824
C	0.238600328	-2.024995461	-2.935006208
C	-0.060860647	-0.702356842	-2.615064294
H	0.376680891	-3.317628160	0.209644038
H	0.624395466	-4.005993658	-2.160763631
H	0.346684335	-2.320734590	-3.975386589
H	-0.187741895	0.034217369	-3.405587000
O	-0.162825005	-0.801622664	1.030648715
C	-0.940638064	-1.620657852	1.962348333
C	-2.398943760	-1.673569426	1.520811246
C	-0.757296999	-1.020444700	3.346823422
H	-0.505996886	-2.627625812	1.955394457
H	-2.488101204	-2.091446430	0.514152126
H	-2.975882280	-2.299723153	2.210064832
H	-2.816356288	-0.663548192	1.520975009
H	0.302314483	-0.931707281	3.598505925
H	-1.212318860	-0.028110011	3.408682771
H	-1.245502057	-1.664739054	4.086249157
C	-1.275537936	3.330093371	0.776143995
N	-1.200016942	4.379332310	-0.114376305
C	-1.897957374	5.589248558	0.346922834
C	-2.789329637	5.044816972	1.474572512
N	-2.144221281	3.763235133	1.762108457
H	-1.180207384	6.333827689	0.718812457
H	-2.470403305	6.048857543	-0.465636036
H	-2.803168956	5.694276530	2.356606086
H	-3.827904041	4.902146883	1.142137002
C	-0.230036042	4.479993136	-1.155216520
C	-0.640696628	4.229385876	-2.477506796
C	-2.077905319	3.867232641	-2.766412136
C	0.308777822	4.303395913	-3.497803768
C	1.642897677	4.636174592	-3.233816339
C	2.648437147	4.708784595	-4.359901421
C	2.011944843	4.914490371	-1.914990542
C	1.091736020	4.850142709	-0.860642543
C	1.517235584	5.143927461	0.558384370
H	-2.748402030	4.709455819	-2.556261033
H	-2.403436378	3.033919513	-2.136612069
H	-2.210373059	3.587670775	-3.815377808
H	0.005151992	4.087928835	-4.520491451
H	2.514667552	5.623085995	-4.950915048
H	2.537760653	3.863165904	-5.047595215
H	3.676330585	4.707993987	-3.984543107
H	3.044043857	5.180424711	-1.695099068
H	1.093554917	6.091213675	0.913491652
H	1.182937274	4.357493119	1.241599387
H	2.605495709	5.219909092	0.631206083
C	-2.619807169	2.863428092	2.764331280
C	-2.010837634	2.897411534	4.031941207
C	-0.905109863	3.884792196	4.318047735
C	-2.455822212	2.003531204	5.012543529
C	-3.485859291	1.094501956	4.759730904
C	-3.904239956	0.085862932	5.803354094
C	-4.091447636	1.102259083	3.497408212
C	-3.677619039	1.972551181	2.486883182
C	-4.314561336	1.934301404	1.119398444
H	-0.483557708	3.723664956	5.313968870
H	-0.099071033	3.792833170	3.585282187
H	-1.274387527	4.916422678	4.270346654
H	-1.980781007	2.014152939	5.991294703
H	-3.377404596	-0.865158959	5.651952521
H	-3.671547197	0.430155765	6.815648337
H	-4.977158819	-0.126510330	5.752248383
H	-4.895875699	0.399650370	3.289160561
H	-4.765179696	2.897161100	0.853038103
H	-3.552338471	1.702867749	0.366295030
H	-5.098741041	1.173201372	1.073938332
S	1.504428588	1.645692657	2.564940326
C	2.670249608	1.753378322	1.320589724
N	2.435783691	2.159873683	0.057208583
C	3.584422265	2.066218290	-0.710660639
C	4.569602200	1.605247380	0.098482986
N	4.004760318	1.426879107	1.369866481
H	1.438666185	2.282790162	-0.218061781
H	3.593292949	2.361208086	-1.747013883
H	5.604396580	1.357832787	-0.084225039
C	4.545614793	0.492003117	2.346485002
H	3.966110651	0.587731921	3.264732910
H	5.602398739	0.696275505	2.522936993
S	7.132900699	-1.099570513	1.211743576
C	5.504746988	-1.526025689	1.239058600
N	4.443990915	-0.859685006	1.823533401
C	3.233428771	-1.493480447	1.512327416
C	3.539112095	-2.588546577	0.781445157
N	4.922279780	-2.609516634	0.642838542
H	2.279130041	-1.080737057	1.804874631
H	2.898420730	-3.329461669	0.333166452
H	5.469226422	-3.280686131	0.126762246

2c‡ (21.75*i* cm^{-1})

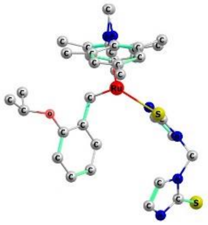

94

RuC38N6H46S2O

Ru	-0.096716288	1.699574265	1.381476914
C	-0.455225030	0.885260564	-0.229587914
H	-0.878473705	1.267619544	-1.164081284
C	-0.034828291	-0.544500718	-0.252722570
C	-0.931542927	-1.595919758	0.040859288
C	-0.500120518	-2.924271922	0.078456333
C	0.829723756	-3.244721054	-0.192424475
C	1.735222227	-2.225485078	-0.490771272
C	1.304162138	-0.900492899	-0.513130787
H	-1.219528524	-3.696204359	0.338088295
H	1.153666348	-4.281919038	-0.161071186
H	2.776711191	-2.456761002	-0.697111601
H	2.013278055	-0.108555639	-0.732079249
O	-2.246653718	-1.310111264	0.347135820
C	-3.230276732	-1.708444192	-0.630802911
C	-3.332904941	-0.643713738	-1.720641569
C	-4.536995747	-1.914235006	0.123593384
H	-2.914664260	-2.660997632	-1.081387555
H	-2.361355651	-0.491025605	-2.199695420
H	-4.057879340	-0.937578031	-2.487788677
H	-3.650194701	0.307042978	-1.281342997
H	-4.413492773	-2.672594279	0.902111235
H	-4.848013389	-0.980516220	0.601068501
H	-5.329162019	-2.235970221	-0.560320598
C	-1.247978033	3.215035899	1.066754216
N	-1.425962455	4.098259225	0.020368998
C	-2.252928171	5.261253339	0.381827280
C	-2.911428897	4.807749349	1.697478470
N	-2.075045032	3.666925419	2.069728363
H	-1.627800441	6.153678261	0.525253759
H	-2.982490481	5.485505367	-0.403580579
H	-2.896796011	5.586381953	2.468378177
H	-3.956868151	4.499258728	1.551023577
C	-0.572336424	4.166185200	-1.121541784
C	-1.063073517	3.694239879	-2.352606803
C	-2.468027333	3.148970221	-2.450442310
C	-0.228758308	3.742824944	-3.471755790
C	1.067262665	4.263981521	-3.397432262
C	1.968149955	4.259548155	-4.611234327
C	1.512736536	4.763773482	-2.170140422
C	0.712256388	4.728894144	-1.021863062
C	1.228164238	5.261731032	0.293016561
H	-3.209913289	3.954770968	-2.381981078
H	-2.676950165	2.455426983	-1.632014036
H	-2.626664291	2.629915791	-3.399832072
H	-0.596131914	3.360832094	-4.422250980
H	1.416408282	4.517758316	-5.521251471
H	2.405418654	3.265826609	-4.773950744
H	2.793702858	4.969593140	-4.503737588
H	2.511109135	5.191466788	-2.099821673
H	0.652348529	6.132205924	0.627940267
H	1.151160722	4.498160415	1.074630457
H	2.274600716	5.566450252	0.207456907
C	-2.256811941	2.920313963	3.273768743
C	-1.492143190	3.278951936	4.399627461
C	-0.489676520	4.406455141	4.309753679
C	-1.656470885	2.547584625	5.578846555
C	-2.559461323	1.482908305	5.657815949
C	-2.745619493	0.724137532	6.951566508
C	-3.301298748	1.147478011	4.520166793
C	-3.162810774	1.844277053	3.317184859
C	-3.895856954	1.396953108	2.077385643
H	0.018718483	4.556280533	5.266382247
H	0.269691217	4.190152197	3.550025534
H	-0.967571641	5.351932649	4.027645482
H	-1.061999343	2.810240337	6.451551484
H	-3.014900889	-0.320834000	6.768134448
H	-1.835084195	0.738456973	7.558887644
H	-3.548077685	1.166554730	7.555670728
H	-3.993146205	0.308699518	4.564171401
H	-4.248462643	2.241460822	1.477309535
H	-3.214175876	0.812423099	1.447433356
H	-4.757515593	0.774172814	2.336825292
S	2.190917849	1.535465161	2.574396776
C	3.139827015	1.844496259	1.175700027
N	2.669420633	2.312620754	0.002673910
C	3.678393060	2.368129381	-0.943605773
C	4.818166262	1.947662690	-0.337777022
N	4.480323616	1.642063638	0.984820881
H	1.624698134	2.397796257	-0.075166477
H	3.490658752	2.712998597	-1.946842196
H	5.826028219	1.804104540	-0.696233036
C	5.352380391	0.979848452	1.939272325
H	4.980432056	1.190461983	2.942517176
H	6.369715711	1.352449406	1.812002106
S	7.666862910	-0.442987181	0.171119994
C	6.321451416	-1.093648590	0.946498033
N	5.379656189	-0.452415756	1.727128314
C	4.392002306	-1.347031322	2.148144098
C	4.728414981	-2.562930210	1.662944871
N	5.904575471	-2.395513370	0.939014014
H	3.538434895	-1.011164428	2.715822184
H	4.231399170	-3.514859422	1.752221124
H	6.421241878	-3.108486391	0.448311776

3c′

94

RuC38N6H46S2O

Ru	-0.061506545	1.357920837	1.297044432
C	-0.532882561	0.709548685	-0.371471205
H	-0.913158685	1.154360034	-1.291232448
C	-0.517211499	-0.754709637	-0.283000992
C	-1.704181107	-1.495989919	-0.557172556
C	-1.762177172	-2.864927387	-0.311507921
C	-0.643874079	-3.531838580	0.214704722
C	0.527377462	-2.835711541	0.482611209
C	0.590071387	-1.460739618	0.224676105
H	-2.665984909	-3.428792625	-0.511638789
H	-0.704771654	-4.601007029	0.399844650
H	1.397925735	-3.354228842	0.875248252
H	1.514164497	-0.913932527	0.396942560
O	-2.745367493	-0.733124353	-1.016388296
C	-4.033224571	-1.317546484	-1.250228866
C	-4.059396287	-2.037967667	-2.599545050
C	-5.022974224	-0.159097363	-1.191241336
H	-4.261344441	-2.023643702	-0.438533369
H	-3.294550608	-2.816830904	-2.650821620
H	-5.037174093	-2.500916626	-2.770746236
H	-3.867695716	-1.320630507	-3.404087533
H	-4.988162804	0.323872373	-0.211303472
H	-4.770108717	0.587405014	-1.951470816
H	-6.042051483	-0.514328553	-1.375345648
C	-1.022474252	3.028037710	1.154920399
N	-1.209646934	3.971955873	0.171451345
C	-1.912881343	5.171610145	0.653757640
C	-2.537627645	4.678412649	1.969791051
N	-1.735896915	3.484470260	2.241624372
H	-1.204932620	5.995298169	0.822878217
H	-2.658337706	5.510452103	-0.073026334
H	-2.455975094	5.410745430	2.780189285
H	-3.600492256	4.423078645	1.848473068
C	-0.455989968	4.030143235	-1.038637455
C	-1.085526232	3.643988250	-2.235921017
C	-2.518436970	3.164356986	-2.220886895
C	-0.354332568	3.704312806	-3.424339327
C	0.971703478	4.151392654	-3.447309463
C	1.755543916	4.161292351	-4.739799078
C	1.558511146	4.557125980	-2.245471226
C	0.866013880	4.503849655	-1.029799699
C	1.529281907	4.930352782	0.258105087
H	-3.203480755	3.968228941	-1.925186045
H	-2.654108147	2.348254143	-1.504884021
H	-2.823178605	2.812029922	-3.210546173
H	-0.827164528	3.390408140	-4.352958524
H	1.144584697	4.515236702	-5.577062556
H	2.099064737	3.151435179	-4.999082784
H	2.639266015	4.802933738	-4.671037276
H	2.584786019	4.919155324	-2.248649448
H	1.109230347	5.871268144	0.632709917
H	1.387558807	4.173580140	1.035876210
H	2.602737914	5.080256089	0.111957057
C	-1.911382301	2.663475218	3.398179397
C	-1.068062942	2.883124433	4.501598497
C	-0.006066930	3.955686319	4.445343612
C	-1.220556614	2.072070117	5.631266207
C	-2.187923829	1.065748979	5.682819616
C	-2.360848125	0.214113469	6.919218439
C	-3.013028169	0.871360640	4.568305676
C	-2.892476754	1.650710104	3.416672731
C	-3.744549308	1.376318147	2.201353175
H	0.547669851	4.008065570	5.387141906
H	0.706838580	3.748976319	3.640508994
H	-0.441312476	4.942703558	4.250903918
H	-0.565586531	2.228448778	6.485978070
H	-2.556761735	-0.831447047	6.658757640
H	-1.470984189	0.243530532	7.555147211
H	-3.209399676	0.561906462	7.522368626
H	-3.759930961	0.080158019	4.591938544
H	-4.260246210	2.276519717	1.849056828
H	-3.112459890	1.020416671	1.378924741
H	-4.501046089	0.616425418	2.418492318
S	2.227574012	1.429354017	2.522653029
C	3.167580713	1.845572456	1.149924307
N	2.697213087	1.981339062	-0.105661406
C	3.722791633	2.279723517	-0.984933611
C	4.869189124	2.333832960	-0.260021191
N	4.514895494	2.079582718	1.068970686
H	1.660823497	1.880556505	-0.234384947
H	3.540217522	2.434698275	-2.035005746
H	5.900734730	2.494115891	-0.533871857
C	5.439289333	1.954121069	2.179836178
H	4.891847388	2.165955686	3.099408490
H	6.259945188	2.659467812	2.041542737
S	8.308311474	1.248512351	0.849043982
C	7.214108443	0.274735073	1.677843222
N	6.013013835	0.627222249	2.260577183
C	5.383765506	-0.489517863	2.815421852
C	6.202528324	-1.546120465	2.610214342
N	7.313208776	-1.066246880	1.924809860
H	4.409697103	-0.410066681	3.273057898
H	6.096416869	-2.582676131	2.885934358
H	8.108271115	-1.600212704	1.610301313

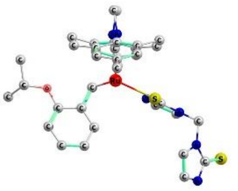

3c

94

RuC38N6H46S2O

Ru	-0.239238584	1.330383853	1.352630625
C	-0.663911142	0.645239369	-0.324570267
H	-0.818203926	1.150705887	-1.282131989
C	-0.443543362	-0.810636067	-0.453217949
C	0.381147259	-1.336552054	-1.485205199
C	0.580657002	-2.713793354	-1.609346133
C	-0.016920299	-3.591232839	-0.697503760
C	-0.810974175	-3.101329947	0.334885174
C	-1.021031273	-1.725277852	0.444687893
H	1.200435963	-3.119235426	-2.400362943
H	0.147702919	-4.660243536	-0.805985880
H	-1.278622509	-3.782268010	1.040587380
H	-1.667542232	-1.322871668	1.220391960
O	0.942118121	-0.394789409	-2.308530156
C	1.698854819	-0.794214172	-3.460560079
C	3.133878695	-1.154320418	-3.068692472
C	1.632153996	0.389217488	-4.419143222
H	1.208375757	-1.658213943	-3.931227714
H	3.154648257	-1.959105039	-2.329681396
H	3.701591541	-1.477410705	-3.948090720
H	3.634359220	-0.285644154	-2.630696912
H	0.593491276	0.614349792	-4.676078130
H	2.057271237	1.281210796	-3.950306709
H	2.189262947	0.175291839	-5.336851145
C	-1.147203528	3.019053031	1.171641708
N	-1.309655848	3.932282230	0.149819604
C	-1.943906381	5.184647337	0.592924704
C	-2.546265719	4.788231605	1.949714507
N	-1.795917533	3.571027123	2.257283311
H	-1.197053506	5.983915781	0.697823237
H	-2.697310746	5.519720284	-0.127583413
H	-2.401504148	5.553774294	2.719804413
H	-3.623844204	4.581537418	1.874463370
C	-0.554163583	3.936096991	-1.061627866
C	-1.193400621	3.537067111	-2.248664499
C	-2.628098812	3.068806742	-2.211175586
C	-0.470203675	3.568005557	-3.443725580
C	0.858360764	4.003787830	-3.484674727
C	1.612219356	4.060973212	-4.794012808
C	1.462329811	4.408308842	-2.290241618
C	0.778384297	4.383156154	-1.069197674
C	1.464455076	4.802844131	0.208898836
H	-3.307115665	3.890973084	-1.954003659
H	-2.762022775	2.293162192	-1.451399506
H	-2.937117382	2.669001490	-3.180982690
H	-0.952708659	3.239892572	-4.362224915
H	1.443864864	5.016649891	-5.306639599
H	1.292293422	3.265337390	-5.474133935
H	2.692290585	3.961108764	-4.642801982
H	2.496710185	4.746419225	-2.304294604
H	1.065499548	5.750622020	0.590013847
H	1.319962722	4.048870734	0.988624470
H	2.538310757	4.934870088	0.048594010
C	-1.917670877	2.875484022	3.498681182
C	-0.977328050	3.150398819	4.508548832
C	0.137522029	4.139228676	4.263036835
C	-1.091991454	2.477192038	5.728644305
C	-2.115677504	1.554738864	5.961478963
C	-2.249265016	0.867686128	7.301183623
C	-3.034560016	1.300841290	4.936522438
C	-2.955609304	1.945462490	3.700091388
C	-3.925295986	1.630889531	2.588007299
H	0.746819602	4.273734830	5.161377500
H	0.790607750	3.790265793	3.456763710
H	-0.252614840	5.119264382	3.965543037
H	-0.365522452	2.678667057	6.513243618
H	-2.634586912	-0.151290252	7.192444550
H	-1.288277543	0.813310515	7.821994045
H	-2.945486016	1.410129444	7.954123328
H	-3.828548689	0.575309679	5.101982836
H	-4.496736387	2.516003123	2.284783285
H	-3.374747184	1.275980322	1.709370777
H	-4.638217384	0.860377027	2.894538730
S	2.078850551	1.315862359	2.463197318
C	2.987246437	1.655307426	1.044952424
N	2.489572763	1.784943553	-0.199854429
C	3.498952164	2.033609443	-1.110053150
C	4.666412004	2.060119135	-0.417608028
N	4.339553783	1.838822504	0.923419538
H	1.457077318	1.697721073	-0.319228367
H	3.290294305	2.187829067	-2.154228469
H	5.694558510	2.182013490	-0.721789731
C	5.291087986	1.684168742	2.007792254
H	4.782693009	1.931934972	2.940642011
H	6.139085800	2.348284344	1.834875304
S	8.075055513	0.824715607	0.589238095
C	6.962083107	-0.085910030	1.464269286
N	5.802550016	0.332029788	2.086452381
C	5.135013582	-0.747768211	2.669217758
C	5.889908610	-1.846571085	2.443072331
N	6.999894665	-1.428754764	1.716890288
H	4.181718427	-0.616230887	3.157397330
H	5.738437496	-2.874883307	2.728188483
H	7.754898857	-2.005018047	1.379383757

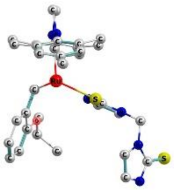

4c*cis*

100

RuC40N6H50S2O

Ru	0.039969547	0.959730093	1.626362510
C	-0.361543205	0.359004968	-0.100596020
H	-0.785675613	1.039486858	-0.837105772
C	-0.260878121	-0.997370602	-0.665924262
C	-1.404782440	-1.566639592	-1.286320102
C	-1.359015487	-2.848181646	-1.839555405
C	-0.169126458	-3.583153667	-1.798698570
C	0.968354399	-3.042635202	-1.206976709
C	0.911210183	-1.766004393	-0.643370069
H	-2.235970006	-3.286162155	-2.302105350
H	-0.143972441	-4.577800138	-2.235902525
H	1.896116187	-3.607771008	-1.181804065
H	1.792914438	-1.334742224	-0.179323819
O	-2.525742739	-0.774912567	-1.251075369
C	-3.736180820	-1.187276062	-1.900431098
C	-3.626939519	-0.994843456	-3.414167539
C	-4.838839290	-0.336088941	-1.281036227
H	-3.931737096	-2.243431476	-1.665812503
H	-2.786720481	-1.558758518	-3.827207418
H	-4.544348316	-1.326205977	-3.912329933
H	-3.470212465	0.064637663	-3.640501156
H	-4.879239527	-0.492128470	-0.199819827
H	-4.645311359	0.725418337	-1.466378827
H	-5.810787190	-0.594024752	-1.713684257
C	-0.914954871	2.737042273	1.161480253
N	-1.025304206	3.584961809	0.091013838
C	-1.796921094	4.808024528	0.393417187
C	-2.501321028	4.435776923	1.704819121
N	-1.724232283	3.264203215	2.127768218
H	-1.115024987	5.658778917	0.520671901
H	-2.491199170	5.048152541	-0.417535440
H	-2.460785181	5.231664062	2.455448048
H	-3.555459310	4.167206013	1.550333025
C	-0.195255155	3.576621748	-1.074811912
C	-0.746121715	3.133336679	-2.293782523
C	-2.182424738	2.668299397	-2.375051422
C	0.069831777	3.118533523	-3.426452976
C	1.401199432	3.547793527	-3.379914447
C	2.279524588	3.461997482	-4.606672110
C	1.899483022	4.034509793	-2.170370919
C	1.118114982	4.068288878	-1.009253133
C	1.674759346	4.654187106	0.264998748
H	-2.875584252	3.429357654	-1.999983471
H	-2.344909319	1.763631674	-1.780193646
H	-2.457832888	2.446233063	-3.410266510
H	-0.340782219	2.758161189	-4.367505250
H	1.730431657	3.735108660	-5.514034582
H	2.651058243	2.439332676	-4.753016810
H	3.150429669	4.119284111	-4.525956281
H	2.926598127	4.389016651	-2.120581767
H	1.310690670	5.678740197	0.414744710
H	1.392286781	4.067796183	1.140552551
H	2.767012006	4.698773325	0.227339876
C	-2.022495904	2.553955105	3.332283414
C	-1.308448705	2.883061380	4.496110727
C	-0.263599444	3.973318071	4.469656384
C	-1.591496904	2.179500822	5.672991787
C	-2.570698396	1.185188916	5.712371029
C	-2.873160791	0.433851739	6.987989754
C	-3.280381628	0.896557517	4.538934238
C	-3.022649188	1.559997719	3.338953681
C	-3.730815167	1.163809478	2.067030015
H	0.267900587	4.028740544	5.423671549
H	0.469560804	3.800512443	3.677197122
H	-0.718561618	4.954198356	4.282809737
H	-1.032164043	2.414899190	6.576018743
H	-2.937910314	-0.644431392	6.805459986
H	-2.102944920	0.601539506	7.746407139
H	-3.834347233	0.747287000	7.414257784
H	-4.038913519	0.116310785	4.555482290
H	-4.159489178	2.025622459	1.544458937
H	-3.015815719	0.692547421	1.381971647
H	-4.540928206	0.458707760	2.274671725
S	2.342290316	1.936499136	2.638225646
C	3.197209227	1.847654160	1.166195911
N	2.712322244	1.392172764	-0.009883967
C	3.677196832	1.449977942	-1.000162855
C	4.800967019	1.956987322	-0.435721861
N	4.492817306	2.220816862	0.903445380
H	1.718015002	1.081026267	-0.050551809
H	3.471941546	1.138596615	-2.011002100
H	5.793019518	2.139310556	-0.819454472
C	5.442908315	2.651666363	1.908897307
H	4.882984416	3.098439502	2.731820640
H	6.133710255	3.371791999	1.467937373
S	8.397080361	1.989686409	0.746073260
C	7.468481820	1.194977298	1.903683829
N	6.230598034	1.542418368	2.406814492
C	5.777368932	0.583377178	3.315324089
C	6.746382965	-0.354726081	3.415234844
N	7.771156270	0.034280715	2.559568733
H	4.802908793	0.665098468	3.772010980
H	6.799312654	-1.251704844	4.010492990
H	8.636574338	-0.453810380	2.389388216
C	-0.675013575	-0.945004446	2.497133456
C	0.717318612	-1.003543359	2.617261864
H	-1.287837359	-0.645490762	3.347651921
H	-1.194605534	-1.545614550	1.755639830
H	1.199518538	-0.735821665	3.554755558
H	1.306423862	-1.634262026	1.959863670

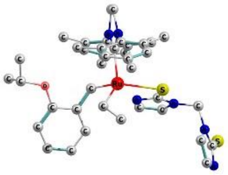

4c*trans*

100

RuC40N6H50S2O

Ru	-0.045901584	1.054860614	1.533403454
C	-0.873055775	0.425444588	-0.028986603
H	-1.017752771	1.125585600	-0.860226855
C	-1.183419488	-0.948859201	-0.467960387
C	-0.640239965	-1.473869213	-1.669387499
C	-0.957657808	-2.769474914	-2.091420667
C	-1.841959809	-3.552463389	-1.341451282
C	-2.403612385	-3.049903143	-0.170663485
C	-2.063799931	-1.764907588	0.256150591
H	-0.527094162	-3.182700012	-2.996155750
H	-2.082719752	-4.555598900	-1.683338544
H	-3.096339550	-3.652481461	0.410096052
H	-2.496480409	-1.352219983	1.161871315
O	0.197717691	-0.622246930	-2.342893889
C	0.835953774	-1.044024675	-3.556632937
C	2.064520085	-1.896578391	-3.236461607
C	1.183393011	0.234242509	-4.308050131
H	0.124328356	-1.621224200	-4.163431364
H	1.792487310	-2.774201709	-2.644320336
H	2.551993272	-2.235974089	-4.156679144
H	2.784455462	-1.309345485	-2.656667711
H	0.278199683	0.805253313	-4.528033508
H	1.830508918	0.873726785	-3.700854379
H	1.697257649	0.001488598	-5.246369297
C	-0.891125234	2.939613650	1.233514334
N	-0.930605766	3.843220793	0.217580159
C	-1.606601514	5.106365132	0.580334684
C	-2.307338744	4.735909320	1.895328718
N	-1.641169372	3.469749573	2.238877263
H	-0.861803196	5.901762374	0.714491827
H	-2.300945627	5.417964043	-0.205508415
H	-2.164586741	5.482361876	2.682699011
H	-3.386557432	4.579811268	1.765510436
C	-0.222329177	3.769699100	-1.021909573
C	-0.942052599	3.438555463	-2.186000901
C	-2.418604278	3.122293818	-2.122709372
C	-0.262547068	3.420461115	-3.405578868
C	1.101615983	3.718258683	-3.492087680
C	1.809121488	3.707381391	-4.827850021
C	1.784311893	4.051656860	-2.319814536
C	1.139352687	4.104808610	-1.078743365
C	1.882758746	4.562823601	0.151007373
H	-3.018346719	4.036499031	-2.021673680
H	-2.654187961	2.483488974	-1.267463683
H	-2.747734320	2.613632830	-3.033130979
H	-0.808998720	3.157525094	-4.309213055
H	1.687029786	4.666711326	-5.346895959
H	1.409779926	2.929057146	-5.485845226
H	2.883612231	3.533658436	-4.711944336
H	2.845933846	4.284286750	-2.368017514
H	1.686401512	5.625428242	0.345755131
H	1.589154276	4.002425098	1.039702630
H	2.962240626	4.448987726	0.016943469
C	-1.980980045	2.734202365	3.419191047
C	-1.241682732	2.963185256	4.589355037
C	-0.131700009	3.986372811	4.605448538
C	-1.556412129	2.216038655	5.730177040
C	-2.588982878	1.275830955	5.727563764
C	-2.928187023	0.481484117	6.967400325
C	-3.326848269	1.094362571	4.551225107
C	-3.043928733	1.809487581	3.387987080
C	-3.811874021	1.553972787	2.114978976
H	0.413777056	3.957493764	5.552604078
H	0.580212530	3.811142413	3.794080347
H	-0.526210389	5.002602966	4.478865859
H	-0.977751979	2.371207715	6.638305156
H	-3.111436202	-0.571034279	6.725615921
H	-2.122058730	0.523020467	7.705580231
H	-3.837645655	0.865585097	7.446578379
H	-4.133851341	0.364225323	4.536655376
H	-4.209835653	2.478014125	1.680827312
H	-3.143665692	1.103621282	1.372232351
H	-4.651819811	0.876445818	2.292123195
S	2.412468953	1.922060257	2.762763197
C	3.195838121	1.774024994	1.251017189
N	2.638631844	1.320907222	0.107711085
C	3.545745506	1.356359583	-0.934310251
C	4.711987809	1.839989430	-0.436848269
N	4.485480345	2.112115835	0.915762620
H	1.619054028	1.042384402	0.132544352
H	3.277649589	1.043294493	-1.929187568
H	5.685646535	1.996441458	-0.875378303
C	5.498619097	2.520684315	1.866135535
H	4.994500597	2.984279088	2.715437390
H	6.186075235	3.218942056	1.386829277
S	8.364699423	1.752556684	0.550303372
C	7.471465140	0.995057559	1.759893838
N	6.276449062	1.388329184	2.327127232
C	5.838068731	0.448928870	3.262752415
C	6.776145428	-0.523718816	3.314808938
N	7.766987827	-0.174254038	2.403857627
H	4.892628569	0.567601971	3.769817953
H	6.828029259	-1.420736383	3.910043744
H	8.603501228	-0.693999418	2.189409516
C	-0.032103125	-0.764497177	2.764288320
C	0.967695516	-0.981394162	1.817046779
H	0.227052240	-0.400083447	3.760149677
H	-0.981662388	-1.287078225	2.705115992
H	2.010599107	-0.801309431	2.062861229
H	0.789827110	-1.642020305	0.974194398

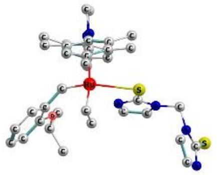

5c‡$_{cis}$ (298.83i cm^{-1})

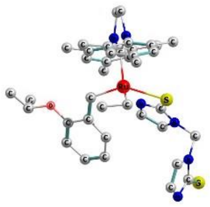

100

RuC40N6H50S2O

Ru	-0.006183111	1.352508980	1.919349675
C	-0.920889484	-0.024035873	0.880679886
H	-1.985065952	-0.175773802	0.687565935
C	-0.048601727	-0.930711332	0.122636818
C	-0.468820737	-1.489705874	-1.119332733
C	0.401183667	-2.266171196	-1.889018956
C	1.701809073	-2.532471712	-1.440953332
C	2.122331061	-2.040516984	-0.207620215
C	1.249283594	-1.274616979	0.562921570
H	0.080017269	-2.671131497	-2.841486027
H	2.363852954	-3.138148324	-2.053508778
H	3.120308207	-2.256565176	0.165006129
H	1.576868534	-0.913175576	1.534221010
O	-1.758264425	-1.193335752	-1.481313973
C	-2.359692143	-1.802407092	-2.631928281
C	-1.973096021	-1.048386203	-3.905622587
C	-3.862623732	-1.767908823	-2.375596322
H	-2.031228071	-2.849399170	-2.696338366
H	-0.888104958	-0.976564159	-4.013291698
H	-2.378094151	-1.553296470	-4.789023071
H	-2.377596667	-0.032876623	-3.870134746
H	-4.108437589	-2.316981019	-1.462450078
H	-4.199477689	-0.733019527	-2.256771497
H	-4.405262737	-2.216464193	-3.214008270
C	-1.085028860	2.944744116	1.575004977
N	-1.378713788	3.635157861	0.414132909
C	-2.116360837	4.886625428	0.644165561
C	-2.598372773	4.718880156	2.091151344
N	-1.736661433	3.635605087	2.578828955
H	-1.454221233	5.755438995	0.532506090
H	-2.937506742	4.996193478	-0.072673410
H	-2.470650336	5.625828317	2.692289092
H	-3.657367494	4.429833447	2.137778955
C	-0.702524051	3.459412659	-0.831396450
C	-1.340024041	2.733330513	-1.853886403
C	-2.671315900	2.067567425	-1.615104160
C	-0.693566122	2.607080948	-3.087237128
C	0.555000086	3.187661097	-3.326627568
C	1.256687991	2.989101200	-4.650922973
C	1.153104870	3.925748777	-2.300412709
C	0.545119641	4.076425414	-1.050161072
C	1.237412588	4.837295598	0.054430701
H	-3.295326177	2.640598142	-0.924766930
H	-2.513973140	1.078383665	-1.173874115
H	-3.216589815	1.935783220	-2.555221522
H	-1.177025989	2.041388459	-3.880504752
H	0.546063857	2.969377096	-5.483721886
H	1.800097269	2.034718739	-4.672088901
H	1.985121150	3.782430933	-4.844201945
H	2.125500788	4.383715627	-2.469902328
H	0.725323279	5.779190668	0.285503372
H	1.264387963	4.244150450	0.973932710
H	2.265299934	5.082310959	-0.227793213
C	-1.954219847	3.086250919	3.878520584
C	-1.122400952	3.500654576	4.930158567
C	0.013874663	4.457319371	4.663197844
C	-1.343530066	2.971305056	6.205300179
C	-2.364372356	2.047418151	6.449083611
C	-2.605194573	1.502597687	7.838104524
C	-3.170736509	1.647315010	5.377814679
C	-2.986735240	2.153839275	4.088609733
C	-3.852362866	1.683630989	2.942587264
H	0.567206213	4.678211312	5.580456361
H	0.708997193	4.031079304	3.932184803
H	-0.346882347	5.404274937	4.245401588
H	-0.695753817	3.278273878	7.023913462
H	-2.948830599	0.463442146	7.805767585
H	-1.696559766	1.539768558	8.446911132
H	-3.375163112	2.082962793	8.362899535
H	-3.956852478	0.914346290	5.549137355
H	-4.567009382	2.455128220	2.629513756
H	-3.237942244	1.430542019	2.073465587
H	-4.431671069	0.800670646	3.228531492
S	2.415012978	2.073505519	2.495906187
C	3.060393627	1.844812825	0.927889035
N	2.353399544	1.663403312	-0.205778052
C	3.184790353	1.429620157	-1.285664390
C	4.456905477	1.472286397	-0.820444881
N	4.377867100	1.747901275	0.549884435
H	1.325849192	1.651921172	-0.154396380
H	2.792627814	1.258348411	-2.273934635
H	5.411699730	1.308472759	-1.295602889
C	5.482227810	1.610417657	1.480241302
H	5.221938546	2.134636843	2.400231415
H	6.388059871	2.023808391	1.035054219
S	7.786293935	-0.083434669	-0.061286793
C	6.657928611	-0.558947974	1.093060325
N	5.737257922	0.212671597	1.775179698
C	4.943339936	-0.583308786	2.605164472
C	5.387265112	-1.854669684	2.477493877
N	6.435510900	-1.823329258	1.564010721
H	4.135446664	-0.155557482	3.178626655
H	5.061138109	-2.765549891	2.952793816
H	6.979099622	-2.607313217	1.238153424
C	-0.915741663	-0.548316292	2.924450231
C	-0.005612068	0.219801445	3.737666518
H	-1.978976656	-0.433828068	3.114591934
H	-0.622298400	-1.561191761	2.650147602
H	-0.426117251	0.829932543	4.532685822
H	0.966413725	-0.217737293	3.979300639

5c‡$_{trans}$ (255.37i cm^{-1})

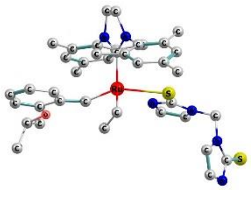

100

RuC40N6H50S2O

Ru	0.391733367	0.981425426	0.985252678
C	0.080418013	-0.152118628	-0.530397452
H	0.924298937	-0.082234496	-1.233339762
C	-1.027959396	-0.939361899	-1.094091211
C	-1.017728394	-1.370957521	-2.449117468
C	-2.143280187	-1.993706440	-3.005234015
C	-3.274384974	-2.232800697	-2.223396533
C	-3.290942781	-1.857447327	-0.880103893
C	-2.180860263	-1.214791189	-0.340987518
H	-2.148375838	-2.299337219	-4.044850622
H	-4.137753199	-2.716948540	-2.672630564
H	-4.164784903	-2.050545109	-0.263887092
H	-2.183246303	-0.889109781	0.691221171
O	0.121552888	-1.100387903	-3.155289935
C	0.287821358	-1.574509976	-4.499748979
C	0.567259496	-3.078115418	-4.516511716
C	1.444443943	-0.763808204	-5.070991424
H	-0.620123069	-1.350044040	-5.078799475
H	-0.242763564	-3.644271672	-4.050552490
H	0.688720454	-3.432440158	-5.545631591
H	1.489553899	-3.286400740	-3.964937345
H	1.215326789	0.302142389	-5.023489540
H	2.354104712	-0.946270812	-4.489336505
H	1.633023278	-1.043530243	-6.112514311
C	-0.846100298	2.529285900	0.979441791
N	-1.246310193	3.212799752	-0.153936773
C	-2.173184482	4.315169284	0.132973840
C	-2.669783981	3.940705167	1.535657876
N	-1.593345681	3.058025387	2.005218639
H	-1.646454221	5.280951404	0.131012359
H	-2.976914643	4.362101599	-0.608772223
H	-2.790107050	4.805211695	2.195732952
H	-3.626546011	3.399037488	1.498030712
C	-0.496938273	3.182125928	-1.368561106
C	-1.033227814	2.517889486	-2.482484674
C	-2.398418185	1.879948180	-2.413120969
C	-0.279491999	2.474283507	-3.661221709
C	0.977821900	3.074639601	-3.752565325
C	1.793937062	3.004837107	-5.023238176
C	1.480198145	3.742608523	-2.629074208
C	0.759523819	3.819989390	-1.434932585
C	1.298359199	4.608093991	-0.261616729
H	-3.186921735	2.629341278	-2.563288675
H	-2.561436088	1.410971672	-1.441766330
H	-2.516565693	1.113155907	-3.182930204
H	-0.688198701	1.954995077	-4.525562260
H	2.286308069	3.960425511	-5.234052287
H	1.174734566	2.745465710	-5.887291388
H	2.584161004	2.247295608	-4.946671984
H	2.449657257	4.234605672	-2.691958597
H	0.710167621	5.519827152	-0.099303698
H	1.261288591	4.027049791	0.663767936
H	2.335228825	4.908930763	-0.436766343
C	-1.572538572	2.557880899	3.343286316
C	-0.824484648	3.263691387	4.301713407
C	-0.124131669	4.547262657	3.926170569
C	-0.750693516	2.750867348	5.599036688
C	-1.411903493	1.572305672	5.962204544
C	-1.323549332	1.040249850	7.373759204
C	-2.184189123	0.919566993	4.997337841
C	-2.286891420	1.399728032	3.688092790
C	-3.172806672	0.710004577	2.681032601
H	0.478612711	4.923012975	4.757798127
H	0.534531388	4.391558290	3.068440222
H	-0.845705040	5.327760121	3.653139426
H	-0.155353014	3.278578565	6.341406957
H	-1.440860386	-0.047776597	7.399430670
H	-0.362344068	1.288566902	7.835246002
H	-2.109862253	1.468694871	8.008277068
H	-2.716983525	0.009346743	5.266454891
H	-4.178328927	1.151428264	2.678966439
H	-2.763368000	0.798901579	1.674275149
H	-3.286012588	-0.352919690	2.915737538
S	2.108920918	2.005394323	2.940331439
C	3.370925138	2.295429483	1.835424751
N	3.289000078	2.148111025	0.492049942
C	4.494491441	2.447282800	-0.121922232
C	5.361342037	2.797340983	0.859874474
N	4.658481997	2.720212207	2.067147706
H	2.388041135	1.813375896	0.104661787
H	4.621863426	2.389227997	-1.190658712
H	6.406514608	3.065192502	0.846099396
C	5.242078159	2.851101256	3.386986958
H	4.443672152	3.113492684	4.082797349
H	6.015156050	3.620240609	3.364570148
S	8.411356126	2.285174534	2.972874024
C	7.196253295	1.314138135	3.617159868
N	5.864449130	1.615329918	3.820165770
C	5.179165900	0.511463256	4.333416118
C	6.086141505	-0.479869920	4.487333064
N	7.308395501	0.024187437	4.056005710
H	4.114356447	0.549967288	4.505344550
H	5.973932794	-1.484783535	4.860585766
H	8.189919022	-0.463896501	4.029969599
C	0.863442915	-1.327718794	1.133566207
C	0.235421432	-0.696763410	2.269023246
H	0.359032444	-2.139341747	0.622917926
H	1.949988517	-1.401398190	1.102763679
H	-0.785221667	-0.988836748	2.514769123
H	0.856674325	-0.503128661	3.141514205

6c*cis*

100
RuC40N6H50S2O

Ru	0.211933874	1.451663852	1.340847184
C	-1.086728380	-0.263351676	1.174011573
H	-2.093679124	-0.253019236	0.765031809
C	-0.058504065	-0.658762871	0.228379012
C	-0.290103326	-0.702966072	-1.188814483
C	0.742789824	-0.877068526	-2.090205744
C	2.068309329	-1.057335449	-1.647466338
C	2.325618385	-1.084126172	-0.286525541
C	1.289770596	-0.894972616	0.645004332
H	0.506008225	-0.881115332	-3.151537095
H	2.869593376	-1.196355606	-2.367784167
H	3.334822268	-1.261074088	0.076854131
H	1.507813574	-1.049842152	1.693380415
O	-1.583914102	-0.540983469	-1.647987228
C	-2.218200007	-1.718415715	-2.190494070
C	-3.499302091	-1.231490738	-2.852800710
C	-2.476833007	-2.756159647	-1.099847875
H	-1.553859124	-2.154885171	-2.952271971
H	-3.276094157	-0.487223237	-3.623254916
H	-4.035144037	-2.065779119	-3.316279326
H	-4.153446464	-0.768977410	-2.106655587
H	-1.542533732	-3.045391882	-0.610736725
H	-3.147886672	-2.347268799	-0.337960353
H	-2.935857748	-3.653800555	-1.528399845
C	-0.983745452	2.961063253	1.603455470
N	-1.170033884	3.829658378	0.538297554
C	-1.833022775	5.081599495	0.901428816
C	-2.487441911	4.693544612	2.236098271
N	-1.700716176	3.516879410	2.636877686
H	-1.105647427	5.897223300	1.027773683
H	-2.559655001	5.383302646	0.139917601
H	-2.419685915	5.482186717	2.992106715
H	-3.547351617	4.430579229	2.110532442
C	-0.661283961	3.537225229	-0.760141170
C	-1.424777790	2.699940725	-1.600282355
C	-2.781405834	2.217127454	-1.157105902
C	-0.897007775	2.334971652	-2.839552289
C	0.344636002	2.807685022	-3.274075419
C	0.932180697	2.322221991	-4.577682870
C	1.043696885	3.703224585	-2.456749307
C	0.561560412	4.081566725	-1.194962461
C	1.327066432	5.053169548	-0.330707795
H	-3.390109178	3.047751166	-0.783398817
H	-2.672943941	1.492291136	-0.348948984
H	-3.310326273	1.730685019	-1.978620857
H	-1.459336365	1.647150026	-3.464802228
H	0.164389812	2.219946463	-5.351678177
H	1.387705350	1.332397428	-4.442614781
H	1.706358770	2.998366056	-4.953481810
H	1.991827462	4.113633109	-2.801004145
H	0.893838687	6.059159571	-0.396194504
H	1.310649968	4.741621314	0.716091382
H	2.371953481	5.123567788	-0.647307078
C	-1.964457464	2.890407219	3.890558539
C	-1.139246030	3.203495000	4.983919927
C	0.042920415	4.121271550	4.795815766
C	-1.389840041	2.581200849	6.207647975
C	-2.432309194	1.659674535	6.360664343
C	-2.677267201	0.997036794	7.696134143
C	-3.235853736	1.371419143	5.255079898
C	-3.021356499	1.975387468	4.011454149
C	-3.869825286	1.611699778	2.816661614
H	0.572391439	4.284055688	5.738981710
H	0.742984758	3.687400276	4.073589381
H	-0.258124056	5.096873381	4.397601383
H	-0.742117610	2.798764744	7.054765411
H	-3.273903453	0.086098278	7.589850259
H	-1.734200256	0.728406268	8.183368738
H	-3.214450642	1.666839586	8.379087832
H	-4.037847364	0.642750711	5.355381311
H	-4.547471951	2.427105249	2.534303606
H	-3.237984493	1.393310877	1.952278000
H	-4.484082335	0.731492085	3.027196352
S	2.414619966	2.443375721	1.994346810
C	3.458990624	2.064423290	0.698185938
N	3.168587974	2.081572354	-0.625939556
C	4.251203108	1.663028892	-1.383408263
C	5.256127095	1.386988665	-0.520234098
N	4.767654422	1.648838536	0.764845071
H	2.245561319	2.307650440	-0.981742251
H	4.200053105	1.589667416	-2.457071439
H	6.249584372	0.992638656	-0.664183505
C	5.435896094	1.259004899	1.994722468
H	5.049755201	1.874514797	2.806727430
H	6.510845566	1.398128299	1.873453210
S	7.464331139	-1.076429542	1.018105810
C	6.003612338	-1.166519811	1.850426932
N	5.200595178	-0.137539166	2.302975741
C	4.053741362	-0.640622083	2.923243746
C	4.143851614	-1.989076204	2.886841030
N	5.334933091	-2.294538226	2.236253803
H	3.280457014	0.016977868	3.287435052
H	3.470770611	-2.747723431	3.251346746
H	5.698882625	-3.213776513	2.039141699
C	-0.935366806	-0.620268025	2.657757458
C	0.061324407	0.434586233	3.162726941
H	-1.903081526	-0.514458047	3.159156932
H	-0.605788775	-1.662528102	2.815247673
H	-0.302430338	1.009795390	4.011050411
H	1.045815580	0.022799718	3.430978783

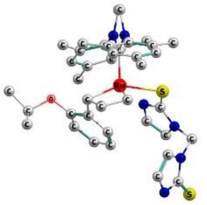

6c*trans*

100
RuC40N6H50S2O

Ru	0.874296350	1.568727436	1.162058613
C	0.585796523	-0.496114204	0.656886164
H	1.330123474	-0.537838415	-0.148747853
C	-0.618263178	-1.240589216	0.250673663
C	-0.857094272	-1.566123997	-1.105891096
C	-1.984788336	-2.282778179	-1.504505317
C	-2.907052571	-2.731479701	-0.558812784
C	-2.690731046	-2.437749440	0.788933047
C	-1.572788483	-1.704663242	1.179337184
H	-2.132783329	-2.480100004	-2.563210292
H	-3.782930585	-3.292336285	-0.873289864
H	-3.400505850	-2.774466458	1.540868556
H	-1.439616348	-1.463285745	2.229744842
O	0.027229353	-1.095432126	-2.067929984
C	0.770224569	-2.080426462	-2.808887591
C	1.806564857	-2.769539991	-1.921215120
C	1.400554966	-1.331060264	-3.976521057
H	0.074193279	-2.839662005	-3.196810402
H	1.325452804	-3.207707513	-1.041872026
H	2.314613999	-3.568091614	-2.473762552
H	2.556624947	-2.048901999	-1.578785196
H	0.627662536	-0.894533434	-4.616655986
H	2.030852914	-0.519511335	-3.596897423
H	2.017816926	-2.002676930	-4.582119480
C	-0.727601807	2.605920653	1.089112171
N	-0.912864533	3.518274254	0.045994010
C	-2.209481201	4.196941920	0.097147613
C	-2.588929306	3.996542127	1.568438304
N	-1.785615457	2.824434003	1.940609728
H	-2.118524509	5.252261429	-0.180708306
H	-2.937927814	3.724365552	-0.577649440
H	-2.309756328	4.865380081	2.182947712
H	-3.655775630	3.805483575	1.714939362
C	-0.176656708	3.395608717	-1.170515538
C	-0.487923519	2.376330818	-2.093943508
C	-1.671884668	1.471703439	-1.875213481
C	0.308344146	2.244438325	-3.234685160
C	1.368455385	3.115783513	-3.499278540
C	2.209586625	2.939138398	-4.742341134
C	1.609314186	4.165278953	-2.603483268
C	0.846043236	4.325574532	-1.437054646
C	1.108713313	5.466272039	-0.483322208
H	-2.609979454	2.027879916	-1.991870089
H	-1.654096402	1.044019613	-0.871841585
H	-1.668739677	0.647670096	-2.588133871
H	0.096566726	1.433161066	-3.925689801
H	3.125371579	3.537155723	-4.700235782
H	1.658185429	3.244127837	-5.640170737
H	2.493122795	1.889940939	-4.880995092
H	2.402265189	4.880525396	-2.817542326
H	0.246918377	6.141795365	-0.434810245
H	1.286683864	5.095069876	0.530031379
H	1.976087978	6.052824399	-0.800689362
C	-2.099181687	2.102570625	3.129433790
C	-1.478291699	2.417804661	4.347829483
C	-0.399172553	3.466782252	4.420106119
C	-1.833270322	1.680992716	5.481242034
C	-2.782511820	0.655806033	5.426842707
C	-3.155709563	-0.113723999	6.672742436
C	-3.394676847	0.377349229	4.200626763
C	-3.075009951	1.094232146	3.044542101
C	-3.748671751	0.793482548	1.728131379
H	-0.252249843	3.814363767	5.447095453
H	0.543023959	3.044884485	4.058294218
H	-0.620045525	4.331330201	3.787713122
H	-1.346282412	1.905670372	6.428375172
H	-3.587397932	-1.089046692	6.427592380
H	-2.283747779	-0.280326561	7.313349045
H	-3.896859601	0.433371657	7.269620183
H	-4.134587019	-0.418376574	4.140632173
H	-4.380351065	1.629102720	1.401622313
H	-3.011144641	0.611561919	0.943913881
H	-4.379387160	-0.095559655	1.800425340
S	2.540211016	3.187385001	2.142560034
C	3.819952530	3.107552550	1.013129276
N	3.739487915	3.167185235	-0.341428835
C	4.987120668	2.994883136	-0.923681557
C	5.876135766	2.831803443	0.084542761
N	5.154849041	2.917678201	1.278318903
H	2.862658686	3.291060678	-0.840417962
H	5.123881375	3.007540418	-1.992810850
H	6.936676680	2.633444749	0.092454051
C	5.680732406	2.551396779	2.585066833
H	5.039936659	2.996574541	3.345655804
H	6.705829992	2.913879388	2.671259264
S	8.337085780	0.797838536	1.965835244
C	6.794437965	0.323745445	2.446091833
N	5.703091843	1.113550574	2.753711307
C	4.590182230	0.323268503	3.057759674
C	4.987680814	-0.966412280	2.973877267
N	6.329875717	-0.951028720	2.610805136
H	3.631765003	0.761201449	3.286271785
H	4.438559402	-1.878844974	3.139565659
H	6.924052897	-1.750240748	2.453926614
C	1.176434908	-0.840374356	2.051009466
C	0.781351286	0.396802018	2.869850802
H	0.813278029	-1.798688069	2.458287508
H	2.268467192	-0.939381452	1.977804328
H	-0.223707724	0.297800604	3.279976775
H	1.471636114	0.688085026	3.675444257

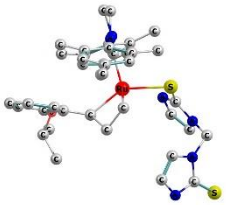

$\mathbf{7c}^{‡}$ (280.65*i* cm^{-1}) *from* $\mathbf{6c}_{cis}$

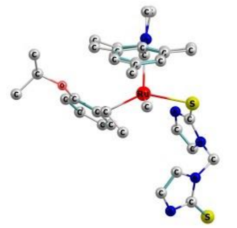

100
RuC40N6H50S2O

Ru	0.554010665	1.609428690	1.665204877
C	0.183704752	-0.466140072	1.159354318
H	-0.742226030	-0.930151782	1.488549686
C	0.425628879	-0.574692069	-0.296193483
C	-0.615377274	-0.988264088	-1.171458024
C	-0.390242773	-1.142113486	-2.541015070
C	0.866759628	-0.864588123	-3.089167865
C	1.897161292	-0.433147752	-2.259865590
C	1.675199860	-0.302996057	-0.888237093
H	-1.187848934	-1.478598527	-3.193206149
H	1.025668763	-0.992393840	-4.156607029
H	2.882141923	-0.221124367	-2.668672573
H	2.497462065	0.008332169	-0.252214944
O	-1.831894088	-1.194698638	-0.568959995
C	-2.933515869	-1.750311560	-1.299526414
C	-4.173697166	-1.421867004	-0.477732201
C	-2.741987285	-3.254577093	-1.497479388
H	-3.016784532	-1.248879357	-2.274422121
H	-4.282816147	-0.340184378	-0.364186440
H	-5.070748512	-1.818952024	-0.963180453
H	-4.089886858	-1.863500953	0.520061678
H	-1.815066741	-3.470490824	-2.034772962
H	-2.694578331	-3.750880268	-0.522883092
H	-3.577927425	-3.677579366	-2.064844321
C	-0.894235057	2.928362811	1.227298172
N	-1.138372682	3.684118494	0.101002070
C	-2.160689788	4.722787300	0.280303223
C	-2.798687085	4.313388580	1.617273150
N	-1.827484632	3.348019895	2.147432449
H	-1.698939441	5.718360449	0.333450407
H	-2.874105845	4.724224745	-0.551172883
H	-2.928207533	5.158912478	2.301383914
H	-3.781664426	3.840393715	1.479729678
C	-0.440778626	3.531002600	-1.130455084
C	-0.913124305	2.570217339	-2.046420363
C	-2.165757228	1.794993557	-1.729459632
C	-0.215435511	2.381909717	-3.237662064
C	0.924060874	3.134094863	-3.546309737
C	1.694645079	2.849997036	-4.814045462
C	1.332404304	4.125470822	-2.650442679
C	0.660209514	4.347652561	-1.437002223
C	1.111407957	5.429979920	-0.486073546
H	-3.021386949	2.471645893	-1.614299887
H	-2.060666856	1.241199720	-0.794350611
H	-2.396505846	1.084645043	-2.526151646
H	-0.550586311	1.614158591	-3.928906039
H	2.522633791	3.551439211	-4.954634103
H	1.048411271	2.910404756	-5.697261564
H	2.107869165	1.834071562	-4.789053093
H	2.201390901	4.736693605	-2.887480875
H	2.123854366	5.767407169	-0.726697853
H	0.450629729	6.303657352	-0.546563005
H	1.106508278	5.073772057	0.547420158
C	-2.079965987	2.710487443	3.399920102
C	-1.537697465	3.278405538	4.565027149
C	-0.655571262	4.497978474	4.465625646
C	-1.778168668	2.652158137	5.789831190
C	-2.542590120	1.482653606	5.873510042
C	-2.798889631	0.828719931	7.211838657
C	-3.067587300	0.942361974	4.695567472
C	-2.848032994	1.536963525	3.448216107
C	-3.349898483	0.890708949	2.181773553
H	-0.256489758	4.778058504	5.444494915
H	0.182800850	4.302164846	3.789296631
H	-1.197756903	5.361399590	4.062033501
H	-1.345902989	3.074009269	6.695079380
H	-3.103875553	-0.215896206	7.096717514
H	-1.904388986	0.851628659	7.843732168
H	-3.595473869	1.346272542	7.761468649
H	-3.651480665	0.025007718	4.744853409
H	-3.921923561	1.588510660	1.559829320
H	-2.503972346	0.544876356	1.578268687
H	-3.993019345	0.034525709	2.403212119
S	2.500510277	3.278965552	2.228946073
C	3.545187188	2.890073204	0.942950133
N	3.218185944	2.740945173	-0.366489276
C	4.318306099	2.370359761	-1.123726271
C	5.372003406	2.295743844	-0.276074992
N	4.894977319	2.635967840	0.992996397
H	2.277262577	2.868972571	-0.721399712
H	4.243477386	2.200806303	-2.185201312
H	6.403521632	2.013876069	-0.421735052
C	5.631264796	2.425983540	2.228824254
H	5.139358393	3.000444412	3.013927163
H	6.666555044	2.741436297	2.093371688
S	8.140256977	0.496156485	1.523762331
C	6.655642987	0.152275752	2.240802062
N	5.638504130	1.022338208	2.583139575
C	4.535400828	0.324247913	3.084890727
C	4.873907285	-0.985016735	3.099348333
N	6.168862221	-1.073066736	2.598926492
H	3.611356423	0.825556220	3.331149745
H	4.312059818	-1.850131699	3.410969340
H	6.708885460	-1.912718984	2.459883029
C	1.317487826	-0.542793956	2.075313277
C	0.568481599	1.051665573	3.440447483
H	1.189855611	-1.132442910	2.978892882
H	2.325407565	-0.639870689	1.682202932
H	-0.140530422	0.342991572	3.882629344
H	1.264235202	1.471310594	4.185002439

$\mathbf{7c}^{‡}$ (294.00*i* cm^{-1}) *from* $\mathbf{6c}_{trans}$

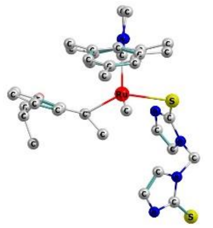

100
RuC40N6H50S2O

Ru	0.778987081	1.525308384	1.314326034
C	0.695180385	-0.431741158	0.384529441
H	1.070768882	-0.179327224	-0.609941855
C	-0.465409264	-1.340681610	0.322125343
C	-1.048803936	-1.678454786	-0.923175284
C	-2.185394912	-2.483337348	-1.009217564
C	-2.754912102	-3.027537318	0.140752485
C	-2.171255893	-2.750972414	1.379256409
C	-1.055556941	-1.923714234	1.462199986
H	-2.609766151	-2.680872820	-1.990043366
H	-3.637308524	-3.657070624	0.068887739
H	-2.592799297	-3.172891661	2.288167705
H	-0.636863510	-1.707976286	2.438408430
O	-0.487886462	-1.175106864	-2.088796650
C	0.135265741	-2.133982954	-2.965304278
C	1.380224649	-2.742420912	-2.321913353
C	0.447066793	-1.373340620	-4.247183585
H	-0.584604005	-2.937686698	-3.183618117
H	1.126907755	-3.225552213	-1.374049591
H	1.826227136	-3.491571946	-2.985376738
H	2.123250879	-1.964112387	-2.119852682
H	-0.467981377	-0.955242228	-4.677804691
H	1.134522206	-0.548834061	-4.029928093
H	0.913236195	-2.032396792	-4.986439355
C	-0.707491256	2.812925120	0.936245961
N	-0.813472133	3.750062115	-0.072528569
C	-2.086649495	4.487552031	-0.052092435
C	-2.596170944	4.187460965	1.367392600
N	-1.794731968	3.015047858	1.740900349
H	-1.928722251	5.555915418	-0.231047050
H	-2.772263382	4.110801700	-0.824168469
H	-2.408780620	5.024888646	2.054100328
H	-3.665476137	3.955017013	1.396691031
C	-0.019452059	3.680189653	-1.255758611
C	-0.276824508	2.677536151	-2.215111886
C	-1.418108519	1.712578162	-2.027675103
C	0.548642634	2.605594746	-3.340339749
C	1.592660493	3.513995406	-3.548778494
C	2.455728478	3.415256311	-4.785704360
C	1.794822224	4.526312059	-2.603879881
C	0.998860775	4.629617820	-1.453000847
C	1.244984097	5.708518198	-0.426649035
H	-2.374807023	2.242974662	-1.962103801
H	-1.289896991	1.147490900	-1.102309631
H	-1.471513527	0.993276439	-2.844360373
H	0.366700374	1.822353228	-4.072816044
H	3.387754602	3.978276987	-4.675088706
H	1.933045508	3.815263778	-5.663492334
H	2.710380819	2.373988012	-5.011227177
H	2.593408061	5.249735421	-2.757207962
H	0.383598658	6.381292608	-0.346266325
H	1.406993571	5.271461257	0.564135373
H	2.117975804	6.311501691	-0.691866312
C	-2.138723377	2.191053747	2.854972959
C	-1.704562672	2.534323914	4.143341994
C	-0.860620105	3.766515617	4.358461234
C	-2.035373738	1.684419346	5.204034689
C	-2.775664785	0.515148127	5.000743028
C	-3.134231420	-0.391512650	6.155237252
C	-3.198778381	0.206629231	3.703764700
C	-2.897757795	1.032294770	2.618750351
C	-3.334899338	0.673154727	1.221559054
H	-0.458219328	3.795768707	5.374919399
H	-0.024456440	3.784078192	3.653648239
H	-1.438857982	4.686196042	4.203710684
H	-1.690872165	1.931117168	6.206319989
H	-3.103527509	-1.445523978	5.858754127
H	-2.451725911	-0.256337088	6.999503818
H	-4.149848895	-0.188093515	6.517605221
H	-3.765667333	-0.705257913	3.528021431
H	-3.871039259	1.499792370	0.740882098
H	-2.462404182	0.445164864	0.604952519
H	-3.981480434	-0.207337422	1.221146702
S	2.500585646	3.273297119	2.278847747
C	3.723024494	3.039539483	1.117728576
N	3.583386370	3.061057672	-0.233422943
C	4.786223933	2.792914818	-0.868853438
C	5.712023711	2.607167440	0.102398943
N	5.055472255	2.776865839	1.323363634
H	2.699286768	3.254281698	-0.691149590
H	4.868999498	2.766072567	-1.943152832
H	6.759760494	2.349761726	0.069891124
C	5.634091551	2.461343806	2.618992364
H	5.005269400	2.919044792	3.383080303
H	6.655173114	2.842621817	2.662798274
S	8.334851967	0.747082397	2.071298066
C	6.800896141	0.255533285	2.563418092
N	5.688943813	1.030543437	2.827821046
C	4.589174233	0.229941471	3.145684173
C	5.015829230	-1.052775940	3.118958232
N	6.363749554	-1.021502550	2.776425828
H	3.610281518	0.652643802	3.307803414
H	4.484937181	-1.972550721	3.302203810
H	6.977382438	-1.812490796	2.658850731
C	1.743995342	-0.571151609	1.392083281
C	0.828269760	0.669156063	2.965822539
H	1.642855404	-1.361138833	2.132743470
H	2.779653721	-0.440812609	1.088155743
H	0.141323909	-0.120653845	3.281453639
H	1.484454784	1.007795341	3.784044327

8c
from **6c**$_{cis}$

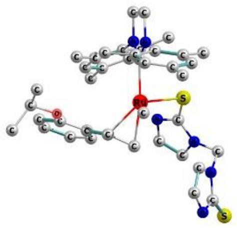

100
RuC40N6H50S2O

Ru	0.623790122	1.416361684	1.387153292
C	0.975275203	-0.527962525	0.358027082
H	0.304457312	-1.245246991	0.817930833
C	0.905254722	-0.562603969	-1.116985935
C	-0.256646322	-1.066679730	-1.762713618
C	-0.303742182	-1.212399233	-3.152543287
C	0.803041412	-0.870960409	-3.936664941
C	1.953305738	-0.376424130	-3.327969258
C	1.990841001	-0.226158900	-1.941163348
H	-1.191012976	-1.605222564	-3.634946298
H	0.757819112	-1.005035007	-5.014176465
H	2.830126376	-0.136029529	-3.924464770
H	2.906464935	0.116051341	-1.471772703
O	-1.292081260	-1.378511939	-0.926267551
C	-2.499588360	-1.966886229	-1.427198667
C	-3.547922623	-1.714964706	-0.350303255
C	-2.291482642	-3.455879324	-1.709005903
H	-2.805880496	-1.445589173	-2.345820542
H	-3.677515642	-0.642006891	-0.187184282
H	-4.509971597	-2.147659885	-0.643079520
H	-3.230797019	-2.168068450	0.594128576
H	-1.489242221	-3.616275818	-2.433910298
H	-2.019866726	-3.970591042	-0.781798058
H	-3.209197204	-3.906065211	-2.102561546
C	-0.929400897	2.852035677	1.325196730
N	-1.068298473	3.718758759	0.268188208
C	-2.208875551	4.642065536	0.369522912
C	-2.709970922	4.378428284	1.796588549
N	-1.894424807	3.223047959	2.212833543
H	-1.886734438	5.677558111	0.213899803
H	-2.963801376	4.406944640	-0.392403214
H	-2.536819749	5.228957360	2.467351777
H	-3.777273078	4.133245698	1.838879138
C	-0.362118391	3.601611987	-0.965319525
C	-0.742146853	2.595105628	-1.877846994
C	-1.911060703	1.693806007	-1.569008648
C	-0.019505360	2.462651650	-3.062420004
C	1.045598861	3.318196559	-3.371419218
C	1.818633989	3.129624577	-4.655415925
C	1.356633519	4.347873899	-2.478076726
C	0.654933646	4.518724093	-1.274848120
C	0.965710261	5.690379836	-0.376358727
H	-2.803049760	2.278407570	-1.316056338
H	-1.685015181	1.052473219	-0.712120388
H	-2.146344501	1.058407591	-2.425591918
H	-0.275979790	1.661066463	-3.748229389
H	2.712633025	3.760175935	-4.687476268
H	1.203530924	3.380451008	-5.528152637
H	2.125545153	2.084510496	-4.768379113
H	2.159638324	5.042287304	-2.719653470
H	2.034198868	5.923834595	-0.388639382
H	0.427218930	6.584581446	-0.718769007
H	0.680276377	5.487207911	0.655898105
C	-2.101661377	2.684792083	3.516996911
C	-1.389266383	3.205919846	4.608058767
C	-0.324882905	4.254965417	4.397771995
C	-1.627029914	2.662112264	5.873986989
C	-2.543511669	1.622834112	6.069239630
C	-2.796161122	1.067162145	7.451788880
C	-3.227152106	1.119441889	4.956982946
C	-3.017781405	1.633099392	3.674390838
C	-3.663373168	1.014493430	2.459652432
H	0.055126345	4.629473408	5.352504646
H	0.513810628	3.828742376	3.836752237
H	-0.691627933	5.109327832	3.818966770
H	-1.069768266	3.047013008	6.725828797
H	-3.141041572	0.029240378	7.409334757
H	-1.890843525	1.098428407	8.066784894
H	-3.566291821	1.646933641	7.976925966
H	-3.921545914	0.291746454	5.087008080
H	-4.223264148	1.749828232	1.870190575
H	-2.883018572	0.603926982	1.808972800
H	-4.348263644	0.209148828	2.739625002
S	2.344967969	3.269432726	1.676772799
C	3.642349304	2.830697438	0.663860902
N	3.597268267	2.619684283	-0.677553859
C	4.838550608	2.235515592	-1.163105656
C	5.689508546	2.223145927	-0.107731588
N	4.949257006	2.609221865	1.010248665
H	2.746214439	2.731479871	-1.219131373
H	4.999620046	2.005908264	-2.203806554
H	6.731082924	1.955770012	-0.015908027
C	5.436142828	2.625616278	2.380863664
H	4.796667102	3.299098622	2.951393909
H	6.472350997	2.967085693	2.383747732
S	8.048465126	0.730081665	2.420089232
C	6.487886812	0.457641149	2.996431844
N	5.403352559	1.311832559	2.978973608
C	4.269596629	0.691400675	3.512357454
C	4.649506367	-0.547701947	3.896926778
N	6.000606291	-0.672380352	3.586742653
H	3.301332086	1.169563826	3.495938123
H	4.080930492	-1.348983057	4.339493669
H	6.582813215	-1.482601101	3.730389062
C	2.144450074	-0.149660527	1.076879364
C	0.075424927	0.463018910	2.853876135
H	2.374603827	-0.680150382	1.997202927
H	3.040241336	0.170141562	0.547511532
H	0.381988722	-0.584363284	3.029451967
H	-0.476303474	0.886332369	3.695658772

8c
from **6c**$_{trans}$

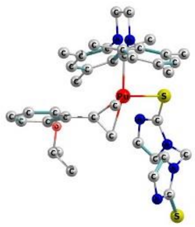

100
RuC40N6H50S2O

Ru	0.607755012	1.476126834	1.365674449
C	1.081616121	0.197822125	-0.293583070
H	1.448302481	0.891922864	-1.051769980
C	0.162704347	-0.794994570	-0.923580255
C	0.341745176	-1.124546892	-2.284807047
C	-0.510775891	-2.016601852	-2.940634076
C	-1.538646527	-2.646861097	-2.242509857
C	-1.710561176	-2.366834272	-0.885357394
C	-0.880468029	-1.446676849	-0.248713521
H	-0.352031245	-2.206883328	-3.998932671
H	-2.200188247	-3.340614712	-2.754679346
H	-2.509103347	-2.848038364	-0.326144677
H	-1.048451847	-1.179349653	0.788935748
O	1.370569488	-0.537445523	-3.012392761
C	2.459432422	-1.424459141	-3.356111440
C	3.284471765	-1.790456169	-2.124164707
C	3.278043826	-0.703752025	-4.418334471
H	2.036093985	-2.342783104	-3.790322376
H	2.656340574	-2.256899777	-1.361205435
H	4.082012194	-2.491001865	-2.396485414
H	3.733260592	-0.896940082	-1.680643194
H	2.668813979	-0.487744784	-5.301083035
H	3.655372597	0.245151680	-4.021608879
H	4.132961535	-1.314521349	-4.724877903
C	-0.986635850	2.864100156	1.455559573
N	-1.272901950	3.649246638	0.368998387
C	-2.502033209	4.446193614	0.505299842
C	-2.795925602	4.312178163	2.006319089
N	-1.906112678	3.202434645	2.399719862
H	-2.335302043	5.481437631	0.190917018
H	-3.305057059	4.025393242	-0.114393099
H	-2.536920108	5.220414168	2.567088132
H	-3.841817266	4.071536216	2.221285816
C	-0.647523669	3.459642963	-0.903166832
C	-1.061656534	2.391732581	-1.728262918
C	-2.209979115	1.502166146	-1.323283995
C	-0.374222038	2.167673569	-2.919730417
C	0.694766902	2.980294112	-3.317119539
C	1.428175251	2.676862413	-4.601153894
C	1.043630289	4.066680241	-2.508624089
C	0.373070156	4.334430490	-1.303543195
C	0.723749364	5.559334609	-0.496231400
H	-3.160532463	2.049773597	-1.351823602
H	-2.061571866	1.125170097	-0.308847162
H	-2.289778229	0.642094009	-1.990612005
H	-0.650167392	1.315698416	-3.534400690
H	2.384063406	3.207866426	-4.660928904
H	0.833654418	2.970064210	-5.475661268
H	1.618046081	1.602802264	-4.676318095
H	1.841019832	4.737463562	-2.826224678
H	0.175836796	6.431214142	-0.877697954
H	0.472233688	5.428547808	0.556445663
H	1.791969256	5.783479673	-0.560431047
C	-1.930076076	2.733470346	3.748568046
C	-1.056244904	3.276314741	4.700575200
C	-0.029270033	4.310670254	4.311761422
C	-1.119143338	2.787419126	6.010690134
C	-2.023906105	1.789577409	6.381504371
C	-2.091481608	1.287465824	7.805296981
C	-2.882561955	1.269636886	5.405641351
C	-2.849643164	1.724659853	4.087034714
C	-3.701706691	1.088673910	3.015824987
H	0.383463615	4.808058875	5.194203768
H	0.795045133	3.837481868	3.768694459
H	-0.444087450	5.076153287	3.648541960
H	-0.437999712	3.192692191	6.756314318
H	-2.280439850	0.209239530	7.838509308
H	-1.160920212	1.484685450	8.345875691
H	-2.904042467	1.775441330	8.358496084
H	-3.576325216	0.475376346	5.673932951
H	-4.358319455	1.814414826	2.522606410
H	-3.059266972	0.656031989	2.240446033
H	-4.329173419	0.295160499	3.431027418
S	2.387306793	3.312517834	1.524052819
C	3.616352861	2.640761798	0.546101380
N	3.583860596	2.509716956	-0.803992255
C	4.707919732	1.839160263	-1.263246379
C	5.476218555	1.567584339	-0.179109831
N	4.802152200	2.079729344	0.930264132
H	2.785609894	2.813119852	-1.356015185
H	4.856161810	1.614236803	-2.306599653
H	6.408545424	1.036769899	-0.061319101
C	5.218464299	1.928778023	2.318906963
H	4.718647372	2.706724279	2.895697243
H	6.303521738	2.032631346	2.369069608
S	7.295180091	-0.535069682	2.226372150
C	5.723554079	-0.449526330	2.833780055
N	4.871946916	0.637119566	2.862337081
C	3.641846109	0.291626506	3.431008890
C	3.727284916	-1.010380824	3.782504921
N	4.999092740	-1.445743973	3.418999157
H	2.806221136	0.974490665	3.438813610
H	2.992581634	-1.665533983	4.220508706
H	5.377490095	-2.372987638	3.533147755
C	2.012304326	-0.116538412	0.744786306
C	-0.081063092	0.235980392	2.546803236
H	1.901870335	-1.061988980	1.275609164
H	3.051747643	0.181196262	0.633933958
H	0.281353166	-0.799917502	2.649329855
H	-0.771559064	0.519149350	3.347597091

9c

100

RuC40N6H50S2O

Ru	0.086734094	0.960238938	1.646415477
C	3.842596708	-1.959896066	-0.143542558
H	4.610479322	-2.212418962	-0.869710911
C	2.467669832	-1.946204824	-0.668011662
C	2.240714490	-1.838491303	-2.058307801
C	0.944125217	-1.810744527	-2.577355687
C	-0.153253233	-1.887958177	-1.723277480
C	0.043714151	-1.970655457	-0.342876640
C	1.338948714	-2.003672164	0.169804022
H	0.804076066	-1.701474610	-3.648569529
H	-1.159030784	-1.852296282	-2.130550060
H	-0.801482665	-1.989554824	0.335042856
H	1.482935572	-2.091707728	1.242024214
O	3.332003054	-1.661947637	-2.890287420
C	3.515894929	-2.612700163	-3.966132561
C	3.875678665	-3.985870973	-3.404293633
C	4.604787543	-2.022200862	-4.849465577
H	2.581877081	-2.688606116	-4.540396939
H	3.095100461	-4.343363982	-2.726129914
H	3.987458685	-4.714054297	-4.214512943
H	4.818623157	-3.934455701	-2.849908592
H	4.304957270	-1.038961508	-5.224595805
H	5.530740322	-1.906250579	-4.277031779
H	4.802506643	-2.676728464	-5.703834702
C	-1.122741022	2.441272601	1.405275042
N	-1.038918386	3.259506627	0.284015577
C	-2.076331681	4.294251398	0.239697890
C	-2.557383670	4.313079656	1.696879511
N	-2.089679849	3.012134022	2.194931109
H	-1.670206848	5.257300068	-0.087747681
H	-2.882403975	4.013503326	-0.455204171
H	-2.102048141	5.136274967	2.266399629
H	-3.645057593	4.400583665	1.788076994
C	-0.306882720	2.903916144	-0.888474727
C	-0.775450165	1.889226045	-1.752938742
C	-2.045275466	1.135481022	-1.448200718
C	-0.016781699	1.578420742	-2.883477453
C	1.169391451	2.256698750	-3.191385486
C	1.985531863	1.843947996	-4.395685300
C	1.586542768	3.282074456	-2.340716979
C	0.872343446	3.611396759	-1.182091112
C	1.379585847	4.669183862	-0.232615669
H	-2.869254757	1.815346486	-1.207992676
H	-1.890402975	0.498934196	-0.569169976
H	-2.346802396	0.516549653	-2.298076000
H	-0.357611290	0.780776040	-3.539277884
H	2.878598972	2.465479218	-4.511673653
H	1.401981094	1.917450370	-5.321114744
H	2.311045153	0.799016642	-4.304704578
H	2.512926598	3.810052390	-2.556064688
H	0.705354360	5.532793203	-0.188180858
H	1.457393755	4.264189449	0.780947305
H	2.365257486	5.030275217	-0.540666450
C	-2.413373367	2.608951711	3.525850483
C	-1.615579244	3.011260346	4.608857577
C	-0.348033530	3.794460808	4.378718576
C	-1.997804595	2.616635186	5.894306926
C	-3.141222494	1.843409156	6.119624367
C	-3.548205868	1.457550314	7.523451778
C	-3.906674149	1.448577137	5.017935846
C	-3.557106699	1.817954563	3.716488609
C	-4.332175128	1.319859884	2.521705051
H	0.152049997	4.022121509	5.324368472
H	0.332278172	3.212232118	3.747557422
H	-0.538024977	4.741293459	3.860181941
H	-1.380541666	2.912291841	6.740633263
H	-4.118950524	0.523375815	7.532671875
H	-2.675481941	1.327301494	8.171735223
H	-4.180153854	2.230622312	7.980105141
H	-4.784960717	0.824940685	5.173359353
H	-4.715876455	2.144360364	1.909606121
H	-3.672015218	0.723228445	1.881752008
H	-5.180150829	0.701238784	2.829639826
S	2.367036748	1.762462893	2.101534360
C	3.322004661	1.543045304	0.687931949
N	2.908608403	1.097118010	-0.518449315
C	3.953406224	1.063113864	-1.428375697
C	5.052095188	1.515042572	-0.779669966
N	4.653144950	1.827077397	0.522757238
H	1.953748008	0.801356817	-0.690752941
H	3.828744669	0.668583341	-2.421968365
H	6.084442231	1.604840168	-1.076950793
C	5.528423455	2.274148097	1.588047272
H	4.950039750	2.902604307	2.266896636
H	6.354314041	2.833796568	1.146718923
S	8.303991737	0.857156006	0.713469554
C	7.253020562	0.518109875	1.982591013
N	6.091946230	1.171982366	2.341185215
C	5.509131483	0.571272805	3.458604852
C	6.312133896	-0.454288296	3.821225403
N	7.367072216	-0.476598659	2.914634783
H	4.565218535	0.916584387	3.848950872
H	6.223001049	-1.169005289	4.622975076
H	8.148245167	-1.113910924	2.908943230
C	4.210183777	-1.645729882	1.106118911
C	-0.905781955	-0.175649571	2.694223103
H	3.502959549	-1.312376185	1.859363766
H	5.253106547	-1.682499894	1.403520986
H	-0.543459935	-1.225834450	2.637045402
H	-1.625914531	-0.029215016	3.506550625

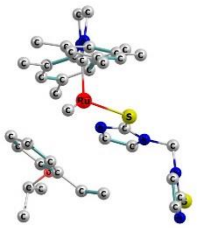

1d

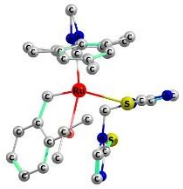

94
RuC38N6H46S2O

Ru	-1.029659436	1.892202531	1.382779541
C	-1.435658479	1.411865452	-0.388633504
H	-1.310271074	1.986885223	-1.310283210
C	-1.749542756	0.007452766	-0.650743549
C	-2.003661601	-0.873901289	0.425204480
C	-2.137287527	-2.246151884	0.240464965
C	-2.095204884	-2.778931765	-1.050599178
C	-1.900053074	-1.929660489	-2.142709477
C	-1.724725598	-0.563216741	-1.942274689
H	-2.246603317	-2.894821863	1.102492695
H	-2.201605304	-3.850250833	-1.196649403
H	-1.861673654	-2.339282682	-3.148818754
H	-1.532307787	0.091234722	-2.789434816
O	-1.962058207	-0.322394755	1.712869538
C	-3.252388995	-0.159688236	2.408073063
C	-2.950305286	0.593728465	3.695547474
C	-3.936682046	-1.494556271	2.691404704
H	-3.881383806	0.457790387	1.754419048
H	-2.575270632	1.599789979	3.487827260
H	-3.866662441	0.702377735	4.283639068
H	-2.208301826	0.049416673	4.288464572
H	-4.232446074	-2.010499327	1.776475803
H	-3.278984012	-2.149180373	3.274244662
H	-4.842376383	-1.310664236	3.279032192
C	-0.855756685	3.765484886	0.921855101
N	-0.195570602	4.452855932	-0.084130960
C	-0.414389691	5.909949940	-0.037648017
C	-1.570646372	6.051021242	0.964334145
N	-1.589503347	4.727918799	1.589907994
H	0.491692027	6.425987247	0.307098369
H	-0.660689648	6.298610668	-1.031855037
H	-1.399724645	6.841101088	1.704894153
H	-2.525208754	6.263881033	0.462984887
C	1.011219730	3.998449864	-0.705105867
C	1.010468823	3.648144784	-2.064066121
C	-0.236802511	3.731949393	-2.912891164
C	2.215321025	3.243160093	-2.653833964
C	3.415653815	3.218526981	-1.943239278
C	4.691313635	2.716547508	-2.575374744
C	3.396804970	3.630144196	-0.603795398
C	2.215918027	4.015214949	0.032324969
C	2.235094730	4.442762043	1.481328699
H	-0.118093268	4.494588700	-3.692620616
H	-1.113599486	3.982547290	-2.314091377
H	-0.430457097	2.780136952	-3.419785687
H	2.211435023	2.944165822	-3.700384577
H	5.568940292	3.233132303	-2.172120327
H	4.684313087	2.851662526	-3.661302556
H	4.814780935	1.645861344	-2.370883693
H	4.325100769	3.633248219	-0.036083754
H	2.080916014	5.523643088	1.588175038
H	1.441563374	3.946524833	2.044885761
H	3.196288448	4.199920957	1.943321882
C	-2.529836871	4.405972443	2.616307896
C	-2.119841988	4.521669841	3.956379310
C	-0.706346771	4.930877273	4.293709937
C	-3.037269771	4.214616959	4.967306983
C	-4.339566567	3.805368475	4.673458589
C	-5.296790930	3.414095757	5.775157980
C	-4.725989219	3.720680890	3.330603972
C	-3.843781365	4.014806742	2.289154198
C	-4.263334132	3.862982646	0.848334406
H	-0.603287543	5.136606307	5.363543114
H	-0.008836905	4.128181440	4.030443220
H	-0.397987679	5.823566156	3.739941124
H	-2.723070192	4.290093554	6.006483401
H	-5.252391624	2.333148713	5.962458672
H	-5.056119175	3.916500298	6.717355303
H	-6.332593080	3.655138149	5.514625955
H	-5.738412839	3.403077062	3.088017054
H	-4.183302259	4.806777226	0.296712655
H	-3.609264331	3.133394765	0.355705756
H	-5.299566628	3.519978364	0.775884263
S	4.492390637	-0.932060022	-0.875911144
C	3.090847989	-1.441580667	-0.080642217
N	2.684667207	-2.728897856	0.123978959
C	1.500196508	-2.780709278	0.852465375
C	1.144729904	-1.501253203	1.108838346
N	2.111174309	-0.684996519	0.522215422
H	3.217549432	-3.509298502	-0.225859430
H	1.015246200	-3.709382807	1.103615726
H	0.311051216	-1.079277409	1.645877096
C	2.113772738	0.758299065	0.572002531
H	1.074541884	1.145078550	0.569977831
H	2.665439278	1.136126325	-0.285540560
S	0.500907622	1.383112957	3.415659155
C	2.144399643	1.494877988	2.975395480
N	2.768274279	1.235973105	1.787225277
C	4.146680260	1.386591112	1.921225730
C	4.389060147	1.759327044	3.200667935
N	3.152190160	1.825673643	3.829913889
H	4.800514520	1.171039877	1.090461491
H	5.311775146	1.973851079	3.714436645
H	2.970541970	2.081450041	4.788203367

2d‡ (20.21*i* cm^{-1})

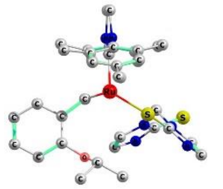

94
RuC38N6H46S2O

Ru	-0.944191772	1.935850697	1.510561795
C	-0.996757497	1.041905921	-0.107370868
H	-0.435304784	1.358670212	-0.992780903
C	-1.918250573	-0.077084344	-0.439707099
C	-1.899704277	-1.386907953	0.081194687
C	-2.776633762	-2.369038838	-0.394260434
C	-3.722978019	-2.073115195	-1.369488051
C	-3.774931359	-0.777329711	-1.892287068
C	-2.882416420	0.186443191	-1.441489610
H	-2.685487367	-3.371423268	0.012412720
H	-4.401561991	-2.843572230	-1.724856481
H	-4.502571256	-0.523313077	-2.659083593
H	-2.911923840	1.187071109	-1.863922998
O	-0.934587548	-1.776437793	0.996359561
C	-1.271039301	-1.684463721	2.401867925
C	-0.000022504	-2.084182523	3.138027686
C	-2.457176088	-2.566952691	2.786208208
H	-1.516613184	-0.638532605	2.628559804
H	0.846041285	-1.482554172	2.799972913
H	-0.121949853	-1.938098555	4.215282587
H	0.229710569	-3.138018305	2.946626801
H	-3.367296239	-2.267858853	2.260798835
H	-2.250018017	-3.617837737	2.553974721
H	-2.641419147	-2.479928721	3.862553401
C	-0.946373914	3.715404315	0.690977763
N	-0.409440295	4.294832985	-0.433046838
C	-0.708484457	5.731759072	-0.559527471
C	-1.653621885	5.999270056	0.629787839
N	-1.648991945	4.710505806	1.325473557
H	0.212133510	6.324983370	-0.499134004
H	-1.177570410	5.943894467	-1.527317382
H	-1.295244032	6.805725276	1.281216047
H	-2.669236282	6.260960374	0.305375991
C	0.672199279	3.730034361	-1.175595512
C	0.426166511	3.143517532	-2.426504108
C	-0.957032133	3.167553940	-3.031996684
C	1.490024326	2.520411538	-3.088843453
C	2.777670209	2.494223489	-2.547816779
C	3.911202596	1.792120824	-3.259415694
C	3.005728794	3.147190202	-1.330240174
C	1.970622786	3.774498525	-0.634675934
C	2.229280531	4.472075775	0.680355099
H	-1.103703693	4.068795160	-3.642237292
H	-1.721501680	3.166908782	-2.251743388
H	-1.120058048	2.301408422	-3.680246631
H	1.300026109	2.024990026	-4.039181094
H	4.426220423	2.471296338	-3.950428204
H	3.545861451	0.943498239	-3.847503175
H	4.655578016	1.427874863	-2.544408507
H	4.004924641	3.133708363	-0.902163642
H	1.989501884	5.539660281	0.622380866
H	1.607223138	4.050065112	1.476778837
H	3.280781802	4.388091390	0.968848176
C	-2.442301578	4.460675196	2.487050387
C	-1.887455899	4.717275411	3.752707645
C	-0.467402419	5.212876590	3.879431553
C	-2.668485135	4.465760241	4.885290257
C	-3.970949955	3.972123608	4.780978897
C	-4.787775920	3.664070325	6.014527694
C	-4.499384745	3.737475511	3.505824537
C	-3.756260200	3.970639980	2.348182290
C	-4.317640525	3.659675511	0.982860060
H	-0.198996505	5.366977864	4.928245797
H	0.226570037	4.480495519	3.455064461
H	-0.315337670	6.158822018	3.347359884
H	-2.244463866	4.650952634	5.869873055
H	-4.776031864	2.588614412	6.231629080
H	-4.398149040	4.181970661	6.896089447
H	-5.835643478	3.955394339	5.884455946
H	-5.512856640	3.352302536	3.412408172
H	-4.234439682	4.515317268	0.303192632
H	-3.752762742	2.832942712	0.535757704
H	-5.371490570	3.374475577	1.045471357
S	5.492645923	0.875509803	0.429653647
C	4.044829565	0.070978539	0.088645857
N	3.862229757	-0.946068643	-0.800690054
C	2.547251781	-1.402879996	-0.791165829
C	1.879159820	-0.650939205	0.112427914
N	2.797682186	0.256155855	0.644362676
H	4.624606551	-1.299551427	-1.356826064
H	2.209002453	-2.210820914	-1.418884680
H	0.849733094	-0.677324492	0.435627155
C	2.468129266	1.305018102	1.579853281
H	1.435612799	1.666172752	1.382181014
H	3.203942159	2.097299632	1.458427705
S	-0.092090578	1.478425219	3.822061470
C	1.519619676	0.907900716	3.893615015
N	2.519391275	0.827143137	2.963490374
C	3.653777709	0.236873361	3.524811639
C	3.358809772	-0.060855533	4.811172916
N	2.053232420	0.358631678	5.021555095
H	4.553243538	0.112141381	2.939784966
H	3.950655166	-0.521234068	5.585262858
H	1.515971474	0.271156263	5.870479146

3d’

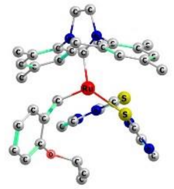

94
RuC38N6H46S2O

Ru	-1.141754106	1.833866293	1.322524008
C	-0.994519631	0.846952677	-0.218346995
H	-0.205694200	0.902845939	-0.979253042
C	-1.992381862	-0.228258733	-0.468566363
C	-1.655669134	-1.599190191	-0.489005559
C	-2.590197564	-2.569537570	-0.851986694
C	-3.897522521	-2.207713500	-1.172338525
C	-4.269261380	-0.863091102	-1.119114506
C	-3.328139866	0.103894367	-0.772368390
H	-2.270408208	-3.607375343	-0.861182656
H	-4.620342007	-2.970584262	-1.449043253
H	-5.288474208	-0.566642167	-1.352914223
H	-3.602189790	1.153941484	-0.739506430
O	-0.382594854	-2.035795919	-0.160899815
C	-0.069829008	-2.128965051	1.257691524
C	1.166590074	-3.014314377	1.344692992
C	-1.224425047	-2.667509431	2.096966050
H	0.163019213	-1.117953628	1.608263824
H	1.959501701	-2.636145043	0.694955761
H	1.545379445	-3.035805024	2.372127239
H	0.922589921	-4.036754950	1.036484197
H	-2.085511366	-1.996448576	2.073649963
H	-1.537498898	-3.656703687	1.746241757
H	-0.895079947	-2.754442685	3.137740414
C	-0.918366351	3.649821636	0.600574717
N	-0.303723216	4.239138912	-0.476737094
C	-0.380961802	5.711255387	-0.457306623
C	-1.430711371	5.980257428	0.635322373
N	-1.504794560	4.679723679	1.302121397
H	0.591437128	6.150935857	-0.203206736
H	-0.678850027	6.095100712	-1.438615014
H	-1.127367127	6.768933418	1.332890431
H	-2.404757184	6.260930861	0.210214640
C	0.679546695	3.595998411	-1.289892359
C	0.268767437	2.980823359	-2.487049084
C	-1.189767457	2.980881630	-2.872891767
C	1.233406809	2.364232924	-3.284907073
C	2.587407016	2.358033716	-2.927015977
C	3.601279760	1.653989116	-3.798862357
C	2.968206240	2.994979629	-1.745811803
C	2.031900148	3.619984750	-0.912243976
C	2.493013204	4.306505916	0.352024250
H	-1.564673843	4.002831346	-3.004144569
H	-1.790062056	2.516423969	-2.084330136
H	-1.352015553	2.434163203	-3.805976411
H	0.921601111	1.872849140	-4.204622696
H	3.534782450	1.986996805	-4.840932976
H	3.431881815	0.569922057	-3.799162619
H	4.623660200	1.832925358	-3.452600365
H	4.014305511	2.994719311	-1.447727597
H	2.673435395	5.375378966	0.178872514
H	1.745613227	4.224097847	1.145829123
H	3.435030181	3.878134310	0.707089141
C	-2.270513593	4.442988915	2.484891997
C	-1.608641294	4.485259882	3.726064759
C	-0.123505784	4.746219168	3.788647063
C	-2.346306065	4.231798462	4.884715878
C	-3.711486543	3.937413922	4.832993626
C	-4.476918001	3.610243201	6.094471351
C	-4.344971868	3.912381581	3.585525586
C	-3.646528462	4.158147735	2.400888259
C	-4.331159046	4.088171022	1.058102906
H	0.219165034	4.828098849	4.823977230
H	0.417170228	3.921264805	3.310873762
H	0.152834867	5.665402548	3.260544077
H	-1.840085225	4.249635821	5.847465338
H	-4.444269374	2.532698315	6.299884165
H	-4.053345009	4.120693571	6.965447270
H	-5.530867785	3.894415813	6.012002190
H	-5.406908940	3.681102551	3.532805398
H	-4.363590541	5.068378269	0.566938125
H	-3.785917986	3.403530796	0.399690209
H	-5.359584610	3.730899104	1.160121270
S	5.406724655	1.694697561	1.520126916
C	4.249049742	0.854065679	0.625945845
N	4.458269748	0.102487228	-0.494346057
C	3.277994667	-0.474196148	-0.950037391
C	2.298752357	-0.070977510	-0.110957239
N	2.892574174	0.742590100	0.854770476
H	5.376288798	0.006398081	-0.898436978
H	3.235141923	-1.101355698	-1.824829470
H	1.242159441	-0.274187278	-0.119710453
C	2.183888284	1.393675288	1.931921978
H	1.172545732	1.716719356	1.583024172
H	2.782423368	2.240691571	2.265145756
S	-0.770077714	0.874179063	3.543754931
C	0.818337191	0.247754136	3.729058807
N	1.988610139	0.475806473	3.058371509
C	3.016985617	-0.287597560	3.610757944
C	2.484898740	-1.011042461	4.623626079
N	1.140855373	-0.672439806	4.681600219
H	4.025399625	-0.212139532	3.233443660
H	2.934266344	-1.718009276	5.301661336
H	0.449826430	-1.032064934	5.322426992

3d

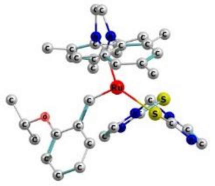

94
RuC38N6H46S2O

Ru	-0.868077518	1.917247960	1.517512331
C	-0.918794227	0.910360278	-0.050084656
H	-0.532393692	1.257043699	-1.008001391
C	-1.455537008	-0.460009532	-0.128621380
C	-1.530261013	-1.156654430	-1.372605878
C	-2.013780928	-2.470056755	-1.432188524
C	-2.449915783	-3.117430228	-0.276156802
C	-2.403760660	-2.457605408	0.951179440
C	-1.913263905	-1.158193862	1.011668650
H	-2.060571766	-2.999613749	-2.376088848
H	-2.822621315	-4.136204057	-0.344177800
H	-2.742530827	-2.953462700	1.856722496
H	-1.866550190	-0.651910285	1.970599852
O	-1.127134053	-0.456905353	-2.482927399
C	-1.010699925	-1.116968001	-3.752308407
C	0.259179199	-1.969397834	-3.790905513
C	-0.990943390	-0.015002895	-4.802466425
H	-1.896006955	-1.744616787	-3.922894055
H	0.250624805	-2.727290402	-3.002269278
H	0.358453849	-2.476608468	-4.756368030
H	1.128632555	-1.321163532	-3.642226306
H	-1.900554030	0.588094752	-4.745067082
H	-0.132677826	0.640875018	-4.638146486
H	-0.916722817	-0.448017136	-5.805316940
C	-1.024803015	3.640031346	0.603486635
N	-0.479744211	4.224222002	-0.519330482
C	-0.744308633	5.672579062	-0.602354881
C	-1.844471377	5.878675134	0.453871449
N	-1.770853411	4.626602197	1.206908552
H	0.161747843	6.246908609	-0.367092535
H	-1.064206436	5.955474894	-1.610754552
H	-1.659352692	6.746440391	1.097455809
H	-2.836739122	6.003986738	-0.002484982
C	0.649954182	3.684544597	-1.211277809
C	0.467043165	3.137475897	-2.491255434
C	-0.903290239	3.105547628	-3.124999877
C	1.570309303	2.569989356	-3.136636824
C	2.834553386	2.536888055	-2.541704589
C	4.004963254	1.887450054	-3.244267537
C	2.997933281	3.139811470	-1.290749423
C	1.925344682	3.731386710	-0.617732438
C	2.133726015	4.380599459	0.729764842
H	-1.474276793	4.011456378	-2.906810724
H	-1.479941984	2.258017543	-2.738564281
H	-0.829338304	2.995756323	-4.210326555
H	1.438351007	2.135455247	-4.125571719
H	4.402240471	2.533322957	-4.037454201
H	3.705381688	0.944327251	-3.715838355
H	4.819119884	1.678533992	-2.544107139
H	3.979285094	3.129962521	-0.822281858
H	1.851081536	5.439019246	0.720455877
H	1.514329499	3.891953226	1.489793092
H	3.183024814	4.323676473	1.033129664
C	-2.519063560	4.385793296	2.399621660
C	-1.922958019	4.683338394	3.638470705
C	-0.504677166	5.198272494	3.705304448
C	-2.656583848	4.450448432	4.806015702
C	-3.951854988	3.929956400	4.764005861
C	-4.715297828	3.644346604	6.036601818
C	-4.519394645	3.644517493	3.516088922
C	-3.823817552	3.860156060	2.325780354
C	-4.418493877	3.491780447	0.990059129
H	-0.241981861	5.492970607	4.725356966
H	0.197074451	4.420004138	3.384332847
H	-0.348600696	6.060417593	3.048351878
H	-2.201132042	4.672752718	5.768912769
H	-4.636392854	2.584515805	6.308661411
H	-4.329487934	4.228423109	6.877819122
H	-5.781014238	3.870985049	5.925294923
H	-5.525175380	3.231385622	3.471292425
H	-4.475461282	4.355295516	0.317345687
H	-3.786731280	2.738413046	0.505493364
H	-5.426960163	3.085318307	1.106408187
S	5.605231181	0.897274822	0.388687401
C	4.187768168	0.089832210	-0.049780055
N	4.052853554	-0.866260921	-1.013455233
C	2.744000924	-1.330437309	-1.093725761
C	2.033522602	-0.650082570	-0.165388514
N	2.918650191	0.219618384	0.470201251
H	4.838058288	-1.163599586	-1.570648263
H	2.438765778	-2.089399964	-1.793738387
H	0.988313987	-0.674863133	0.095888692
C	2.548393398	1.149585365	1.511790704
H	1.519253741	1.526478849	1.331785590
H	3.276480430	1.959251630	1.508867862
S	-0.111119462	0.901323975	3.628780643
C	1.520929782	0.365473499	3.688214920
N	2.570353529	0.498821162	2.823808148
C	3.713930959	-0.097663717	3.358567452
C	3.373732877	-0.612819075	4.562933020
N	2.030961808	-0.319541411	4.748393331
H	4.648005111	-0.078016667	2.816113860
H	3.956031032	-1.149634291	5.293918116
H	1.460582609	-0.556818136	5.545956347

4d

100

RuC40N6H50S2O

Ru	-1.764175748	1.464822146	0.936110977
C	-0.661215728	0.608030361	-0.256408816
H	0.394277872	0.909111538	-0.297841302
C	-0.917027570	-0.552591364	-1.148997348
C	-0.520982616	-1.866051796	-0.826892267
C	-0.614637320	-2.894808480	-1.767920184
C	-1.138332304	-2.651168183	-3.036098888
C	-1.567924174	-1.364278268	-3.362962268
C	-1.452177833	-0.335646954	-2.429779992
H	-0.273077048	-3.884059720	-1.478951322
H	-1.211777163	-3.459965452	-3.758330626
H	-1.985402882	-1.157108368	-4.345222134
H	-1.745398543	0.672903136	-2.694002026
O	0.023965528	-2.168323650	0.411528001
C	-0.924582496	-2.525284802	1.463716235
C	-0.083176825	-2.751621656	2.711140931
C	-1.745858932	-3.760254260	1.099567235
H	-1.588736407	-1.670117458	1.614904934
H	0.435816608	-1.838061799	3.007373108
H	-0.724312704	-3.057819423	3.543237257
H	0.657922719	-3.538065031	2.529757611
H	-2.350325343	-3.594968679	0.204447784
H	-1.094494933	-4.624457710	0.924569504
H	-2.424761522	-3.999863874	1.925005388
C	-0.935814700	3.361561110	0.739050034
N	-0.393592635	3.976229564	-0.366732069
C	-0.165327980	5.422525363	-0.213031173
C	-1.007136343	5.736178285	1.020699134
N	-1.132234818	4.403845327	1.638656636
H	0.904034201	5.631122870	-0.058015467
H	-0.489380496	5.965428942	-1.105917099
H	-0.532846936	6.449033244	1.699288460
H	-2.001661911	6.124435090	0.755636401
C	-0.046130897	3.364699041	-1.608947151
C	-1.016743986	3.334783601	-2.629237213
C	-2.403510976	3.863711713	-2.362840458
C	-0.677859274	2.764414769	-3.854829836
C	0.590447082	2.214103519	-4.082551779
C	0.885858902	1.492358063	-5.375440129
C	1.540914904	2.292645039	-3.065507649
C	1.248391477	2.872589102	-1.823968605
C	2.312135302	2.907691909	-0.751508361
H	-2.393837778	4.938302973	-2.142025499
H	-2.830001665	3.354999563	-1.492456064
H	-3.059051123	3.704132934	-3.223583060
H	-1.427933366	2.717046635	-4.642104944
H	0.385913197	1.965874217	-6.227155950
H	0.523503255	0.458071673	-5.316204590
H	1.959052383	1.454946104	-5.585839169
H	2.534029118	1.878379616	-3.229809274
H	3.231558681	3.373723298	-1.121941797
H	1.984644801	3.457659280	0.131615350
H	2.574801501	1.892805818	-0.431436955
C	-1.443157510	4.305778942	3.028503725
C	-0.440807134	4.639510848	3.968136629
C	0.933139481	5.105145581	3.545366403
C	-0.736485332	4.557865204	5.328789800
C	-1.995519836	4.155829599	5.784883793
C	-2.261002947	4.019394076	7.265628254
C	-2.976850487	3.862487072	4.839153176
C	-2.728507976	3.941257193	3.463768412
C	-3.835731031	3.609463433	2.495568340
H	1.708828063	4.681632073	4.190776754
H	1.158156148	4.828415872	2.514334131
H	1.018625565	6.196430059	3.617538817
H	0.041262682	4.800345493	6.050172547
H	-1.751346440	3.136934150	7.675851071
H	-1.888335035	4.885752614	7.822930159
H	-3.328275819	3.911981581	7.478933805
H	-3.968141251	3.562126305	5.173089062
H	-4.794327969	4.001101599	2.851765513
H	-3.642561651	4.006494286	1.497525484
H	-3.950264847	2.521960105	2.404088796
S	4.682924458	2.415848763	3.011969559
C	3.882446431	1.100340000	2.318153252
N	4.442993217	-0.009255626	1.754823670
C	3.472878121	-0.871047307	1.250970527
C	2.272455619	-0.301440762	1.503779191
N	2.526794345	0.901830860	2.163055483
H	5.442711965	-0.133185569	1.719472754
H	3.715066454	-1.800118464	0.761490405
H	1.276381094	-0.635890900	1.256289363
C	1.530751248	1.875707763	2.542725749
H	0.685734208	1.851617994	1.849102893
H	2.013105732	2.851110663	2.520826840
S	-1.529143246	0.614252648	3.235187914
C	-0.240928128	1.198684814	4.202652372
N	1.009681905	1.658215699	3.886462828
C	1.737683794	1.925038989	5.046898941
C	0.936314796	1.629732592	6.096829890
N	-0.264535506	1.186357917	5.563362556
H	2.756511128	2.278085593	4.981638427
H	1.110676638	1.691782602	7.158426597
H	-1.098323451	0.935063585	6.071692767
C	-3.606377665	1.182413492	-0.174572270
C	-3.488494748	0.070899375	0.711142899
H	-3.461264616	1.031806174	-1.240233564
H	-4.315866910	1.981977271	0.049451930
H	-3.232720513	-0.903423307	0.301011883
H	-4.109830811	0.021421821	1.607069755

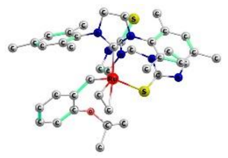

5d‡_trans_ (298.72_i_ cm^{-1})

100

RuC40N6H50S2O

Ru	-1.894069553	1.612830444	1.111080530
C	-1.235753034	-0.094756766	0.511032331
H	-0.984906800	-0.683762167	1.394607230
C	-0.780428954	-0.794599964	-0.697244217
C	-0.127220228	-2.051799104	-0.582408949
C	0.354003430	-2.726801046	-1.702564708
C	0.167795438	-2.191661190	-2.977314108
C	-0.485336411	-0.965745605	-3.118321210
C	-0.931150035	-0.274853874	-1.995493009
H	0.864844583	-3.673367198	-1.552434909
H	0.534522412	-2.726343825	-3.849858762
H	-0.626895670	-0.531387324	-4.103080909
H	-1.382748093	0.701482038	-2.105656188
O	0.105227459	-2.619826383	0.659406622
C	-1.018379274	-3.309885661	1.269001033
C	-0.750214140	-3.305725948	2.767216867
C	-1.145828048	-4.712358352	0.681110976
H	-1.931619229	-2.737218602	1.057523601
H	-0.718059729	-2.281972929	3.152707949
H	-1.536401836	-3.851523410	3.298488811
H	0.210068573	-3.790086503	2.976072920
H	-1.294803574	-4.664974999	-0.401653989
H	-0.236299491	-5.289709138	0.880630480
H	-1.998107238	-5.239704114	1.123017873
C	-1.016431466	3.401443608	0.838756313
N	-0.321586148	3.839680693	-0.259493066
C	0.186803338	5.219328321	-0.125374875
C	-0.671158880	5.750253752	1.016628703
N	-1.049379081	4.496772156	1.696472916
H	1.258231449	5.213414238	0.123641052
H	0.055184819	5.771108738	-1.059292236
H	-0.137662925	6.431001128	1.680318303
H	-1.573236630	6.262773045	0.651481195
C	-0.252966758	3.225483938	-1.549815558
C	-1.285418555	3.492584193	-2.469595688
C	-2.494334089	4.289901264	-2.044850198
C	-1.158952630	3.009553004	-3.773754936
C	-0.022373056	2.304122635	-4.188669384
C	0.107585099	1.826039673	-5.616040188
C	0.984817766	2.062654482	-3.253341562
C	0.885733899	2.501488306	-1.928361308
C	1.939549009	2.121100449	-0.921912716
H	-2.226768074	5.327495528	-1.806379178
H	-2.941128284	3.852679370	-1.147728020
H	-3.247828879	4.315047492	-2.836964248
H	-1.960680070	3.196568217	-4.485640153
H	-0.856233856	1.489343803	-6.013212809
H	0.815715992	0.995645778	-5.696309717
H	0.465015001	2.628991706	-6.273365746
H	1.858944915	1.486706605	-3.548580064
H	2.880617512	1.848703133	-1.407354203
H	2.145636809	2.921586349	-0.206800213
H	1.590848901	1.251393730	-0.355942276
C	-1.443516998	4.512785627	3.067397819
C	-0.493190845	4.872923642	4.046035865
C	0.932983006	5.233017478	3.697577424
C	-0.881031372	4.882499945	5.389794002
C	-2.174227920	4.540175001	5.788027619
C	-2.571349352	4.554727956	7.246602585
C	-3.097159960	4.192307355	4.798103818
C	-2.757738338	4.176321639	3.443884742
C	-3.762280060	3.725486576	2.419461709
H	1.612848047	4.921436950	4.494486782
H	1.257325175	4.756066023	2.770139294
H	1.059704890	6.316073465	3.570366914
H	-0.140805833	5.143087317	6.143895239
H	-1.699163009	4.451386106	7.900892476
H	-3.069432373	5.494788697	7.516417329
H	-3.269290573	3.743148824	7.479269537
H	-4.107958557	3.911519083	5.087070146
H	-4.785983428	3.819580299	2.794256435
H	-3.676626117	4.273635610	1.478529839
H	-3.616967908	2.649440229	2.196658694
S	4.856377019	2.118528172	2.329516644
C	3.826448849	0.814498684	2.029074034
N	4.167352724	-0.442394223	1.614857156
C	3.046583247	-1.257437371	1.474659157
C	1.975976631	-0.503195225	1.806821740
N	2.454771211	0.763042155	2.138175121
H	5.127776475	-0.695961933	1.444477355
H	3.096636501	-2.281758942	1.144814527
H	0.928864438	-0.743309369	1.823506840
C	1.625073211	1.894184721	2.482481815
H	0.711026327	1.874152438	1.882847189
H	2.207175158	2.793417832	2.280017962
S	-1.358766585	0.933145705	3.561755035
C	0.021790752	1.497546783	4.370155274
N	1.245097394	1.889617285	3.883015648
C	2.100680711	2.240816372	4.928193771
C	1.403813472	2.092712368	6.078816134
N	0.141041872	1.636213003	5.723289152
H	3.119096876	2.537787081	4.725778303
H	1.691190495	2.258011985	7.104405569
H	-0.643701974	1.484787217	6.337198056
C	-3.391164359	1.385517067	-0.376488311
C	-3.352535516	0.046258511	0.219151855
H	-3.192534502	1.457573698	-1.445565879
H	-4.245202645	2.004806043	-0.083098223
H	-3.219892190	-0.814628936	-0.426469343
H	-4.011027269	-0.177416829	1.058313646

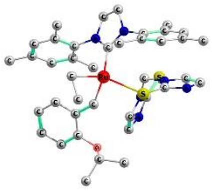

5d'‡_cis_ (328.50_i_ cm^{-1})

100

RuC40N6H50S2O

Ru	-1.132144716	1.258913690	1.051889649
C	-1.332359171	0.753652528	-0.848532763
H	-1.731124239	1.332445243	-1.679743907
C	-0.872713687	-0.558564223	-1.266273379
C	-0.603241791	-1.587238594	-0.324741255
C	-0.136980097	-2.838000482	-0.720921084
C	0.075120691	-3.106338580	-2.077989722
C	-0.182791882	-2.115745742	-3.029239670
C	-0.655747178	-0.870999141	-2.628600811
H	0.072299315	-3.604835453	0.013387208
H	0.441512861	-4.083697802	-2.379333589
H	-0.019873960	-2.319128052	-4.084668745
H	-0.855924747	-0.096327610	-3.364715889
O	-0.772407505	-1.197867291	1.000733824
C	-1.182106309	-2.130093072	2.052087741
C	0.006872727	-2.894258184	2.634910586
C	-2.356109586	-3.006742734	1.629698909
H	-1.521032445	-1.438776810	2.825337196
H	0.826377001	-2.205346829	2.854275565
H	-0.301370245	-3.366795230	3.574162303
H	0.381434638	-3.681295996	1.975707722
H	-3.169710945	-2.388846094	1.239908111
H	-2.084919335	-3.738021078	0.865469427
H	-2.726963114	-3.548178463	2.506409927
C	-0.839768067	3.192432243	0.846988605
N	-0.320950492	3.854968336	-0.262183395
C	0.091915540	5.236336815	0.014184175
C	-0.687995650	5.548472712	1.284600634
N	-0.960162825	4.203137678	1.808920898
H	1.179696316	5.291299488	0.173100311
H	-0.159088363	5.895881993	-0.821487886
H	-0.119625029	6.142657444	2.006516638
H	-1.627910219	6.079565878	1.070844464
C	-0.153601323	3.385956446	-1.602545148
C	-1.189326805	3.636376585	-2.520778851
C	-2.490959008	4.240847031	-2.054412795
C	-1.020781395	3.243179586	-3.850729269
C	0.149015396	2.611400698	-4.283316294
C	0.295637051	2.129740465	-5.707512679
C	1.168875177	2.393762786	-3.352907732
C	1.041251036	2.776539292	-2.015198932
C	2.120611880	2.445437485	-1.023087847
H	-2.348086002	5.209423956	-1.562202194
H	-2.970489815	3.585498814	-1.319634957
H	-3.182486862	4.383686925	-2.889692546
H	-1.827517456	3.417178150	-4.560112167
H	-0.434992735	2.601469750	-6.371429897
H	0.143773891	1.044525907	-5.766589965
H	1.296714653	2.336175574	-6.101164373
H	2.084269374	1.899361165	-3.672155573
H	3.074748739	2.234880582	-1.514189834
H	2.278653897	3.243988983	-0.292919323
H	1.813791315	1.552447467	-0.471586503
C	-1.738682241	4.142401266	3.004618823
C	-1.093697961	4.276536220	4.244662603
C	0.397039736	4.494270004	4.333752747
C	-1.859918640	4.228439495	5.413410080
C	-3.243331009	4.055637506	5.376586221
C	-4.048234039	3.923309907	6.648475648
C	-3.869145103	3.993260677	4.125774783
C	-3.143933441	4.061170721	2.934938157
C	-3.861614503	4.114594069	1.605900023
H	0.830760997	3.957060032	5.182807331
H	0.897046760	4.164924090	3.422236239
H	0.627229833	5.558272119	4.473843114
H	-1.356607623	4.312625937	6.375462736
H	-4.220126048	2.867351144	6.894510900
H	-3.533775472	4.378923188	7.500595415
H	-5.031761480	4.395974959	6.554369028
H	-4.953122049	3.906157910	4.078240029
H	-3.819074436	5.125630749	1.181411980
H	-3.407375237	3.439844563	0.879054979
H	-4.917069653	3.849365778	1.717416814
S	5.461825646	1.902698818	1.970317072
C	4.299628862	0.767669117	1.515576152
N	4.499107878	-0.405844575	0.842752949
C	3.304628753	-1.093053443	0.645228226
C	2.326929636	-0.342293964	1.202258749
N	2.939706610	0.796387790	1.728220012
H	5.420123126	-0.692731095	0.550867976
H	3.248857761	-2.031480342	0.117819818
H	1.256877103	-0.471367108	1.254029008
C	2.227302723	1.890446722	2.347528976
H	1.258634003	2.012987791	1.839268567
H	2.848320702	2.783421712	2.263290406
S	-0.771694116	1.005955073	3.559168817
C	0.748184778	1.246742835	4.271482129
N	1.958560716	1.629483516	3.750486280
C	2.926216892	1.701823361	4.754031882
C	2.320222937	1.371215973	5.917297468
N	0.992484977	1.097404877	5.608796128
H	3.945561545	1.964538035	4.512086364
H	2.706443754	1.301451343	6.921167627
H	0.257823581	0.848956599	6.252973000
C	-3.215676006	0.595402806	0.178864058
C	-3.155212315	0.769625919	1.601474490
H	-3.340239720	-0.410485906	-0.217415160
H	-3.706864147	1.362077841	-0.414782198
H	-3.191019988	-0.127254680	2.223196323
H	-3.662324753	1.612079621	2.057440384

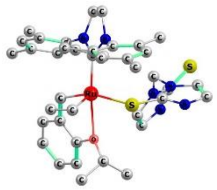

6d$_{trans}$

100

RuC40N6H50S2O

Ru	-1.947235904	1.630891561	1.342101161
C	-2.040287769	0.077674219	-0.041756848
H	-2.496455205	-0.520181604	0.781124302
C	-0.962636654	-0.713777031	-0.675974929
C	-0.347943585	-1.800818187	-0.008145677
C	0.666635241	-2.549115039	-0.605487822
C	1.100812887	-2.249609846	-1.897050951
C	0.496383515	-1.196876749	-2.584858218
C	-0.518373534	-0.454781971	-1.985587793
H	1.105484928	-3.362723658	-0.034481021
H	1.891697251	-2.835199151	-2.358436608
H	0.810917075	-0.950187157	-3.595666944
H	-0.972374117	0.356176676	-2.540891236
O	-0.698991173	-2.097839040	1.297957396
C	-1.814340040	-3.004980845	1.472690557
C	-2.329584008	-2.771609327	2.885482935
C	-1.381510055	-4.451403328	1.236450885
H	-2.593950606	-2.737134067	0.744244885
H	-2.642424898	-1.733164327	3.017174601
H	-3.177934252	-3.430483811	3.097560394
H	-1.535714028	-2.976440758	3.610977923
H	-1.012941436	-4.590219833	0.216536358
H	-0.583308475	-4.722999893	1.936106296
H	-2.225666437	-5.132749481	1.389879294
C	-1.352511998	3.357180105	0.739638921
N	-1.046219781	3.903076040	-0.489301536
C	-0.682512551	5.329562005	-0.401693204
C	-1.304950469	5.737352617	0.931759734
N	-1.371282543	4.443617981	1.613407288
H	0.412019054	5.444565279	-0.399781594
H	-1.080878749	5.883754640	-1.256153029
H	-0.696082341	6.454139517	1.490553353
H	-2.311689861	6.163597810	0.803789116
C	-0.684326371	3.248912187	-1.709594774
C	-1.549128439	3.351785603	-2.814149570
C	-2.929517993	3.956300942	-2.704182710
C	-1.122205750	2.850201175	-4.050324523
C	0.141634219	2.282902692	-4.220789150
C	0.595347220	1.769990386	-5.567646471
C	0.984607795	2.204770001	-3.108596827
C	0.591564554	2.667877580	-1.852794826
C	1.487043996	2.461435156	-0.662865386
H	-3.166082199	4.552109232	-3.591677440
H	-3.035114997	4.584479701	-1.818421252
H	-3.685569035	3.166689430	-2.623813605
H	-1.798323256	2.912014213	-4.901054095
H	-0.254822212	1.539160929	-6.216520460
H	1.197802714	0.861116157	-5.464274284
H	1.215878506	2.511366923	-6.086817937
H	1.963299590	1.741774106	-3.211787597
H	2.515163343	2.241786139	-0.962803598
H	1.500464916	3.327892182	0.004962970
H	1.105747951	1.609036621	-0.092826601
C	-1.731691324	4.337005548	2.991165609
C	-0.717843202	4.524237802	3.956454959
C	0.654152297	5.008817621	3.555150702
C	-1.015533164	4.291323401	5.296475041
C	-2.296198337	3.896321609	5.703671085
C	-2.577822939	3.611030876	7.160486796
C	-3.300399268	3.812706348	4.742249507
C	-3.052529669	4.053816949	3.383845477
C	-4.217294798	4.063910918	2.420223350
H	1.415344789	4.691977526	4.272332436
H	0.928941262	4.651064398	2.561331151
H	0.680057472	6.105395858	3.517510306
H	-0.225612626	4.397764480	6.037481811
H	-1.750169528	3.059421834	7.621837783
H	-2.700883386	4.538366571	7.734007654
H	-3.489607559	3.020001395	7.286287186
H	-4.312866824	3.555719880	5.047942860
H	-4.965214105	4.791887888	2.757973208
H	-3.912243523	4.321008002	1.406684027
H	-4.700841468	3.084851635	2.370211329
S	5.063731354	2.203164299	2.394001302
C	3.990421887	0.974819585	1.957461924
N	4.284493033	-0.230399695	1.387598501
C	3.138000104	-0.993812713	1.183332374
C	2.093722102	-0.260630719	1.630540282
N	2.620153396	0.946564556	2.091738369
H	5.232699252	-0.489494026	1.165392679
H	3.159303587	-1.967379949	0.724313036
H	1.036856164	-0.469222456	1.681315545
C	1.846703466	2.047447905	2.612142223
H	0.909001845	2.129754488	2.058818007
H	2.450915801	2.947598377	2.502027794
S	-0.956064824	0.704640655	3.582938245
C	0.357473850	1.297427224	4.461105149
N	1.519066320	1.873834419	4.011324057
C	2.353320081	2.202752328	5.079371116
C	1.702251122	1.850885307	6.212796064
N	0.487540706	1.298332081	5.822155010
H	3.332943847	2.620238281	4.901862714
H	1.994225534	1.930941971	7.247181454
H	-0.246873928	0.960747372	6.423896416
C	-3.632366578	1.757028204	0.100546313
C	-3.149919904	0.689544237	-0.889142961
H	-3.913721229	2.712083926	-0.350175262
H	-4.479462542	1.402050292	0.711932306
H	-2.731449421	1.155077681	-1.785410925
H	-3.912584601	-0.035362241	-1.232063979

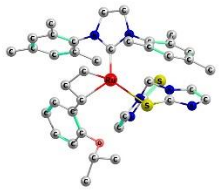

6d’$_{cis}$

100

RuC40N6H50S2O

Ru1	-0.906270333	1.350483532	1.090803688
C2	-1.983241231	0.794904477	-0.635226613
H3	-2.164108969	1.508990067	-1.435478130
C4	-1.311443841	-0.405808565	-1.214322769
C5	-0.674406463	-1.358390548	-0.392237945
C6	-0.000205971	-2.462543416	-0.909581489
C7	0.051595653	-2.659015808	-2.293477413
C8	-0.554587495	-1.727864728	-3.136856121
C9	-1.218035738	-0.622876014	-2.600261498
H10	0.493396394	-3.156823512	-0.239280248
H11	0.571843761	-3.522270376	-2.699070883
H12	-0.514262992	-1.862282715	-4.215316230
H13	-1.677046251	0.105842822	-3.262945886
O14	-0.647808131	-1.054868270	0.975861150
C15	-1.077952602	-2.035664265	1.979768319
C16	0.101178909	-2.865173901	2.484853587
C17	-2.253393413	-2.878380121	1.502647087
H18	-1.403631526	-1.390409781	2.797653522
H19	0.946083104	-2.219734880	2.736681766
H20	-0.200436261	-3.401762959	3.391400295
H21	0.431929160	-3.610419837	1.755496672
H22	-3.071425925	-2.239729144	1.162985109
H23	-1.972007968	-3.546291460	0.684103645
H24	-2.614320061	-3.489784229	2.336362307
C25	-0.872089432	3.280626767	0.874239311
N26	-0.303107802	3.913329015	-0.227980555
C27	0.028340253	5.322472327	0.014935903
C28	-0.835816726	5.645909370	1.235246621
N29	-1.114152227	4.307912757	1.772261978
H30	1.101620049	5.438928312	0.229831854
H31	-0.206974786	5.938474891	-0.857524079
H32	-0.317324148	6.263137730	1.975867252
H33	-1.769568017	6.157432415	0.959139156
C34	-0.104473068	3.359883794	-1.532461666
C35	-1.125315213	3.520840502	-2.489102520
C36	-2.418482487	4.202942932	-2.115997374
C37	-0.943558851	2.980755586	-3.763697029
C38	0.222998154	2.290673786	-4.110470114
C39	0.363640187	1.621613398	-5.455921772
C40	1.236748022	2.187344321	-3.156759176
C41	1.095402281	2.717793632	-1.870057744
C42	2.194838541	2.526675473	-0.857570767
H43	-2.257351738	5.227672613	-1.762333071
H44	-2.914386794	3.663507718	-1.302928712
H45	-3.103119207	4.244037533	-2.967497505
H46	-1.741907243	3.079670962	-4.496319674
H47	-0.332603972	2.036483963	-6.190935259
H48	0.153193213	0.548020980	-5.366130691
H49	1.379457856	1.720448585	-5.853038366
H50	2.156485235	1.663429024	-3.410206741
H51	3.182056840	2.526210067	-1.330330688
H52	2.182864883	3.299404020	-0.085247437
H53	2.074908975	1.557496608	-0.364818014
C54	-1.883990119	4.210832496	2.969904534
C55	-1.235210418	4.285229422	4.211385544
C56	0.260305939	4.462556811	4.300634553
C57	-2.005311891	4.212820546	5.376494545
C58	-3.392630560	4.074794594	5.330628970
C59	-4.204859186	3.928164589	6.596312262
C60	-4.017497211	4.049927390	4.078679836
C61	-3.287634733	4.131188360	2.891908749
C62	-3.989335924	4.164602514	1.554626060
H63	0.665719221	3.953993380	5.180380066
H64	0.754090769	4.062648096	3.413677443
H65	0.526813186	5.524293302	4.384442373
H66	-1.503949445	4.250788154	6.342075938
H67	-4.418560608	2.871809933	6.802026980
H68	-3.675355232	4.331177795	7.465240294
H69	-5.168928326	4.441878973	6.518343402
H70	-5.101387507	3.968056717	4.026859412
H71	-3.905735609	5.154363034	1.088399925
H72	-3.553679907	3.440768690	0.863276287
H73	-5.053549747	3.938181200	1.664287302
S74	5.824150696	1.604431611	2.110714196
C75	4.557594715	0.602734380	1.630232827
N76	4.643629748	-0.580991788	0.950430311
C77	3.388887527	-1.141893597	0.737998919
C78	2.485214960	-0.300814460	1.290775437
N79	3.203610531	0.770911398	1.825096107
H80	5.533656619	-0.959136646	0.666523361
H81	3.244099144	-2.067867831	0.206121999
H82	1.407225214	-0.348430597	1.345277473
C83	2.613612225	1.910948088	2.495302383
H84	1.670245231	2.166614405	2.005542748
H85	3.327932831	2.733387000	2.436235420
S86	-0.397080339	1.089410738	3.565200344
C87	1.094006810	1.222673760	4.354227738
N88	2.323505807	1.626229724	3.884476435
C89	3.280717861	1.584027401	4.901906750
C90	2.647637641	1.176515287	6.024542768
N91	1.316068540	0.964673727	5.680404061
H92	4.311944856	1.828943992	4.697265126
H93	3.016904790	1.018220702	7.024542489
H94	0.571634987	0.668756035	6.291826801
C95	-3.286621533	0.564553389	0.199039983
C96	-2.863336562	0.890346482	1.634427851
H97	-3.692089074	-0.451507004	0.075130349
H98	-4.065317285	1.254591221	-0.153931761
H99	-2.854176797	0.020131671	2.302467062
H100	-3.428853400	1.681855609	2.123143315

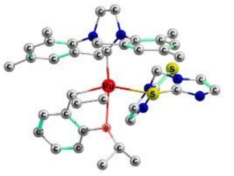

7d‡ (401.38*i* cm^{-1})

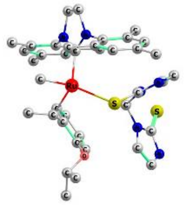

100
RuC40N6H50S2O

Ru	-2.235670089	1.720989786	1.243093899
C	-2.177866568	-0.075384900	0.001547155
H	-2.431243221	-0.455129298	1.023161364
C	-0.845460185	-0.541063429	-0.469709889
C	-0.194533032	-1.628543444	0.151033164
C	1.030310383	-2.103410991	-0.322059476
C	1.644568987	-1.499916803	-1.418088907
C	1.009611102	-0.429170358	-2.050193942
C	-0.218603033	0.035143102	-1.584033637
H	1.494168924	-2.932474628	0.201767100
H	2.609893662	-1.856216158	-1.765936643
H	1.477651834	0.059191230	-2.899105925
H	-0.701634023	0.874160296	-2.067626644
O	-0.724493204	-2.206703264	1.291388353
C	-1.794756807	-3.163501787	1.091701055
C	-2.525669137	-3.247305555	2.423778415
C	-1.221812291	-4.503714314	0.637614670
H	-2.471133180	-2.769669166	0.320814201
H	-2.909787609	-2.264529968	2.711888445
H	-3.361425194	-3.951558209	2.362205299
H	-1.840209082	-3.588384705	3.207233228
H	-0.673795501	-4.392734233	-0.302495314
H	-0.533310466	-4.894428462	1.395231053
H	-2.023153233	-5.234479678	0.484190514
C	-1.784460731	3.589558661	0.843108513
N	-1.522629122	4.188368731	-0.370034335
C	-1.432021433	5.659179664	-0.281227203
C	-2.085366392	5.941112258	1.072359030
N	-1.995440144	4.627368348	1.724707360
H	-0.382367502	5.984164034	-0.311245155
H	-1.954415290	6.128201652	-1.120282049
H	-1.566610306	6.710148658	1.652331483
H	-3.136303378	6.245929742	0.968575413
C	-0.866994819	3.590971554	-1.495602581
C	-1.574447171	3.393443949	-2.693080119
C	-3.067640750	3.587033845	-2.772074635
C	-0.868645662	2.963920790	-3.823102746
C	0.508797829	2.732837445	-3.793153572
C	1.234067070	2.223445515	-5.016965184
C	1.187222916	2.939124823	-2.588418562
C	0.522421395	3.363403071	-1.436407601
C	1.275868427	3.547798113	-0.143891395
H	-3.379669982	3.832475415	-3.791510246
H	-3.416163445	4.372806026	-2.097751501
H	-3.572795281	2.662010840	-2.471382304
H	-1.413670611	2.804528618	-4.751531246
H	0.822451327	2.651907826	-5.936745297
H	1.141736754	1.132624865	-5.099509560
H	2.302100728	2.458795981	-4.979018144
H	2.257844905	2.752322896	-2.539234638
H	2.324882576	3.259755792	-0.254693028
H	1.252538679	4.586334698	0.205054299
H	0.821853973	2.940654971	0.643937628
C	-2.130192550	4.524007111	3.145206540
C	-0.976352829	4.748811909	3.921391910
C	0.344756226	5.044490389	3.255171836
C	-1.079034736	4.694091730	5.313595575
C	-2.297488259	4.422307572	5.947405441
C	-2.383158587	4.313888825	7.451906980
C	-3.429806610	4.243218896	5.151390112
C	-3.376229732	4.304704194	3.753876304
C	-4.637899802	4.120530774	2.949972293
H	1.140373382	5.175389903	3.992657885
H	0.633896763	4.232872672	2.583632132
H	0.301605123	5.957226717	2.650486523
H	-0.194680462	4.892184064	5.919187297
H	-1.574761269	4.864835849	7.943282253
H	-3.334974261	4.701554993	7.829154936
H	-2.311471408	3.267093910	7.772697143
H	-4.390086046	4.058617725	5.629041676
H	-5.508552215	4.482356838	3.506406320
H	-4.590400620	4.639755176	1.990213601
H	-4.783059956	3.060825950	2.716868795
S	5.185580853	0.160227048	1.010243461
C	4.029511571	-0.813569487	1.759088571
N	4.229782800	-2.027214138	2.354325341
C	3.052200307	-2.528104679	2.902522518
C	2.083628306	-1.620115447	2.642994762
N	2.683084344	-0.581334762	1.931428489
H	5.142830795	-2.452737094	2.383441214
H	3.009023529	-3.479944763	3.406290089
H	1.020752474	-1.628058144	2.831514420
C	2.014745184	0.635824255	1.521653465
H	1.017900392	0.408087328	1.148359246
H	2.617611903	1.097161823	0.741236470
S	-0.780283710	0.985826385	3.238173498
C	0.780808642	1.599975397	3.468483032
N	1.889023539	1.542343196	2.651099144
C	2.973968612	2.180305902	3.258116138
C	2.539388242	2.662644621	4.445059593
N	1.204337671	2.295297729	4.565285218
H	3.943174271	2.190712609	2.782377922
H	3.052246469	3.229131066	5.205292254
H	0.542587850	2.624099403	5.254119019
C	-3.915320467	1.984316512	0.391427735
C	-3.263242514	-0.076246661	-0.894216042
H	-4.232812127	2.628928196	-0.435440916
H	-4.765166330	1.407659767	0.792893911
H	-3.095581182	-0.082857933	-1.967529966
H	-4.266601888	-0.294401423	-0.553546044

8d

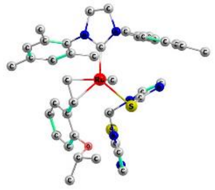

100
RuC40N6H50S2O

Ru	-1.946432813	1.638977208	1.129270812
C	-2.369145889	-0.122250371	-0.068356453
H	-2.942830556	-0.850379083	0.489510201
C	-1.072767995	-0.606337585	-0.564933572
C	-0.448806493	-1.744432530	0.017175006
C	0.782817469	-2.210672520	-0.433340172
C	1.467285991	-1.546967673	-1.455747509
C	0.890174791	-0.415084322	-2.026725724
C	-0.357073045	0.040511794	-1.595543400
H	1.211428565	-3.077914728	0.057708105
H	2.438163113	-1.905092283	-1.785993195
H	1.408554077	0.124385336	-2.813015046
H	-0.786769228	0.915023154	-2.068068364
O	-1.015143141	-2.357440990	1.117281805
C	-2.040671196	-3.344444908	0.862692069
C	-2.889884646	-3.392854266	2.125298170
C	-1.403045465	-4.687493877	0.514178211
H	-2.652885492	-3.002319434	0.016376930
H	-3.308199092	-2.404947228	2.338028180
H	-3.707160416	-4.113212939	2.017042147
H	-2.271969219	-3.691811894	2.978821083
H	-0.781581760	-4.599231016	-0.381679379
H	-0.771565288	-5.028381105	1.342049183
H	-2.171430729	-5.445233262	0.326148026
C	-1.738335973	3.653525003	0.731713824
N	-1.441320970	4.277783057	-0.459291181
C	-1.601847814	5.741873771	-0.415723752
C	-2.456519988	5.923813358	0.839473638
N	-2.217514468	4.656068716	1.545563955
H	-0.620606168	6.232418226	-0.330150860
H	-2.088503358	6.110690854	-1.323022665
H	-2.149651412	6.777370117	1.451096600
H	-3.523134568	6.038093965	0.598269700
C	-0.752747109	3.707697814	-1.570352485
C	-1.447587702	3.521292640	-2.779488788
C	-2.918364444	3.844979566	-2.888327706
C	-0.746150858	3.034212813	-3.887011136
C	0.619043138	2.741936092	-3.824516064
C	1.340633121	2.163791547	-5.019400627
C	1.287237046	2.953235051	-2.614526528
C	0.624898117	3.431606465	-1.481989253
C	1.370427576	3.645417690	-0.189361487
H	-3.401901348	3.213396057	-3.639333360
H	-3.071924370	4.888419927	-3.193113638
H	-3.424572902	3.696377127	-1.931946947
H	-1.283313517	2.870307288	-4.819128095
H	0.961486857	2.578983030	-5.958904013
H	1.202997073	1.075777506	-5.067153272
H	2.416947148	2.355506228	-4.971152883
H	2.350088623	2.730610115	-2.547204952
H	2.433055847	3.412486167	-0.301742227
H	1.285747106	4.681233316	0.158140188
H	0.956589894	3.017510784	0.604602845
C	-2.379926023	4.568394799	2.958435273
C	-1.248331929	4.783102677	3.767266549
C	0.066862601	5.152505745	3.127952462
C	-1.385252897	4.681729259	5.153094561
C	-2.622917061	4.395370472	5.745724034
C	-2.743088045	4.238369076	7.243582088
C	-3.737622659	4.249920633	4.917441657
C	-3.641662223	4.342242476	3.523703577
C	-4.867913824	4.228654812	2.652393641
H	0.839036007	5.337297944	3.879583931
H	0.415125001	4.360282904	2.461713167
H	-0.030712452	6.059673547	2.520495772
H	-0.519802853	4.864848913	5.789845374
H	-2.014016088	4.860024035	7.773853733
H	-3.742262460	4.510484235	7.597680502
H	-2.564210233	3.197908025	7.542312117
H	-4.709770747	4.052398785	5.364748211
H	-5.312305795	5.218192574	2.483276916
H	-4.617923982	3.794936176	1.682420852
H	-5.630063364	3.601787829	3.123958689
S	5.387319963	0.162144960	1.003445345
C	4.214144296	-0.870547785	1.639378247
N	4.408041664	-2.095722856	2.210495315
C	3.208525841	-2.650084951	2.649649945
C	2.232411712	-1.764473018	2.344675129
N	2.851793017	-0.683618606	1.714997401
H	5.328787999	-2.496813824	2.292641053
H	3.159060686	-3.616241577	3.124533230
H	1.161343140	-1.795978990	2.479044041
C	2.184885171	0.524200232	1.282293890
H	1.193847776	0.276160208	0.904595299
H	2.789338361	0.982790914	0.500714843
S	-0.492894483	0.633028452	3.072883691
C	0.941316776	1.524204403	3.202558518
N	2.043775367	1.463671982	2.385814437
C	3.040271774	2.323409488	2.845928809
C	2.550097524	2.942946732	3.946736309
N	1.272159596	2.438709049	4.155943628
H	4.000218505	2.368324302	2.354709450
H	2.991329463	3.684534677	4.592193679
H	0.585433418	2.767733227	4.820460366
C	-3.407895999	1.430312531	2.198552741
C	-3.050933384	1.037466770	-0.591353854
H	-3.298949530	1.453192717	3.290561416
H	-4.438961742	1.286330527	1.838293910
H	-2.720650853	1.473836438	-1.530482930
H	-4.135858600	1.073331948	-0.473834450

9d

100

RuC40N6H50S2O

Ru	-1.787554824	0.997012021	0.306967811
C	-3.735983154	-2.086354000	-2.683313555
H	-4.199911275	-1.572952090	-1.844254700
C	-2.284139486	-1.883002266	-2.817697617
C	-1.495226270	-1.533342652	-1.702598452
C	-0.122139875	-1.330563931	-1.829659221
C	0.496571963	-1.460978565	-3.068714081
C	-0.264963698	-1.800083808	-4.190539690
C	-1.634089046	-2.002126347	-4.060049054
H	0.442099081	-1.045320865	-0.953293328
H	1.564631276	-1.284985863	-3.159093428
H	0.205720210	-1.891742342	-5.165590976
H	-2.229985837	-2.226103170	-4.940144015
O	-2.074560204	-1.369379567	-0.447973322
C	-2.502375938	-2.609773239	0.204291765
C	-3.675143240	-2.266904013	1.107807256
C	-1.318155151	-3.233913025	0.933777700
H	-2.842731546	-3.288741437	-0.586307451
H	-4.489288605	-1.824569803	0.527480281
H	-4.044598076	-3.175516583	1.595577675
H	-3.378403282	-1.554008983	1.879847626
H	-0.487478421	-3.394731746	0.238709454
H	-0.987205426	-2.580178225	1.744967139
H	-1.600624236	-4.203824054	1.357434287
C	-1.367653963	2.901885560	0.371514187
N	-0.992278920	3.662994907	-0.731608317
C	-0.665552017	5.056313296	-0.402733604
C	-1.379787709	5.224167420	0.929491784
N	-1.426112875	3.833991072	1.411415133
H	0.424455667	5.184530050	-0.306944972
H	-1.022206501	5.744597901	-1.173523343
H	-0.851586649	5.879623003	1.625409150
H	-2.401638558	5.612684806	0.800070772
C	-0.643717958	3.188099217	-2.036360508
C	-1.462843271	3.549413665	-3.123118208
C	-2.675166791	4.435479810	-2.950051302
C	-1.139542249	3.077796587	-4.399318102
C	-0.014519562	2.286166827	-4.628179843
C	0.272434762	1.701212232	-5.990053694
C	0.827088203	2.013643405	-3.546471083
C	0.539377046	2.456509838	-2.255612687
C	1.467626590	2.100836060	-1.126403687
H	-3.481238030	4.131183525	-3.625020613
H	-2.434715047	5.481172066	-3.185898250
H	-3.056330574	4.403863334	-1.927232620
H	-1.795702477	3.326342369	-5.231725026
H	-0.049788342	0.652598636	-6.027380981
H	1.343463669	1.720029405	-6.220700564
H	-0.255668197	2.239438164	-6.783297796
H	1.728068450	1.425482892	-3.709834501
H	2.466240597	1.846049312	-1.493510637
H	1.564936569	2.914504870	-0.401073188
H	1.058232809	1.236937119	-0.594100785
C	-1.795734661	3.605738691	2.771223830
C	-0.846597450	3.906494027	3.771856232
C	0.513554691	4.474242821	3.436740944
C	-1.181007804	3.692294322	5.111279972
C	-2.429256771	3.187657620	5.480732645
C	-2.760437461	2.945022916	6.935753623
C	-3.367558812	2.942987293	4.474962984
C	-3.087704716	3.165980140	3.122885763
C	-4.141641295	2.922536668	2.078289826
H	1.272751413	4.113230661	4.136815185
H	0.820020332	4.214771583	2.421291894
H	0.514281303	5.570057226	3.504226294
H	-0.438685964	3.903559062	5.878517202
H	-1.890714697	2.565814612	7.485567248
H	-3.073479256	3.870001423	7.436367418
H	-3.575105179	2.222596286	7.048135549
H	-4.354412814	2.573582651	4.746856706
H	-4.197753058	3.755969806	1.369071097
H	-3.875117952	2.036451642	1.481361918
H	-5.126093296	2.784511175	2.535830996
S	4.760197642	1.627090599	2.298806247
C	3.729864607	0.422543038	1.731598089
N	4.072512015	-0.780429798	1.176777456
C	2.949457702	-1.541881761	0.869520481
C	1.871159115	-0.805648876	1.223957627
N	2.351555058	0.400963241	1.732325103
H	5.037111359	-1.042944450	1.047206656
H	3.016366263	-2.524819632	0.432205963
H	0.813244932	-1.010754380	1.190557258
C	1.529543087	1.486788881	2.216539417
H	0.685888367	1.648901058	1.543477763
H	2.160666272	2.373358811	2.263028027
S	-1.387081084	0.076438109	2.679512898
C	-0.214863399	0.629055009	3.776953572
N	1.003339839	1.210464565	3.537793914
C	1.692561604	1.416494207	4.730922685
C	0.885925528	0.990566139	5.731709935
N	-0.269974293	0.506478877	5.133324384
H	2.691130807	1.826078300	4.726477510
H	1.029968279	0.982602984	6.799714179
H	-1.106435494	0.194370054	5.601306169
C	-2.934992539	1.342726946	-1.107565381
C	-4.501518226	-2.839574965	-3.484746675
H	-3.284710959	2.272060851	-1.566482625
H	-3.515997373	0.482693549	-1.479414010
H	-4.091343956	-3.409092617	-4.314791196
H	-5.572997569	-2.915373790	-3.327040663

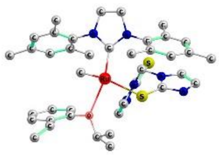

3d$_r$

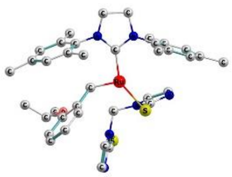

94
RuC38N6H46S2O

Ru	-0.956521168	1.861303419	1.385190772
C	-0.080723810	1.215999245	-0.102288485
H	0.787796118	1.732745722	-0.529011711
C	-0.275818465	-0.108830406	-0.711168351
C	0.839319518	-0.831811149	-1.214920931
C	0.669279616	-2.070300807	-1.841871060
C	-0.609810891	-2.618984107	-1.963476291
C	-1.718205101	-1.940367256	-1.455916647
C	-1.543593727	-0.703365148	-0.839507336
H	1.523324817	-2.618974217	-2.222786797
H	-0.730726497	-3.582197593	-2.452820694
H	-2.712819517	-2.367568939	-1.550112958
H	-2.393166764	-0.149301940	-0.452266253
O	2.062626030	-0.263849035	-0.968299030
C	3.235464068	-0.700605129	-1.676304189
C	4.410386763	-0.064202040	-0.944547105
C	3.153677416	-0.276326503	-3.142416021
H	3.322211322	-1.793990810	-1.598742890
H	4.472815747	-0.413459151	0.089541926
H	5.351285046	-0.314299224	-1.444493872
H	4.298961848	1.025276303	-0.940221197
H	2.287313760	-0.721410619	-3.638508577
H	3.053946061	0.812212266	-3.201575590
H	4.056666122	-0.579381696	-3.682510854
C	-1.322011459	3.655039940	0.651656321
N	-1.250747071	4.213099973	-0.601710891
C	-1.806984467	5.572203634	-0.674600800
C	-1.942695356	5.957603944	0.808405138
N	-1.789557859	4.658683068	1.473729660
H	-1.142738985	6.243045768	-1.229860838
H	-2.778643898	5.562667264	-1.187885332
H	-1.156705414	6.657297110	1.125862921
H	-2.911263457	6.412748078	1.043741729
C	-1.011916069	3.473804487	-1.799714447
C	-2.021980223	2.662028282	-2.346765731
C	-3.366478776	2.550918931	-1.675133054
C	-1.717600847	1.895751160	-3.472815221
C	-0.448944808	1.919845494	-4.060779173
C	-0.122464749	0.991418348	-5.206211918
C	0.520603016	2.767538708	-3.519398885
C	0.259738886	3.546550379	-2.388106750
C	1.344569468	4.372950520	-1.741989888
H	-3.841756090	3.529225947	-1.540425734
H	-3.237866463	2.116662627	-0.675320409
H	-4.046203820	1.919170242	-2.253803717
H	-2.478962809	1.233379884	-3.879712554
H	-0.941245318	0.939032684	-5.932006626
H	0.042873680	-0.025970870	-4.829732345
H	0.783547602	1.301680991	-5.735551991
H	1.512871099	2.797902123	-3.965385924
H	1.095161531	5.440668192	-1.727787911
H	1.483454205	4.064999432	-0.699180513
H	2.295875932	4.257202195	-2.269159347
C	-1.839542644	4.543337564	2.897047122
C	-0.706069018	4.839490777	3.674113618
C	0.615923734	5.139452077	3.013863774
C	-0.822802101	4.777641611	5.067989109
C	-2.016630291	4.400574409	5.693340763
C	-2.131762415	4.368962841	7.200537291
C	-3.114684645	4.072654262	4.890253178
C	-3.049040623	4.143397513	3.497188213
C	-4.226337436	3.756492211	2.635220606
H	1.412735981	5.248794630	3.754693553
H	0.873147763	4.313914678	2.338597396
H	0.581329691	6.056277964	2.414657867
H	0.043452789	5.029052735	5.678451575
H	-1.150142679	4.309940032	7.682006179
H	-2.623088823	5.275368163	7.575384916
H	-2.728215044	3.515117153	7.538458525
H	-4.042447935	3.753989680	5.360628000
H	-4.511882548	4.568389022	1.956911061
H	-3.968798873	2.895300694	2.006641882
H	-5.094999250	3.495483426	3.246145648
S	5.168260315	-0.822604201	3.102453807
C	3.702584578	-1.517063520	2.639429563
N	3.400480811	-2.845315645	2.546883634
C	2.097431943	-3.041511506	2.095782732
C	1.563035901	-1.814719256	1.902685073
N	2.546569580	-0.885354576	2.238366595
H	4.073859242	-3.555538307	2.786423315
H	1.674187555	-4.022486037	1.956091236
H	0.589850949	-1.500290217	1.564122746
C	2.410895939	0.549364514	2.154266123
H	1.622892423	0.796097264	1.438378865
H	3.363911205	0.964362682	1.827265109
S	-0.666748253	0.563115649	3.402312122
C	0.800106663	1.296953402	3.917862025
N	2.074359757	1.166838242	3.428151989
C	2.964712597	1.892669161	4.215967032
C	2.245791975	2.472990983	5.206490090
N	0.927065500	2.079596773	5.023159609
H	4.022451773	1.870867027	4.008836053
H	2.552231960	3.096925122	6.029838394
H	0.115346052	2.450793846	5.497122671

4d$_r$

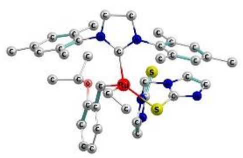

100
RuC40N6H50S2O

Ru	-1.716293409	1.667464683	1.109806480
C	-0.609539810	0.924460846	-0.201472022
H	-0.189694283	1.458819624	-1.050410879
C	-0.289710290	-0.508141277	-0.265638224
C	0.978276876	-0.938664308	-0.749227274
C	1.265017900	-2.298161281	-0.898502137
C	0.320064766	-3.260240582	-0.529756372
C	-0.913275295	-2.867495420	-0.015386608
C	-1.208931070	-1.508767546	0.101725757
H	2.229093284	-2.622952421	-1.270446837
H	0.559108942	-4.314015798	-0.648490338
H	-1.653634590	-3.610394098	0.268899345
H	-2.183092697	-1.193928330	0.459056309
O	1.883504255	0.065930015	-0.978054016
C	3.150386561	-0.233632753	-1.591945865
C	4.045547961	0.966922413	-1.321389697
C	2.958855371	-0.499525454	-3.085821192
H	3.589815896	-1.111593024	-1.097584001
H	4.174866024	1.122653047	-0.246939206
H	5.034709130	0.802981462	-1.760531740
H	3.611923463	1.866701232	-1.765255172
H	2.290029998	-1.346326708	-3.260361244
H	2.515385531	0.383606382	-3.556091934
H	3.919834850	-0.710977418	-3.566409392
C	-1.419034957	3.530556704	0.464799572
N	-1.249467600	4.153036670	-0.746372521
C	-1.498526047	5.602681659	-0.723116358
C	-1.576448497	5.904346097	0.779384294
N	-1.677994618	4.558785083	1.359108134
H	-0.690592119	6.143126488	-1.228727108
H	-2.436601088	5.835665345	-1.245115985
H	-0.677570531	6.418772826	1.147467441
H	-2.443616936	6.518682423	1.045134704
C	-0.908460571	3.550977110	-1.995291742
C	-1.907048598	3.166986599	-2.900179227
C	-3.372926467	3.387808194	-2.616974904
C	-1.516364002	2.571826733	-4.106764476
C	-0.174118649	2.382632515	-4.435079768
C	0.230266634	1.681701373	-5.710762081
C	0.801277498	2.827609247	-3.533568413
C	0.457200929	3.405265878	-2.312503129
C	1.520840408	3.783400009	-1.312204335
H	-3.778336070	4.173629024	-3.266908818
H	-3.543763691	3.674064418	-1.580239178
H	-3.948973460	2.477175332	-2.809239425
H	-2.287233333	2.245113640	-4.802336222
H	-0.587216982	1.659729899	-6.437474419
H	0.518313686	0.642717460	-5.505833504
H	1.091547834	2.168433184	-6.181254603
H	1.853668242	2.705665361	-3.782049665
H	1.279485161	4.707031993	-0.778990953
H	1.614726639	2.990759072	-0.560980742
H	2.492289755	3.909678541	-1.798093344
C	-1.548157275	4.408112084	2.774962961
C	-0.263730533	4.333978164	3.352766434
C	0.972511928	4.311743891	2.488203182
C	-0.163319853	4.251421082	4.740361837
C	-1.297468253	4.219710333	5.559879133
C	-1.151438786	4.129720604	7.061643832
C	-2.555617552	4.293765763	4.960824761
C	-2.701944970	4.403301956	3.572585014
C	-4.069560259	4.515770475	2.945734424
H	1.873135763	4.185951499	3.094902018
H	0.908765884	3.487707017	1.770706930
H	1.084969076	5.234153035	1.906856768
H	0.823748472	4.185881068	5.191418189
H	-0.299759219	3.499345810	7.343479053
H	-0.972629979	5.117277029	7.505292878
H	-2.050500919	3.718762666	7.531447868
H	-3.446874206	4.269818015	5.584614284
H	-4.198005658	5.479523849	2.438809106
H	-4.226106032	3.737950507	2.194815298
H	-4.854087249	4.428528265	3.701918768
S	5.131826625	0.392110524	2.527936737
C	3.867893091	-0.723308741	2.412067722
N	3.957599372	-2.083642310	2.484691416
C	2.703365277	-2.683030029	2.405495120
C	1.803231620	-1.685178540	2.260945466
N	2.520940663	-0.488795241	2.246484845
H	4.844533103	-2.545078862	2.611829816
H	2.569009039	-3.751884561	2.423281291
H	0.732748375	-1.698187645	2.126200292
C	1.924403368	0.830740620	2.258132712
H	1.075719771	0.878494369	1.573876327
H	2.690003135	1.546163585	1.959856180
S	-1.208752163	0.397160202	3.291326449
C	0.205645780	0.921048792	4.080026355
N	1.468850621	1.158809488	3.601230804
C	2.325819322	1.519401966	4.642869144
C	1.590560871	1.525430071	5.779464574
N	0.301862667	1.156814834	5.419805701
H	3.375099519	1.691543901	4.456016059
H	1.862893722	1.758681336	6.795904602
H	-0.514322479	1.152420792	6.011823162
C	-3.735215221	1.682174969	0.327544350
C	-3.779843751	0.956059726	1.545961013
H	-3.764014128	1.150399578	-0.620929695
H	-4.167013616	2.681295339	0.280696328
H	-3.832652356	-0.130745880	1.545095999
H	-4.157537554	1.419293431	2.456968205

5d$_r$‡$_{cis}$ (397.51*i* cm^{-1})

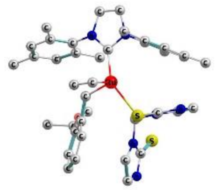

100

RuC40N6H50S2O

Ru	-1.480616172	1.869735671	1.373240240
C	-1.116791832	0.774615660	-0.205823135
H	-0.999596284	1.207505727	-1.197489438
C	-0.849868380	-0.669077457	-0.189758559
C	0.205201646	-1.209117080	-0.982684644
C	0.383689002	-2.590110562	-1.098931103
C	-0.430330403	-3.473153762	-0.381430228
C	-1.413440706	-2.971417186	0.467240680
C	-1.612970236	-1.593770174	0.554737335
H	1.169584486	-2.994692339	-1.724663297
H	-0.278223113	-4.544236393	-0.484514780
H	-2.039541465	-3.644685924	1.046795613
H	-2.385449238	-1.212077997	1.212496953
O	1.042839831	-0.277297080	-1.543946325
C	2.151278756	-0.710523738	-2.352493145
C	3.184039306	0.406903209	-2.309247529
C	1.663319074	-1.018552449	-3.768233500
H	2.599361107	-1.604765079	-1.898707039
H	3.550782526	0.560317524	-1.290756749
H	4.040787864	0.148947355	-2.939946438
H	2.750022341	1.339589204	-2.675797420
H	0.890724479	-1.792493955	-3.759174522
H	1.232286920	-0.114940700	-4.209712666
H	2.490141967	-1.359473737	-4.400335531
C	-1.474238061	3.687811375	0.573600374
N	-1.561252542	4.269072777	-0.672160090
C	-1.525858053	5.741523720	-0.639615462
C	-1.846837447	6.041303833	0.829212093
N	-1.526899960	4.755833802	1.456717288
H	-0.528968585	6.104793627	-0.929377789
H	-2.256454083	6.166240459	-1.334918287
H	-1.234839774	6.847281427	1.247727996
H	-2.904443862	6.309126133	0.968489602
C	-1.320621917	3.623765539	-1.920354746
C	-2.404235715	3.422280723	-2.794412298
C	-3.797897235	3.877863526	-2.428644438
C	-2.166666671	2.806041207	-4.025832819
C	-0.882148109	2.415741883	-4.413604590
C	-0.652341845	1.704134179	-5.726623579
C	0.180339878	2.666794526	-3.540777376
C	-0.012549220	3.260477474	-2.290385627
C	1.144035131	3.431110235	-1.338101267
H	-3.961300591	4.921602001	-2.727609589
H	-3.969747231	3.821014838	-1.350970929
H	-4.555465560	3.272680159	-2.935585165
H	-3.004781414	2.626666601	-4.696545835
H	-1.382712255	2.006561103	-6.483969049
H	-0.744723953	0.618103944	-5.599967322
H	0.349709454	1.900575369	-6.121600852
H	1.187225296	2.387102778	-3.839864508
H	1.046941042	4.329642700	-0.722637000
H	1.169505013	2.576248533	-0.652725302
H	2.095391437	3.477360957	-1.875544359
C	-1.570445016	4.570846064	2.870128805
C	-0.351960816	4.515382433	3.574972762
C	0.958536537	4.596854359	2.832650138
C	-0.390818699	4.391162307	4.965321961
C	-1.600152997	4.303751282	5.664527745
C	-1.624775570	4.172243985	7.170419331
C	-2.792606742	4.359208723	4.940292280
C	-2.802872257	4.504062466	3.549185269
C	-4.115693964	4.636641065	2.816721304
H	1.805694401	4.509737372	3.517199851
H	1.017788112	3.788325900	2.096247977
H	1.048566333	5.543061666	2.286294848
H	0.548026299	4.369655362	5.514305535
H	-0.621317550	4.016019292	7.579308406
H	-2.033652099	5.074482120	7.641306645
H	-2.254011966	3.332544391	7.486316234
H	-3.741609001	4.294228202	5.469427247
H	-4.484561184	5.669773098	2.861319057
H	-4.018857791	4.361229745	1.765718577
H	-4.881059938	3.997861621	3.267815462
S	5.137402477	-0.198704731	1.243231186
C	3.816271797	-1.214864204	1.520371432
N	3.836071592	-2.569140691	1.691568506
C	2.559246687	-3.073016583	1.926691156
C	1.712834350	-2.019816872	1.892345007
N	2.483469689	-0.887499029	1.632966361
H	4.698197082	-3.089689107	1.657937239
H	2.367087606	-4.123872343	2.066442965
H	0.640290549	-1.956001073	1.988005565
C	1.975601615	0.466127231	1.601942904
H	1.004310512	0.501342054	1.108541300
H	2.690694781	1.079202166	1.054211881
S	-0.870442905	0.370880400	3.346086167
C	0.684898479	0.981669344	3.698827564
N	1.834412457	1.005226661	2.944995754
C	2.883284444	1.560957473	3.673878753
C	2.392855170	1.897996629	4.890043596
N	1.053760410	1.529075686	4.890918407
H	3.873113607	1.624592937	3.247917329
H	2.867802479	2.354151359	5.743052430
H	0.367541611	1.739806001	5.599153909
C	-3.204899986	1.048791095	0.057544184
C	-3.560921300	1.378647157	1.415957047
H	-3.330993621	0.020485049	-0.274779139
H	-3.419612897	1.771840076	-0.721174648
H	-3.825425488	0.563450328	2.093918264
H	-4.167820547	2.267371387	1.570024799

5d$_r$‡$_{trans}$ (289.84*i* cm^{-1})

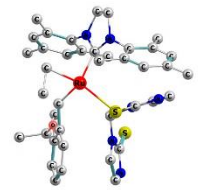

100

RuC40N6H50S2O

Ru	-1.866296021	1.578440901	1.027696320
C	-1.374129417	0.148253488	-0.148881197
H	-1.438305313	0.304235308	-1.231575944
C	-0.817459569	-1.185563711	0.167541124
C	0.224697957	-1.755093641	-0.610764512
C	0.608640119	-3.088388702	-0.424157233
C	0.001578997	-3.860898226	0.567482405
C	-0.983682977	-3.306649559	1.382624177
C	-1.381806040	-1.986736520	1.174143012
H	1.391304979	-3.528231704	-1.029801211
H	0.310958819	-4.894572256	0.700075904
H	-1.455489523	-3.900145393	2.160813530
H	-2.170849869	-1.548965598	1.775864754
O	0.830284422	-0.896877355	-1.485841804
C	1.833981040	-1.383060716	-2.399536678
C	2.610917519	-0.156711046	-2.852275700
C	1.165795820	-2.120582960	-3.560099634
H	2.516937136	-2.052724005	-1.859261500
H	3.143390701	0.299021602	-2.013068394
H	3.347691955	-0.435862334	-3.612606139
H	1.922313452	0.576203228	-3.276107942
H	0.552829397	-2.953909819	-3.205654148
H	0.517138768	-1.429216213	-4.108205919
H	1.919145153	-2.514331799	-4.250710233
C	-1.436281822	3.540547158	0.897711827
N	-1.162173460	4.298709134	-0.213410930
C	-1.289962508	5.748155116	0.003087063
C	-2.122266308	5.788617047	1.283815251
N	-1.851765213	4.457485145	1.857541422
H	-0.300855395	6.213688168	0.129169279
H	-1.787977036	6.224398766	-0.846164340
H	-1.833253624	6.588452315	1.968919641
H	-3.194828728	5.895307676	1.067453025
C	-0.589077553	3.825365163	-1.430136699
C	-1.384311560	3.723784133	-2.583175218
C	-2.837168184	4.135006916	-2.577762086
C	-0.781447419	3.296454976	-3.770377896
C	0.582779803	3.005006789	-3.842003773
C	1.196624748	2.525400578	-5.137272154
C	1.358534767	3.164687464	-2.688413021
C	0.793793259	3.575318164	-1.479949784
C	1.648986768	3.799235720	-0.257282419
H	-2.945171775	5.159299446	-2.958640591
H	-3.259794623	4.098940814	-1.573169777
H	-3.434869803	3.485846418	-3.225049694
H	-1.394008077	3.201194079	-4.665431608
H	0.850887708	3.121362014	-5.989104406
H	0.921493351	1.482989183	-5.340642443
H	2.289021485	2.575397874	-5.107983725
H	2.427442401	2.968695023	-2.730603387
H	1.712293949	4.866335960	-0.007987401
H	1.222405617	3.294977131	0.612017763
H	2.670205538	3.439070609	-0.413928390
C	-1.836613596	4.277130217	3.273381357
C	-0.769578273	4.828330601	4.008160217
C	0.395002485	5.491375766	3.310042254
C	-0.777862451	4.705858475	5.403288927
C	-1.806573830	4.037205128	6.076509592
C	-1.815957564	3.942416904	7.584865436
C	-2.837308486	3.475753752	5.317468116
C	-2.873384967	3.585998171	3.924794973
C	-3.957970258	2.916570522	3.126440887
H	1.234567674	5.630714687	3.997000683
H	0.741976898	4.884551492	2.468733794
H	0.135738561	6.479598094	2.911491077
H	0.037460666	5.145461107	5.976653751
H	-0.814039009	4.080769720	8.003881300
H	-2.462383128	4.713258046	8.023683873
H	-2.193990893	2.971881949	7.922111572
H	-3.632825097	2.931458675	5.821749876
H	-4.803395233	2.629465871	3.758025804
H	-4.317459335	3.550330885	2.311486195
H	-3.566623778	1.989777150	2.663491485
S	5.099085574	0.317992136	0.322908650
C	4.012667122	-0.723913661	1.091661481
N	4.274594546	-1.965585573	1.595427277
C	3.150165359	-2.516918913	2.204717131
C	2.152976581	-1.614324896	2.069251764
N	2.680015037	-0.526980800	1.375271706
H	5.193649293	-2.374328597	1.533433249
H	3.153868817	-3.499681522	2.646453746
H	1.114184878	-1.638598971	2.358647717
C	1.974929817	0.710006198	1.115402449
H	0.953702760	0.514675673	0.797084103
H	2.514464368	1.234966197	0.330594362
S	-0.616967266	0.785777011	3.118754250
C	0.884718558	1.571536651	3.198188267
N	1.931491023	1.540408239	2.306629802
C	2.995242533	2.310782556	2.774647231
C	2.605711284	2.850022787	3.954146893
N	1.319663928	2.385718895	4.202426300
H	3.919221877	2.368374892	2.219562084
H	3.121489366	3.510275394	4.632131332
H	0.689744310	2.706680428	4.925995642
C	-3.558246179	1.650348367	-0.274444298
C	-3.500341112	0.242278718	0.093015351
H	-3.403253632	1.870334961	-1.330006981
H	-4.355654803	2.252884670	0.168476447
H	-3.464629264	-0.524968643	-0.676139533
H	-4.055860856	-0.109218697	0.961391870

$6d_r$

100

RuC40N6H50S2O

Ru	-1.752940419	1.468024478	1.131127783
C	-1.605369321	0.543290405	-0.761509673
H	-0.839882719	0.891347412	-1.449992956
C	-1.334608849	-0.802938923	-0.216144331
C	-0.089391021	-1.466899824	-0.413492185
C	0.153135150	-2.722674526	0.137741402
C	-0.807861203	-3.343461433	0.946486713
C	-2.024746148	-2.714405406	1.174427839
C	-2.287494810	-1.475088576	0.582104716
H	1.098902821	-3.219847880	-0.044127792
H	-0.596590416	-4.318545925	1.377451871
H	-2.785180722	-3.191637454	1.786460191
H	-3.271174076	-1.035869019	0.710482527
O	0.845569546	-0.779836949	-1.151881921
C	1.874681430	-1.488654768	-1.869251283
C	2.770538379	-0.407642940	-2.459623755
C	1.265240582	-2.383916169	-2.948806903
H	2.462546514	-2.092541699	-1.161982135
H	3.271138017	0.169277643	-1.678488773
H	3.545541062	-0.859202722	-3.086725825
H	2.169583908	0.272062866	-3.071096725
H	0.584500513	-3.124603421	-2.522121423
H	0.701793469	-1.770396154	-3.659526759
H	2.054895847	-2.913310731	-3.492385803
C	-1.662980574	3.370393298	0.864891456
N	-1.577292273	4.119152058	-0.301504925
C	-1.660116544	5.568397888	-0.065622237
C	-2.278265088	5.626365110	1.331222158
N	-1.931098675	4.303684342	1.861557629
H	-0.658678027	6.024285024	-0.092891946
H	-2.275968043	6.055036378	-0.827436342
H	-1.868611760	6.427888493	1.952616554
H	-3.370079426	5.752876134	1.289924068
C	-0.957494824	3.685308882	-1.514319600
C	-1.710128114	3.634722886	-2.697302573
C	-3.160570062	4.052922714	-2.722540010
C	-1.081292896	3.205283898	-3.873014011
C	0.267620812	2.850951509	-3.900198979
C	0.916602208	2.324517674	-5.158430696
C	1.010402388	2.977453578	-2.720019193
C	0.423517228	3.402179157	-1.529400747
C	1.253924303	3.575964829	-0.284500920
H	-3.252143989	5.129811764	-2.916384299
H	-3.649471272	3.843619216	-1.769110040
H	-3.706786822	3.533345430	-3.515208104
H	-1.668946857	3.136633082	-4.786358270
H	0.339455085	2.583997114	-6.050980948
H	0.997992333	1.230647209	-5.125419606
H	1.931372100	2.718116683	-5.282147125
H	2.072946177	2.743667038	-2.729882531
H	1.185818526	4.599705146	0.100829659
H	0.892197921	2.916125257	0.508264675
H	2.309016273	3.366606771	-0.481753907
C	-1.899614156	4.080286154	3.271553976
C	-0.746806019	4.482624646	3.970415305
C	0.436953027	5.055101470	3.229975154
C	-0.706445787	4.316734173	5.359591014
C	-1.774946811	3.747704037	6.060004032
C	-1.722369107	3.576591227	7.560413614
C	-2.909999714	3.365248276	5.339298843
C	-3.002043670	3.535794274	3.954670581
C	-4.246229669	3.127989764	3.208507559
H	1.267161213	5.262309525	3.910454484
H	0.789077698	4.356507783	2.466661098
H	0.188612931	5.990431369	2.716569942
H	0.172833106	4.654377093	5.908276468
H	-0.711606421	3.733978174	7.949459260
H	-2.384524508	4.291853696	8.063522728
H	-2.046216452	2.573248241	7.857161657
H	-3.752995512	2.928712903	5.870928899
H	-5.066917925	2.912754042	3.898572578
H	-4.568734891	3.909770470	2.513408104
H	-4.052691232	2.238616816	2.599238327
S	5.508965577	0.420085042	0.383325615
C	4.406896239	-0.662235954	1.060777488
N	4.655365553	-1.932567358	1.497476700
C	3.518259457	-2.515493219	2.049615120
C	2.524291762	-1.603541358	1.945890790
N	3.065125244	-0.480799068	1.321251230
H	5.575204572	-2.339375142	1.432411914
H	3.518695007	-3.514544300	2.453701413
H	1.485172007	-1.632511935	2.232422911
C	2.363003292	0.766089525	1.101775716
H	1.351531758	0.570991111	0.748656047
H	2.920213579	1.326835203	0.354649727
S	-0.250070032	0.709154481	3.047561321
C	1.236377553	1.512375158	3.201422927
N	2.293271278	1.545711817	2.323620905
C	3.336354196	2.309573414	2.845214428
C	2.922643635	2.776810614	4.047280438
N	1.644024012	2.274840888	4.255397877
H	4.267413348	2.410366530	2.308530591
H	3.417618868	3.410184589	4.765215499
H	0.997975967	2.537321975	4.988007662
C	-3.591173684	1.512215279	0.100975652
C	-3.055706578	0.836769763	-1.174726362
H	-4.056131675	2.488935972	-0.070721396
H	-4.313710092	0.889935309	0.654066927
H	-3.064786879	1.509923494	-2.034394642
H	-3.609747045	-0.066913844	-1.475111702

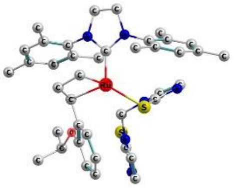

1e

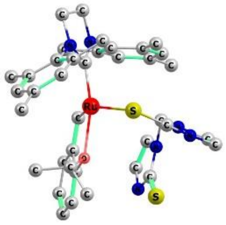

94

RuC38N6H46S2O

Ru	-0.563052967	1.691675094	1.206284786
C	-0.769056420	1.006032804	-0.533837696
H	-0.706195362	1.515681872	-1.501346473
C	-0.892853668	-0.442991015	-0.683571128
C	-0.912542630	-1.259649199	0.473909584
C	-0.990137530	-2.646331890	0.395879723
C	-1.101925400	-3.258093170	-0.858491948
C	-1.118948493	-2.480278270	-2.020577873
C	-1.004223672	-1.094408537	-1.928555145
H	-0.946004557	-3.246504092	1.294443056
H	-1.180529293	-4.340527985	-0.919628751
H	-1.216368353	-2.956516178	-2.993306585
H	-0.999174145	-0.483977059	-2.828813478
O	-0.779783291	-0.556398215	1.653477258
C	-1.580002901	-0.861248683	2.843935651
C	-0.923452432	-1.940140996	3.698879843
C	-3.039774584	-1.113185588	2.486944176
H	-1.516022491	0.085379367	3.387712373
H	0.152170165	-1.758934792	3.752758786
H	-1.347045959	-1.903259551	4.709243955
H	-1.087367603	-2.947535330	3.306014062
H	-3.413486314	-0.291841987	1.869169997
H	-3.175819647	-2.054884665	1.948242713
H	-3.631804424	-1.153895796	3.407603724
C	-0.862926481	3.551013766	0.727638984
N	-0.497131569	4.367238170	-0.325750814
C	-0.965080619	5.754893623	-0.179887674
C	-2.046594004	5.622858050	0.903317340
N	-1.701628923	4.333955088	1.503085574
H	-0.142526406	6.409769613	0.142818468
H	-1.354307343	6.145665271	-1.125886864
H	-2.011452171	6.432947340	1.639985061
H	-3.057417868	5.602601153	0.469959899
C	0.578510760	4.087960584	-1.217241295
C	0.283167229	3.788179437	-2.557549808
C	-1.148725531	3.758516864	-3.037126625
C	1.339341943	3.519558952	-3.435586385
C	2.671234099	3.565035885	-3.016372949
C	3.802342600	3.221509148	-3.959237640
C	2.936315088	3.907295978	-1.684646052
C	1.911265931	4.172898312	-0.771943316
C	2.224563953	4.529682415	0.660574521
H	-1.544027112	4.774826197	-3.164378553
H	-1.793169519	3.250023041	-2.315327681
H	-1.230620469	3.250683817	-4.002484398
H	1.114070369	3.267080183	-4.469836737
H	3.492510116	3.298822812	-5.005561793
H	4.154201490	2.193857366	-3.796804531
H	4.663474556	3.881852013	-3.810556702
H	3.970696838	3.973120181	-1.349674293
H	1.860801757	5.532405320	0.912367166
H	1.736696203	3.834144188	1.351361794
H	3.303246542	4.511800727	0.843578743
C	-2.438622225	3.756654569	2.583100505
C	-1.969156806	3.961974542	3.891671148
C	-0.768881689	4.843431981	4.139751774
C	-2.635967904	3.332644722	4.949475202
C	-3.755094745	2.526689143	4.730865848
C	-4.431489963	1.801458509	5.871176091
C	-4.229809381	2.381520034	3.421275018
C	-3.593251177	2.984163960	2.335125805
C	-4.105955490	2.782357314	0.929949802
H	-0.468916944	4.810536097	5.190666684
H	0.081282408	4.525312518	3.530892803
H	-0.985656838	5.887874949	3.882852531
H	-2.266116607	3.470773279	5.963270800
H	-4.146430353	0.741605885	5.883374226
H	-4.156299694	2.226749060	6.840941079
H	-5.522531509	1.839667751	5.780175206
H	-5.111709011	1.770101475	3.240168451
H	-4.301816284	3.736387359	0.427498339
H	-3.354424668	2.242990531	0.341787991
H	-5.035983393	2.206472618	0.932708093
S	2.477922712	-2.061551198	1.767877232
C	2.424493911	-1.348920646	0.241664207
N	2.155497092	-2.023320929	-0.923153510
C	2.194644151	-1.188325615	-2.024856541
C	2.531072979	0.037878841	-1.568780219
N	2.690039621	-0.058557520	-0.182172996
H	1.785607924	-2.961817410	-0.898974961
H	1.968603216	-1.533975319	-3.019419033
H	2.622119337	0.985301982	-2.076424873
C	2.696088391	1.181279059	0.592698740
H	1.644530368	1.554899412	0.663407951
H	3.302839934	1.898251919	0.037548454
S	0.928582241	1.790668643	3.370499732
C	2.498576628	1.210197119	3.080271272
N	3.218182996	1.037191557	1.924878740
C	4.509337008	0.606933535	2.220954001
C	4.607841487	0.511514546	3.564070277
N	3.373323495	0.884877889	4.074032733
H	5.208273755	0.362601911	1.438377605
H	5.422337160	0.194588147	4.194149376
H	3.103368844	0.912410189	5.044567093

2e

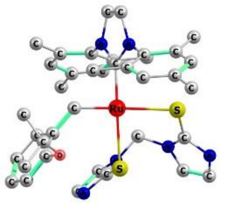

94

RuC38N6H46S2O

Ru	-0.059110984	1.574531084	1.546102618
C	-0.826241288	0.795304772	0.015875626
H	-1.447043868	1.384018949	-0.669701387
C	-0.636038028	-0.550655598	-0.573656888
C	-1.108462105	-1.736508094	0.034870696
C	-0.851458509	-2.989312092	-0.537803397
C	-0.173959853	-3.092840285	-1.756443378
C	0.254762816	-1.927884133	-2.398934423
C	0.027312336	-0.684807314	-1.810308924
H	-1.199625511	-3.874789175	-0.014683486
H	0.003909215	-4.069750195	-2.199475898
H	0.775458410	-1.988393148	-3.351948357
H	0.390341289	0.219084349	-2.293741616
O	-1.792602402	-1.664399101	1.224136631
C	-3.238224427	-1.689446035	1.196749075
C	-3.744269182	-3.131302992	1.282876813
C	-3.836425763	-0.938963938	0.007222757
H	-3.508048868	-1.160647406	2.117837591
H	-3.260800381	-3.651763833	2.115002347
H	-4.828948247	-3.152269605	1.439884539
H	-3.526443171	-3.675794932	0.357819717
H	-3.436539080	0.075010559	-0.053535716
H	-3.617228929	-1.452399372	-0.934401213
H	-4.924373097	-0.881523037	0.120860453
C	-0.894759802	3.274742306	1.017276434
N	-0.849220812	3.918414554	-0.213018405
C	-1.484405638	5.245896861	-0.201436495
C	-2.312753889	5.195441082	1.088409856
N	-1.683310935	4.081853244	1.806747401
H	-0.724080316	6.039331563	-0.168300589
H	-2.093826897	5.401774743	-1.097869564
H	-2.262658954	6.124697087	1.664803569
H	-3.372129403	4.974775010	0.890967354
C	0.118498868	3.629599677	-1.227595274
C	-0.298320809	3.070363862	-2.445829094
C	-1.751756471	2.798439800	-2.757019747
C	0.671434754	2.737381248	-3.402239379
C	2.029415005	2.960243140	-3.182036833
C	3.071163920	2.503581736	-4.176828830
C	2.408625765	3.598596914	-1.994194640
C	1.477187962	3.955062315	-1.018263091
C	1.924478697	4.682483053	0.226510827
H	-2.078704760	3.392126522	-3.619740407
H	-2.397057665	3.032732278	-1.909207836
H	-1.899915591	1.742744756	-3.010701778
H	0.349600924	2.274132678	-4.333394153
H	2.665692895	2.444421270	-5.191699710
H	3.442467435	1.503150851	-3.917245641
H	3.935512219	3.176264096	-4.194825286
H	3.459558966	3.833943238	-1.829736675
H	1.460634971	5.673386312	0.298613738
H	1.650286540	4.135091346	1.132280753
H	3.009420479	4.829911899	0.223768048
C	-2.099781174	3.742954740	3.130279700
C	-1.554383079	4.461099388	4.208886321
C	-0.570125538	5.583716324	3.978486244
C	-1.945027453	4.119219552	5.507899631
C	-2.860788895	3.094474800	5.752186489
C	-3.227651570	2.693339990	7.162291618
C	-3.414799846	2.422952128	4.657096369
C	-3.056052823	2.730469749	3.343720474
C	-3.646947798	1.965828998	2.188143912
H	0.025993944	5.770234436	4.876249339
H	0.112449097	5.349474942	3.158432968
H	-1.085842369	6.520099784	3.725258145
H	-1.510548334	4.658550333	6.347387844
H	-2.655453775	1.812022844	7.478953513
H	-3.019053036	3.494796406	7.877809818
H	-4.288934147	2.433943834	7.242626739
H	-4.143063460	1.632746044	4.830396775
H	-3.952052176	2.630592295	1.372346323
H	-2.884353766	1.293360345	1.779133864
H	-4.517454846	1.382643293	2.503589348
S	1.135543172	-0.675535331	2.258781168
C	2.177741120	-0.842896630	0.937359364
N	2.342584453	-1.952419203	0.159608286
C	3.248707783	-1.720177211	-0.861791006
C	3.705344940	-0.455312019	-0.701480235
N	3.054073142	0.079455637	0.409230355
H	1.664847536	-2.704217749	0.184392292
H	3.481347710	-2.465217211	-1.604350795
H	4.418596048	0.119067113	-1.270729237
C	3.097243384	1.455970731	0.845783610
H	2.036093020	1.833112886	0.841207593
H	3.716324360	2.024345351	0.150412330
S	1.408760986	2.717964451	3.446627442
C	2.939242120	2.006447238	3.304538911
N	3.624983347	1.559889211	2.198441806
C	4.872633754	1.066428332	2.575931883
C	4.987015525	1.220604212	3.914520148
N	3.805261006	1.807008550	4.340982850
H	5.546841931	0.633505150	1.854960079
H	5.788960186	0.972001085	4.590520473
H	3.538798389	2.011084475	5.291718003

3e’

94
RuC38N6H46S2O

Ru	-0.102602435	1.697571620	1.498295348
C	-0.876045534	1.017319879	-0.078185750
H	-1.802081622	1.428066590	-0.505886628
C	-0.395079393	-0.084613802	-0.931177717
C	-1.221279926	-1.179104822	-1.281354753
C	-0.764314327	-2.186452528	-2.133144663
C	0.514890585	-2.121457588	-2.688287492
C	1.340613869	-1.040149521	-2.374932908
C	0.889536715	-0.048434796	-1.504340837
H	-1.439854930	-3.007124860	-2.357616187
H	0.856662472	-2.904000465	-3.361384546
H	2.335550122	-0.966722321	-2.807940432
H	1.506884171	0.817452012	-1.289006810
O	-2.524817971	-1.264225659	-0.842476985
C	-2.727877578	-1.460561040	0.579919904
C	-2.311258027	-2.874304573	0.976705957
C	-4.203680811	-1.188190735	0.823923502
H	-2.120653544	-0.731351119	1.127688165
H	-1.257615183	-3.038942145	0.731914098
H	-2.433154268	-3.022165025	2.054629570
H	-2.914500256	-3.618409345	0.443590324
H	-4.454540519	-0.169937804	0.515225189
H	-4.822204395	-1.888397996	0.251459295
H	-4.441170815	-1.298935400	1.887006345
C	-0.881326605	3.463574686	1.006720228
N	-0.832575844	4.106612266	-0.211930877
C	-1.396815036	5.463988232	-0.186707816
C	-2.190318477	5.453646980	1.125637210
N	-1.603321890	4.301293282	1.823374926
H	-0.593448095	6.214728392	-0.177149096
H	-2.021017178	5.649160453	-1.066884241
H	-2.071445885	6.373894032	1.705919849
H	-3.265304643	5.292758662	0.958579545
C	-0.001943489	3.714181545	-1.307496419
C	-0.593534814	3.184021395	-2.465787408
C	-2.089187914	3.026480813	-2.608181476
C	0.237118195	2.762233280	-3.509531522
C	1.625235026	2.863127817	-3.432701419
C	2.499990578	2.291019277	-4.522710913
C	2.184063911	3.454952978	-2.294200683
C	1.394135018	3.895754143	-1.230811589
C	2.029676412	4.533327775	-0.020213247
H	-2.471676329	3.660593515	-3.417786696
H	-2.612950034	3.286338996	-1.687092408
H	-2.341638638	1.989777438	-2.854863189
H	-0.217035109	2.310265472	-4.389309073
H	2.093920922	2.495371462	-5.519166383
H	2.567428747	1.200015308	-4.419691681
H	3.517034115	2.694059264	-4.481120643
H	3.264946216	3.573305355	-2.231660001
H	1.601605982	5.520433359	0.189048827
H	1.862517702	3.923853383	0.872991323
H	3.106752428	4.662613379	-0.166696418
C	-2.047093034	3.955155459	3.135702284
C	-1.482023062	4.621992376	4.235236868
C	-0.443471763	5.701564434	4.042404649
C	-1.905557178	4.270840111	5.521394302
C	-2.874769046	3.288924533	5.731067023
C	-3.281122977	2.878417641	7.127719383
C	-3.448867826	2.671348202	4.614131221
C	-3.057820995	2.989351600	3.312740366
C	-3.682136040	2.290506321	2.131539548
H	0.174513472	5.813424114	4.937892504
H	0.213742070	5.474033758	3.200246892
H	-0.915035113	6.673496283	3.844903084
H	-1.456286754	4.769991676	6.377952449
H	-2.756907633	1.964570592	7.435232391
H	-3.044745249	3.655166837	7.861711271
H	-4.354747909	2.669594081	7.188470637
H	-4.220705817	1.918006023	4.760828553
H	-3.953751295	2.995710139	1.338091524
H	-2.958571747	1.590576341	1.698636029
H	-4.580727619	1.741832022	2.427945474
S	0.528342541	-0.669461698	2.333847638
C	1.808503570	-1.184180885	1.360814091
N	2.026078318	-2.447802627	0.886909419
C	3.184813393	-2.514293649	0.130876351
C	3.725878752	-1.273870175	0.141971966
N	2.879542546	-0.457587142	0.890493507
H	1.341083202	-3.178161380	1.004234070
H	3.509087853	-3.420977740	-0.352889850
H	4.608488299	-0.885106637	-0.340048195
C	3.033265901	0.961026466	1.110059948
H	2.027080252	1.454439726	0.959040309
H	3.764717586	1.345215545	0.395922629
S	1.314994503	2.750009529	3.382510896
C	2.785937376	1.910373978	3.442165285
N	3.481383668	1.228966522	2.470430294
C	4.671489208	0.725682198	2.996447190
C	4.736775517	1.099755628	4.294178030
N	3.584677198	1.827162274	4.547876490
H	5.343987625	0.131966236	2.398852772
H	5.486114558	0.916321352	5.047079993
H	3.299467835	2.223776669	5.429972713

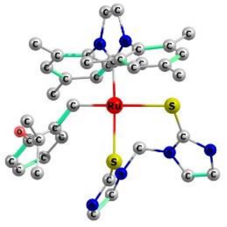

3e

94
RuC38N6H46S2O

Ru	-0.088904041	1.628927561	1.701682291
C	-1.158047455	0.768524031	0.383777233
H	-1.599515031	1.320298981	-0.455803762
C	-1.392968469	-0.680104602	0.192921979
C	-1.155468390	-1.347872937	-1.039126203
C	-1.408762238	-2.718323503	-1.180846247
C	-1.890100282	-3.462467472	-0.097349192
C	-2.128735410	-2.832405730	1.123697398
C	-1.881664959	-1.463625177	1.252876426
H	-1.228977737	-3.223494904	-2.122175004
H	-2.087537275	-4.524024464	-0.223965058
H	-2.512374496	-3.396747118	1.969522181
H	-2.052941665	-0.956530168	2.196139739
O	-0.602982891	-0.573830874	-2.034059101
C	-0.312429126	-1.152624049	-3.313135150
C	0.709063128	-0.239436251	-3.981711629
C	-1.588072923	-1.289009205	-4.146247214
H	0.153737984	-2.136080323	-3.159737015
H	1.559000442	-0.065933982	-3.317266578
H	1.068998789	-0.692000483	-4.911612430
H	0.263484507	0.729013567	-4.219825921
H	-2.331732537	-1.910681643	-3.642311379
H	-2.026822836	-0.298932962	-4.306456625
H	-1.366638837	-1.733767543	-5.122375455
C	-0.814927723	3.358937622	1.100708642
N	-0.471839846	4.119309678	-0.002533294
C	-1.144656561	5.429101902	-0.048365327
C	-2.246877356	5.269810972	1.012662320
N	-1.819271309	4.055863763	1.718971879
H	-0.445019226	6.235991145	0.206954876
H	-1.540984601	5.632456167	-1.049252274
H	-2.308951775	6.125275009	1.694338233
H	-3.238640933	5.127882423	0.561921683
C	0.726891071	3.913542763	-0.755759663
C	0.654984802	3.265253042	-1.998030667
C	-0.664789643	2.823902301	-2.578571105
C	1.844560782	3.027122777	-2.700441067
C	3.087633549	3.418683904	-2.200073577
C	4.368459740	3.094406636	-2.935577630
C	3.122937116	4.125532575	-0.991830022
C	1.958604763	4.410932757	-0.275821974
C	2.032542027	5.307353196	0.936658010
H	-0.664786312	2.940768581	-3.667992603
H	-1.497503264	3.397210114	-2.166401792
H	-0.848540571	1.771996538	-2.345153029
H	1.794345879	2.523116411	-3.662785413
H	4.169933418	2.685825782	-3.931134048
H	4.964145589	2.354150129	-2.384730002
H	5.000084336	3.982059245	-3.055293100
H	4.077824331	4.479371714	-0.606815424
H	1.949206093	6.358357279	0.628192566
H	1.240023556	5.094911767	1.652021169
H	2.985070659	5.188806831	1.458045137
C	-2.476556524	3.623986389	2.913478597
C	-1.978552122	4.071357367	4.148847634
C	-0.759003829	4.959300671	4.208854164
C	-2.623285668	3.662207496	5.318553235
C	-3.747468927	2.832401183	5.283063883
C	-4.400628164	2.361449204	6.562481014
C	-4.235713517	2.427612075	4.036851955
C	-3.618310849	2.809242560	2.842593421
C	-4.123157739	2.307802626	1.515336658
H	-0.519295543	5.228590239	5.241399577
H	0.113399099	4.459295591	3.778904744
H	-0.903933925	5.887778384	3.643771903
H	-2.236131953	3.996278384	6.278862575
H	-3.948196321	1.424317623	6.911527907
H	-4.286307765	3.096168087	7.366505315
H	-5.470352431	2.173869795	6.423133058
H	-5.115009390	1.787596749	3.992454992
H	-4.280299010	3.121276912	0.798506809
H	-3.377218227	1.632334541	1.083475329
H	-5.067694166	1.768413653	1.630008777
S	0.950570718	-0.644810910	2.588698687
C	1.531698128	-1.258065474	1.124792770
N	1.352166343	-2.526106810	0.650071371
C	1.922973450	-2.677689131	-0.603257951
C	2.512043815	-1.496253487	-0.907580473
N	2.275755294	-0.624677899	0.153297059
H	0.646056178	-3.128575720	1.053154555
H	1.865353512	-3.600766779	-1.155488865
H	3.077482507	-1.191528614	-1.773241220
C	2.564262731	0.795568603	0.161429674
H	1.604486405	1.337070748	0.418148171
H	2.911661459	1.090666938	-0.828709963
S	2.016640012	2.623366071	2.954942233
C	3.374157949	1.847967802	2.318658166
N	3.568484751	1.136564637	1.154574373
C	4.899394351	0.732786820	1.058108985
C	5.552303722	1.193686530	2.148320065
N	4.611831064	1.876169107	2.904068098
H	5.245114282	0.144866696	0.223385838
H	6.582870807	1.101092495	2.450053596
H	4.756146067	2.310014457	3.802292460

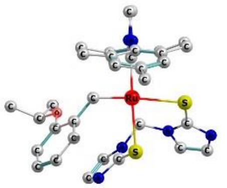

4e$_{cis}$

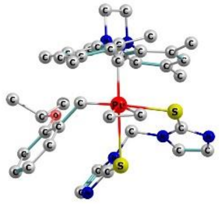

100
RuC40N6H50S2O

Ru	0.016462091	1.559279531	2.036015081
C	-1.014417718	0.728347213	0.618083628
H	-1.579133229	1.318077992	-0.114493521
C	-1.296865574	-0.704011878	0.387216004
C	-1.120555463	-1.330227886	-0.877808791
C	-1.435213224	-2.678435865	-1.076325504
C	-1.907736072	-3.460344066	-0.015645350
C	-2.067999711	-2.879893113	1.242095415
C	-1.770384648	-1.528394177	1.426082704
H	-1.309399436	-3.138339562	-2.049680784
H	-2.156497567	-4.504522408	-0.187307477
H	-2.444580695	-3.467523216	2.075883713
H	-1.904285272	-1.067765798	2.396992683
O	-0.554395994	-0.535331545	-1.860241322
C	-0.474194708	-1.016894937	-3.208495040
C	0.543568019	-0.141932023	-3.934426925
C	-1.847961833	-0.968376418	-3.879180191
H	-0.090219982	-2.046795649	-3.199563617
H	1.494986739	-0.122442684	-3.395540865
H	0.722185667	-0.530815086	-4.942362487
H	0.184620481	0.885243496	-4.021680989
H	-2.576143961	-1.573744562	-3.334214857
H	-2.209137516	0.065043222	-3.896681987
H	-1.790577330	-1.335593962	-4.909559267
C	-0.779022752	3.292574212	1.417996259
N	-0.286993776	4.106443449	0.409424955
C	-1.034368042	5.360132088	0.226476200
C	-2.336265768	5.056337630	0.960535188
N	-1.959825825	3.895941839	1.782710677
H	-0.490163842	6.204036375	0.673622197
H	-1.176515351	5.573140438	-0.837724814
H	-2.680804960	5.883852198	1.587126008
H	-3.152337203	4.788510345	0.274642642
C	0.899576321	3.943742552	-0.366136375
C	0.816317704	3.312239292	-1.618233840
C	-0.496721293	2.808303241	-2.158477308
C	1.984225706	3.176332032	-2.380373818
C	3.210630546	3.679102553	-1.940669469
C	4.478882893	3.463635498	-2.733669393
C	3.240091039	4.394169818	-0.738738271
C	2.098091149	4.560166363	0.047387089
C	2.143392836	5.453781844	1.264048870
H	-0.536875325	2.938728490	-3.245418305
H	-1.344909249	3.335989554	-1.717617841
H	-0.627366252	1.748737330	-1.929563965
H	1.926221535	2.677724208	-3.346288714
H	4.267441956	3.131964715	-3.755134020
H	5.106446294	2.700549567	-2.255877088
H	5.077188740	4.379744168	-2.792593297
H	4.176393536	4.838688727	-0.405846031
H	1.957687229	6.495825840	0.969582638
H	1.395528063	5.166848084	2.004027252
H	3.122798472	5.412977489	1.746617955
C	-2.779282806	3.604046773	2.912622496
C	-2.500557718	4.269519950	4.118794468
C	-1.339367847	5.232613445	4.212204626
C	-3.321092980	4.021959437	5.221032065
C	-4.412873182	3.151942618	5.139707113
C	-5.254547681	2.859157265	6.360207597
C	-4.684899636	2.535838244	3.914506319
C	-3.881967660	2.744905370	2.788505474
C	-4.139473230	2.000369126	1.505317338
H	-1.145392088	5.513366533	5.251162500
H	-0.427407525	4.793758060	3.797659204
H	-1.537504074	6.155375172	3.651321996
H	-3.100848389	4.515493583	6.165037848
H	-4.818867709	2.039703178	6.946056390
H	-5.321397515	3.730023499	7.020816528
H	-6.271372575	2.560507705	6.086350852
H	-5.534145949	1.860124488	3.835143452
H	-4.088058443	2.657781631	0.631839416
H	-3.367128088	1.236576985	1.367193048
H	-5.120641618	1.517104761	1.517240861
S	1.281123396	-0.821715443	2.636789161
C	1.608794032	-1.501385632	1.125642165
N	1.406275823	-2.808567924	0.784821931
C	1.774991494	-3.053564345	-0.527859658
C	2.251339208	-1.886350817	-1.018020671
N	2.146316872	-0.931609607	-0.007744895
H	0.815010697	-3.399577698	1.352280647
H	1.665742652	-4.021345593	-0.988183168
H	2.639702720	-1.637206673	-1.991663809
C	2.365679704	0.491642234	-0.199639814
H	1.489545399	1.030705545	0.192826181
H	2.443305045	0.680947083	-1.270499060
S	2.431777681	2.461414158	2.557616167
C	3.617896076	1.804835848	1.558537589
N	3.561444697	0.974638633	0.457218964
C	4.849166395	0.681436839	0.004595922
C	5.723577692	1.326678070	0.807596971
N	4.963107759	2.013681602	1.741535554
H	5.012108176	0.035245138	-0.842850350
H	6.800995371	1.360064982	0.798196038
H	5.305325726	2.560865734	2.515683773
C	-1.067655379	1.095478233	3.926805633
C	0.021271069	1.847145111	4.395346360
H	-2.068902635	1.500689443	3.947150489
H	-1.027172540	0.010879923	4.011643608
H	0.908802427	1.353283357	4.780817808
H	-0.106559187	2.879954299	4.697975812

5e$^{‡}_{cis}$ (331.99i cm^{-1})

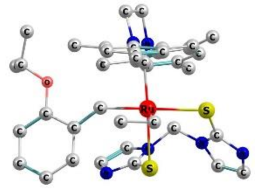

100
RuC40N6H50S2O

Ru	0.260976227	1.395738460	1.957065456
C	-1.261874762	0.204493736	1.286579669
H	-2.094127168	0.804506905	0.930784447
C	-1.545245621	-1.189082520	1.029028180
C	-2.460666732	-1.516218302	-0.035265402
C	-2.777596989	-2.831241155	-0.357992729
C	-2.235269979	-3.901348703	0.372255523
C	-1.374406696	-3.627102134	1.430146572
C	-1.040432585	-2.305722226	1.747765519
H	-3.460114929	-3.044872492	-1.172652596
H	-2.500804049	-4.921855833	0.112295270
H	-0.966665611	-4.437360276	2.030949335
H	-0.377081417	-2.114832662	2.582180157
O	-2.982922130	-0.425325433	-0.695468757
C	-3.961753901	-0.575993423	-1.726541763
C	-3.299186203	-0.941949218	-3.057559406
C	-4.686677037	0.765418425	-1.797551951
H	-4.682029017	-1.354877328	-1.437135910
H	-2.696710321	-1.848630559	-2.963219594
H	-4.055994275	-1.106321170	-3.832414064
H	-2.642087402	-0.128972382	-3.381058828
H	-5.178813454	0.987721785	-0.847031265
H	-3.968082430	1.565432648	-2.007043233
H	-5.439745568	0.757707510	-2.592271599
C	-0.823490438	2.892965418	1.160050799
N	-0.528573258	3.463052170	-0.057141046
C	-1.500558727	4.469044590	-0.515156275
C	-2.683472584	4.201970722	0.413128192
N	-2.065744472	3.398473511	1.487369777
H	-1.091767294	5.482366548	-0.399117803
H	-1.741115279	4.318064371	-1.572139380
H	-3.126026653	5.114730154	0.822316189
H	-3.472637058	3.627029260	-0.087176910
C	0.644520453	3.281569954	-0.852745664
C	0.636515074	2.306950856	-1.874929518
C	-0.512456500	1.346718918	-2.002824553
C	1.723983138	2.250122813	-2.752287427
C	2.806304869	3.131546878	-2.647996530
C	3.993364705	3.018800780	-3.577956134
C	2.778649386	4.095341652	-1.634877387
C	1.704616664	4.203785113	-0.745075419
C	1.672615176	5.325182702	0.265086898
H	-0.377540238	0.679024301	-2.858520810
H	-1.466070776	1.873279914	-2.123217577
H	-0.618953932	0.743251639	-1.092690843
H	1.718395226	1.502890525	-3.544059908
H	3.711146549	2.574000535	-4.537525809
H	4.781770707	2.386589735	-3.146126798
H	4.443144196	3.996902683	-3.777068111
H	3.606958029	4.795682975	-1.543176437
H	1.266697816	6.239652113	-0.188799404
H	1.060567378	5.068116405	1.128969130
H	2.678632243	5.553630910	0.626967105
C	-2.682332387	3.434177357	2.774327823
C	-2.109155333	4.234255625	3.782759739
C	-0.835463053	5.003932613	3.533649307
C	-2.746211894	4.309290751	5.023819910
C	-3.943922736	3.637236470	5.279204745
C	-4.588836518	3.692100673	6.644329167
C	-4.516807408	2.894467591	4.244580629
C	-3.912657914	2.783105499	2.988136327
C	-4.585394188	1.974882823	1.903266912
H	-0.633861693	5.701699066	4.351046076
H	0.019796602	4.329297591	3.437657632
H	-0.893661196	5.576563783	2.600974032
H	-2.296870817	4.914214716	5.808733348
H	-4.238096597	2.864933601	7.274038823
H	-4.346871947	4.623450851	7.166344165
H	-5.678705967	3.611240453	6.578816175
H	-5.457351455	2.376022615	4.420084061
H	-5.115846783	2.623717918	1.194048124
H	-3.866294685	1.383172073	1.331892311
H	-5.324055080	1.290697223	2.330996427
S	1.819953514	-0.646897263	2.542400890
C	2.040901013	-1.080674859	0.911869371
N	1.591754470	-2.210460204	0.290885908
C	1.880323662	-2.171688601	-1.064216281
C	2.594077336	-1.043266336	-1.287630988
N	2.728817403	-0.386660518	-0.062885909
H	0.845931173	-2.762086991	0.716439990
H	1.575958064	-2.957845555	-1.736134364
H	3.022244298	-0.640911506	-2.191528259
C	3.055525890	1.020642768	0.058726973
H	2.124934067	1.566051561	0.300471961
H	3.458680591	1.362350153	-0.892380337
S	2.243043935	2.849762548	2.663658936
C	3.694488800	2.108526658	2.205359790
N	3.984795665	1.306559955	1.127194051
C	5.304080109	0.867317786	1.186723160
C	5.858869093	1.404430768	2.296861694
N	4.870867229	2.171680097	2.900244777
H	5.708017443	0.200147276	0.442446271
H	6.850695290	1.310239658	2.707859372
H	4.931371653	2.642800189	3.788981993
C	-1.296283059	0.510689062	3.401756862
C	-0.313012619	1.377398820	4.006109745
H	-2.321416587	0.860959696	3.363183658
H	-1.214114111	-0.552699955	3.602453011
H	0.462481517	0.914028148	4.618950658
H	-0.646764456	2.332178786	4.400226025

6e*cis*

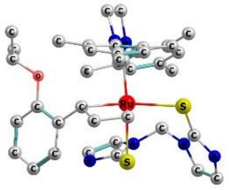

100
RuC40N6H50S2O

Ru	0.209415549	1.437056562	1.851572571
C	-1.586935481	0.201745124	2.117421741
H	-2.409102699	0.807691597	1.759306755
C	-1.665208117	-1.123878626	1.479520191
C	-2.358284434	-1.319620382	0.244414639
C	-2.339593214	-2.550764193	-0.418604982
C	-1.710355739	-3.664684168	0.148205374
C	-1.110487739	-3.533631249	1.400661499
C	-1.096342919	-2.289396256	2.039226410
H	-2.834552896	-2.663095783	-1.375921457
H	-1.721921441	-4.617493467	-0.374286041
H	-0.662341756	-4.394908378	1.891553406
H	-0.606821829	-2.207377899	3.000390871
O	-3.051708664	-0.231528981	-0.249839141
C	-4.113364442	-0.439913256	-1.195767004
C	-3.606084554	-0.349269174	-2.638669378
C	-5.168424974	0.623416906	-0.909960596
H	-4.553064428	-1.431119801	-1.020337998
H	-2.765894360	-1.025289666	-2.813294535
H	-4.409016913	-0.605547598	-3.338825135
H	-3.269613280	0.667792939	-2.861387610
H	-5.575796662	0.504934126	0.096212581
H	-4.725175221	1.621979228	-0.986939360
H	-5.988427965	0.552585764	-1.632494916
C	-0.948830929	2.842774652	1.143971094
N	-0.759927605	3.308778238	-0.157454130
C	-1.748715865	4.296739055	-0.599384686
C	-2.828025247	4.168383138	0.480474803
N	-2.130739835	3.439334710	1.551509877
H	-1.316415345	5.306671638	-0.632113100
H	-2.120256475	4.056684164	-1.602034896
H	-3.187230730	5.136981511	0.842936114
H	-3.694170380	3.597437815	0.121136122
C	0.406244595	3.112108189	-0.956369994
C	0.495154321	1.993733226	-1.808699497
C	-0.526056183	0.892034275	-1.751977420
C	1.575286612	1.907983486	-2.696474434
C	2.580111931	2.877038330	-2.734910711
C	3.762635090	2.737227983	-3.666609221
C	2.490194553	3.956779774	-1.850591022
C	1.417029178	4.099080314	-0.966394269
C	1.352105219	5.297748966	-0.052119829
H	-0.650875653	0.416493918	-2.729293107
H	-1.489132642	1.248652977	-1.392436024
H	-0.221499994	0.115550525	-1.040872047
H	1.622998066	1.059412236	-3.376199889
H	3.512507043	2.136632088	-4.546856336
H	4.610382704	2.246821379	-3.167120973
H	4.120322102	3.712469399	-4.012229763
H	3.272239350	4.713985851	-1.851614461
H	0.864735265	6.147599953	-0.548269828
H	0.799867094	5.068777280	0.859104360
H	2.357117303	5.616303303	0.237487986
C	-2.638210621	3.544162375	2.882995989
C	-1.962790223	4.365895898	3.810352337
C	-0.698859487	5.090520816	3.426661294
C	-2.474814767	4.487017949	5.102365877
C	-3.648436839	3.835820903	5.494957948
C	-4.144990104	3.932887680	6.918604348
C	-4.328787838	3.078795209	4.541761062
C	-3.850675370	2.925768866	3.234803107
C	-4.660485405	2.128517305	2.242377051
H	-0.419263552	5.823337586	4.189056567
H	0.127615276	4.384725670	3.308220412
H	-0.814516201	5.614711773	2.471531465
H	-1.941761466	5.104831405	5.822513361
H	-3.645500687	3.192990143	7.557137588
H	-3.942451830	4.919861173	7.348429580
H	-5.221761723	3.747654844	6.984287644
H	-5.255334802	2.579831554	4.819486036
H	-5.378084285	2.770592886	1.714060525
H	-4.029253210	1.648428025	1.495246089
H	-5.234713607	1.346140937	2.748119262
S	1.778655743	-0.639825543	2.477621346
C	2.014231203	-0.986507738	0.834373194
N	1.483918774	-2.044174133	0.160286776
C	1.864190625	-2.040485886	-1.168246145
C	2.679697819	-0.972758869	-1.338400285
N	2.781436331	-0.325403850	-0.106338163
H	0.761588403	-2.629268515	0.582314232
H	1.528680985	-2.792896524	-1.863150329
H	3.186037092	-0.600333569	-2.213523026
C	3.325100977	1.027522673	0.015342574
H	2.514573479	1.756077099	0.001675962
H	3.965745924	1.190446555	-0.853478472
S	2.198087499	2.909657072	2.435324933
C	3.642235403	2.045301954	2.261830007
N	4.075246564	1.247418291	1.221890220
C	5.302427778	0.664736999	1.530416359
C	5.661333201	1.110320820	2.755091064
N	4.649688415	1.961106663	3.180929545
H	5.790634374	-0.018570560	0.854413700
H	6.534150053	0.903385322	3.352554888
H	4.567243383	2.393745639	4.087571050
C	-1.454602824	0.308481166	3.656581137
C	-0.364252246	1.366868565	3.850273955
H	-2.411581264	0.592063486	4.120724241
H	-1.157222952	-0.645053714	4.107570520
H	0.485852645	1.033953851	4.460577443
H	-0.726153943	2.312357813	4.250586448

7e‡ (282.82*i* cm^{-1})

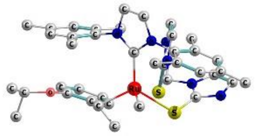

100
RuC40N6H50S2O

Ru	-1.640483736	1.643447762	1.628395408
C	-2.527216422	0.542194513	0.007522542
H	-3.256554459	1.002958199	-0.651605825
C	-1.335661401	0.000071805	-0.689134342
C	-1.256767307	0.020717773	-2.105287745
C	-0.103297112	-0.409892487	-2.763110405
C	0.989893064	-0.892362905	-2.038741255
C	0.920480423	-0.975510136	-0.651505520
C	-0.237248595	-0.548128533	0.005309245
H	-0.049656959	-0.370935521	-3.844875469
H	1.880625560	-1.221540958	-2.567236209
H	1.748696920	-1.395837277	-0.086071350
H	-0.303492820	-0.661318028	1.084327610
O	-2.377393558	0.471224215	-2.756290647
C	-2.588186231	0.162854976	-4.140130096
C	-3.836146051	0.942940091	-4.535189902
C	-2.769860438	-1.341633583	-4.355370307
H	-1.736198184	0.531474603	-4.729829327
H	-3.693504165	2.012023401	-4.362361124
H	-4.069733747	0.783735026	-5.592537458
H	-4.688125743	0.610907896	-3.933485340
H	-1.893245975	-1.906663218	-4.031439556
H	-3.632923220	-1.693702354	-3.781185868
H	-2.944966698	-1.554024551	-5.415331454
C	-1.052953962	3.480036695	1.164404527
N	-0.633582822	4.085680208	-0.005270472
C	-0.388126398	5.529405823	0.130978608
C	-1.131473384	5.856406714	1.427379776
N	-1.190033988	4.536301659	2.060083989
H	0.691970584	5.732896796	0.207693151
H	-0.773389840	6.075049884	-0.736047910
H	-0.600164076	6.573775226	2.060757267
H	-2.139678450	6.251964293	1.229675901
C	-0.345591345	3.495355006	-1.271551871
C	-1.339784749	3.542455848	-2.263803144
C	-2.741282890	3.959724797	-1.894684966
C	-1.002512773	3.175482093	-3.567811006
C	0.283269099	2.739915997	-3.898812035
C	0.634312503	2.338785216	-5.312873042
C	1.238175677	2.658371700	-2.883605685
C	0.945036460	3.029119287	-1.569628584
C	1.990632023	2.873749243	-0.497091153
H	-3.434365996	3.789110557	-2.722606131
H	-2.793298511	5.021873124	-1.623480946
H	-3.079937235	3.386796575	-1.025505132
H	-1.764697234	3.218254365	-4.341818054
H	1.101732407	1.347361999	-5.336265836
H	1.346430735	3.040456640	-5.764522690
H	-0.251714524	2.307263347	-5.954526879
H	2.232703346	2.281057554	-3.114300973
H	1.673543987	3.340863712	0.437287955
H	2.157318218	1.808056403	-0.304900368
H	2.952135929	3.304718996	-0.797027331
C	-1.421143049	4.400077347	3.457342565
C	-0.321371029	4.150797887	4.298094313
C	1.058056832	3.997223516	3.706664885
C	-0.532104705	4.060045168	5.675266950
C	-1.806599529	4.210403598	6.231041090
C	-2.024560579	4.070777241	7.719979362
C	-2.880344329	4.465365673	5.373437695
C	-2.710237373	4.566448182	3.989523841
C	-3.880244430	4.854320099	3.081718791
H	1.391099309	4.925842970	3.226427460
H	1.791716240	3.719446255	4.468815411
H	1.037983550	3.223558200	2.933402071
H	0.319925938	3.881477302	6.329057603
H	-2.403157921	3.071737806	7.971089509
H	-1.095356294	4.222732914	8.278948712
H	-2.760358318	4.793101769	8.088777491
H	-3.878748093	4.581980282	5.791195959
H	-3.753607282	4.349127148	2.121526539
H	-4.818884902	4.517143108	3.531440038
H	-3.975680778	5.930954339	2.889852952
S	4.354490075	-1.758693315	1.559102492
C	4.442184977	-0.087555663	1.610131728
N	5.515144413	0.700713510	1.282503733
C	5.238616073	2.050945455	1.459242074
C	3.964805961	2.132201689	1.902922263
N	3.472494036	0.827077334	1.990533026
H	6.376122828	0.297367415	0.947648275
H	5.960920253	2.823139572	1.249499087
H	3.354700818	2.982136120	2.159351104
C	2.149636488	0.468082102	2.429472417
H	1.995691523	-0.591691995	2.216031718
H	1.393597940	1.054229148	1.898383397
S	-0.730358671	0.317040665	3.806477230
C	0.766104300	0.707805506	4.474212841
N	1.995676799	0.721202683	3.863507304
C	2.993327382	1.040794518	4.779192598
C	2.386817330	1.257548831	5.972243476
N	1.030836755	1.055443812	5.771578753
H	4.032743420	1.081759400	4.501622443
H	2.800854073	1.522478067	6.931485519
H	0.287419369	1.180034007	6.441210170
C	-3.040292863	-0.125525796	1.192061331
C	-3.323223754	1.676161337	2.442370307
H	-4.116508091	-0.191074301	1.318209048
H	-2.527036018	-1.003772607	1.579898509
H	-3.416800384	1.513486108	3.527060483
H	-4.289160352	1.891310150	1.967116614

8e

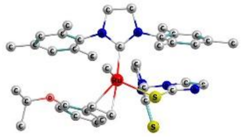

100

RuC40N6H50S2O

Ru	-1.876137857	1.403410540	1.289469456
C	-2.271238555	-0.350638494	0.014980971
H	-2.964736766	-0.094275468	-0.775361125
C	-0.923875994	-0.706396970	-0.459389283
C	-0.598648034	-0.558253214	-1.834876981
C	0.695307541	-0.808157688	-2.296498384
C	1.690862012	-1.244208822	-1.414618015
C	1.382035592	-1.454695031	-0.071438555
C	0.088919141	-1.192802100	0.389668028
H	0.926786889	-0.668007489	-3.347027987
H	2.692922478	-1.437745977	-1.788086622
H	2.134975006	-1.829777767	0.616754259
H	-0.152611121	-1.368601207	1.433628992
O	-1.620408881	-0.144291806	-2.643504189
C	-1.656158306	-0.515878073	-4.029209814
C	-2.933381430	0.117907375	-4.567645190
C	-1.655555922	-2.035521901	-4.203236415
H	-0.790562476	-0.076561150	-4.545327243
H	-2.933670843	1.191136881	-4.367350570
H	-3.021930093	-0.050204411	-5.646013965
H	-3.804609441	-0.322972170	-4.072075003
H	-0.748025984	-2.490865198	-3.800290026
H	-2.515136307	-2.466158739	-3.679429055
H	-1.728747788	-2.292423677	-5.265199291
C	-1.177942006	3.371216954	1.150188950
N	-1.047855775	4.030630833	-0.042312778
C	-0.627179534	5.439179205	0.099428096
C	-0.979506711	5.712594334	1.553732151
N	-1.021165988	4.343213034	2.110993405
H	0.452034140	5.529689812	-0.097386724
H	-1.155147254	6.083319602	-0.607985876
H	-0.244417782	6.332426026	2.072220507
H	-1.965507105	6.185078583	1.663110119
C	-0.922358760	3.476591037	-1.359943959
C	-1.928378110	3.766459199	-2.304452971
C	-3.246344400	4.361272919	-1.877534505
C	-1.703561940	3.443594192	-3.642061670
C	-0.520344024	2.827849067	-4.066150070
C	-0.300656899	2.520945363	-5.529011872
C	0.441912172	2.522863943	-3.106192340
C	0.266063594	2.843395679	-1.755254783
C	1.329501188	2.461326755	-0.761086323
H	-3.859163497	4.623905155	-2.744576653
H	-3.128589442	5.257599996	-1.259582920
H	-3.791788047	3.631041338	-1.271191195
H	-2.474674017	3.675464684	-4.374610234
H	0.565641458	1.869172491	-5.677680203
H	-0.128972834	3.437956790	-6.106341027
H	-1.174049786	2.024877156	-5.966493867
H	1.359639645	2.023003794	-3.406998514
H	1.357984802	3.160549547	0.079044276
H	1.118816850	1.461955072	-0.364631736
H	2.320540701	2.432037539	-1.223637741
C	-0.905083623	4.223347638	3.532493367
C	0.368168478	4.442117217	4.101008249
C	1.596075804	4.627321503	3.240030029
C	0.491417468	4.505777572	5.489451393
C	-0.611692005	4.325525400	6.327302233
C	-0.441280916	4.345938058	7.828703287
C	-1.856775745	4.089946035	5.740590527
C	-2.034423428	4.051463920	4.351470670
C	-3.403352275	3.828807955	3.770225336
H	1.830225746	5.688688149	3.084484758
H	2.469807062	4.167663748	3.712347331
H	1.453050619	4.178031757	2.254633315
H	1.473228292	4.678873667	5.924700472
H	0.038418629	3.424340600	8.187149974
H	0.196054208	5.177244813	8.149028283
H	-1.401334433	4.436932493	8.344596202
H	-2.727585836	3.953458701	6.378650340
H	-3.594326351	4.498747256	2.926412401
H	-3.484715375	2.808613380	3.379698030
H	-4.179821877	3.985645801	4.524621261
S	3.865816558	-1.456734867	3.072115142
C	4.122387012	0.050131362	2.393435461
N	5.299604812	0.571907318	1.922835360
C	5.135819177	1.865932503	1.445721576
C	3.832582518	2.181644224	1.617468330
N	3.208482306	1.073322047	2.193323994
H	6.150896837	0.032106816	1.932109199
H	5.948578006	2.437724438	1.028103227
H	3.286300922	3.076112919	1.374938017
C	1.806455276	0.949980136	2.488985682
H	1.490841040	-0.070774976	2.280194787
H	1.231147736	1.631303446	1.863324415
S	-1.170811443	0.626160274	3.602405450
C	0.253849148	1.087518727	4.402347287
N	1.515451870	1.242731808	3.894230167
C	2.423458611	1.508393038	4.912771478
C	1.719979403	1.553201666	6.070268853
N	0.399704543	1.287858575	5.744955630
H	3.476731305	1.621099619	4.721559013
H	2.041910637	1.742010922	7.081087764
H	-0.398149256	1.347161593	6.357979401
C	-2.773297972	-0.660796972	1.296313371
C	-3.522130757	2.124447521	0.822712442
H	-3.849718911	-0.636287365	1.450735626
H	-2.243677343	-1.336420071	1.963439786
H	-3.820442348	3.180384659	0.878183079
H	-4.387125127	1.476524967	0.601663618

9e

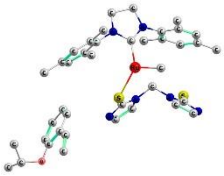

100

RuC40N6H50S2O

Ru	0.528448555	2.574834541	2.254693856
C	-3.025991724	-2.869693628	3.909872667
H	-2.763028571	-1.871987914	4.261577720
C	-3.427703413	-2.896526137	2.493374923
C	-3.965335870	-4.017875522	1.817223018
C	-4.240373201	-3.957707565	0.446915338
C	-4.040442813	-2.780538390	-0.272528986
C	-3.554014284	-1.648796384	0.384531502
C	-3.250299571	-1.714721067	1.744753426
H	-4.598812568	-4.854921468	-0.045001583
H	-4.262660727	-2.750169511	-1.335885185
H	-3.394150016	-0.721379429	-0.157434283
H	-2.845997322	-0.833962475	2.235687367
O	-4.161128102	-5.198770489	2.492571938
C	-5.515769972	-5.558531781	2.893724454
C	-6.267262447	-6.245636468	1.753475973
C	-6.288874698	-4.375592099	3.473555826
H	-5.334145690	-6.291475704	3.687745404
H	-5.651078625	-7.032199939	1.308182610
H	-7.189659407	-6.699064045	2.132483904
H	-6.543870036	-5.530053886	0.972902646
H	-5.723814890	-3.898465013	4.277135473
H	-6.495620198	-3.626344685	2.702744477
H	-7.247212655	-4.723704556	3.872412583
C	-0.162590105	4.208471670	1.419031394
N	0.413120935	5.214916019	0.682940005
C	-0.524235856	6.287296327	0.321629317
C	-1.892517663	5.655598530	0.634188207
N	-1.510045542	4.509678742	1.467142173
H	-0.338277849	7.182863772	0.931052307
H	-0.416261516	6.568611219	-0.730815488
H	-2.557595758	6.331768806	1.182092362
H	-2.417183386	5.335463099	-0.278043368
C	1.732981824	5.155454649	0.143149481
C	1.923668330	4.530456392	-1.102799999
C	0.739145517	4.001304485	-1.876173563
C	3.225130441	4.379028915	-1.586625277
C	4.327763300	4.853802336	-0.865852008
C	5.726067450	4.637142055	-1.397128342
C	4.100202964	5.526668206	0.338842479
C	2.811019933	5.693716076	0.858165654
C	2.571157580	6.372579496	2.183053058
H	1.061173289	3.470924976	-2.777250411
H	0.069445428	4.809793718	-2.193256495
H	0.143509036	3.321111691	-1.259330703
H	3.390452019	3.847583295	-2.520818756
H	5.986489568	5.388966997	-2.153304605
H	5.811482245	3.654882526	-1.874610646
H	6.472425904	4.701813409	-0.598970125
H	4.948166869	5.908913171	0.904047258
H	2.060815381	5.685279613	2.866240045
H	3.511940685	6.692021630	2.640197005
H	1.932907945	7.256749219	2.068253331
C	-2.481201241	3.607056266	2.002764705
C	-2.866752601	3.737546063	3.347538243
C	-2.212123159	4.778909158	4.219649810
C	-3.828586184	2.858408620	3.857666181
C	-4.415681861	1.871381620	3.062562526
C	-5.447294991	0.915896074	3.616578560
C	-4.014653798	1.766426318	1.725981955
C	-3.042112803	2.606925000	1.181916041
C	-2.549677470	2.393505308	-0.229084188
H	-1.137124958	4.575345667	4.290019591
H	-2.321796613	5.783373880	3.795082576
H	-2.640952146	4.779772204	5.225748102
H	-4.122016720	2.947278410	4.901439876
H	-5.096170606	-0.121139836	3.547284112
H	-5.673778498	1.127143880	4.666020883
H	-6.385254422	0.972214091	3.050224716
H	-4.459906780	0.996314859	1.100939124
H	-1.505727188	2.060844882	-0.204560947
H	-3.143722543	1.631159655	-0.741775168
H	-2.584597930	3.305342821	-0.833128255
S	-0.055338376	0.232027699	1.942275919
C	0.273582891	-0.345341363	0.353353660
N	-0.336963477	-1.433964600	-0.184539375
C	0.122633768	-1.684499474	-1.468669364
C	1.048467395	-0.733672645	-1.739367224
N	1.132625444	0.091664493	-0.617545995
H	-1.089228007	-1.917374539	0.291894921
H	-0.245600274	-2.506334179	-2.060893608
H	1.683191105	-0.556661894	-2.595023710
C	2.031338358	1.238993480	-0.528139799
H	1.637548712	1.916426214	0.259599686
H	2.056598306	1.726353684	-1.498834728
S	4.300036247	0.820728946	-2.826603406
C	4.378508520	0.668688091	-1.145035394
N	3.382438113	0.842399556	-0.210213309
C	3.863112944	0.603697036	1.076285047
C	5.172728372	0.286070685	0.957204637
N	5.472944358	0.333984494	-0.400703538
H	3.218897277	0.716601619	1.933819263
H	5.909710166	0.038642175	1.703595127
H	6.363993662	0.146011615	-0.832564033
C	-2.905389441	-3.887407753	4.775805797
C	2.247528557	3.015759032	2.656967817
H	-2.563476372	-3.697238791	5.789079626
H	-3.118889749	-4.911780009	4.500381979
H	2.531403900	2.623817105	3.657247105
H	3.081859944	3.452350450	2.096888658

1f

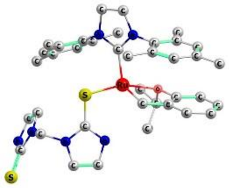

94

RuC38N6H46S2O

Ru	0.014603987	0.984727383	0.607956990
C	0.976530757	0.260842038	-0.895983345
H	1.911363831	0.689001540	-1.283832182
C	0.194075861	-0.428371355	-1.906547926
C	-1.135359503	-0.795039816	-1.609342696
C	-1.973916327	-1.421109736	-2.520126120
C	-1.478791277	-1.762417207	-3.783000277
C	-0.153240692	-1.450150075	-4.105116239
C	0.659012524	-0.778769195	-3.195587094
H	-3.003936944	-1.637631063	-2.251109547
H	-2.121412140	-2.261710648	-4.502246195
H	0.242038099	-1.719906230	-5.081960887
H	1.674868796	-0.504579168	-3.469915560
O	-1.587697353	-0.446598938	-0.321442299
C	-2.019557971	-1.595916798	0.486442866
C	-2.759994168	-1.053822230	1.694919364
C	-0.833388342	-2.481920747	0.852926264
H	-2.717578300	-2.168652895	-0.134689332
H	-3.638238195	-0.482767735	1.386793353
H	-3.088309460	-1.885429139	2.327262944
H	-2.107112611	-0.403526690	2.286244968
H	-0.282080795	-2.771829544	-0.045992482
H	-0.152287126	-1.947698282	1.521880953
H	-1.190823567	-3.390633497	1.350602184
C	-0.771822333	2.816029819	0.517584493
N	-1.002453113	3.509550478	-0.633872003
C	-1.335893947	4.925427100	-0.422519668
C	-1.591946027	4.984776302	1.092670267
N	-1.043002150	3.697276236	1.548339189
H	-0.499946985	5.569508193	-0.725197573
H	-2.210544009	5.209396424	-1.017558099
H	-1.082464350	5.823659627	1.578995966
H	-2.662267101	5.057153378	1.328961127
C	-0.760981863	3.029959668	-1.959333655
C	-1.817450954	2.422916668	-2.656679134
C	-3.135724734	2.158198828	-1.970565826
C	-1.599684907	2.022447586	-3.976477361
C	-0.363234715	2.204456923	-4.600670764
C	-0.137357668	1.706909175	-6.007603305
C	0.673401859	2.793700412	-3.870961719
C	0.499365829	3.209426129	-2.548401314
C	1.647523594	3.774996790	-1.749097761
H	-3.526128138	3.052405883	-1.471849557
H	-3.006783311	1.387562500	-1.202685585
H	-3.886056462	1.807209106	-2.684887131
H	-2.403543316	1.530779579	-4.519612065
H	-1.014856576	1.875826045	-6.641022409
H	0.053269144	0.627114477	-5.996005489
H	0.722084183	2.194715918	-6.478004935
H	1.649128551	2.916768954	-4.337015149
H	1.456924410	4.803069133	-1.418095728
H	1.813319731	3.167187087	-0.853375757
H	2.568160052	3.782589131	-2.339527448
C	-1.266580182	3.255506911	2.888192313
C	-0.351585069	3.597668310	3.894298202
C	0.842852566	4.472131609	3.605054170
C	-0.583057073	3.130298785	5.195643065
C	-1.689177848	2.339558827	5.507106792
C	-1.918665222	1.825244240	6.909889785
C	-2.606073128	2.044656756	4.489357736
C	-2.426138985	2.507251951	3.185640733
C	-3.471990772	2.259789246	2.124036605
H	1.705445114	4.173049248	4.208440191
H	1.126232382	4.419192638	2.553566713
H	0.620957591	5.519493296	3.846798777
H	0.131905099	3.379945931	5.977056706
H	-1.084507677	2.075094620	7.572701631
H	-2.828145947	2.253817269	7.347539539
H	-2.048134133	0.735879655	6.917177264
H	-3.489768786	1.453625312	4.722483559
H	-3.929123386	3.202224630	1.799392015
H	-3.039593516	1.789529527	1.237951574
H	-4.271327134	1.618553794	2.504290865
S	1.924043922	1.694056838	1.817586636
C	2.846843124	0.240932114	1.968881575
N	2.738858766	-0.848650353	1.186411797
C	3.609596125	-1.836991207	1.610462349
C	4.283841306	-1.351782710	2.683928859
N	3.818647175	-0.048884663	2.890352691
H	2.021573798	-0.800887898	0.402746065
H	3.673735783	-2.792857005	1.115585318
H	5.014932294	-1.789798756	3.345998013
C	4.115137442	0.771211455	4.050664185
H	3.958071076	1.816382302	3.782721209
H	5.148620330	0.599756325	4.354525262
S	5.085237109	-1.270149792	6.375962318
C	3.620628932	-0.470173876	6.158287771
N	3.265414118	0.431259602	5.173877333
C	1.933351497	0.826985827	5.320489821
C	1.454639576	0.201746083	6.419978981
N	2.492453626	-0.575982415	6.923682020
H	1.457069798	1.483995508	4.610686428
H	0.480270708	0.240581489	6.877818582
H	2.462541789	-1.177714850	7.731737136

2f

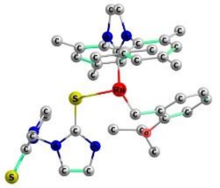

94

RuC38N6H46S2O

Ru	0.248655802	1.253300672	0.307350733
C	0.835947611	0.031410478	-1.025732749
H	1.503612883	0.167139934	-1.884833296
C	-0.523633022	-0.481045718	-1.297659476
C	-1.244070680	-1.241724529	-0.321981182
C	-2.594379658	-1.569622551	-0.533862447
C	-3.238610970	-1.196429780	-1.709441380
C	-2.543469765	-0.496750786	-2.702486426
C	-1.219741438	-0.141286835	-2.484869352
H	-3.132476433	-2.147708625	0.209003869
H	-4.281128856	-1.467787903	-1.855866193
H	-3.041041170	-0.215468903	-3.626749183
H	-0.682740187	0.444424857	-3.225034650
O	-0.552410138	-1.723425804	0.761163877
C	-1.216684096	-1.837833206	2.038241862
C	-0.271352331	-1.272859748	3.094311165
C	-1.569058203	-3.302715537	2.294328166
H	-2.125760593	-1.222483652	2.017662696
H	-0.046272971	-0.226975236	2.871117993
H	-0.730996926	-1.332254724	4.087039091
H	0.667264962	-1.836492266	3.111614162
H	-2.226150448	-3.702152604	1.516784971
H	-0.655189765	-3.905937986	2.304274498
H	-2.067742085	-3.417141663	3.263061174
C	-0.593436009	3.064619833	0.228912415
N	-0.751458778	3.885078298	-0.847120817
C	-1.215650556	5.234159097	-0.487863380
C	-1.736514451	5.026759086	0.942149130
N	-1.087716366	3.757929340	1.316924822
H	-0.377920630	5.944706955	-0.522442400
H	-1.986219434	5.587187877	-1.179632141
H	-1.454075657	5.837277475	1.621236466
H	-2.830775062	4.923664806	0.971953607
C	-0.345963303	3.581657604	-2.183497306
C	-1.346321503	3.225467999	-3.103808201
C	-2.780734661	3.074685697	-2.657106337
C	-0.975232215	2.959077091	-4.424275453
C	0.359743146	3.021946873	-4.834571957
C	0.751302063	2.681113679	-6.253868189
C	1.330350876	3.378392982	-3.893102298
C	1.003072230	3.668614986	-2.565369481
C	2.077867923	4.021211132	-1.569581834
H	-3.195699916	4.011104654	-2.266538739
H	-2.853209995	2.325754488	-1.862608787
H	-3.416078918	2.748796908	-3.485207655
H	-1.742946470	2.678469174	-5.142759194
H	-0.093739154	2.787255382	-6.941118365
H	1.103297618	1.644381780	-6.324037007
H	1.563262415	3.322855811	-6.611739904
H	2.372815092	3.435742262	-4.200271157
H	1.755025816	4.809033337	-0.881539335
H	2.312107262	3.146101154	-0.953733803
H	2.989201952	4.353875721	-2.075249334
C	-1.475121238	3.075964047	2.513898384
C	-0.785647828	3.359455933	3.705080233
C	0.348943783	4.354317425	3.712553359
C	-1.156252240	2.685226734	4.873698175
C	-2.182321796	1.736382583	4.878801585
C	-2.528054504	0.963360885	6.130408815
C	-2.870417729	1.496559765	3.685928187
C	-2.542840587	2.153888544	2.496590317
C	-3.317848812	1.848781742	1.236146846
H	0.852669768	4.368438223	4.683482652
H	1.084695138	4.104046148	2.942983306
H	-0.004116347	5.371439466	3.505153690
H	-0.621155071	2.901162037	5.796535821
H	-2.178856019	1.475688117	7.033165346
H	-3.608526510	0.813837442	6.227453415
H	-2.068710723	-0.034132012	6.111728226
H	-3.688248352	0.778069647	3.679314758
H	-3.454833296	2.733978493	0.609487747
H	-2.785384585	1.110324845	0.626891551
H	-4.305511424	1.443593672	1.477002886
S	2.156825022	1.761524475	1.593457580
C	2.807182372	0.173954066	1.857987445
N	2.549260646	-0.909601810	1.110485726
C	3.201848511	-2.011102242	1.628223160
C	3.899130169	-1.601852610	2.721059474
N	3.662631492	-0.230204675	2.847536201
H	1.840610566	-0.777856249	0.293907351
H	3.115656747	-2.986528210	1.176690442
H	4.511645541	-2.126463139	3.438457878
C	4.036208763	0.582180350	3.990694979
H	4.040368991	1.627920206	3.682287018
H	5.022312316	0.277300499	4.343579260
S	4.641978140	-1.454314867	6.438908824
C	3.300764175	-0.489478774	6.119718994
N	3.105191024	0.406973816	5.087087805
C	1.825296898	0.964391898	5.149404456
C	1.223654231	0.448448908	6.245164470
N	2.134580938	-0.424575730	6.830595346
H	1.463909078	1.637826873	4.388686604
H	0.239713273	0.618905932	6.647700149
H	1.994705718	-0.982137822	7.658477859

3f’

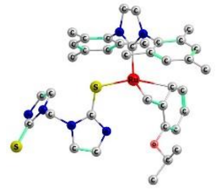

94
RuC38N6H46S2O

Ru	0.062592255	1.377030226	0.225624134
C	0.488902817	0.154014447	-1.123018660
H	1.391812618	-0.245757697	-1.581079110
C	-0.800499690	-0.453269621	-1.018935638
C	-1.059479553	-1.816511899	-0.645530081
C	-2.301570229	-2.224768053	-0.226837288
C	-3.371471161	-1.293403621	-0.139512243
C	-3.172677192	0.023124138	-0.497528735
C	-1.908343126	0.464757586	-0.970324551
H	-2.439316619	-3.256823105	0.086629637
H	-4.345947451	-1.633605202	0.200565008
H	-4.002237680	0.726232717	-0.466629727
H	-1.839850455	1.368352166	-1.560433443
O	0.031977537	-2.675771859	-0.570132969
C	0.150449892	-3.665068840	-1.615944699
C	1.184144574	-4.669593655	-1.124511160
C	0.534647254	-3.012352192	-2.941643267
H	-0.824732612	-4.162626659	-1.722687010
H	0.869146805	-5.107554803	-0.172085555
H	1.318435133	-5.476297116	-1.851921768
H	2.150550507	-4.173807748	-0.979633515
H	-0.201115458	-2.255248919	-3.226488984
H	1.510779854	-2.523880040	-2.854023362
H	0.587929671	-3.762970477	-3.737934456
C	-0.160128723	3.368090976	0.054335121
N	-0.130043785	4.099983941	-1.099166665
C	-0.100910173	5.551864823	-0.871504307
C	-0.547828350	5.651805716	0.593877280
N	-0.291418690	4.284378398	1.073453767
H	0.914834654	5.945800808	-1.019387584
H	-0.771199909	6.073816875	-1.560813394
H	0.021180250	6.391249212	1.165116808
H	-1.615993417	5.898080895	0.685467548
C	-0.008133370	3.545181028	-2.409313557
C	-1.175386035	3.397548039	-3.176258008
C	-2.516250810	3.828607842	-2.628211521
C	-1.072096547	2.823401156	-4.446336836
C	0.157943695	2.404291103	-4.959629833
C	0.246525363	1.732619446	-6.310361385
C	1.304375525	2.589480224	-4.180492170
C	1.246299967	3.153181473	-2.904241341
C	2.486737577	3.280804372	-2.058317858
H	-2.606839812	4.921718893	-2.592437763
H	-2.661346154	3.463126601	-1.606609011
H	-3.333020884	3.450241591	-3.249018840
H	-1.973577197	2.687852219	-5.040524304
H	-0.602620322	1.995533608	-6.948800632
H	0.248929751	0.640933757	-6.201775145
H	1.166496642	2.007458693	-6.837072548
H	2.269233530	2.271349307	-4.570803586
H	2.548610131	4.259078139	-1.570509783
H	2.469171732	2.527981296	-1.263095388
H	3.389747599	3.139593728	-2.659363113
C	-0.555393488	3.913765252	2.426761148
C	0.440317556	4.150651197	3.392198323
C	1.765517723	4.756777907	2.997223035
C	0.184633265	3.799201444	4.721503522
C	-1.021851791	3.205787781	5.103594915
C	-1.272550192	2.791838688	6.536009786
C	-1.995380655	2.989383113	4.124503880
C	-1.792108378	3.342136227	2.786687184
C	-2.879143566	3.098309852	1.766882116
H	2.494933907	4.665207849	3.807458522
H	2.169400389	4.262444815	2.109703218
H	1.668494902	5.824100837	2.761453035
H	0.953118323	3.979679900	5.470787320
H	-0.536492181	3.230634581	7.217889333
H	-2.267193130	3.100835246	6.875514270
H	-1.224700877	1.700469964	6.643866227
H	-2.945697332	2.541412984	4.408482238
H	-2.939015476	3.910855567	1.035966917
H	-2.677437944	2.181495489	1.202568361
H	-3.853591387	2.999839057	2.254779895
S	2.122434011	1.384031470	1.415107574
C	2.556567950	-0.243070288	1.773682638
N	2.041618405	-1.380710945	1.257242788
C	2.650951925	-2.495929014	1.808540076
C	3.579101045	-2.050699603	2.689964236
N	3.529012572	-0.654707632	2.650459475
H	1.312373112	-1.385006426	0.541871001
H	2.361164049	-3.493389587	1.522100027
H	4.248996690	-2.560485190	3.364999698
C	4.261810025	0.235076588	3.532169886
H	4.360121755	1.201551131	3.036707646
H	5.241323488	-0.198127881	3.739413558
S	5.049039122	-1.513252124	6.142015803
C	3.862332319	-0.338654147	5.925297688
N	3.586013267	0.412988874	4.800375773
C	2.484792958	1.245475953	5.016934936
C	2.084089794	1.044305048	6.292783688
N	2.935070632	0.086474152	6.835284739
H	2.080403937	1.864769174	4.232143032
H	1.276543032	1.488170521	6.850602334
H	2.900504462	-0.298365838	7.766230329

3f

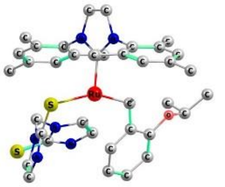

94
RuC38N6H46S2O

Ru	0.584996043	1.508311351	0.462558462
C	-0.214293664	0.581719461	-0.917640983
H	-0.886703433	0.777521167	-1.750623616
C	0.207271233	-0.780639159	-0.598248557
C	-0.748372921	-1.818191920	-0.372652225
C	-0.358934560	-3.038621635	0.163766358
C	0.992327803	-3.269361910	0.489177896
C	1.946562364	-2.291088681	0.258451477
C	1.561068981	-1.064283634	-0.306268733
H	-1.088792925	-3.821238555	0.342835460
H	1.277845209	-4.228639067	0.912781407
H	2.991566072	-2.473827808	0.494186870
H	2.309914926	-0.325978060	-0.580860924
O	-2.027901561	-1.479147954	-0.721282907
C	-3.146321022	-2.240160706	-0.246349839
C	-4.333508422	-1.776170785	-1.081256850
C	-3.355098464	-1.997857164	1.249047288
H	-2.975021282	-3.309949837	-0.435602207
H	-4.142461344	-1.947411284	-2.144220642
H	-5.240338006	-2.317761100	-0.793707454
H	-4.499776442	-0.705174473	-0.930227291
H	-2.475515507	-2.306167491	1.818931697
H	-3.519436612	-0.931245671	1.431352734
H	-4.220916463	-2.558055560	1.617305011
C	-0.608417256	3.043138781	0.605636835
N	-1.334156591	3.709351858	-0.349082652
C	-1.952825481	4.948616458	0.141722074
C	-1.901729738	4.747911922	1.659729870
N	-0.859281914	3.721770087	1.786511470
H	-1.369999026	5.825103193	-0.177708469
H	-2.970972172	5.061221755	-0.243874422
H	-1.634425703	5.658874871	2.204501687
H	-2.859475463	4.381558813	2.058478376
C	-1.319208707	3.438453109	-1.750820235
C	-2.407800439	2.736238678	-2.296574516
C	-3.480149933	2.182269462	-1.389422821
C	-2.423094591	2.490723531	-3.670794546
C	-1.380561956	2.918081822	-4.500628368
C	-1.396475484	2.595550951	-5.977346231
C	-0.311031234	3.610053092	-3.925791053
C	-0.257705824	3.880420409	-2.554052258
C	0.939674218	4.563910580	-1.944101364
H	-3.955759054	2.961865324	-0.783804650
H	-3.035396637	1.458680937	-0.696056685
H	-4.260412094	1.676983780	-1.966232695
H	-3.255219160	1.936735838	-4.101042636
H	-0.760184885	3.280156623	-6.546560245
H	-2.409669784	2.651416530	-6.389603452
H	-1.028657754	1.577689436	-6.159613394
H	0.510268588	3.941094963	-4.558477941
H	0.651347531	5.371347714	-1.262688005
H	1.508577767	3.836927756	-1.353360866
H	1.592982345	4.981930046	-2.715559377
C	-0.451643450	3.272211487	3.078853752
C	0.592967137	3.961349028	3.722653052
C	1.299865230	5.096190350	3.023330404
C	0.970260153	3.560728565	5.008857637
C	0.343243220	2.493748634	5.660322818
C	0.804010229	2.031085843	7.022398183
C	-0.699120038	1.834008182	4.998285153
C	-1.120674518	2.208979917	3.719169593
C	-2.258044307	1.489279630	3.035894625
H	2.148045621	5.453265516	3.613966825
H	1.670909701	4.772277974	2.047261762
H	0.628269207	5.945957191	2.852449069
H	1.782545518	4.085698487	5.507387778
H	1.453008993	1.148979797	6.941841861
H	1.369218228	2.811729323	7.540520124
H	-0.044049055	1.748838305	7.655259885
H	-1.195119870	1.000219717	5.490685236
H	-3.100491530	2.165044893	2.846752023
H	-1.929337134	1.098810234	2.065332906
H	-2.619748844	0.659512526	3.649322747
S	2.855660453	2.392854101	0.879047345
C	3.702922257	1.079979671	1.585273758
N	4.798342567	0.475101620	1.047421471
C	5.264263727	-0.546591387	1.865109596
C	4.445129855	-0.580911279	2.942853299
N	3.490797505	0.419423935	2.766864909
H	5.174653422	0.760500234	0.156315540
H	6.122901475	-1.146393906	1.611211985
H	4.437238998	-1.201692780	3.826822375
C	2.424422915	0.706099865	3.721343175
H	1.837258311	1.536177394	3.334527052
H	2.867201459	0.972174836	4.682383625
S	2.929344181	-1.356165359	6.173136003
C	1.741899783	-1.336925956	4.970603934
N	1.573440309	-0.442250516	3.935003435
C	0.473630095	-0.790370414	3.148814919
C	-0.058019018	-1.905739007	3.695636362
N	0.721629295	-2.224914781	4.804631841
H	0.194383141	-0.193302569	2.278647351
H	-0.897583156	-2.505335725	3.389060559
H	0.600344001	-3.006612118	5.429259172

4f‡ (27.70*i* cm-1)

100

RuC40N6H50S2O

Ru	0.621672994	1.636100780	0.568431614
C	-0.779262452	0.450267201	0.137453016
H	-1.826488351	0.710291263	-0.005555504
C	-0.589238782	-1.001487701	0.209382734
C	-1.654121778	-1.851075225	0.634747561
C	-1.463309506	-3.227788287	0.779624278
C	-0.215337159	-3.798315274	0.502057643
C	0.842412704	-2.994622111	0.082770048
C	0.648084366	-1.621385347	-0.060342203
H	-2.278976482	-3.867947492	1.097182952
H	-0.085126294	-4.872034293	0.609349103
H	1.810720509	-3.434862904	-0.140340663
H	1.468030561	-0.999653770	-0.410099441
O	-2.839917898	-1.212592566	0.882482463
C	-3.932411024	-1.888483462	1.518719668
C	-5.145398850	-0.995991938	1.281313400
C	-3.642129869	-2.093323052	3.006717546
H	-4.103830975	-2.857882476	1.028051269
H	-5.329920095	-0.877139436	0.209885677
H	-6.038026010	-1.426957113	1.745814631
H	-4.968036261	-0.004789004	1.710820165
H	-2.747194864	-2.702586529	3.158075000
H	-3.479265173	-1.122655177	3.485555443
H	-4.483676268	-2.592272026	3.498578553
C	-0.636332684	3.068650060	0.813296385
N	-1.568270253	3.533723077	-0.087718427
C	-2.252440287	4.755151115	0.359817627
C	-1.926631476	4.779898098	1.858931516
N	-0.795350332	3.846215165	1.943121225
H	-1.851014528	5.633519124	-0.165494548
H	-3.326126040	4.701283782	0.153254893
H	-1.645788622	5.774887083	2.219110020
H	-2.767204837	4.422428291	2.470409769
C	-1.638601398	3.169416304	-1.469193844
C	-2.733859578	2.418941293	-1.923696413
C	-3.840983212	1.958278777	-1.001957764
C	-2.790214295	2.071513546	-3.279384029
C	-1.799778846	2.458125293	-4.180563211
C	-1.845541793	2.031958763	-5.629582482
C	-0.741502162	3.243719278	-3.706274501
C	-0.645658354	3.617048753	-2.366294508
C	0.515939749	4.455892880	-1.892147655
H	-4.805707389	2.382381428	-1.306956649
H	-3.650034678	2.237968160	0.035498102
H	-3.936798863	0.867500703	-1.030425696
H	-3.628376688	1.472748381	-3.631554186
H	-1.555871710	2.850745599	-6.297478153
H	-2.845169385	1.695159893	-5.920212620
H	-1.150792530	1.203297084	-5.815825381
H	0.028441620	3.573839805	-4.401497468
H	0.195860040	5.260668505	-1.222107540
H	1.221529321	3.839643655	-1.324044547
H	1.045223519	4.906157851	-2.737481494
C	-0.224400455	3.551823342	3.219935758
C	0.806864947	4.372637594	3.704942723
C	1.320309051	5.532877568	2.887915496
C	1.350527915	4.092592663	4.965547837
C	0.893053145	3.027131346	5.744168217
C	1.539181343	2.677547180	7.064567263
C	-0.160326803	2.248354183	5.246961827
C	-0.740075031	2.495623430	4.001205288
C	-1.884153181	1.645799037	3.503460454
H	2.332067245	5.811747744	3.194995984
H	1.339771951	5.285977765	1.824488356
H	0.680635017	6.417149786	3.011908728
H	2.165559763	4.711447679	5.335178923
H	2.126459648	1.752945077	6.983448471
H	2.210165628	3.470424842	7.408112415
H	0.787225330	2.508776349	7.843524019
H	-0.531647175	1.417612364	5.843534939
H	-2.768520732	2.255245219	3.282512776
H	-1.612765937	1.129376058	2.577640134
H	-2.167121256	0.900877400	4.252353297
S	2.817197410	2.858660462	0.993315832
C	3.763339840	1.530352494	1.519279529
N	4.732202229	0.908271010	0.798285397
C	5.328031385	-0.109948941	1.529111153
C	4.724316429	-0.117596255	2.742593095
N	3.762938988	0.890434056	2.728215771
H	4.887610440	1.132786246	-0.173746688
H	6.122757124	-0.719753178	1.131139309
H	4.874346398	-0.725416680	3.623015239
C	2.806827326	1.123186698	3.813821849
H	2.155046725	1.940230546	3.512267910
H	3.348595674	1.370640016	4.727270463
S	3.570458484	-0.992300262	6.168049122
C	2.336355508	-1.016994197	5.013344841
N	2.027372356	-0.066788981	4.063528579
C	0.945001924	-0.475278529	3.281759687
C	0.564748066	-1.688168360	3.739654601
N	1.415113751	-2.000732114	4.800045854
H	0.570036171	0.169833312	2.479515238
H	-0.204869759	-2.354651443	3.384512776
H	1.403706637	-2.837872356	5.361481196
C	2.002309194	0.865878071	-2.395848217
C	3.180063280	0.239444626	-2.309026804
H	1.056248244	0.341212821	-2.312783110
H	1.930026697	1.935409884	-2.560950503
H	3.252660450	-0.834122294	-2.151349750
H	4.120542355	0.778068367	-2.418862275

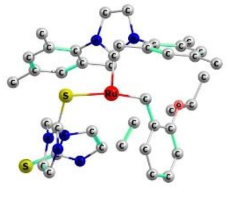

4f$_{cis}$

100

RuC40N6H50S2O

Ru	-0.067919141	1.469586784	-0.052430621
C	-0.564024905	0.417189127	1.396113744
H	-1.295071458	0.781833900	2.117424646
C	-0.070682378	-0.920285807	1.762069398
C	-0.823890630	-1.775920047	2.616874535
C	-0.290754947	-2.995866205	3.058467445
C	0.988350927	-3.396683085	2.663768850
C	1.740223279	-2.585238761	1.815380237
C	1.203702022	-1.378122540	1.379081493
H	-0.866850897	-3.647725996	3.704075804
H	1.385142010	-4.343429555	3.020869015
H	2.744372544	-2.872753262	1.518589634
H	1.782243499	-0.727558098	0.741769505
O	-2.076793355	-1.335203230	2.949551079
C	-2.908881195	-2.103901393	3.832355206
C	-4.330281203	-1.608259712	3.595467129
C	-2.464500962	-1.921631895	5.284835770
H	-2.855796386	-3.164011683	3.547480199
H	-4.610960783	-1.738362689	2.546670843
H	-5.037554068	-2.162135880	4.221474517
H	-4.402600184	-0.546230465	3.840979150
H	-1.431859939	-2.249023923	5.431614096
H	-2.528214921	-0.864361522	5.551793595
H	-3.106551736	-2.496304285	5.960769590
C	-1.121633771	3.141958143	0.605696168
N	-1.246029610	4.185668693	-0.284671910
C	-2.123026043	5.276408643	0.152746932
C	-2.296326664	4.953968413	1.641090899
N	-1.802327261	3.564510807	1.723638298
H	-1.656379166	6.251529266	-0.023866138
H	-3.078912594	5.253730847	-0.388559579
H	-1.693248795	5.612088693	2.280482970
H	-3.335511357	5.021993413	1.979838094
C	-0.771529719	4.115354787	-1.629715868
C	-1.584918531	3.530705876	-2.623562651
C	-2.969368391	3.032509802	-2.281641697
C	-1.084571218	3.439338168	-3.921231564
C	0.186801031	3.927615879	-4.256991880
C	0.697504054	3.793752528	-5.673564697
C	0.948557500	4.542567961	-3.263591609
C	0.484204450	4.657671587	-1.945900408
C	1.305362699	5.397088695	-0.918530885
H	-3.372336636	2.410002269	-3.085369889
H	-3.663008041	3.870311715	-2.136055890
H	-2.952473814	2.442410940	-1.361614907
H	-1.696434328	2.973984962	-4.691702608
H	1.717159271	4.176774929	-5.774001394
H	0.061368975	4.343734386	-6.377873086
H	0.698244130	2.745549219	-5.994771349
H	1.929015813	4.943642320	-3.511706823
H	1.014841525	5.126039001	0.097514377
H	2.369956437	5.179180793	-1.036890276
H	1.169919678	6.481003090	-1.032323517
C	-1.742505482	2.978897754	3.023734195
C	-0.608267312	3.184023182	3.831900733
C	0.566335289	3.982769644	3.320841928
C	-0.603074023	2.647435038	5.121295242
C	-1.708342966	1.959944977	5.637873385
C	-1.667995139	1.397905315	7.039569995
C	-2.833269458	1.803223616	4.823245558
C	-2.864266130	2.290634695	3.511235960
C	-4.045050622	2.027690819	2.608989023
H	1.402894697	3.946523941	4.024366657
H	0.906410174	3.609452803	2.351576740
H	0.299704099	5.037157832	3.182366023
H	0.276298389	2.788598243	5.746974121
H	-1.027273219	0.508608108	7.089992431
H	-1.262475327	2.125847967	7.750641435
H	-2.663800038	1.105909328	7.384864478
H	-3.707390557	1.288618250	5.215764921
H	-4.344463943	2.918744595	2.049527394
H	-3.780577623	1.260817610	1.871303848
H	-4.909392070	1.673466505	3.177893252
S	2.253839138	2.382274576	0.289679048
C	3.308556022	1.059589254	0.039270898
N	3.331067956	0.224601481	-1.027519827
C	4.265686068	-0.783769618	-0.846762218
C	4.874360361	-0.551692296	0.341422091
N	4.286132404	0.596590700	0.879587999
H	2.550898694	0.233870598	-1.678092805
H	4.412300851	-1.566674468	-1.572784232
H	5.627931329	-1.094865629	0.888527295
C	4.503727703	1.094060081	2.232978691
H	4.306220604	2.165747351	2.234335755
H	5.536727878	0.883651625	2.513186289
S	5.346924627	-1.651529339	3.720063596
C	3.936683709	-0.733542586	3.829012084
N	3.634536563	0.455929820	3.196500265
C	2.346758421	0.881331889	3.537894843
C	1.846776199	-0.026984180	4.402796631
N	2.827953988	-0.994252007	4.580610006
H	1.893148329	1.733948983	3.067217791
H	0.871512136	-0.087363824	4.856478820
H	2.732332557	-1.857151472	5.092067374
C	-1.057535015	0.269330734	-1.532113690
C	0.326708720	-0.118444920	-1.571787358
H	-1.794602183	-0.412379681	-1.114791419
H	-1.448705096	0.878484221	-2.345108048
H	0.611281484	-1.105941781	-1.209396297
H	0.880539148	0.198326210	-2.466003028

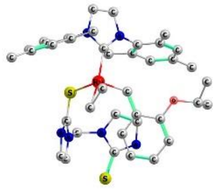

5f‡$_{cis}$ (372.31*i* cm^{-1})

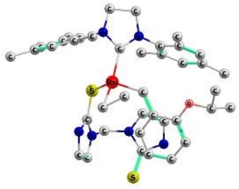


```
100
RuC40N6H50S2O
Ru    -0.138448839    1.478573016    0.004368064
C     -0.905677335    0.278037664    1.305503859
H     -1.828793485    0.524965039    1.829968583
C     -0.212906157   -0.866714376    1.968108128
C     -0.859691856   -1.665166070    2.949524061
C     -0.184904621   -2.739018876    3.550241030
C      1.123559985   -3.050800377    3.176251015
C      1.774286950   -2.291560157    2.207227827
C      1.101785320   -1.218640196    1.628093414
H     -0.674570701   -3.351699012    4.297026360
H      1.625682154   -3.892064146    3.647978052
H      2.797537953   -2.515190794    1.919928275
H      1.588978021   -0.606921048    0.883879040
O     -2.159408967   -1.340980051    3.237551645
C     -2.971740945   -2.227162134    4.022601746
C     -4.412771811   -1.939404439    3.616893143
C     -2.744889458   -2.003453922    5.519479732
H     -2.727952648   -3.265043289    3.754709686
H     -4.542357795   -2.078817029    2.540428982
H     -5.100328934   -2.607495144    4.145628030
H     -4.675087220   -0.906519517    3.865769429
H     -1.694575557   -2.129825200    5.792762589
H     -3.042016508   -0.987992286    5.791954211
H     -3.340874920   -2.710545918    6.106315160
C     -1.098834841    3.196473672    0.615759067
N     -1.339511098    4.175746856   -0.323015818
C     -2.012671063    5.372014338    0.194790978
C     -2.501940421    4.885816828    1.568731487
N     -1.732352352    3.641253573    1.748780061
H     -1.309910981    6.213202086    0.278766711
H     -2.831520872    5.680536024   -0.464147298
H     -2.289557895    5.595313585    2.375282749
H     -3.580786667    4.680588452    1.572880607
C     -0.802412843    4.128086654   -1.645233443
C     -1.535151136    3.465591686   -2.651473335
C     -2.879002130    2.856865735   -2.332196417
C     -0.996222189    3.399512643   -3.935327282
C      0.240731294    3.982802106   -4.243358064
C      0.802289681    3.875576781   -5.642736545
C      0.929610121    4.654501818   -3.233744188
C      0.426568659    4.742047569   -1.929312162
C      1.194826250    5.494399257   -0.870293029
H     -3.288052632    2.327620121   -3.197221577
H     -3.600649353    3.627160568   -2.032940320
H     -2.791486307    2.155023353   -1.498441146
H     -1.551006598    2.882703018   -4.716178886
H      1.733804880    4.439131055   -5.748698351
H      0.091846494    4.255763860   -6.386436111
H      1.011601574    2.831421683   -5.904922371
H      1.890100241    5.114172033   -3.457529319
H      0.959821279    5.128960116    0.131330907
H      2.271995712    5.386769871   -1.021847397
H      0.957811362    6.565835714   -0.903768154
C     -1.670639114    3.018457219    3.028084750
C     -0.524567597    3.209115348    3.821052390
C      0.612007736    4.062910838    3.315698770
C     -0.481639753    2.618811082    5.086108902
C     -1.557668668    1.875145883    5.585836071
C     -1.473327017    1.217668905    6.943940292
C     -2.695162581    1.734261459    4.788168065
C     -2.769415190    2.288504019    3.506595193
C     -3.997596067    2.092860701    2.650239121
H      1.441730243    4.080117905    4.027933522
H      0.978787662    3.694572247    2.353325136
H      0.283476739    5.096944710    3.154045883
H      0.404575730    2.756408701    5.702957662
H     -1.070321123    0.200124737    6.863051651
H     -0.819792283    1.776540114    7.621306862
H     -2.458228833    1.135160496    7.415016267
H     -3.546323693    1.171683094    5.165003114
H     -4.694019408    2.936450774    2.744631817
H     -3.726326620    2.005796024    1.594058335
H     -4.536834560    1.187769567    2.940918204
S      2.138108776    2.383834639    0.142992355
C      3.141805476    1.026650089   -0.135899253
N      2.999996726    0.089996648   -1.107750349
C      3.924079218   -0.932380914   -0.946161078
C      4.692023414   -0.610700004    0.122573860
N      4.212609589    0.609493993    0.612459402
H      2.134492625    0.066492182   -1.640760859
H      3.953032823   -1.788192783   -1.600776637
H      5.499141375   -1.126708379    0.617726718
C      4.597386643    1.208279335    1.883077873
H      4.411098636    2.280138296    1.823453125
H      5.654519343    1.002719728    2.056991328
S      5.654981431   -1.356367065    3.550901175
C      4.250650291   -0.438308887    3.723381260
N      3.846659815    0.661688337    2.994276628
C      2.590984275    1.103186281    3.424820737
C      2.219001297    0.301418108    4.446085557
N      3.243590871   -0.619555588    4.625983373
H      2.064598806    1.886541982    2.909506483
H      1.301526102    0.279297873    5.010330259
H      3.239939372   -1.405993236    5.255744273
C     -1.387886339   -0.348367492   -0.454789550
C     -0.134156124   -0.284319869   -1.259579596
H     -1.604994496   -1.306882942    0.015492834
H     -2.304382214    0.053188664   -0.889022634
H      0.506322361   -1.172831723   -1.169848968
H     -0.305781539   -0.019867369   -2.309079699
```

6f$_{cis}$

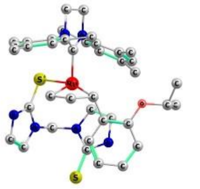


```
100
RuC40N6H50S2O
Ru     0.477836712    1.583084046    1.068036680
C     -0.718514536   -0.021813870    0.258450129
H     -1.800864466    0.092423425    0.309977349
C     -0.293717864   -1.288201202    0.909590146
C     -1.150793729   -2.011645178    1.791406958
C     -0.775995896   -3.246987262    2.325434469
C      0.472275916   -3.809095556    2.016299474
C      1.343630253   -3.116511039    1.181614622
C      0.957477576   -1.886500207    0.645744154
H     -1.452561675   -3.797380834    2.968729723
H      0.745612135   -4.775924796    2.429997575
H      2.325909696   -3.519627258    0.957230739
H      1.641780115   -1.381121588   -0.026512185
O     -2.373570179   -1.429367343    2.031346986
C     -3.369052624   -2.100263439    2.815248459
C     -4.708199429   -1.505890759    2.391698975
C     -3.091335131   -1.907387019    4.308519478
H     -3.366343618   -3.170257561    2.565603557
H     -4.865893061   -1.652187832    1.319746024
H     -5.527703825   -1.985314033    2.937153345
H     -4.733670007   -0.432550070    2.599846451
H     -2.101482943   -2.283028510    4.581942441
H     -3.134129856   -0.842352581    4.555884461
H     -3.836424853   -2.433275135    4.914579008
C     -0.659903885    3.113306882    0.818441807
N     -1.191025964    3.790629376   -0.258689741
C     -1.806871421    5.077170107    0.102668378
C     -2.004952126    4.934029173    1.612624455
N     -1.060368428    3.861179447    1.925992232
H     -1.132338745    5.909009171   -0.145017416
H     -2.743326097    5.227236200   -0.443512278
H     -1.763502889    5.847883011    2.165508058
H     -3.034126889    4.640856362    1.870342415
C     -1.015076656    3.529142207   -1.650400108
C     -2.052309853    2.871531861   -2.332934450
C     -3.247439508    2.349272424   -1.572842289
C     -1.931901720    2.682300069   -3.711162678
C     -0.812415233    3.135501946   -4.416060769
C     -0.718095887    2.939485266   -5.911874281
C      0.199745301    3.792020652   -3.709159705
C      0.116102727    4.007764866   -2.329421171
C      1.224472099    4.704107174   -1.578203455
H     -3.920343532    1.793451037   -2.231455030
H     -3.821432788    3.163080770   -1.113344575
H     -2.928851663    1.686526363   -0.762732239
H     -2.722379308    2.158354740   -4.244665880
H      0.321795769    2.934743789   -6.252712125
H     -1.235826336    3.744553246   -6.449437178
H     -1.179737851    1.995713977   -6.220274717
H      1.080064691    4.143036565   -4.244323458
H      1.547255207    4.110913107   -0.716782519
H      2.085314943    4.883090988   -2.229945515
H      0.900435559    5.677850183   -1.190658140
C     -0.913955431    3.412126256    3.270315469
C     -0.007261277    4.083322547    4.116058511
C      0.752856899    5.298672495    3.643350638
C      0.152525580    3.624292302    5.426465869
C     -0.575828591    2.535841490    5.917586894
C     -0.308554909    1.980531521    7.295605225
C     -1.521750011    1.939330871    5.080217816
C     -1.716151579    2.362649086    3.760479451
C     -2.780146206    1.709825955    2.913155098
H      1.765933061    5.314439716    4.054625251
H      0.826979212    5.325750337    2.555115515
H      0.247529150    6.217178574    3.969879483
H      0.871990822    4.121742159    6.073995138
H      0.404328732    1.147752283    7.233086594
H      0.122713577    2.734651537    7.960933507
H     -1.221000810    1.593779199    7.760994516
H     -2.131247066    1.124113073    5.466093962
H     -3.680740810    1.537216866    3.513332146
H     -3.052613332    2.319989513    2.050467018
H     -2.439357820    0.747942348    2.524733797
S      2.524408104    3.117508398    1.822928126
C      3.695163926    1.974158920    1.361945824
N      4.154231203    1.731067206    0.107307219
C      5.037722211    0.659603072    0.097087869
C      5.162624531    0.244964809    1.380881692
N      4.345103625    1.066400913    2.156978068
H      3.738374634    2.180972291   -0.695404932
H      5.490609576    0.293340459   -0.809629819
H      5.708161837   -0.571758994    1.823857962
C      4.100780487    0.916872634    3.589714988
H      3.968147298    1.911973289    4.014411132
H      4.965937157    0.412694612    4.022987838
S      4.198870701   -2.298851700    3.584220458
C      2.899856984   -1.248469338    3.810310767
N      2.921698568    0.133367821    3.869135591
C      1.630135234    0.636290349    4.071180934
C      0.803747771   -0.433108358    4.144386623
N      1.588980474   -1.567251259    4.000723366
H      1.435236771    1.691953836    4.143695135
H     -0.265149680   -0.478251689    4.259370009
H      1.236932154   -2.496633810    3.803757372
C     -0.127313119    0.206088007   -1.153854595
C      1.070418737    1.109943259   -0.853319210
H      0.119803025   -0.731116912   -1.684341246
H     -0.851366256    0.736601296   -1.774127784
H      2.004587012    0.531294202   -0.732522211
H      1.247428236    1.899050173   -1.588963750
```

7f‡ (391.01*i* cm^{-1})

100
RuC40N6H50S2O

Ru	-0.412445458	1.649502375	0.330993098
C	-1.404355538	0.187732161	1.524028471
H	-1.771664387	0.694135076	2.411814800
C	-0.628293600	-1.046874360	1.811989470
C	0.241561369	-1.133726652	2.926749309
C	1.122768833	-2.206678139	3.082171250
C	1.104698694	-3.283482549	2.198321652
C	0.175675246	-3.283139122	1.159498845
C	-0.665256245	-2.187082796	0.981552004
H	1.826188032	-2.187667027	3.907714174
H	1.796147995	-4.110676469	2.332408700
H	0.101488999	-4.132973554	0.485441539
H	-1.383027979	-2.221143801	0.170367124
O	0.229075844	-0.133489193	3.890102676
C	-0.107972241	-0.601659433	5.218586777
C	-1.576942225	-1.017333446	5.273550459
C	0.227984177	0.521158838	6.188631508
H	0.518096071	-1.472922818	5.457495742
H	-1.770064132	-1.826064092	4.563632584
H	-1.841081527	-1.368766568	6.277322051
H	-2.214181523	-0.168542391	5.012758750
H	1.281417141	0.805855072	6.125111328
H	-0.370934508	1.402374532	5.964932142
H	0.014152557	0.206858721	7.215737688
C	-1.199485056	3.417323499	1.003958696
N	-0.887966712	4.503543143	0.198897356
C	-1.865008559	5.599247050	0.278491058
C	-2.585246636	5.263376442	1.589918616
N	-2.255651704	3.835497959	1.767534768
H	-1.366909612	6.573713488	0.299540306
H	-2.552342623	5.585358020	-0.578665197
H	-2.197535761	5.844548723	2.438518692
H	-3.667575616	5.415745033	1.539775469
C	-0.150026281	4.239854006	-1.003055034
C	-0.777638031	3.632173908	-2.108960093
C	-2.269103798	3.380823546	-2.168468968
C	0.011756025	3.304999670	-3.220667918
C	1.378242798	3.576301342	-3.258776707
C	2.219683636	3.191442062	-4.453304591
C	1.961742453	4.232050950	-2.166086052
C	1.217793501	4.578004938	-1.040105508
C	1.849193565	5.290852190	0.128451086
H	-2.482691416	2.462973404	-2.723637965
H	-2.778194908	4.204732909	-2.686695596
H	-2.713158994	3.273518936	-1.178860924
H	-0.459140878	2.813559953	-4.069695319
H	3.143159276	2.692046984	-4.139949874
H	2.512182789	4.074894215	-5.033937416
H	1.681729920	2.515222530	-5.124067793
H	3.024507027	4.463932632	-2.188992393
H	1.720657341	4.716369184	1.048438730
H	2.919596645	5.442202214	-0.035670966
H	1.385194444	6.271600391	0.288019933
C	-2.832178595	3.145619340	2.872492144
C	-2.117232590	3.023363782	4.079576031
C	-0.673893333	3.453850433	4.164552765
C	-2.770117192	2.456927639	5.176849750
C	-4.080391636	1.970349332	5.093037716
C	-4.722547159	1.295358234	6.282216103
C	-4.753196583	2.086313791	3.876439294
C	-4.155614580	2.684537360	2.762113132
C	-4.942555014	2.840614352	1.481767742
H	-0.367261853	3.606743765	5.203580846
H	-0.035879322	2.681667495	3.718460265
H	-0.488678313	4.378638216	3.610256087
H	-2.245502590	2.387486356	6.126450376
H	-5.810065713	1.239922401	6.177056237
H	-4.348463599	0.269578573	6.394295453
H	-4.497284953	1.824447892	7.214495897
H	-5.769806173	1.707932316	3.788899790
H	-5.538648407	3.763007288	1.491064652
H	-4.286585800	2.885297509	0.609785566
H	-5.639403359	2.007490353	1.348337535
S	1.740549203	1.951575113	1.382460894
C	2.582410004	0.548943720	0.861152722
N	2.112664249	-0.390519615	0.016934471
C	3.034648154	-1.411297063	-0.139201841
C	4.117400794	-1.097367795	0.614063358
N	3.837242328	0.131233950	1.221622140
H	1.158383881	-0.255885214	-0.382030900
H	2.831909614	-2.272337969	-0.754099700
H	5.046022897	-1.611723252	0.809650191
C	4.660494875	0.775611817	2.222241009
H	4.350095004	1.818835469	2.295003854
H	5.711394535	0.702522025	1.936769274
S	6.917981011	-1.245857850	3.380453366
C	5.449024659	-0.748687744	4.035174040
N	4.524636358	0.136151350	3.516216573
C	3.398093528	0.219258675	4.335153186
C	3.628677798	-0.582455106	5.400988033
N	4.883098221	-1.154165671	5.211805575
H	2.523124764	0.780653687	4.041969431
H	3.016647412	-0.804128860	6.259532583
H	5.348175726	-1.811829524	5.817651422
C	-2.226623950	0.274956717	0.301623586
C	-0.774421610	0.265563847	-1.064440988
H	-2.633444538	-0.620994875	-0.161577182
H	-2.999981760	1.040211695	0.314041710
H	-1.010300023	0.730799210	-2.033724500
H	-0.830331410	-0.820819789	-1.188360008

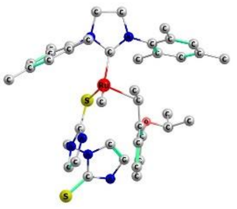

8f

100
RuC40N6H50S2O

Ru	-0.461234273	1.552095017	0.411726366
C	-1.455785790	-0.167564386	1.203542959
H	-1.548408364	0.107447873	2.255321364
C	-0.910051040	-1.530418855	1.043530697
C	-0.242938567	-2.152152410	2.124451370
C	0.303739742	-3.432833427	2.010862687
C	0.197848625	-4.143797495	0.816059927
C	-0.459934807	-3.550555620	-0.268295150
C	-1.001268251	-2.273269518	-0.150025607
H	0.832525819	-3.851673585	2.862453111
H	0.622842016	-5.140125560	0.731589797
H	-0.557707716	-4.089790592	-1.207245336
H	-1.499597988	-1.823169873	-1.003016021
O	-0.074511105	-1.438088626	3.296242991
C	-0.737846216	-1.953389517	4.468312675
C	-2.255679414	-1.839419039	4.331887959
C	-0.187524684	-1.145221779	5.634889813
H	-0.466499699	-3.012345549	4.596415952
H	-2.601317178	-2.371998670	3.441405398
H	-2.752107124	-2.273418151	5.207051039
H	-2.554088583	-0.790988379	4.242714441
H	0.899313563	-1.252268455	5.693336343
H	-0.421364842	-0.086653111	5.485014460
H	-0.631878126	-1.477722743	6.579064996
C	-1.253824835	3.451426740	0.613469900
N	-1.176486256	4.454365761	-0.303659237
C	-2.049039208	5.601650832	0.000422062
C	-2.447162516	5.325670603	1.457287342
N	-2.054453828	3.913972280	1.618517992
H	-1.507727214	6.544145708	-0.126120170
H	-2.916837073	5.615985157	-0.672376605
H	-1.896728758	5.955810224	2.169321971
H	-3.517221288	5.459729687	1.641132989
C	-0.541114408	4.346104130	-1.579795898
C	-1.235036251	3.767740813	-2.656436131
C	-2.624558939	3.209075817	-2.468441735
C	-0.577869762	3.667284928	-3.883570892
C	0.736044431	4.118115418	-4.054913383
C	1.416156313	3.997827693	-5.399162675
C	1.393976555	4.690046734	-2.963302417
C	0.771470960	4.816249863	-1.717493874
C	1.500138904	5.404280743	-0.534428867
H	-3.004674018	2.784869199	-3.401972635
H	-3.335824232	3.975537158	-2.137771513
H	-2.607862666	2.422168762	-1.707194879
H	-1.095500086	3.206081193	-4.722423515
H	2.502941842	4.085677090	-5.308766379
H	1.076961090	4.782910429	-6.086907579
H	1.193133457	3.035739423	-5.872696698
H	2.418972636	5.036169986	-3.078530402
H	1.601147821	4.660602767	0.261638209
H	2.502010326	5.738982659	-0.816697530
H	0.962146581	6.261880211	-0.113322073
C	-2.398474349	3.190359741	2.799847269
C	-1.457949046	3.031237191	3.834863554
C	-0.060560803	3.586329887	3.716614096
C	-1.844400407	2.330890614	4.981396607
C	-3.127004037	1.792515988	5.123420497
C	-3.520237677	1.053930987	6.381813149
C	-4.041865718	1.977723048	4.083282052
C	-3.705226111	2.681122692	2.923545214
C	-4.738546448	2.897914877	1.842553486
H	0.382190981	3.739009101	4.706059311
H	0.581935435	2.902596616	3.151749913
H	-0.046348208	4.538947992	3.179710419
H	-1.124365101	2.209014677	5.788113546
H	-3.847736390	1.751787826	7.163096881
H	-4.344197356	0.358550340	6.195013653
H	-2.680297947	0.479727411	6.785302099
H	-5.043051185	1.561204675	4.173507214
H	-5.316394661	3.813016968	2.028849571
H	-4.275702139	2.991801295	0.857794671
H	-5.449392728	2.067150927	1.809623160
S	1.992183988	1.954458172	1.070526940
C	2.654166008	0.375253542	0.963641321
N	2.116251072	-0.677399924	0.315314809
C	2.891943013	-1.811579935	0.473741601
C	3.962332195	-1.457637396	1.226259541
N	3.818593150	-0.095783818	1.513033504
H	1.206432496	-0.568068112	-0.151245035
H	2.587659750	-2.762109296	0.068096933
H	4.792062401	-2.020959760	1.623566089
C	4.662794485	0.663673041	2.413967832
H	4.582298369	1.718394587	2.148487191
H	5.691523277	0.313755173	2.318278784
S	6.071513105	-1.536991155	4.338811132
C	4.811170298	-0.462266724	4.637062906
N	4.268501568	0.490387582	3.798141795
C	3.207810819	1.151888134	4.420011017
C	3.098444347	0.640470339	5.666682428
N	4.081992572	-0.335838172	5.787079373
H	2.627848091	1.891358498	3.891763356
H	2.412014058	0.872354964	6.464314569
H	4.266171593	-0.915957063	6.590472858
C	-2.338036915	0.526652696	0.298567729
C	-0.046499177	0.968741194	-1.283837949
H	-2.561447762	0.088174561	-0.675850186
H	-3.191421011	1.043663365	0.727270653
H	0.921227419	1.256519566	-1.717266067
H	-0.765149521	0.538822531	-1.997139541

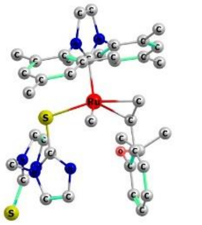

9f

100
RuC40N6H50S2O

Ru	-1.596712985	1.760888425	2.096341166
C	-0.859574177	-3.154450127	5.196952925
H	-0.754151840	-2.199223167	5.704742459
C	0.279630772	-3.550918848	4.353865646
C	1.155400059	-2.558404044	3.845357498
C	2.182472179	-2.914433793	2.961900588
C	2.372826473	-4.249893584	2.608963288
C	1.547182526	-5.243882342	3.134285000
C	0.514801690	-4.886917787	3.996782231
H	2.819698134	-2.151850078	2.533009568
H	3.159573648	-4.510846835	1.908404903
H	1.709582726	-6.285544236	2.874405742
H	-0.122849488	-5.657843334	4.420597913
O	0.888226882	-1.279658175	4.231186424
C	1.835369357	-0.212715329	3.994277827
C	3.118749749	-0.426994395	4.800046802
C	1.117663464	1.059255606	4.412738496
H	2.055995442	-0.168400073	2.918208633
H	3.617195980	-1.367873442	4.555661805
H	3.817482748	0.393679421	4.605166151
H	2.885324294	-0.432554660	5.869576799
H	0.220577251	1.235484043	3.800121835
H	0.819416238	0.993819103	5.464458766
H	1.780123882	1.918961039	4.285610573
C	-1.795865958	3.660752817	1.613807306
N	-0.754408230	4.494659130	1.243223850
C	-1.199966999	5.750069437	0.624389681
C	-2.678405782	5.793459877	1.044367465
N	-2.933345202	4.401994751	1.441532286
H	-0.623254429	6.600622073	1.002611606
H	-1.088493609	5.722023211	-0.468382521
H	-2.845080173	6.478112097	1.887888807
H	-3.343712897	6.093769561	0.228458779
C	0.588196350	4.025245519	1.134207515
C	1.071895260	3.523734501	-0.086658318
C	0.191612036	3.459186546	-1.311273693
C	2.393296390	3.064069695	-0.142340846
C	3.226955042	3.086834039	0.978206706
C	4.658138660	2.608901573	0.893150468
C	2.715190315	3.584333923	2.182522336
C	1.406062622	4.059322213	2.280979993
C	0.853351940	4.574812853	3.586150462
H	0.515086253	2.655732926	-1.978338495
H	0.230876933	4.397118948	-1.879294762
H	-0.850174535	3.271663174	-1.040211199
H	2.772648225	2.666540697	-1.082091922
H	4.805915929	1.935458734	0.043428803
H	4.956443108	2.083071401	1.807650212
H	5.351242339	3.450189436	0.768928748
H	3.352316832	3.604663651	3.064744794
H	0.028594094	3.934813857	3.920118090
H	1.623889257	4.596417472	4.362314334
H	0.445670661	5.585135237	3.471290903
C	-4.265014617	3.926959013	1.625818456
C	-4.917201257	4.139907655	2.848206359
C	-4.178260542	4.778909776	3.996917769
C	-6.233008118	3.687678324	2.988008114
C	-6.893817266	3.023327189	1.950613410
C	-8.301115942	2.501490874	2.131279436
C	-6.208896227	2.813485570	0.748530914
C	-4.896198027	3.253801585	0.566470623
C	-4.140624005	2.961805184	-0.705499051
H	-4.779466056	4.762632792	4.910397470
H	-3.245237358	4.234382320	4.180436043
H	-3.915159236	5.822986337	3.788753101
H	-6.746695358	3.842918441	3.934801313
H	-8.832516762	3.039921404	2.922073001
H	-8.883980132	2.592424259	1.208415501
H	-8.294547801	1.438744438	2.405571054
H	-6.706981313	2.287099507	-0.063646681
H	-3.709450883	3.871251627	-1.140451355
H	-3.307624720	2.282420759	-0.486573536
H	-4.788763445	2.498056977	-1.454797609
S	-0.645244400	0.543661665	0.252213208
C	-0.667373864	-1.128554730	0.628409501
N	-1.395791417	-1.745435189	1.586974350
C	-1.211468469	-3.120343047	1.542964636
C	-0.347066591	-3.371711989	0.531372704
N	-0.026482505	-2.137176142	-0.040682908
H	-1.993094302	-1.210880275	2.215198546
H	-1.675180432	-3.778048839	2.257524649
H	0.115875511	-4.282269758	0.186404902
C	0.941415436	-1.914023072	-1.097035519
H	0.659489934	-1.006355626	-1.632014686
H	0.942454126	-2.775432688	-1.766212151
S	3.035224159	-4.376969882	-1.097052970
C	3.193721275	-2.806796785	-0.517872771
N	2.287547052	-1.766594944	-0.579682708
C	2.802464665	-0.619961946	0.030041871
C	4.046897053	-0.930595305	0.463798260
N	4.270916403	-2.261136860	0.125183649
H	2.225697440	0.289718490	0.108734087
H	4.779162400	-0.331172376	0.977982169
H	5.108689767	-2.795985383	0.292743237
C	-2.000113746	-3.844941070	5.346136611
C	-3.178445410	1.163615412	2.748353241
H	-2.184903758	-4.786751368	4.834441115
H	-2.791674147	-3.486177559	5.996556712
H	-4.227895166	1.124342329	2.442373268
H	-2.914244161	0.655057777	3.706805756

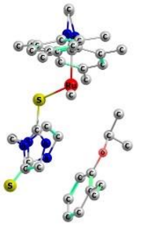

1g

94
RuC38N6H46S2O

Ru	0.412419418	1.139423225	0.680568073
N	-1.232518493	3.388723757	-0.410296329
N	-0.768732764	3.921492721	1.656275887
C	0.788010925	0.262317345	-0.973956747
C	-0.629720652	2.910238842	0.724417711
C	-1.273605274	2.760075056	-1.695480245
C	-2.422402881	2.020570749	-2.031172101
C	-3.515128801	1.813954109	-1.011138590
C	-2.509048479	1.459011976	-3.304203619
C	-1.485489437	1.619419398	-4.243837913
C	-1.569874160	0.933377726	-5.585761076
C	-0.367020168	2.372789408	-3.886441948
C	-0.237730943	2.951101614	-2.618739082
C	1.026040779	3.694209524	-2.267998532
C	-1.690708693	4.781922156	-0.318654025
C	-1.573975590	5.064729579	1.181460627
C	-0.705018555	3.748437988	3.075278083
C	0.346836415	4.339659581	3.792878365
C	1.466432429	5.038233924	3.063173307
C	0.345310377	4.239260473	5.187367702
C	-0.671680631	3.572542426	5.875455584
C	-1.710588909	3.001783973	5.135983560
C	-1.746606042	3.074975250	3.741822637
C	-2.846429235	2.398376551	2.962371347
C	-0.622126101	3.426075904	7.378979795
H	1.598561558	0.497752434	-1.677922158
H	2.218111149	5.411578972	3.765497526
H	1.945326817	4.350584333	2.358640263
H	1.099830876	5.889479917	2.477433888
H	1.164182260	4.685431531	5.748705918
H	-0.064112636	2.524689668	7.665480073
H	-0.124172635	4.278828552	7.852185400
H	-1.625371420	3.337931171	7.808924891
H	-2.507000334	2.472174494	5.655906497
H	-3.414593493	3.104723588	2.346396444
H	-2.416536470	1.660750376	2.278401961
H	-3.549459533	1.893144307	3.630719414
H	-1.077966006	6.015443393	1.404703437
H	-2.552731977	5.073918843	1.680142716
H	-1.043458802	5.435659455	-0.919495699
H	-2.712935353	4.883383120	-0.699512950
H	-3.952168507	2.764736033	-0.681953743
H	-3.110476711	1.317262933	-0.125478295
H	-4.317877809	1.194021867	-1.419916200
H	-3.376879465	0.855105023	-3.558907333
H	-2.581316376	0.986432089	-6.003844947
H	-1.317768408	-0.128733779	-5.477272511
H	-0.877429750	1.371937267	-6.311390108
H	0.441339514	2.501827202	-4.604427577
H	0.930660268	4.271331122	-1.346764071
H	1.838153296	2.978235986	-2.104114776
H	1.324597007	4.368835391	-3.078133464
C	-0.205207002	-0.639353804	-1.518854468
C	-1.381267952	-0.916238675	-0.780425365
C	-0.113514317	-1.247614576	-2.793430818
C	-2.428415612	-1.668528544	-1.297137871
C	-1.131583454	-2.049262889	-3.300209413
H	0.775858161	-1.053166961	-3.388433347
C	-2.302738220	-2.251966187	-2.563458431
H	-3.342249123	-1.796547765	-0.731376003
H	-1.023692304	-2.503482831	-4.282923538
H	-3.114525151	-2.852697796	-2.963898599
O	-1.433079744	-0.301271546	0.479423193
C	-1.761446680	-1.109684974	1.656097089
C	-1.008578066	-2.435724135	1.646818612
C	-3.268568332	-1.262333034	1.865058871
H	-1.371928410	-0.485735060	2.461779851
H	0.046226523	-2.255646379	1.425577226
H	-1.083793921	-2.908030800	2.632248766
H	-1.408291058	-3.125109062	0.898539559
H	-3.778142837	-0.312915200	1.681542941
H	-3.710307295	-2.029659503	1.224251366
H	-3.455900733	-1.553932400	2.905228695
S	0.661764004	0.896892771	3.390299357
S	2.500127688	2.264124004	0.611657653
N	3.789695913	0.169422642	1.982667970
C	3.670686641	1.475703968	1.552200008
C	4.922394265	0.028674966	2.787733554
C	5.535218990	1.231072372	2.845762768
N	4.772830277	2.100667246	2.079243412
H	4.918302663	3.091380058	1.966783390
H	6.435192407	1.541240938	3.351386737
H	5.175829286	-0.916627143	3.239845220
C	1.264075280	-0.669587445	3.586997357
N	2.200988263	-1.390181171	2.872052087
C	2.370281619	-2.656321812	3.431517342
H	3.045656907	-3.378209706	3.000435501
C	1.548927140	-2.744048673	4.503730604
H	1.379008149	-3.551459151	5.197360876
N	0.885365283	-1.531069169	4.587775193
H	0.199936540	-1.256086981	5.274185662
C	2.877446595	-0.913347596	1.680936781
H	3.427564477	-1.752466269	1.248914921
H	2.119006908	-0.537385869	0.955167435

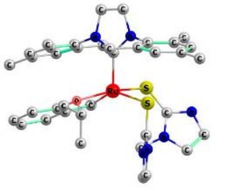

1h

94
RuC38N6H46S2O

Ru	0.320764284	1.212524530	0.989112187
C	0.819494840	0.355282172	-0.616983776
H	1.652121671	0.683924327	-1.261217355
C	-0.102558177	-0.536157054	-1.275002130
C	-1.295626183	-0.898992296	-0.610912906
C	-2.283491285	-1.672906345	-1.200793750
C	-2.080856582	-2.181931600	-2.489026023
C	-0.889362186	-1.886556748	-3.161713178
C	0.071069298	-1.069001201	-2.575411453
H	-3.204203074	-1.883137441	-0.664108803
H	-2.842725040	-2.800437424	-2.954533027
H	-0.720295606	-2.286096472	-4.159412457
H	0.972254194	-0.806722524	-3.124823653
O	-1.432954171	-0.385660918	0.689746968
C	-1.664048011	-1.404249850	1.713910270
C	-2.010062659	-0.681897263	3.004681961
C	-0.446157570	-2.313989695	1.830329249
H	-2.530869372	-1.994694729	1.393268975
H	-2.927459225	-0.100992403	2.891977012
H	-2.171941127	-1.419449176	3.799905833
H	-1.205334713	-0.004255673	3.306956794
H	-0.235464109	-2.796916014	0.872215314
H	0.428698983	-1.726163716	2.119122276
H	-0.627875619	-3.093323694	2.578998970
C	-0.635241279	2.985232321	0.772161057
N	-1.070126233	3.451625040	-0.439502565
C	-1.459029079	4.869418746	-0.437241064
C	-1.495405282	5.197553654	1.057850763
N	-0.812302817	4.033682125	1.653217924
H	-0.718587232	5.473770777	-0.977581040
H	-2.427081567	5.003409330	-0.932426579
H	-0.973883450	6.127285473	1.308609493
H	-2.521216154	5.277098079	1.441802026
C	-1.126472764	2.752037129	-1.684487597
C	-2.297935953	2.043109009	-2.000683544
C	-3.396060137	1.880758030	-0.978608905
C	-2.406636278	1.465879393	-3.266873671
C	-1.382735470	1.575074420	-4.210283667
C	-1.508055714	0.892228165	-5.550489847
C	-0.223981248	2.273327226	-3.859780033
C	-0.073833265	2.868937810	-2.603881154
C	1.198447733	3.586103676	-2.226150040
H	-3.707324494	2.842491673	-0.555471373
H	-3.045872446	1.261355810	-0.147107331
H	-4.273207623	1.398837096	-1.420125064
H	-3.300664450	0.897845296	-3.513802169
H	-2.522302780	0.981757565	-5.954949698
H	-1.293388833	-0.178206488	-5.446694871
H	-0.810657038	1.305647320	-6.285942640
H	0.589036058	2.350309888	-4.579583961
H	1.040066207	4.663593478	-2.093295928
H	1.589190389	3.195871391	-1.281156279
H	1.960439322	3.461258058	-3.001885280
C	-0.909167463	3.895867649	3.075497986
C	0.062144994	4.486842859	3.896505963
C	1.300041564	5.124590271	3.318383713
C	-0.112137077	4.425250997	5.283465835
C	-1.205585861	3.778137661	5.860790440
C	-1.308255104	3.620850687	7.358492584
C	-2.169014920	3.217139545	5.016615165
C	-2.048556393	3.276166112	3.628426929
C	-3.121331313	2.698373599	2.737020667
H	1.702449037	5.885242529	3.994147947
H	2.070225015	4.360237625	3.166551905
H	1.113826646	5.583422526	2.344391615
H	0.645222949	4.867282037	5.927575045
H	-0.802151042	2.698422496	7.671218741
H	-0.832306623	4.452741576	7.887742387
H	-2.350018590	3.555015624	7.689444666
H	-3.032550498	2.717443565	5.451374444
H	-3.620075607	3.475310985	2.144844962
H	-2.696918109	1.984685910	2.027033912
H	-3.889337457	2.191357258	3.328163794
S	2.448156575	2.254182645	1.342223815
C	3.429249878	0.854085936	1.244287743
N	3.972838788	0.337193748	0.098610381
C	4.736206627	-0.783636911	0.363809413
C	4.759175559	-0.931727185	1.709308217
N	3.978380517	0.090311863	2.255452961
H	3.602546037	0.605578250	-0.801826013
H	5.217331389	-1.354013916	-0.414302341
H	5.295749277	-1.632738130	2.328068127
C	3.662294731	0.225309187	3.661647528
H	3.284865307	1.234534252	3.844336847
H	4.577258949	0.050583443	4.236141558
S	0.859038254	1.245741857	4.962395902
C	1.425249754	-0.297044512	4.656818624
N	2.630755671	-0.696542528	4.102819829
C	2.713501603	-2.089556740	4.068177187
C	1.565315698	-2.576884001	4.587824049
N	0.794248618	-1.481431158	4.943026574
H	3.563162789	-2.606458742	3.655753940
H	1.228012629	-3.592261557	4.716977442
H	-0.136382601	-1.493826230	5.328674174

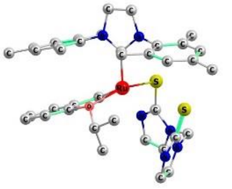

Stereoselectivity

9a

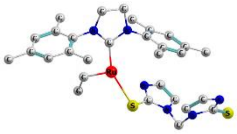

77
RuC30N6H38S2

Ru	-1.525622301	2.768479954	2.246741011
C	-1.606217620	4.203153754	0.916418964
N	-0.446164783	4.575498440	0.258946471
C	-0.629581247	5.670826636	-0.697486880
C	-1.997903734	6.224388936	-0.272669056
N	-2.550100520	5.117261876	0.523126424
H	0.174152003	6.410544111	-0.614884109
H	-0.642725746	5.299642955	-1.732527506
H	-1.901008493	7.132106612	0.339793651
H	-2.647413304	6.459904221	-1.121945436
C	0.739100054	3.779452955	0.284020753
C	0.922009313	2.750044493	-0.658641464
C	-0.178213100	2.409213429	-1.633589694
C	2.115970004	2.024408124	-0.628864162
C	3.110592614	2.275511302	0.323368310
C	4.399423712	1.488307915	0.335090032
C	2.887209679	3.282990254	1.266895250
C	1.719541559	4.052920768	1.257103528
C	1.494497323	5.145206455	2.275482836
H	-0.353491726	3.214894639	-2.356080399
H	-1.114751977	2.246244518	-1.087508169
H	0.063430935	1.504777088	-2.199540798
H	2.267436190	1.230923834	-1.358518282
H	5.224887849	2.082019108	-0.078338083
H	4.318954540	0.577971735	-0.266826872
H	4.687025966	1.193574255	1.349668060
H	3.640991485	3.476141106	2.026261043
H	1.361262840	6.119762174	1.791716873
H	0.586951001	4.950896873	2.858953910
H	2.340769251	5.214390108	2.964342748
C	-3.867839600	5.222387101	1.060138037
C	-4.079169821	5.814251695	2.316432226
C	-2.913112358	6.272281850	3.155669230
C	-5.390799894	5.904286350	2.791994955
C	-6.477882467	5.422515031	2.055594688
C	-7.881217373	5.492688764	2.613362361
C	-6.232648312	4.836462705	0.809769994
C	-4.936915087	4.721560240	0.300021263
C	-4.667833976	4.017342227	-1.007138865
H	-3.254049974	6.773117070	4.066343775
H	-2.300407111	5.403453060	3.431721475
H	-2.260131784	6.962312017	2.609505135
H	-5.566861893	6.354021725	3.767195556
H	-8.014869342	6.368150347	3.256955044
H	-8.629055263	5.540108136	1.815309910
H	-8.107036506	4.605964238	3.219499895
H	-7.066420616	4.444608274	0.230508686
H	-4.188450690	4.678548374	-1.738485829
H	-3.986942495	3.174818872	-0.843544425
H	-5.593640685	3.639545106	-1.450135083
S	-0.238027538	1.858038541	4.209326624
C	0.733639286	0.776684820	3.292353455
N	0.477274575	0.379071381	2.025144843
C	1.484866625	-0.452190132	1.562258855
C	2.377075273	-0.603433227	2.571750525
N	1.900158825	0.155112633	3.646219357
H	-0.290376643	0.884123673	1.517021835
H	1.479957702	-0.854490654	0.563189471
H	3.314216580	-1.132070517	2.641489800
C	2.605068264	0.401569872	4.889595046
H	3.213045661	-0.471496787	5.129730819
H	1.866894765	0.588714095	5.670127404
S	5.696416530	0.100623060	3.978676099
C	4.803427681	1.467549277	4.386675773
N	3.488635502	1.548065070	4.798573457
C	3.120181066	2.875807829	5.022909274
C	4.210984758	3.636774525	4.776803310
N	5.226144631	2.768052516	4.388774981
H	2.109550414	3.135600142	5.294557826
H	4.352020999	4.703604997	4.835403078
H	6.172229364	3.012996765	4.141044945
C	-3.275422109	2.265181117	2.295284827
H	-4.136426602	2.945320793	2.253873986
C	-3.669172397	0.823027341	2.525517900
H	-4.299000350	0.459865797	1.698630417
H	-2.808546217	0.150790638	2.612972204
H	-4.265475987	0.725725805	3.446608105

9a’

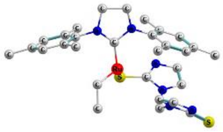

77
RuC30N6H38S2

Ru	0.840121738	2.280024967	0.910876693
C	1.021431956	1.628867693	-0.795553670
H	0.572138956	2.018183065	-1.717334172
C	-0.839104942	3.215250125	0.670581617
N	-1.384664916	3.978582818	-0.337782219
C	-2.530605392	4.789801120	0.104794692
C	-2.915249875	4.125562987	1.436173643
N	-1.705745803	3.361199079	1.740999196
H	-2.232164524	5.839283146	0.241926176
H	-3.338680816	4.765794157	-0.633499451
H	-3.141151112	4.852391104	2.224617133
H	-3.788037287	3.464704988	1.327001465
C	-0.707661827	4.348357695	-1.540900747
C	-1.126771701	3.758950038	-2.744576050
C	-2.201910687	2.699269645	-2.731364496
C	-0.496517380	4.136400582	-3.933251102
C	0.536787935	5.078272571	-3.944010718
C	1.241939056	5.442391571	-5.230549974
C	0.927094928	5.652235892	-2.730243816
C	0.321660150	5.305425062	-1.518981817
C	0.801793162	5.904833080	-0.221500960
H	-2.350700063	2.276649198	-3.729081028
H	-3.164065334	3.097599094	-2.387824176
H	-1.928809139	1.890783083	-2.044835290
H	-0.810029760	3.676034152	-4.868164640
H	0.598059964	5.283834939	-6.101417840
H	2.141010910	4.829092441	-5.372474032
H	1.561796608	6.489665120	-5.230620180
H	1.730025191	6.386957498	-2.724221128
H	1.319208552	5.135140764	0.364035343
H	1.487266111	6.738076426	-0.401665817
H	-0.026188987	6.269277766	0.396221051
C	-1.504817660	2.678035208	2.976405417
C	-0.792930968	3.332151878	3.998616233
C	-0.248737599	4.723386396	3.775270147
C	-0.583885588	2.657980066	5.205653093
C	-1.056042245	1.359391660	5.413466132
C	-0.764620564	0.615462982	6.695186796
C	-1.777055065	0.742279275	4.384705642
C	-2.009376694	1.377980993	3.164090922
C	-2.722087267	0.680283978	2.033796836
H	0.241528951	5.099876104	4.678508959
H	0.476038251	4.728961271	2.953224649
H	-1.041857861	5.429145807	3.505356047
H	-0.031027405	3.154333945	5.999219618
H	0.016113438	-0.140921815	6.542290213
H	-0.420595459	1.291734999	7.483536541
H	-1.653225585	0.091450383	7.063927207
H	-2.146339166	-0.270341863	4.531009590
H	-3.611916547	1.231118373	1.710242301
H	-2.051422959	0.610562516	1.168715747
H	-3.038733374	-0.324570396	2.326610119
S	2.924433545	2.980992871	2.032043802
C	2.825047634	2.449554545	3.661492055
N	2.845135978	3.265167358	4.750255803
C	2.767586524	2.536976861	5.929312070
C	2.698312664	1.232579954	5.575201373
N	2.730019318	1.183970426	4.181104483
H	2.888597890	4.268148327	4.655508057
H	2.759816703	3.005302915	6.899785511
H	2.618501109	0.323233913	6.152219430
C	2.798451834	-0.043089570	3.396070093
H	3.583765879	-0.675401555	3.816864392
H	3.038936950	0.246392675	2.372970529
S	2.171732167	-2.268061002	5.674670702
C	1.296551340	-1.810245713	4.304556216
N	1.570911606	-0.810558462	3.393028331
C	0.602538574	-0.777844117	2.384519106
C	-0.282052378	-1.760986707	2.664570302
N	0.148725555	-2.376671400	3.836188106
H	0.572461230	0.031492339	1.638409251
H	-1.187096082	-2.050445002	2.157167402
H	-0.281267181	-3.156239439	4.309178050
C	1.975203463	0.482734524	-1.059557822
H	2.434826061	0.082218107	-0.145425483
H	1.459167476	-0.351266470	-1.560490842
H	2.794375478	0.798558101	-1.724196358

10a$_{trans}$

86

RuC33N6H44S2

Ru	-2.102060915	1.965740888	2.318791827
C	-1.618511680	3.674938333	1.247876887
N	-0.533743016	3.980628999	0.487126002
C	-0.690231719	5.220969012	-0.299051617
C	-1.971538078	5.825464502	0.297553158
N	-2.505315545	4.694057472	1.077786541
H	0.183352739	5.869869030	-0.187183566
H	-0.798712247	4.981088868	-1.364847053
H	-1.766232362	6.681128001	0.955146681
H	-2.686537404	6.150585329	-0.464097898
C	0.705010989	3.262545766	0.481039043
C	0.930001057	2.229438955	-0.439290734
C	-0.121291970	1.799111623	-1.431439713
C	2.159145294	1.558510536	-0.397506164
C	3.150282804	1.886021044	0.528249934
C	4.457529154	1.133053441	0.585421926
C	2.907420808	2.945217147	1.411982439
C	1.705428505	3.653407415	1.394038963
C	1.479644676	4.821406397	2.323254543
H	-0.776408361	2.623134442	-1.724208698
H	-0.764856471	1.031517323	-0.989786542
H	0.339417121	1.383935395	-2.333206408
H	2.338157547	0.750611335	-1.104630320
H	5.299348546	1.780093255	0.309868022
H	4.459013282	0.278273419	-0.097418177
H	4.657233136	0.758059944	1.595497401
H	3.672240863	3.220876092	2.134201294
H	1.431733612	5.768206593	1.769768335
H	0.543602354	4.705892698	2.873360611
H	2.292870279	4.899609871	3.048601839
C	-3.716415169	4.826714801	1.830578570
C	-3.658263443	5.258320339	3.167440686
C	-2.333896631	5.542589767	3.835584916
C	-4.853147195	5.372520833	3.883323676
C	-6.089686852	5.070919643	3.304269170
C	-7.361972913	5.133235887	4.117273137
C	-6.115291994	4.672431680	1.964944847
C	-4.945124546	4.543893868	1.212140247
C	-4.999558758	4.048591353	-0.210854632
H	-2.481037749	6.001419289	4.817245504
H	-1.762415715	4.618602944	3.973924334
H	-1.707172592	6.213078302	3.237588050
H	-4.815905815	5.691709552	4.922894796
H	-7.304122977	5.896266200	4.900076860
H	-8.230995694	5.353999335	3.489274435
H	-7.553379286	4.172592316	4.612474471
H	-7.069878574	4.439795780	1.497108510
H	-4.452542617	4.701506385	-0.899427762
H	-4.539784221	3.057775885	-0.274335067
H	-6.032480625	3.975891519	-0.562508931
S	-0.046039944	2.260726126	4.314528163
C	0.841404845	0.991601520	3.594507264
N	0.525643227	0.362170564	2.445162448
C	1.447121775	-0.625803235	2.153944944
C	2.371118750	-0.621453198	3.146973431
N	1.985152555	0.378016104	4.045345556
H	-0.378091680	0.648542257	1.971896000
H	1.377227950	-1.227252293	1.262687191
H	3.278935042	-1.182127676	3.300850711
C	2.682368722	0.742899776	5.257580607
H	3.222239948	-0.129155645	5.628830114
H	1.945949336	1.084856619	5.986740913
S	5.739579241	0.126954449	4.380925860
C	4.947480794	1.588594173	4.639811115
N	3.647447988	1.805679401	5.045202394
C	3.374837088	3.170146121	5.127526579
C	4.512030188	3.823505835	4.794678083
N	5.459665424	2.849168240	4.498292726
H	2.386002832	3.523854595	5.372198064
H	4.727116042	4.877855116	4.735600418
H	6.417522041	2.998256443	4.221780344
C	-2.826266993	1.329384952	0.712765580
H	-2.692756923	1.893270279	-0.224561443
C	-3.561351411	0.052465665	0.370584632
H	-2.972670168	-0.558688438	-0.333083313
H	-3.816000808	-0.580233145	1.222886558
H	-4.498717019	0.294048707	-0.156421666
C	-3.580826265	0.880413625	3.530222817
H	-1.777584755	-0.043609108	4.269341881
C	-2.476173600	0.025539757	3.438551633
H	-4.457925615	0.681986759	2.916762800
H	-2.484879181	-0.835522163	2.776481004
C	-3.866670737	1.708129631	4.766131405
H	-2.947428389	1.927995564	5.320987815
H	-4.538666278	1.164260335	5.447640789
H	-4.354347650	2.656033834	4.511162787

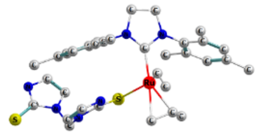

10a’$_{trans}$

86

RuC33N6H44S2

Ru	-1.981338427	2.337374766	2.058110524
C	-1.471850590	4.046049232	0.975396321
N	-0.329997874	4.417025827	0.337110187
C	-0.470524535	5.653141637	-0.458242481
C	-1.788712425	6.223321992	0.074040869
N	-2.369226807	5.048751118	0.748430439
H	0.381175871	6.320385718	-0.304877776
H	-0.521888069	5.406762598	-1.527643348
H	-1.633361727	7.035169315	0.798268474
H	-2.448818631	6.591894981	-0.715203170
C	0.881467835	3.662156050	0.261877166
C	1.004584692	2.606868239	-0.654954832
C	-0.157847527	2.179622559	-1.515419114
C	2.223422365	1.921688313	-0.713976737
C	3.297520528	2.249051368	0.117201203
C	4.594114389	1.477332115	0.071116932
C	3.146493275	3.318446479	1.007660776
C	1.958652449	4.048272376	1.080952583
C	1.833529018	5.233621787	2.006394320
H	-0.690847562	3.036172822	-1.940911765
H	-0.887715948	1.629614253	-0.910252894
H	0.175098247	1.539160067	-2.337435687
H	2.328468321	1.099241580	-1.418972027
H	5.413885076	2.103475603	-0.302087781
H	4.519005746	0.604342686	-0.583914523
H	4.883381442	1.128523738	1.068982563
H	3.972742018	3.586463173	1.662873841
H	1.878725218	6.177709015	1.447858497
H	0.886595555	5.206019703	2.549422119
H	2.646223944	5.240466573	2.735848033
C	-3.690818778	5.103232859	1.289897080
C	-3.881028328	5.329403694	2.665723170
C	-2.710467992	5.527742990	3.598846727
C	-5.184469355	5.338256383	3.164113359
C	-6.293333163	5.136236359	2.335576862
C	-7.687659390	5.084155823	2.915108778
C	-6.074288591	4.960946694	0.968161592
C	-4.786211898	4.947787280	0.423854719
C	-4.602449166	4.778633519	-1.065954899
H	-3.020061227	6.058256676	4.504164311
H	-2.286576724	4.563221234	3.900581655
H	-1.900551049	6.091846079	3.126768607
H	-5.339104139	5.499657838	4.229246677
H	-7.818681031	5.820144552	3.715533854
H	-8.447951807	5.273045453	2.151482908
H	-7.893742760	4.096616771	3.346784996
H	-6.925865183	4.819262526	0.305373066
H	-4.623631052	5.748746016	-1.580582657
H	-3.650153071	4.298321261	-1.300563257
H	-5.405655749	4.170213556	-1.492161511
S	0.244483081	2.738184656	4.008715352
C	1.090427151	1.463051221	3.253776037
N	0.732907202	0.839011110	2.112035828
C	1.619246529	-0.175027461	1.802523986
C	2.569729730	-0.188660314	2.769340530
N	2.233120843	0.823582779	3.672709357
H	-0.179425061	1.116031604	1.653361014
H	1.511157153	-0.774511753	0.914081237
H	3.469656784	-0.767798296	2.897868980
C	2.962748131	1.164525316	4.872835773
H	3.506418314	0.282633795	5.214074433
H	2.246946291	1.497329601	5.626558301
S	5.978363957	0.559134546	3.855068647
C	5.208210169	2.015988792	4.194166878
N	3.926755194	2.227424111	4.656952551
C	3.670022198	3.589806324	4.800790186
C	4.799040151	4.246668607	4.447916257
N	5.725755500	3.277060508	4.078799785
H	2.695035110	3.941980216	5.097215343
H	5.020762675	5.300885644	4.420430233
H	6.674252958	3.429004648	3.773289702
C	-3.069032835	1.899655709	0.598234302
H	-2.963685015	2.476781132	-0.334927387
C	-4.181254361	0.906463916	0.360207032
H	-3.909483759	0.205962305	-0.445889046
H	-4.478648062	0.316659830	1.227201520
H	-5.071622768	1.449144578	0.004200108
C	-3.224802939	1.201004043	3.480555187
H	-1.307327630	0.411923213	3.908600083
C	-2.139799167	0.363209207	3.208375562
H	-3.207416133	1.823215895	4.377098739
H	-4.221522005	0.989397590	3.104880317
C	-2.199512644	-0.943578790	2.450263538
H	-1.269379637	-1.128093664	1.898801475
H	-3.020257734	-0.979713680	1.733641632
H	-2.328733113	-1.786893393	3.145000353

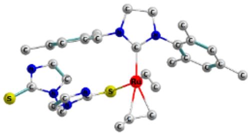

10a$_{cis}$

86

RuC33N6H44S2

Ru	-1.295021632	3.425691844	3.140398342
C	-1.571771828	4.444644555	1.467851058
N	-0.467123092	5.090036089	0.930987000
C	-0.698363151	5.654186336	-0.400680369
C	-2.227168965	5.726628315	-0.435485709
N	-2.607805430	4.771722245	0.618918958
H	-0.218864235	6.632614082	-0.508360331
H	-0.300716634	4.990376341	-1.182537734
H	-2.595346229	6.733059214	-0.193470510
H	-2.652985205	5.433026865	-1.400472601
C	0.862077875	4.868292049	1.405303161
C	1.600708331	3.747534016	0.983964498
C	1.021558616	2.743006360	0.016485596
C	2.905343827	3.594750041	1.459948985
C	3.481404561	4.518288739	2.337147502
C	4.882788543	4.312686224	2.865651026
C	2.729785486	5.629918311	2.726659850
C	1.424996155	5.826225642	2.267926378
C	0.639644911	7.050082133	2.671709027
H	1.217928818	3.039552595	-1.022419305
H	-0.058109196	2.648685527	0.146242461
H	1.468041753	1.755659000	0.165883573
H	3.478860855	2.725608757	1.147239998
H	5.298773663	5.238704677	3.273665699
H	5.558274346	3.961690685	2.077664480
H	4.899608809	3.568192360	3.672117348
H	3.169846223	6.364560465	3.397598107
H	0.333923148	7.634192158	1.796196282
H	-0.273615034	6.775324386	3.204191384
H	1.231984917	7.699293024	3.322317478
C	-4.002214774	4.576327568	0.863346649
C	-4.736598816	5.512374146	1.604475999
C	-4.113484909	6.769694067	2.164119464
C	-6.104865609	5.277530839	1.800436987
C	-6.746146631	4.161218041	1.264724490
C	-8.211564354	3.899815231	1.523932245
C	-5.992933366	3.266363275	0.492681085
C	-4.629371794	3.458666672	0.279165202
C	-3.813437733	2.480651025	-0.530340372
H	-4.440674752	6.940750233	3.194048106
H	-3.024948874	6.720472479	2.165657369
H	-4.416132835	7.644761925	1.575029082
H	-6.678351647	5.989685999	2.390832130
H	-8.727352204	4.800280227	1.870046366
H	-8.719910606	3.542532473	0.621798942
H	-8.340551629	3.128888002	2.293842484
H	-6.480523030	2.395976173	0.058187702
H	-3.366949500	2.960923922	-1.409028293
H	-2.988549708	2.088583585	0.074534922
H	-4.427624149	1.644172695	-0.875451308
S	0.519698319	1.692466186	4.000605494
C	0.374467270	0.449317693	2.840702609
N	-0.537120197	0.386817779	1.844837344
C	-0.349259581	-0.745964138	1.070979400
C	0.701678039	-1.422500491	1.598619295
N	1.134760206	-0.686469710	2.704372592
H	-1.237844608	1.154153524	1.777097094
H	-0.972813108	-0.968739956	0.220337109
H	1.208544787	-2.329175454	1.305777372
C	2.243906416	-1.034956634	3.568867069
H	2.296668691	-2.120800554	3.660491382
H	2.070188076	-0.567665714	4.539498892
S	4.158071079	-2.969217277	1.808729976
C	4.355365666	-1.366342970	2.280991901
N	3.515584270	-0.580689163	3.044834376
C	4.022550087	0.713441324	3.170509184
C	5.203044342	0.740072671	2.509667715
N	5.396097700	-0.531430390	1.981122560
H	3.465343377	1.482588554	3.682824627
H	5.910444307	1.540134042	2.370745030
H	6.176731959	-0.845068809	1.425988755
C	-2.771137837	2.299887296	2.897251751
H	-3.666621125	2.545938018	2.318414870
C	-3.048809144	1.127462527	3.817911464
H	-3.352104721	0.237482696	3.241733211
H	-2.191882686	0.854196529	4.441270301
H	-3.892036936	1.360275070	4.487045509
C	-2.049484514	5.034473586	4.406652536
H	-2.022343219	5.988096175	3.883414217
C	-0.839603838	4.577009916	4.987098507
H	0.088486714	5.132594445	4.862670159
H	-0.868553607	3.918672891	5.854106803
C	-3.396928215	4.729933777	5.033795805
H	-4.192290115	4.693609259	4.283471049
H	-3.381428613	3.762288280	5.546258929
H	-3.662742057	5.497417077	5.777149415

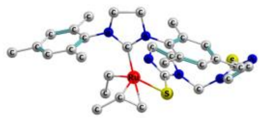

10a'$_{cis}$

86

RuC33N6H44S2

Ru	-1.281296748	3.370382482	2.674574640
C	-1.682974240	4.563248903	1.157209420
N	-0.575394702	5.109545036	0.541933261
C	-0.865829103	5.716722617	-0.758451381
C	-2.376275658	5.956226147	-0.628814624
N	-2.754685596	4.981925789	0.411455760
H	-0.294610180	6.638113737	-0.907798052
H	-0.631399788	5.024568431	-1.580878676
H	-2.600791065	6.977652814	-0.289612720
H	-2.924087565	5.775039152	-1.557979433
C	0.726675346	4.686083717	0.958830640
C	1.242419110	3.443635127	0.517376084
C	0.506996912	2.597417234	-0.499304663
C	2.483620364	3.031797315	1.001022899
C	3.226486636	3.818711775	1.890078403
C	4.564867405	3.332136327	2.392137957
C	2.710700456	5.053596976	2.279569218
C	1.467590725	5.510825426	1.823647395
C	0.977342168	6.874029953	2.247156754
H	0.738794931	2.928282893	-1.520677088
H	-0.574871761	2.644967207	-0.360900077
H	0.810064284	1.549591013	-0.417146259
H	2.879094397	2.068782308	0.683920410
H	4.957294761	3.978290722	3.183128846
H	5.305597859	3.305138058	1.583345792
H	4.482669071	2.315511559	2.790338684
H	3.281799482	5.681835440	2.960135543
H	1.610010985	7.659963380	1.816223402
H	-0.050088919	7.045566165	1.926174364
H	1.017677487	6.980783846	3.335520957
C	-4.132115221	4.831965947	0.763192368
C	-4.711310567	5.652874679	1.742658415
C	-3.935502107	6.774808708	2.391624931
C	-6.058362087	5.450842570	2.065887703
C	-6.835101892	4.486825158	1.420133569
C	-8.274048195	4.250136568	1.816165383
C	-6.243477335	3.727516552	0.404358142
C	-4.901428821	3.887538825	0.058311858
C	-4.287732271	3.074047970	-1.057708760
H	-4.308102004	6.983039264	3.398327551
H	-2.872625898	6.545398188	2.467574203
H	-4.038653473	7.698075226	1.806429976
H	-6.510570756	6.069021856	2.838931657
H	-8.718898606	5.140601212	2.270839009
H	-8.885998280	3.966888999	0.953320067
H	-8.348979967	3.435988804	2.548190943
H	-6.840791175	2.987419583	-0.124335139
H	-4.079320743	3.697288998	-1.936780965
H	-3.338703448	2.627436851	-0.747648387
H	-4.961082401	2.272578690	-1.374149095
S	0.057077433	1.741889144	4.254672236
C	0.176081828	0.359956683	3.254084743
N	-0.342166118	0.222491220	2.015450789
C	-0.049448608	-1.028059942	1.498613716
C	0.680835254	-1.691536477	2.429802830
N	0.804502693	-0.831015607	3.523722694
H	-0.990073591	0.991044372	1.683919780
H	-0.378304785	-1.332364677	0.517829310
H	1.150315218	-2.663234224	2.436578464
C	1.494129666	-1.133774977	4.760003023
H	1.382101783	-2.196793990	4.978691068
H	1.051586762	-0.523892245	5.549216176
S	3.655670042	-3.431802535	4.014911398
C	3.865621541	-1.798274042	4.358676306
N	2.913671229	-0.853428069	4.684764912
C	3.496720215	0.402820239	4.853021740
C	4.828027898	0.248381726	4.663311567
N	5.038430747	-1.094537636	4.370696601
H	2.893287029	1.272585410	5.063930820
H	5.632756075	0.963237124	4.713999413
H	5.918741200	-1.539998808	4.164287114
C	-2.634971835	2.115347516	2.142464645
H	-3.340666573	2.327457613	1.332647094
C	-3.267618553	1.066657227	3.037706971
H	-3.531036618	0.163085651	2.462144594
H	-2.630393395	0.765245173	3.874567218
H	-4.213690069	1.443797033	3.457585931
C	-2.378342009	4.279823176	4.273877681
H	-3.325367711	4.671662997	3.915128309
C	-1.188654391	5.047401428	4.212503865
H	-1.246206379	6.007969090	3.703907785
H	-2.483623678	3.536808750	5.066661859
C	-0.086557535	4.968830613	5.246919737
H	-0.163517917	4.053202324	5.842414985
H	0.911216175	4.978330718	4.788764964
H	-0.131087653	5.817578791	5.947717305

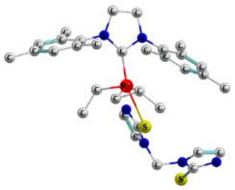

11a‡ (230.64*i* cm^{-1}) *path E*

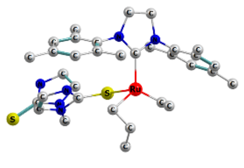

86

RuC33N6H44S2

Ru	-2.223020552	1.950703760	2.196870609
C	-1.632980135	3.492427124	1.068886713
N	-0.498454530	3.719881993	0.317126509
C	-0.637498215	4.854475550	-0.611496132
C	-1.807718838	5.624342524	0.009028349
N	-2.446793366	4.578661263	0.812290992
H	0.285212104	5.439955403	-0.665569691
H	-0.874331009	4.497893758	-1.624655321
H	-1.459754990	6.450591350	0.646740472
H	-2.494918260	6.034934527	-0.738162736
C	0.546852929	2.776762000	0.097615617
C	0.349343791	1.659476056	-0.738011854
C	-0.989320103	1.418108879	-1.386847364
C	1.423421382	0.789566347	-0.945692302
C	2.682334257	1.017123122	-0.376360180
C	3.813200289	0.036910741	-0.587112538
C	2.857643968	2.152019120	0.420214117
C	1.801788801	3.035236496	0.678023113
C	1.976173033	4.202243623	1.619574780
H	-1.237345264	2.220416002	-2.092296969
H	-1.772835866	1.400896248	-0.621896043
H	-0.996168137	0.473328357	-1.937215264
H	1.275500895	-0.085641214	-1.574928025
H	3.749099435	-0.444172828	-1.567764194
H	3.785872030	-0.764046836	0.163375697
H	4.789724327	0.524761874	-0.509772453
H	3.828645041	2.344717211	0.871955387
H	1.837659511	5.162717031	1.110088744
H	1.232104604	4.155455027	2.422558182
H	2.973938535	4.199006897	2.067164910
C	-3.623327797	4.834624882	1.578896942
C	-3.530002030	5.374861097	2.873933058
C	-2.197904175	5.791373172	3.450838500
C	-4.710717887	5.553366173	3.601912845
C	-5.961087281	5.219366468	3.074153184
C	-7.213870760	5.376249120	3.904837817
C	-6.023371868	4.711988148	1.771746397
C	-4.868649968	4.515111891	1.011211905
C	-4.932192244	3.960239189	-0.390080735
H	-2.261639886	5.906665257	4.536786277
H	-1.418959488	5.057779876	3.233437131
H	-1.878488544	6.756120314	3.035701833
H	-4.650722834	5.963521517	4.608195317
H	-7.133253084	6.220416526	4.597431498
H	-8.095811038	5.535269078	3.276397294
H	-7.399354318	4.477936715	4.507292329
H	-6.990103347	4.455969760	1.343327508
H	-4.700020709	4.731866370	-1.135718852
H	-4.199024225	3.159386334	-0.511399220
H	-5.928043310	3.568254466	-0.615841911
S	-0.733801527	2.507212305	4.221309566
C	0.733592782	1.651886296	4.256968099
N	1.433429442	1.194718737	3.187567889
C	2.607692514	0.570606463	3.581976903
C	2.644427622	0.616191705	4.934925536
N	1.477689794	1.264127707	5.349673942
H	1.115758761	1.351842146	2.238673999
H	3.300315683	0.160257925	2.866041452
H	3.378193605	0.285891161	5.653540288
C	1.130960899	1.580842278	6.719253264
H	1.507408079	0.792998951	7.373105270
H	0.043899888	1.656811966	6.780133009
S	3.979754127	1.689636113	8.266813002
C	2.941083113	2.925306613	7.789508731
N	1.717297844	2.832071965	7.158155140
C	1.192615664	4.100588659	6.903356994
C	2.072916431	4.999778207	7.399379765
N	3.127729975	4.271779643	7.939467094
H	0.260738618	4.219825132	6.372910991
H	2.045786324	6.077206257	7.416185106
H	3.953100303	4.640104178	8.386173753
C	-3.857921658	1.214588161	1.578050021
H	-4.693036872	1.568782439	2.215888171
C	-4.353467524	0.363606492	0.434049862
H	-4.871156946	1.015678067	-0.282890801
H	-3.535403811	-0.126991252	-0.104260749
H	-5.080877093	-0.404244928	0.738172312
C	-2.604243703	-0.337009516	2.623994880
H	-0.463319772	-0.123140979	2.485199370
C	-1.385079152	0.017033736	1.920751646
H	-3.311867532	-0.962273057	2.088835959
H	-1.289271197	-0.314177550	0.884592029
C	-2.617708142	-0.624841577	4.113177459
H	-1.836123187	-0.073616559	4.642389810
H	-2.464616193	-1.699717093	4.300503748
H	-3.577775935	-0.351637323	4.566516618

12a *path E*

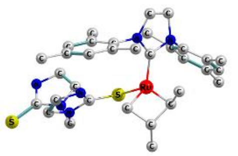

86

RuC33N6H44S2

Ru	-1.829758102	2.008021741	2.117173480
C	-1.590629925	3.135257451	0.611773446
N	-0.709405335	3.244118574	-0.447039347
C	-1.046898357	4.348521416	-1.358747107
C	-1.999854131	5.202216868	-0.503721030
N	-2.467406284	4.217041647	0.475142718
H	-0.146213103	4.891001958	-1.663993731
H	-1.543473617	3.972881905	-2.264164211
H	-1.471733517	6.038358082	-0.021243098
H	-2.833274892	5.614618848	-1.082014018
C	0.440470573	2.430528500	-0.670375862
C	0.438469949	1.446882784	-1.674925341
C	-0.750310421	1.246451546	-2.582523681
C	1.586018845	0.665085032	-1.853212787
C	2.735227352	0.851363937	-1.083353984
C	3.969989710	0.010139300	-1.312704936
C	2.728505770	1.870674443	-0.124366278
C	1.605627304	2.676693029	0.085716804
C	1.647811700	3.797512024	1.095218250
H	-0.609212033	1.792003765	-3.524740508
H	-1.671592288	1.601408280	-2.122799303
H	-0.879574294	0.189615885	-2.835032392
H	1.575583139	-0.112801931	-2.614358392
H	4.598266339	0.438679740	-2.104098830
H	3.710160487	-1.007875073	-1.619547612
H	4.587599080	-0.054575793	-0.410724476
H	3.624848380	2.055301428	0.465005809
H	1.402935065	4.755891070	0.623217806
H	0.919515005	3.639894584	1.896392067
H	2.640942636	3.883379572	1.545008915
C	-3.250630876	4.577304492	1.625377558
C	-2.633052712	5.186241066	2.743906278
C	-1.196744426	5.654594152	2.722695254
C	-3.401523036	5.430883577	3.885909102
C	-4.760758407	5.117841313	3.942198628
C	-5.552771305	5.338026526	5.209846004
C	-5.362163607	4.581686300	2.800936638
C	-4.633394413	4.305169168	1.640443954
C	-5.344351312	3.716881886	0.447757979
H	-0.721370793	5.488381455	3.693131578
H	-0.602484072	5.130746232	1.978952038
H	-1.150628978	6.729951352	2.503900931
H	-2.921271114	5.883785599	4.751304175
H	-5.189995804	6.209577241	5.764732377
H	-6.616656132	5.486047485	4.999729467
H	-5.468415757	4.469978258	5.876003977
H	-6.427886895	4.361227469	2.813712565
H	-6.341943940	4.155633532	0.340788238
H	-4.786166342	3.879193645	-0.475902922
H	-5.466606870	2.636177256	0.568758096
S	-0.512155590	2.438141736	4.269153027
C	0.936302722	1.543304869	4.094974111
N	1.485616245	1.139828685	2.928685932
C	2.671447675	0.457986892	3.140804423
C	2.879480114	0.434937422	4.480367719
N	1.794817894	1.093458434	5.066146093
H	0.972556263	1.253810041	2.055536328
H	3.249003713	0.060769151	2.322740752
H	3.685313667	0.051419749	5.086603469
C	1.623597022	1.334428236	6.486192199
H	2.043428440	0.493730132	7.040275928
H	0.555550181	1.437914038	6.682717736
S	4.634674437	1.251628783	7.684628487
C	3.599239019	2.548308013	7.407374789
N	2.306486069	2.534087441	6.922500547
C	1.805881942	3.832066065	6.805496072
C	2.773297123	4.669585363	7.243438088
N	3.854328670	3.876087511	7.611094679
H	0.822305065	4.012961693	6.400581242
H	2.790584791	5.744050356	7.326336550
H	4.740053528	4.189816651	7.975965413
C	-3.309541555	0.814705796	1.216283716
H	-4.248691714	0.983424545	1.770993377
C	-3.564931486	0.769298411	-0.285218736
H	-3.795013206	1.748430476	-0.714174778
H	-2.678589741	0.386668567	-0.799210508
H	-4.393482409	0.087378521	-0.538117644
C	-2.548574778	-0.449837351	1.680244666
H	-0.414130837	-0.370577486	2.272253899
C	-1.114666300	0.107801957	1.572281962
H	-2.733506580	-1.322476511	1.025735632
H	-0.717292872	0.032175813	0.554805574
C	-2.897041077	-0.866908021	3.117711352
H	-2.749897761	-0.040801076	3.828704947
H	-2.265016565	-1.697655449	3.455185065
H	-3.945728103	-1.177946920	3.195660155

13a‡ (236.14*i* cm^{-1}) *path E*

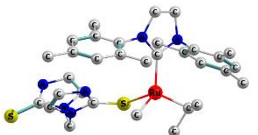

86

RuC33N6H44S2

Ru	-1.876628786	1.760166556	2.115157945
C	-1.497516945	3.245196354	0.848339143
N	-0.549208931	3.416608768	-0.139090229
C	-0.875211166	4.508967446	-1.067752567
C	-1.869152115	5.338596500	-0.244809248
N	-2.327515140	4.348070251	0.732980241
H	0.021897714	5.068146081	-1.350687265
H	-1.335899683	4.116365516	-1.986544141
H	-1.379323325	6.191815889	0.248314016
H	-2.701962229	5.723275396	-0.843072143
C	0.529847431	2.535505312	-0.433276339
C	0.325715770	1.368714673	-1.193298917
C	-1.059932763	1.007173837	-1.657533710
C	1.429435333	0.564133351	-1.486689195
C	2.722394764	0.901754404	-1.068115611
C	3.899208882	0.025697493	-1.432819492
C	2.896341265	2.070481154	-0.321188125
C	1.813746611	2.893307580	0.013889469
C	2.001183417	4.112760306	0.884078859
H	-1.431597774	1.728037910	-2.396387672
H	-1.746797147	1.024272681	-0.809061982
H	-1.079400843	0.015280060	-2.117447902
H	1.277417272	-0.347725844	-2.060680501
H	4.197639758	0.180776750	-2.477216046
H	3.656106580	-1.036525772	-1.322103606
H	4.772395790	0.241136828	-0.808850329
H	3.892414277	2.343577910	0.021854676
H	1.753112852	5.036514642	0.348703905
H	1.341712296	4.064291530	1.756885705
H	3.034481628	4.191488673	1.233284484
C	-3.273534536	4.668081095	1.753700521
C	-2.828565795	5.191728764	2.986510103
C	-1.366788564	5.494221675	3.220051130
C	-3.776224598	5.448140677	3.979658117
C	-5.140793673	5.212619362	3.776341353
C	-6.134652322	5.458051135	4.887795225
C	-5.554489102	4.727453437	2.533834889
C	-4.640426218	4.451831594	1.511484835
C	-5.111709534	3.942890186	0.172102612
H	-0.757245458	4.593403544	3.111849100
H	-0.996375012	6.235582408	2.502568867
H	-1.209238063	5.895908700	4.225231217
H	-3.442368705	5.847161260	4.935569393
H	-5.864974956	6.339981339	5.478565164
H	-7.146307880	5.606414942	4.498151962
H	-6.169430016	4.604812048	5.576875276
H	-6.613022349	4.548697756	2.355894332
H	-5.303263543	4.775241684	-0.518263594
H	-4.359183633	3.297183286	-0.281855771
H	-6.043250617	3.377973424	0.269972096
S	-0.432132827	2.056414967	4.291400920
C	1.111742631	1.437786653	3.945175801
N	1.629249225	1.190140816	2.715500931
C	2.924296163	0.702459939	2.803999710
C	3.235242661	0.639254099	4.120697421
N	2.108214669	1.076266096	4.822731739
H	1.066588701	1.307610888	1.876595759
H	3.494463113	0.456815975	1.924300073
H	4.137340913	0.361100120	4.642923868
C	2.015551057	1.219759124	6.260736893
H	2.610098074	0.437944573	6.735334437
H	0.963595815	1.136288716	6.538929798
S	5.081535500	1.583469314	7.255029126
C	3.831736499	2.702447901	7.117132152
N	2.526687257	2.496851548	6.717609658
C	1.816823181	3.698966662	6.703409343
C	2.666323962	4.664617726	7.122249002
N	3.884737941	4.044147713	7.375703786
H	0.791192970	3.733818222	6.370253512
H	2.516743959	5.722951018	7.260968654
H	4.732852576	4.482499337	7.699051282
C	-3.738095334	0.929705068	1.515367645
H	-4.448811731	1.683036866	1.879690959
C	-4.124845446	0.359029246	0.161758890
H	-4.171085862	1.131213357	-0.612919805
H	-3.400145261	-0.393328255	-0.170655403
H	-5.107824802	-0.136606776	0.191261215
C	-3.303095894	0.001272809	2.561916671
H	-0.519901621	-0.452123676	2.602103044
C	-1.229776423	0.006342752	1.897141105
H	-3.215229391	-1.041274516	2.254059603
H	-1.446765181	-0.656173119	1.050598746
C	-3.768224467	0.120200785	4.002225543
H	-4.016427296	1.156972219	4.255370072
H	-2.995343841	-0.209684803	4.704820382
H	-4.663641379	-0.497429181	4.179467081

14a *path E*

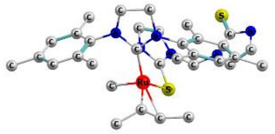

86

RuC33N6H44S2

Ru	-1.119468072	1.241979746	1.997174272
C	-0.853424447	2.812234513	0.777331288
N	-0.044566061	3.052653064	-0.319968833
C	-0.231493559	4.389938499	-0.905218100
C	-0.926258451	5.134217901	0.234234984
N	-1.490214972	4.022356439	1.008009983
H	0.729891726	4.830523090	-1.188405995
H	-0.862106614	4.341383687	-1.803779767
H	-0.213359982	5.709506894	0.844060580
H	-1.710382986	5.816388509	-0.108409160
C	0.519926304	2.031252083	-1.141360809
C	-0.305528684	1.326863323	-2.042808332
C	-1.775790522	1.652991563	-2.161585543
C	0.266831442	0.322069311	-2.822410639
C	1.632025680	0.013547176	-2.741735120
C	2.219892622	-1.073096229	-3.611502329
C	2.425671002	0.743129260	-1.857870708
C	1.889483321	1.753586838	-1.049795684
C	2.787194815	2.500748513	-0.092692587
H	-1.932712215	2.606448941	-2.681477531
H	-2.237263286	1.730946142	-1.174179010
H	-2.302921747	0.881957988	-2.730068560
H	-0.365072794	-0.239071016	-3.507990120
H	2.326076194	-0.733986316	-4.649763080
H	1.577102463	-1.959955479	-3.626696489
H	3.209699525	-1.379219147	-3.260328166
H	3.486734255	0.513875032	-1.779516040
H	3.523112067	3.106882350	-0.634152234
H	2.208766117	3.165052510	0.552658527
H	3.348952767	1.798526620	0.534035536
C	-2.280012489	4.308315572	2.163325832
C	-1.678659089	4.593126186	3.401805935
C	-0.178984298	4.650429289	3.565056086
C	-2.509661471	4.898722874	4.485510679
C	-3.898612936	4.949362157	4.361163785
C	-4.777262733	5.232402200	5.556929367
C	-4.466309480	4.701692908	3.106259257
C	-3.677693868	4.377671754	2.001543106
C	-4.300264123	4.118414183	0.651886978
H	0.327551533	3.995842659	2.856639776
H	0.182951196	5.675350602	3.410386287
H	0.114467066	4.348235591	4.573660896
H	-2.049358642	5.107402497	5.447493571
H	-4.244274802	5.805444296	6.322614609
H	-5.671878681	5.796928623	5.274371116
H	-5.117096038	4.297558803	6.020311410
H	-5.547030486	4.748847383	2.989970542
H	-4.077044421	4.931162409	-0.050442254
H	-3.905832545	3.201424630	0.208015428
H	-5.387612524	4.031947193	0.729024065
S	-0.254484396	0.963560948	4.456490711
C	1.295289139	1.661870868	4.379132299
N	1.988915814	1.955046218	3.253913580
C	3.188122156	2.579939340	3.558260665
C	3.261781578	2.667160757	4.909269099
N	2.096171462	2.082081622	5.412938900
H	1.555233578	1.753879708	2.341677516
H	3.872469560	2.906102243	2.792391101
H	3.991542107	3.105358292	5.571780500
C	1.730691295	1.976851280	6.811061808
H	2.641299151	1.894891885	7.406166346
H	1.105066981	1.090876197	6.929751996
S	3.231243678	4.509605023	8.154974029
C	1.596910000	4.231430772	7.870612094
N	0.995973503	3.131390502	7.290948936
C	-0.388481859	3.301785402	7.221071715
C	-0.666690159	4.503419270	7.777817270
N	0.547641228	5.056064666	8.169298238
H	-1.017744286	2.565775732	6.745766058
H	-1.607971690	5.006756715	7.927224386
H	0.692189307	5.949486568	8.613185783
C	-2.997009432	0.420439563	1.228268184
H	-3.655362158	1.206005484	0.852216116
C	-2.866232321	-0.770871743	0.296374568
H	-2.388345887	-0.513467239	-0.652445608
H	-2.260580678	-1.556326434	0.759667498
H	-3.855054435	-1.197803111	0.066717086
C	-2.962806999	0.241229482	2.643719616
H	0.740519888	-0.497178945	2.012825688
C	0.167880455	0.120895597	1.302750579
H	-2.694408327	-0.755675580	2.998605225
H	0.290611145	-0.172405904	0.255459926
C	-3.817228996	1.037518218	3.608089952
H	-3.979942019	2.059935632	3.256850962
H	-3.352698377	1.102351474	4.598798068
H	-4.808882172	0.576619252	3.739718429

11a‡ (274.48*i* cm⁻¹) *path E'*

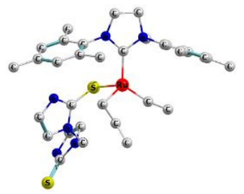

86
RuC33N6H44S2

Ru	-1.512345204	2.383029778	2.281792623
C	-1.535906371	3.632259147	0.760687928
N	-0.668948817	3.747994359	-0.316411131
C	-1.211082736	4.570194483	-1.406867873
C	-2.275015586	5.392305799	-0.671032512
N	-2.533746381	4.554173892	0.502994171
H	-0.431193447	5.186479533	-1.866104113
H	-1.658384350	3.942659234	-2.191963017
H	-1.893518168	6.381593427	-0.375722219
H	-3.183837380	5.543279999	-1.263291224
C	0.397595154	2.837912161	-0.570448078
C	0.147214469	1.574183496	-1.136112846
C	-1.254700860	1.165150641	-1.515182861
C	1.227019225	0.712200417	-1.350076648
C	2.540449392	1.085204817	-1.048491601
C	3.688821189	0.136393527	-1.306057829
C	2.764808427	2.364215248	-0.522105160
C	1.707592256	3.252189814	-0.275987137
C	1.957471248	4.613243621	0.323200706
H	-1.556596969	1.639356522	-2.457450190
H	-1.964357779	1.461767635	-0.739002485
H	-1.321828990	0.082889221	-1.656152580
H	1.036669633	-0.277004635	-1.761568092
H	3.944051825	0.103204075	-2.372537888
H	3.436120529	-0.886650062	-1.006153534
H	4.590795345	0.438328376	-0.764277093
H	3.782827168	2.680776717	-0.301330953
H	1.549800466	5.408064236	-0.310720726
H	1.467914069	4.688538951	1.298665637
H	3.027998579	4.795718188	0.454925845
C	-3.531135485	4.922692365	1.453481567
C	-3.221281558	5.781779334	2.521316226
C	-1.816463468	6.303786042	2.706719291
C	-4.241597460	6.124925056	3.414908942
C	-5.545150214	5.642865326	3.265214296
C	-6.621205339	5.984718809	4.269678811
C	-5.823008412	4.794517613	2.188391163
C	-4.830137629	4.414744942	1.283069695
C	-5.108163549	3.427511560	0.177372950
H	-1.741025386	6.915843803	3.609684967
H	-1.102532352	5.477136625	2.784410441
H	-1.499004963	6.918585759	1.856331609
H	-4.010510111	6.784240812	4.249381167
H	-6.407577550	6.925653860	4.786096090
H	-7.602786681	6.073708757	3.792451434
H	-6.702989848	5.202178573	5.035064941
H	-6.830300132	4.400901494	2.065358025
H	-6.145337324	3.081568482	0.206854921
H	-4.920853435	3.857175121	-0.813493983
H	-4.442538384	2.564467503	0.286754265
S	0.645692780	3.090946163	3.533232086
C	1.611210356	1.688679776	3.529018340
N	2.327735298	1.192386248	2.485036347
C	2.951713462	0.001900627	2.825458808
C	2.626063372	-0.263246118	4.113293519
N	1.787724803	0.769157070	4.536997646
H	2.330271273	1.628863197	1.567187718
H	3.567620574	-0.540145367	2.126562176
H	2.916500541	-1.051928157	4.789392548
C	1.170924099	0.868850944	5.844688425
H	0.993664298	-0.137227902	6.226909763
H	0.234027412	1.416509782	5.730346069
S	3.244121124	-0.708143910	7.775726580
C	2.921706819	0.936578420	7.621567529
N	2.009006017	1.564175929	6.798983917
C	2.074706524	2.950040282	6.949776158
C	3.013991138	3.201041040	7.890182917
N	3.516799490	1.968536404	8.292978519
H	1.459247586	3.601841894	6.349477855
H	3.367069130	4.132138281	8.302547371
H	4.235523159	1.801918592	8.979737038
C	-3.191168666	1.599753478	2.657173610
H	-3.706964556	0.805486048	2.101961192
C	-4.014349524	2.095357692	3.824803465
H	-4.981198800	2.469508342	3.462809862
H	-4.227156666	1.287946864	4.542341926
H	-3.531249802	2.916833421	4.367522425
C	-1.548264419	0.153450681	3.051209072
H	0.126444945	0.364486367	1.645096184
C	-0.957209840	0.407459260	1.748335411
H	-0.890879453	0.320877503	3.905959416
H	-1.464715513	-0.069225400	0.906875067
C	-2.486450861	-1.011056441	3.313988532
H	-1.903134144	-1.915846432	3.542714136
H	-3.106563885	-1.242527430	2.442247804
H	-3.153896333	-0.832059189	4.166094745

12a *path E'*

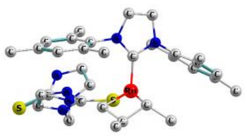

86
RuC33N6H44S2

Ru	-1.858376033	1.905561114	2.252879586
C	-1.549258032	3.126259192	0.828038945
N	-0.651348138	3.232011402	-0.216554798
C	-0.892497769	4.404026351	-1.070227062
C	-1.761923383	5.284857050	-0.163106568
N	-2.321032930	4.282239287	0.743321061
H	0.050257680	4.880791203	-1.356656864
H	-1.426135891	4.116980664	-1.987833804
H	-1.159232026	6.029120597	0.380865002
H	-2.550467838	5.813692128	-0.708220234
C	0.423417065	2.346778519	-0.502987967
C	0.259379627	1.316139257	-1.444727211
C	-1.075921947	1.107896458	-2.113734901
C	1.346261156	0.481544473	-1.716286484
C	2.584429750	0.656517267	-1.088112192
C	3.745499361	-0.254702493	-1.414938860
C	2.723353597	1.702385540	-0.169879866
C	1.658557353	2.560212306	0.131765811
C	1.814024452	3.678787796	1.134526407
H	-1.267918315	1.879563354	-2.869778758
H	-1.884646309	1.160419679	-1.380502594
H	-1.120473237	0.135878358	-2.612209154
H	1.220735262	-0.333680178	-2.425986469
H	4.195263542	0.008367502	-2.380599639
H	3.425218266	-1.299865186	-1.482826149
H	4.534666372	-0.190457605	-0.659143275
H	3.682052929	1.859949489	0.320930789
H	1.623471050	4.654896857	0.673403322
H	1.096906967	3.566734458	1.954506284
H	2.822517081	3.692790215	1.557341103
C	-3.231484214	4.618597016	1.791888293
C	-2.770042552	5.152354083	3.009900231
C	-1.319149639	5.515498965	3.223780033
C	-3.709915869	5.421190917	4.013671319
C	-5.074255403	5.197607879	3.824888405
C	-6.066360738	5.451861479	4.935522681
C	-5.507830851	4.714422168	2.583592631
C	-4.607767422	4.423624409	1.559518415
C	-5.079596864	3.920823897	0.217752027
H	-1.035994777	5.386965046	4.272004664
H	-0.650507833	4.896124349	2.627402356
H	-1.147491470	6.567072584	2.958064966
H	-3.361930264	5.824633834	4.962785067
H	-5.684817129	6.178751347	5.659137943
H	-7.017885130	5.827415151	4.544680511
H	-6.283607268	4.526128693	5.482474637
H	-6.570894584	4.555520140	2.415126427
H	-6.156694715	3.732584392	0.224120905
H	-4.868042854	4.650788025	-0.573173861
H	-4.564023539	2.996385030	-0.051805777
S	-0.487342535	2.236758055	4.368444419
C	0.995740200	1.422699171	4.120371194
N	1.484358140	0.998869246	2.934922100
C	2.732779757	0.419558310	3.086130379
C	3.039784782	0.480670326	4.405442762
N	1.954143034	1.086991981	5.043106069
H	0.919636003	1.079870181	2.090487381
H	3.278824463	0.027026981	2.244791637
H	3.915396340	0.188606688	4.963870014
C	1.876542224	1.408338713	6.456003720
H	2.416408287	0.647058644	7.020730384
H	0.823285971	1.431232674	6.738961748
S	4.970011653	1.667562453	7.384083986
C	3.798961385	2.848462723	7.129512528
N	2.478027193	2.690004123	6.760051363
C	1.849836010	3.929365261	6.620923674
C	2.767194218	4.874245151	6.928140771
N	3.944699570	4.203529340	7.240240829
H	0.824400028	3.998263233	6.293559648
H	2.689877391	5.949037561	6.952465747
H	4.823721044	4.616148485	7.510819096
C	-3.351728362	0.826972725	1.227248279
H	-3.490659833	1.065128812	0.163972253
C	-4.701393416	0.824264987	1.949052967
H	-5.230073689	1.777985335	1.868833972
H	-5.371213476	0.039526460	1.557622665
H	-4.575976744	0.619030953	3.022310912
C	-2.560072807	-0.493259065	1.376511381
H	-0.551585283	-0.500320010	2.312905096
C	-1.134924182	0.055605687	1.562863529
H	-2.875883214	-0.974077178	2.316696666
H	-0.578288246	0.063255861	0.620528360
C	-2.748121148	-1.523407891	0.254714240
H	-2.157629569	-2.430632224	0.437290242
H	-2.427980240	-1.106720714	-0.707331877
H	-3.801030547	-1.818460603	0.157766502

13a‡ (270.93*i* cm^{-1}) *path E'*

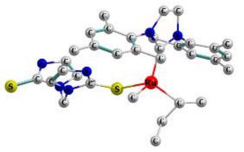

86
RuC33N6H44S2
Ru -1.214909052 2.004476002 2.156881788
C -1.230054486 3.394723908 0.720924924
N -0.618124588 3.329436581 -0.521184954
C -1.091404598 4.350899595 -1.463252701
C -1.677350065 5.395307181 -0.514119950
N -1.944518908 4.585871397 0.681341376
H -0.270489230 4.736537369 -2.076221498
H -1.856842481 3.938988799 -2.138317025
H -0.957203796 6.197175981 -0.290727830
H -2.592321013 5.857299059 -0.897068378
C 0.171596007 2.251193714 -1.009695108
C -0.428445405 1.079192500 -1.504985381
C -1.928663700 0.931397334 -1.498709039
C 0.400438191 0.067910278 -1.997507463
C 1.793277760 0.202871440 -2.032849458
C 2.666194685 -0.923422615 -2.536501988
C 2.358412985 1.393884049 -1.566908270
C 1.566341817 2.425023886 -1.046291260
C 2.187072024 3.696023664 -0.513919958
H -2.394912990 1.613651060 -2.220542376
H -2.324945913 1.173406547 -0.508628144
H -2.225225353 -0.087044896 -1.764957764
H -0.053146628 -0.849033759 -2.368174556
H 2.169349449 -1.493139275 -3.328152883
H 2.900656769 -1.629907647 -1.729601427
H 3.616390106 -0.550426866 -2.931745774
H 3.437923859 1.528446963 -1.612575581
H 1.969241008 4.551759919 -1.164235908
H 1.788790918 3.938161953 0.476223173
H 3.274045269 3.601743885 -0.438697118
C -2.565272997 5.185228285 1.815765468
C -1.780393877 5.682201827 2.877174395
C -0.272719294 5.698146177 2.788516706
C -2.431841405 6.219198266 3.990325673
C -3.826330992 6.312755827 4.057455262
C -4.502498697 6.852904175 5.296219066
C -4.572300770 5.887448859 2.956052975
C -3.964096977 5.328118840 1.828387567
C -4.798828551 4.906920582 0.641944148
H 0.156827324 6.257688834 3.624600594
H 0.146688702 4.689167556 2.795760994
H 0.057060251 6.176157416 1.858773909
H -1.833891299 6.593836444 4.819028255
H -3.916618893 7.656344401 5.755347193
H -5.499690641 7.244623448 5.073053439
H -4.620938200 6.066033040 6.052006873
H -5.656440753 5.978789762 2.978148808
H -5.816400755 4.646069803 0.947400575
H -4.878649541 5.720980161 -0.091137396
H -4.354900005 4.048224410 0.131941922
S 0.800168340 2.293143762 3.847598027
C 2.202002311 1.639501768 3.149720360
N 2.401660484 1.343792423 1.840418805
C 3.671655737 0.828216756 1.629316580
C 4.291209434 0.794196416 2.833089534
N 3.375602302 1.277550793 3.771653123
H 1.663062236 1.451431493 1.151182784
H 4.006160716 0.537772169 0.647722192
H 5.288244278 0.509457274 3.130863477
C 3.630726269 1.445114431 5.187672702
H 4.311248743 0.659975400 5.520046148
H 2.674732294 1.382400815 5.710075157
S 6.849470003 1.773552799 5.403648359
C 5.616902616 2.909153627 5.560270786
N 4.251659492 2.719084552 5.488889237
C 3.573553274 3.930414453 5.637160199
C 4.510376334 4.886587366 5.830573758
N 5.746574323 4.251219908 5.787259435
H 2.498361327 3.977428246 5.562119782
H 4.411142475 5.947471569 5.992813172
H 6.652725802 4.679803911 5.893184873
C -3.163408330 1.103590077 2.173801176
H -3.520047586 0.586959592 1.275723987
C -4.201470363 2.048507546 2.762903743
H -4.611377815 2.714158350 2.003355133
H -5.043319580 1.496791847 3.210359885
H -3.780627711 2.682311163 3.555638218
C -2.436691883 0.249752933 3.104836208
H 0.277218792 -0.163484460 2.140118266
C -0.642693831 0.281438657 1.733510940
H -2.415283520 0.606946774 4.137319574
H -1.154006842 -0.400037649 1.045260092
C -2.509491836 -1.268116020 3.068123510
H -1.643779846 -1.739957186 3.547002841
H -2.586574462 -1.658090610 2.048251112
H -3.406325021 -1.606130383 3.609842410

14a *path E'*

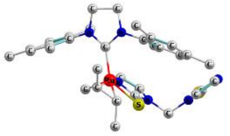

86
RuC33N6H44S2
Ru -0.979931292 1.567700103 2.018865081
C -1.000871690 2.892440782 0.519041863
N -0.600532571 2.873087306 -0.796371131
C -0.966933558 4.087911525 -1.540558455
C -1.209341540 5.082483538 -0.407932701
N -1.497928274 4.179426494 0.714720448
H -0.166149260 4.392535464 -2.220031647
H -1.873805274 3.914093529 -2.138192815
H -0.315406279 5.689035233 -0.197735003
H -2.047207705 5.759820858 -0.598018336
C 0.006914802 1.791083102 -1.498272601
C -0.775872664 0.760130982 -2.041568466
C -2.261357206 0.722358291 -1.796505290
C -0.133863083 -0.259077536 -2.749435091
C 1.253625613 -0.275976016 -2.924422957
C 1.923531484 -1.419421333 -3.650778996
C 2.004248815 0.772331453 -2.384886972
C 1.400484900 1.814667439 -1.675694403
C 2.226598680 2.949710649 -1.117950981
H -2.744504262 1.666911942 -2.068595442
H -2.449711551 0.563756199 -0.730881322
H -2.737314547 -0.081653935 -2.364301895
H -0.732152843 -1.067179706 -3.165532298
H 1.308528223 -1.782316158 -4.480844196
H 2.089215550 -2.268416910 -2.975432045
H 2.898064017 -1.126137661 -4.053149393
H 3.084230841 0.779397929 -2.519686645
H 2.077670255 3.875412170 -1.688375672
H 1.953784445 3.174134157 -0.083223541
H 3.294751887 2.715428611 -1.158803797
C -1.737540596 4.737275919 2.010254509
C -0.669291960 4.950009627 2.911606414
C 0.773041789 4.768617638 2.497918406
C -0.962869793 5.436294257 4.186887339
C -2.265994710 5.758875744 4.577101866
C -2.555268292 6.225397213 5.984533389
C -3.285373826 5.640181896 3.630931758
C -3.041360208 5.151185208 2.342971975
C -4.149771267 5.172914431 1.316290835
H 1.430627447 4.769725293 3.370687746
H 0.922819732 3.831345941 1.962856443
H 1.085985724 5.591650023 1.842110884
H -0.143086451 5.583607825 4.885444955
H -1.725917706 6.815303758 6.390202035
H -3.460095514 6.839338865 6.029026480
H -2.705676186 5.371024190 6.656550345
H -4.297808475 5.939377064 3.895220910
H -5.116852891 4.927216455 1.765113574
H -4.238734407 6.179772970 0.887734577
H -3.959599949 4.475247366 0.500485214
S 0.591353053 1.522408604 4.142976775
C 2.153478477 1.705073335 3.491706829
N 2.500040323 1.635816557 2.184125680
C 3.855107238 1.874065368 2.016266225
C 4.378561413 2.093731512 3.247179400
N 3.325369948 1.971781015 4.157792888
H 1.771040164 1.422754677 1.485953878
H 4.318791378 1.871215742 1.043724025
H 5.371212213 2.352174621 3.581266161
C 3.417873321 2.157576761 5.592273840
H 4.411089844 1.851858753 5.923978912
H 2.643905654 1.548244227 6.061326740
S 5.917229615 4.186685653 5.949833093
C 4.264400966 4.441573366 6.131160368
N 3.229406800 3.539357656 5.986800714
C 1.996537064 4.160502873 6.195760492
C 2.252365447 5.454775620 6.496580891
N 3.633526483 5.610388145 6.458537630
H 1.071690401 3.618728048 6.073564925
H 1.584184207 6.266545141 6.733689867
H 4.154093706 6.456212191 6.631656529
C -3.038732418 0.864512593 1.880618495
H -3.009212688 -0.147480454 1.468354882
C -4.123143028 1.735941254 1.284570374
H -3.948918495 1.977187486 0.230786664
H -5.103568519 1.239510401 1.351412288
H -4.196601017 2.673774675 1.836923147
C -2.679791097 0.990640551 3.259919940
H 0.568452442 -0.440972468 1.840552104
C -0.089479273 0.169821900 1.200374455
H -3.068055246 1.876687701 3.775729981
H -0.318828724 -0.302932939 0.239468355
C -2.478274610 -0.210325434 4.165788713
H -1.794720889 0.003983981 4.994242770
H -2.062704662 -1.056755114 3.610128048
H -3.435333482 -0.534839641 4.604592308

11a‡ (179.01*i* cm^{-1}) *path Z*

86
RuC33N6H44S2

Ru	-1.603413049	3.576322835	3.468202191
C	-1.350684055	4.098772410	1.577297926
N	-0.199439284	4.553115829	0.948478216
C	-0.372987569	4.876993817	-0.470300602
C	-1.895203832	4.978289908	-0.584591041
N	-2.335283274	4.195519352	0.579229802
H	0.137701016	5.811602802	-0.725825565
H	0.035989828	4.080866183	-1.111138303
H	-2.234291426	6.022112138	-0.517920570
H	-2.288350827	4.552484233	-1.513495304
C	1.073074715	4.727304441	1.567478170
C	2.092089260	3.783834725	1.377069697
C	1.895434357	2.566898452	0.506785478
C	3.333881555	4.009931015	1.983522551
C	3.577283191	5.140666221	2.761923693
C	4.906049575	5.343180156	3.452383805
C	2.554054250	6.088060849	2.899326917
C	1.308785751	5.909029244	2.298369767
C	0.241930223	6.973321638	2.375760946
H	2.316940871	2.731329900	-0.493421133
H	0.839641292	2.326574900	0.387797373
H	2.399681663	1.694273201	0.931695199
H	4.123411128	3.274873402	1.843595009
H	5.232243064	6.387532090	3.397931348
H	5.690669845	4.722247620	3.007993667
H	4.840214984	5.082213648	4.516017504
H	2.738276718	6.992083314	3.476691053
H	0.037299666	7.394087058	1.382973778
H	-0.693944232	6.550702419	2.747133631
H	0.547605641	7.793316933	3.031939834
C	-3.738634172	4.063032722	0.813285312
C	-4.482100703	5.149591033	1.324764618
C	-3.815364842	6.456060779	1.683775333
C	-5.857619912	4.997625520	1.499418631
C	-6.518389813	3.804311930	1.183304985
C	-8.002083483	3.660054036	1.429198573
C	-5.763734521	2.751230355	0.668279630
C	-4.380483500	2.861288864	0.475481976
C	-3.618644949	1.682867144	-0.084870293
H	-4.416349641	7.008528575	2.412073668
H	-2.830074458	6.278777447	2.118486017
H	-3.695631540	7.101605503	0.804767137
H	-6.430234999	5.830303980	1.903812241
H	-8.539092847	4.585058969	1.194006860
H	-8.433998495	2.854227810	0.828081706
H	-8.203071512	3.428542853	2.482940176
H	-6.259058387	1.818360859	0.404964611
H	-2.648037776	1.990306918	-0.482359045
H	-3.447119968	0.927524057	0.691898864
H	-4.184425624	1.195628849	-0.886001043
S	0.341076210	1.807826489	3.858408145
C	0.061418103	0.674179594	2.630490416
N	-0.823886522	0.811781524	1.613985486
C	-0.747241433	-0.261764111	0.738074413
C	0.187334562	-1.110407402	1.230902752
N	0.671385767	-0.536880935	2.411149471
H	-1.357142624	1.686785554	1.553378867
H	-1.364526600	-0.327479333	-0.142474002
H	0.585550407	-2.044881458	0.867898476
C	1.727887008	-1.071969226	3.245571253
H	1.688816602	-2.161495278	3.208777916
H	1.570620978	-0.708802414	4.262142920
S	3.446748052	-2.860928192	1.172050951
C	3.804735560	-1.388331354	1.903224272
N	3.045877861	-0.664509551	2.801054449
C	3.687033496	0.527207250	3.146420384
C	4.868006814	0.544645305	2.486348196
N	4.927955722	-0.626108481	1.738122177
H	3.212168834	1.252435004	3.788573207
H	5.658198573	1.277445839	2.480476832
H	5.676258110	-0.919391324	1.129673525
C	-3.250578453	4.252636221	4.117070777
H	-3.566986903	5.290770186	4.297062321
C	-4.360846813	3.268910503	4.418835800
H	-5.207101542	3.459951473	3.746391167
H	-4.051190220	2.225824561	4.276965419
H	-4.730189278	3.372486308	5.450572389
C	-1.743418120	4.319266834	5.706155050
H	-2.441488653	5.077108451	6.054851206
C	-0.628077648	4.800282964	4.923147349
H	-0.596558855	5.869046752	4.717150793
H	0.357825189	4.380710207	5.127805352
C	-1.645337703	3.145204898	6.659524610
H	-2.580244426	2.571563573	6.685938788
H	-0.844038457	2.456635469	6.378845311
H	-1.453045571	3.494108898	7.685243774

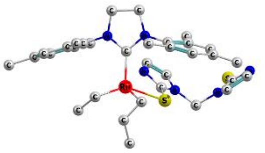

12a *path Z*

86
RuC33N6H44S2

Ru	-1.600406796	3.109350253	3.350514570
C	-1.212709428	3.858081369	1.669005473
N	-0.158169881	4.526591499	1.083912627
C	-0.263987361	4.631681131	-0.375736893
C	-1.760372243	4.409016845	-0.612955407
N	-2.150703312	3.640961212	0.587765898
H	0.085031094	5.608471323	-0.724579742
H	0.351921889	3.862307810	-0.866692935
H	-2.300282334	5.360915711	-0.674589682
H	-1.972174037	3.841459690	-1.525401654
C	1.039515779	4.991516407	1.718492228
C	2.129888984	4.121874695	1.906903465
C	2.021876955	2.666935273	1.541806043
C	3.288829879	4.618632504	2.505673649
C	3.395760610	5.952000462	2.914754536
C	4.639638730	6.452349259	3.612131949
C	2.310820109	6.798881330	2.686432497
C	1.128764060	6.343661125	2.090288998
C	-0.016319906	7.308844853	1.896251068
H	1.759820005	2.525799671	0.490040973
H	1.236541307	2.202563712	2.144886280
H	2.961138825	2.139293186	1.723502076
H	4.126694408	3.942670474	2.665730238
H	4.768665544	7.531283154	3.480743254
H	5.539804540	5.954179748	3.237221917
H	4.586034457	6.259915581	4.691465157
H	2.378902263	7.843550240	2.983925627
H	-0.609968729	7.081210587	1.008457042
H	-0.699485994	7.273343405	2.752167259
H	0.352860022	8.335178048	1.810753019
C	-3.560198801	3.628946546	0.943835108
C	-4.162334688	4.774304342	1.523911028
C	-3.421365181	6.078174776	1.705160209
C	-5.501415570	4.705079389	1.911925584
C	-6.273001603	3.555783608	1.721250269
C	-7.701771180	3.495691122	2.206326623
C	-5.679439543	2.471893185	1.077952216
C	-4.337827368	2.487170731	0.676683649
C	-3.789671921	1.272325525	-0.030674469
H	-4.012766543	6.776800163	2.302287718
H	-2.470258379	5.923227681	2.210487101
H	-3.221010862	6.566500940	0.744117223
H	-5.954827482	5.578334590	2.375983615
H	-8.214550290	4.452915322	2.064436924
H	-8.272751257	2.721927943	1.684312771
H	-7.737912625	3.264903091	3.278405563
H	-6.271500386	1.580889335	0.880086052
H	-3.120918990	1.540583306	-0.853991752
H	-3.238273522	0.632757407	0.665730744
H	-4.604947730	0.677183465	-0.452084233
S	-1.450012458	0.520733846	2.885098759
C	-0.530247553	0.347386674	1.463954973
N	-0.658547703	1.065016574	0.321557926
C	0.246087482	0.638084227	-0.637077065
C	0.961318642	-0.374251146	-0.088746706
N	0.471648279	-0.558088020	1.205101069
H	-1.280094400	1.882207740	0.278256342
H	0.303697154	1.091071983	-1.613608627
H	1.791003499	-0.957230118	-0.455009511
C	1.080073478	-1.408553622	2.215771990
H	1.527800429	-2.271989063	1.722615719
H	0.303908816	-1.715562736	2.916607136
S	4.169196128	-1.453968514	1.236694216
C	3.439831620	-0.665503833	2.532950526
N	2.122450901	-0.714306687	2.942195321
C	1.917978561	0.107834866	4.053143699
C	3.111480383	0.660023704	4.363348679
N	4.028628216	0.178394954	3.433615033
H	0.934786832	0.248293997	4.470325999
H	3.378770325	1.362082978	5.135952497
H	5.013576390	0.389179637	3.388675073
C	-1.707604387	4.847458123	4.543770695
H	-1.263530302	5.731774674	4.074813105
C	-3.080035400	5.202286351	5.118007636
H	-3.744070340	5.611696279	4.351320680
H	-3.593071184	4.330792023	5.543314218
H	-3.008594096	5.950995835	5.925733606
C	-0.662852250	4.248609603	5.527214992
H	-0.038240078	5.021218994	6.012244886
C	0.104467681	3.379677290	4.516257315
H	0.933829828	3.913725265	4.056456482
H	0.474608374	2.426241928	4.922601126
C	-1.276803671	3.393655868	6.648609276
H	-1.897843074	3.985168516	7.329991392
H	-1.912899460	2.588194081	6.249674346
H	-0.487758559	2.912603802	7.239096948

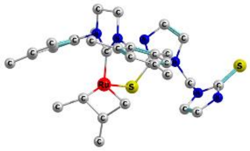

13a‡ (263.82*i* cm⁻¹) *path Z*

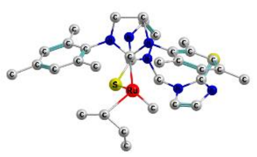

86
RuC33N6H44S2

Ru	-1.102454361	3.241515106	3.344296228
C	-1.150049175	3.811269706	1.458808255
N	-0.104690710	4.431977345	0.805124830
C	-0.326222424	4.643980864	-0.627278845
C	-1.839162738	4.435315532	-0.749824477
N	-2.166605795	3.668090947	0.477999328
H	-0.007525049	5.645834437	-0.935083329
H	0.242724475	3.916261921	-1.225748110
H	-2.378780226	5.390664551	-0.762958775
H	-2.127492185	3.877848680	-1.646741233
C	1.155596523	4.768409146	1.400204981
C	2.206725833	3.839063487	1.426972710
C	2.020864216	2.447912537	0.886235673
C	3.419900134	4.214146119	2.015056087
C	3.607043667	5.479128012	2.575620640
C	4.906911875	5.853300936	3.249146517
C	2.545612081	6.388002124	2.524975924
C	1.319802625	6.055426220	1.945963757
C	0.175122050	7.034246700	1.969444925
H	1.612279101	2.453122069	-0.127621868
H	1.310516530	1.901830419	1.512590742
H	2.957992615	1.886742685	0.867807866
H	4.237768511	3.496058675	2.028375846
H	5.164182498	6.902203833	3.068752128
H	5.737986483	5.235390100	2.895456620
H	4.837592071	5.723097216	4.336907866
H	2.671880899	7.379017543	2.956552080
H	-0.292711270	7.148337684	0.986319796
H	-0.597192047	6.669526548	2.652119011
H	0.504662915	8.020316865	2.308642916
C	-3.567442187	3.691656819	0.852032371
C	-4.108155144	4.813920277	1.522310854
C	-3.265395700	6.002719814	1.910251191
C	-5.464664312	4.811231626	1.853997008
C	-6.309203818	3.747710621	1.526833070
C	-7.755184482	3.739962425	1.962671117
C	-5.765609350	2.684373859	0.808988662
C	-4.411005861	2.639022071	0.457749431
C	-3.916924547	1.441420695	-0.315892228
H	-3.888392026	6.799550144	2.325419279
H	-2.521716700	5.700124128	2.651879324
H	-2.714613533	6.418287939	1.060814317
H	-5.871371110	5.665738596	2.390867653
H	-8.177652308	4.750005452	1.976702450
H	-8.372078708	3.121156966	1.303405084
H	-7.851150870	3.333169666	2.977481506
H	-6.408255043	1.857022914	0.515050567
H	-3.176810132	1.712393164	-1.075646938
H	-3.464924696	0.708084849	0.359815858
H	-4.746024061	0.948720881	-0.831895727
S	-1.810187641	0.737105210	2.897862599
C	-0.815517271	0.297846215	1.593750642
N	-0.861140089	0.792325869	0.328997578
C	0.084135661	0.179828440	-0.479209404
C	0.744961387	-0.717689687	0.290155754
N	0.186250236	-0.646379780	1.569305655
H	-1.412859620	1.624503826	0.124761278
H	0.204792395	0.436570991	-1.519029381
H	1.579116902	-1.367424581	0.082900598
C	0.788070128	-1.197343733	2.775878996
H	1.321502303	-2.111495909	2.514373204
H	-0.005232765	-1.394936882	3.496227633
S	3.908161892	-1.128800833	1.882613252
C	3.050139322	-0.158069835	2.957600522
N	1.734879358	-0.268693853	3.365115283
C	1.405392394	0.768154223	4.247010143
C	2.517417258	1.519190463	4.409567521
N	3.509987631	0.939743597	3.628092406
H	0.409419045	0.883719338	4.639295565
H	2.678391326	2.415330322	4.985084764
H	4.457325092	1.265220477	3.518056327
C	-2.798048699	3.656941952	4.571379638
H	-3.113428013	4.699412354	4.474806373
C	-3.979081228	2.707626865	4.443662667
H	-4.394641667	2.726184871	3.434488644
H	-3.715293276	1.671908547	4.672848520
H	-4.778702538	2.999571444	5.141929689
C	-1.780429135	3.450667670	5.591185367
H	-1.553468791	4.311854927	6.213844113
C	-0.145349186	4.445842491	4.431121784
H	-0.400743779	5.413881766	4.885924606
H	0.895160504	4.160560063	4.672375314
C	-1.603598103	2.156084213	6.363699479
H	-2.286158842	2.120689239	7.227375625
H	-1.794100263	1.266331038	5.758590759
H	-0.584451866	2.077824009	6.759834650

14a *path Z*

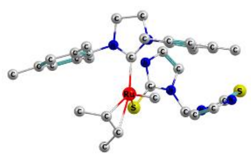

86
RuC33N6H44S2

Ru	-1.479215971	3.129963125	3.438510415
C	-1.180793331	3.818475140	1.524110667
N	-0.135011917	4.384042728	0.848158418
C	-0.346043245	4.563694484	-0.598836559
C	-1.840620990	4.271758335	-0.752059837
N	-2.175042724	3.597001639	0.527357919
H	-0.073313830	5.579033083	-0.906330241
H	0.279299619	3.864626009	-1.170310689
H	-2.427973668	5.190736878	-0.867992747
H	-2.069828243	3.623646703	-1.604519468
C	1.121346708	4.805126706	1.399107476
C	2.243064283	3.965047385	1.323092019
C	2.127090107	2.569005051	0.776594620
C	3.461412956	4.422918373	1.836425931
C	3.586252855	5.683159777	2.424313789
C	4.896087490	6.143814895	3.020161913
C	2.452839593	6.499590878	2.481207780
C	1.218381158	6.084234587	1.977754725
C	0.002789841	6.959499419	2.124277893
H	1.752757045	2.550734513	-0.250839681
H	1.424550394	1.999678112	1.387781455
H	3.086994309	2.048118116	0.788047126
H	4.330434927	3.770318012	1.779243340
H	5.067508808	7.209342457	2.835052467
H	5.744557093	5.587945097	2.609713253
H	4.902859924	6.002319762	4.108553853
H	2.527484958	7.483220538	2.940559578
H	-0.524324951	7.091441896	1.173966093
H	-0.695704249	6.487246545	2.822176878
H	0.271065996	7.947792178	2.507447298
C	-3.588134244	3.612406414	0.865956383
C	-4.177113884	4.785404285	1.394799919
C	-3.369761884	6.011379405	1.734920904
C	-5.549806892	4.796584299	1.650522298
C	-6.363120549	3.690456001	1.400432965
C	-7.832365675	3.710838575	1.748275111
C	-5.768586513	2.562285532	0.837896993
C	-4.399680695	2.504633703	0.551866376
C	-3.861992337	1.245838694	-0.082066373
H	-4.025453680	6.860052506	1.948048480
H	-2.750266891	5.806920861	2.613247430
H	-2.689941609	6.304770972	0.931172662
H	-5.992747122	5.696339012	2.071814363
H	-8.263656005	4.707983219	1.612586351
H	-8.401803899	3.005820071	1.134666286
H	-7.986020990	3.430498891	2.798024905
H	-6.385630669	1.697278561	0.604171057
H	-3.163319121	1.457071778	-0.898142719
H	-3.348938148	0.624901183	0.659184193
H	-4.677620554	0.651527656	-0.502655737
S	-1.571349079	0.687530670	3.025396269
C	-0.645494661	0.355941027	1.626283260
N	-0.782193719	0.872080269	0.378772772
C	0.134629729	0.306334354	-0.493772369
C	0.869284912	-0.581667503	0.218778215
N	0.382500238	-0.552010068	1.527070655
H	-1.380466483	1.685231039	0.220513092
H	0.185932909	0.586784200	-1.533220579
H	1.713706719	-1.198353398	-0.044471178
C	1.072348253	-1.110797653	2.683967580
H	1.569958782	-2.033886744	2.385699664
H	0.336132708	-1.293034319	3.465987722
S	4.144031020	-1.249776353	1.670015208
C	3.382738370	-0.179779720	2.726329939
N	2.078554711	-0.194029524	3.178860656
C	1.827291718	0.913876082	3.999382792
C	2.984074374	1.606097421	4.089717862
N	3.922892568	0.924657316	3.321796721
H	0.843329294	1.130496039	4.384302585
H	3.206546771	2.529256153	4.597836204
H	4.887265608	1.179840919	3.178715775
C	-2.707365101	3.930614924	5.102516000
H	-2.196392485	4.796484508	5.519449277
C	-4.158686632	4.191321264	4.750507049
H	-4.291549955	5.177357124	4.298407531
H	-4.561896975	3.455131382	4.047556033
H	-4.789678955	4.152193192	5.652329094
C	-2.265315822	2.647319639	5.512262619
H	-1.407828346	2.618043139	6.186238970
C	0.022039269	3.838256845	4.224152887
H	0.081022810	4.013212343	5.313444730
H	0.978361410	3.998916886	3.718851978
C	-3.212434182	1.475612732	5.702068457
H	-3.769868416	1.560652330	6.648092484
H	-3.953092343	1.398520469	4.899149565
H	-2.669846050	0.524042583	5.733918913

11a‡ (280.43*i* cm^{-1}) *path self*

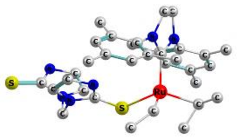

86

RuC33N6H44S2

Ru	-1.007118999	4.078223253	3.529410042
C	-1.109005464	4.694965955	1.649984804
N	-0.124747096	5.502667691	1.090785049
C	-0.372925934	5.848505323	-0.311518380
C	-1.877519016	5.576065461	-0.419475762
N	-2.084826662	4.612703343	0.670079339
H	-0.111489958	6.891267081	-0.520174679
H	0.205162862	5.206627709	-0.993312211
H	-2.466952782	6.489647928	-0.248331913
H	-2.170610885	5.156419203	-1.386135141
C	1.187016692	5.524991772	1.656990269
C	2.034798467	4.404776614	1.532582841
C	1.619567826	3.198570198	0.724659628
C	3.285178026	4.446004200	2.156085838
C	3.715048562	5.563898684	2.877216880
C	5.049414681	5.563375604	3.586692174
C	2.870726538	6.676042687	2.946076507
C	1.610094466	6.677749115	2.343221705
C	0.717518657	7.892049044	2.407686036
H	1.530115719	3.447645378	-0.339484757
H	0.653604197	2.820839478	1.067251923
H	2.348827816	2.389752335	0.819017443
H	3.941924501	3.581945194	2.072225573
H	5.492834950	6.564181242	3.609474902
H	5.762204598	4.887298837	3.103592337
H	4.939170925	5.232973299	4.627429341
H	3.197821531	7.561242417	3.487728226
H	0.700479204	8.419437925	1.444786224
H	-0.309191769	7.602775140	2.636444956
H	1.063290958	8.598795839	3.167260508
C	-3.278070487	3.840301556	0.748482441
C	-4.381872206	4.281116530	1.501674209
C	-4.345968882	5.626646275	2.176217544
C	-5.517795177	3.469121471	1.562863223
C	-5.599641161	2.261109991	0.861081293
C	-6.830907426	1.391419424	0.963949597
C	-4.510389385	1.875194272	0.074269569
C	-3.340871736	2.642981517	0.010423203
C	-2.169669273	2.201935123	-0.836596989
H	-5.111503989	5.703238795	2.953471367
H	-3.368982080	5.801764488	2.625079723
H	-4.533867780	6.425011740	1.446318323
H	-6.367214905	3.797544273	2.158236374
H	-7.744833074	1.993670740	0.993716569
H	-6.907764622	0.700825336	0.118563320
H	-6.810987054	0.789267217	1.881065112
H	-4.572948933	0.958123947	-0.509684986
H	-1.230528698	2.278691756	-0.282313595
H	-2.289477177	1.167593094	-1.170240122
H	-2.065291559	2.828029407	-1.731146349
S	0.269428340	1.825874714	3.784670118
C	-0.566164926	0.677547002	2.861127032
N	-1.726606605	0.879437732	2.187008320
C	-2.137584985	-0.279146358	1.542753418
C	-1.216342161	-1.233709423	1.815562914
N	-0.254823492	-0.644213065	2.640684542
H	-2.173147951	1.794986753	2.177805438
H	-3.038060000	-0.306409979	0.953553097
H	-1.113075410	-2.256462827	1.489340681
C	0.927235243	-1.290299690	3.172257298
H	0.727639146	-2.357754962	3.275015235
H	1.151689081	-0.839407803	4.140580936
S	1.627688790	-3.455256977	0.873164703
C	2.405454789	-2.027098030	1.303892724
N	2.082772292	-1.138868328	2.310046854
C	2.970774780	-0.061388610	2.323917157
C	3.871709278	-0.278911057	1.338290921
N	3.518082447	-1.477988048	0.729087025
H	2.840617124	0.758987037	3.011788635
H	4.719002123	0.305353352	1.018250858
H	3.984332377	-1.925888807	-0.044249475
C	-2.609857959	3.592658474	4.406235462
H	-3.421954600	4.325376612	4.508680938
C	-3.070274255	2.238680956	4.895151260
H	-3.858281059	1.866190162	4.222393559
H	-2.268834078	1.499084270	4.935083597
H	-3.522282267	2.312880846	5.895352413
C	-1.161448489	5.839252170	4.726707002
H	-0.130056702	6.209973495	4.672351419
C	-1.356017702	4.767382496	5.703608753
H	-0.505746921	4.346947585	6.236694358
H	-2.246868425	4.791695809	6.328653066
C	-2.189566408	6.956326126	4.680572611
H	-2.136045589	7.531090340	3.749389342
H	-3.208182796	6.559993317	4.760213239
H	-2.057633916	7.665799696	5.512078912

12a *path self*

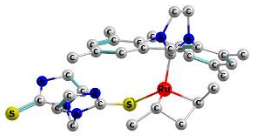

86

RuC33N6H44S2

Ru	-0.594407414	4.078693195	3.461315972
C	-0.981619388	4.998044696	1.845354010
N	-0.110304721	5.977350771	1.359474181
C	-0.463850499	6.448269114	0.017348581
C	-1.932806125	6.005869746	-0.092905790
N	-2.026114482	4.961989457	0.936944762
H	-0.341302861	7.533376896	-0.068182804
H	0.164755915	5.978355153	-0.754501085
H	-2.622576739	6.833644814	0.124320180
H	-2.183693323	5.605523185	-1.080829154
C	1.240752956	6.045612535	1.836707742
C	2.167839805	5.031938624	1.503095972
C	1.828429853	3.880522213	0.585191679
C	3.461611169	5.102770624	2.030237619
C	3.870476133	6.155358408	2.849906712
C	5.260210362	6.188297910	3.441339553
C	2.955686686	7.178104664	3.110638498
C	1.649384685	7.146548287	2.616811487
C	0.719294565	8.289129808	2.941286828
H	2.677511105	3.657084631	-0.069937490
H	0.961063546	4.086970611	-0.040700710
H	1.600385116	2.980458860	1.165073581
H	4.171918780	4.317403810	1.777392125
H	5.605478660	7.215059022	3.599443701
H	5.984765831	5.680722519	2.796355086
H	5.281187896	5.684204862	4.415565146
H	3.265346627	8.027147746	3.717162955
H	-0.078712824	8.391756419	2.203942173
H	0.241196183	8.131161433	3.912285742
H	1.272663647	9.232359035	2.991670162
C	-3.070696554	3.992726415	0.901716078
C	-4.374208200	4.327491519	1.304202129
C	-4.718897678	5.719110164	1.772671677
C	-5.371712552	3.346862176	1.240949104
C	-5.113467322	2.059418687	0.769988775
C	-6.192612469	1.001871391	0.755730868
C	-3.818262819	1.767128672	0.324167791
C	-2.792323848	2.714438698	0.373163121
C	-1.413979784	2.386132703	-0.144766091
H	-5.456226302	5.690557848	2.580869182
H	-3.836776181	6.245664355	2.134298283
H	-5.153770029	6.307061579	0.953865668
H	-6.376088305	3.600187139	1.574727004
H	-7.188442614	1.443774122	0.652288766
H	-6.049251212	0.292397823	-0.065915920
H	-6.190243567	0.425714681	1.689914638
H	-3.606719767	0.784830905	-0.094641873
H	-0.665113628	2.478697662	0.646696793
H	-1.373638258	1.369140948	-0.544754692
H	-1.119753638	3.079867423	-0.940278546
S	0.741256326	1.886336096	3.590673232
C	-0.306614789	0.611965954	3.157160210
N	-1.535245238	0.744682731	2.614643409
C	-2.108442510	-0.491106622	2.364934509
C	-1.207181682	-1.428920043	2.748123286
N	-0.099938587	-0.740757572	3.250480868
H	-1.953612229	1.667610136	2.505270528
H	-3.093195097	-0.582758127	1.938972777
H	-1.210505584	-2.506470962	2.695516915
C	1.131566258	-1.346562978	3.722081656
H	0.898377844	-2.307178555	4.183236104
H	1.586744517	-0.666680898	4.443552161
S	1.251449541	-4.191900641	2.180730834
C	2.129628864	-2.777188253	1.939910667
N	2.066262539	-1.590859764	2.643803612
C	2.937479977	-0.644188342	2.100111420
C	3.574060433	-1.240993432	1.066933543
N	3.078180978	-2.537537354	0.984832953
H	2.988633785	0.357525256	2.497119132
H	4.325856446	-0.865820908	0.391809743
H	3.342036910	-3.246106222	0.318127779
C	-2.481321318	3.715187230	4.350651774
H	-3.274902621	4.228074843	3.800131086
C	-2.984569871	2.347109848	4.802105638
H	-3.507251212	1.803599229	4.005818927
H	-2.172658928	1.708550817	5.173109785
H	-3.708815652	2.456428070	5.625965680
C	-1.222523227	5.668052969	4.681495115
H	-0.318277707	6.059245866	5.174164098
C	-1.987326836	4.616281005	5.497536596
H	-1.316451511	4.048476520	6.163578137
H	-2.789568238	5.011349301	6.151828691
C	-2.140085422	6.807054329	4.248434591
H	-1.794230672	7.316327799	3.345558638
H	-3.145974209	6.427938461	4.045372873
H	-2.247900995	7.565247034	5.040581801

13a‡ (313.36*i* cm^{-1}) *path self*

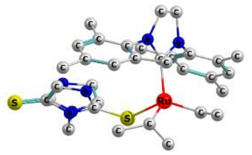

86
RuC33N6H44S2

Ru	-0.965401060	4.198936262	3.577650444
C	-1.072962388	4.650534916	1.630445764
N	-0.090408718	5.449876147	1.066053673
C	-0.354148654	5.836940411	-0.322706324
C	-1.848963938	5.518264340	-0.446393116
N	-2.046727834	4.563731086	0.654288378
H	-0.129662776	6.894918883	-0.495920385
H	0.250496655	5.241286644	-1.022664769
H	-2.469230973	6.414118271	-0.295400666
H	-2.116701762	5.077760850	-1.411209624
C	1.230722578	5.467545808	1.608667483
C	2.077686504	4.354117895	1.429348878
C	1.627895946	3.161362353	0.621069763
C	3.345499017	4.385877417	2.011862481
C	3.790561934	5.487770424	2.750741909
C	5.149450155	5.467792751	3.411292331
C	2.940867969	6.589115737	2.882216732
C	1.661002563	6.599925363	2.317940447
C	0.749623543	7.793929064	2.458904700
H	1.486723164	3.422816566	-0.434679048
H	0.678209834	2.779840778	1.003763507
H	2.359366937	2.350392783	0.669913134
H	4.004997214	3.528721068	1.885426271
H	5.514382130	6.479192440	3.613963125
H	5.891472992	4.958327525	2.786906321
H	5.111116184	4.934337097	4.369551782
H	3.277394261	7.458360489	3.443521702
H	-0.257240245	7.470563599	2.733978738
H	1.120211367	8.486912841	3.219639541
H	0.671402538	8.346451610	1.513360264
C	-3.261503847	3.830526010	0.765849498
C	-4.339065433	4.327311083	1.521271535
C	-4.257055222	5.694011351	2.153084167
C	-5.493506262	3.546985066	1.622747556
C	-5.615648597	2.315763977	0.968182390
C	-6.856007516	1.470851753	1.141682665
C	-4.556318089	1.882175495	0.165548934
C	-3.375197902	2.624775287	0.047136728
C	-2.253086310	2.156366598	-0.851950582
H	-5.052357106	5.837160190	2.889931854
H	-3.290167241	5.833713293	2.641640295
H	-4.369067159	6.474340936	1.389086571
H	-6.322802894	3.912002140	2.225116418
H	-7.756858364	2.089509768	1.208267350
H	-6.986139909	0.769443020	0.311757524
H	-6.799377279	0.881600451	2.065649162
H	-4.653761098	0.949987982	-0.388759595
H	-1.279808671	2.254112521	-0.364240170
H	-2.388981903	1.111930942	-1.145921682
H	-2.211671835	2.752005552	-1.772330526
S	0.228880618	1.933900415	3.726633423
C	-0.529821903	0.713966531	2.826332986
N	-1.646965513	0.854337407	2.071206729
C	-2.001131135	-0.344930097	1.469899559
C	-1.080422524	-1.263878830	1.846347856
N	-0.181720342	-0.611270373	2.695217288
H	-2.138447380	1.741000866	2.015123555
H	-2.871624758	-0.425921444	0.841421434
H	-0.941599128	-2.301587287	1.586408991
C	0.996594385	-1.199546000	3.296501873
H	0.810882445	-2.259313418	3.476503921
H	1.197656285	-0.674223340	4.231653431
S	1.814837707	-3.564911772	1.241079390
C	2.528269491	-2.068510133	1.529231156
N	2.161438246	-1.097452653	2.439367235
C	2.995450777	0.017897170	2.338624488
C	3.909409408	-0.257893583	1.379805993
N	3.617459190	-1.530586316	0.901238267
H	2.826179067	0.897584906	2.939917900
H	4.731143044	0.328088648	1.001647221
H	4.106356523	-2.033519152	0.177242839
C	-2.776057550	3.673303758	4.564091590
H	-3.577374396	4.394054613	4.387013560
C	-3.309054695	2.253867065	4.472923023
H	-3.770276364	2.065427207	3.496497399
H	-2.523463775	1.512403459	4.641557578
H	-4.092096265	2.081006910	5.228198594
C	-1.097406080	5.787397667	4.626190365
H	-0.079827502	6.101084447	4.934090802
C	-1.886194700	4.038436320	5.664154744
H	-1.285071843	3.244045820	6.109557545
H	-2.186870809	4.775162421	6.403063763
C	-2.122523136	6.800520691	5.072928343
H	-1.984472268	7.707790067	4.466702566
H	-3.151771899	6.461119195	4.921662861
H	-2.008309061	7.104633373	6.123966369

14a *path self*

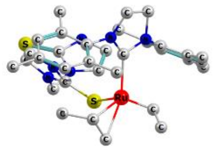

86
RuC33N6H44S2

Ru	-1.120429371	4.086564658	3.742614504
C	-1.178233145	4.514471066	1.644426139
N	-0.175499566	5.266189984	1.090472218
C	-0.458950587	5.755725786	-0.269532182
C	-1.956282098	5.455921454	-0.401429981
N	-2.182684149	4.521018201	0.717991485
H	-0.225805347	6.821051727	-0.364414070
H	0.144801439	5.207649779	-1.005937288
H	-2.571413800	6.355973011	-0.269229673
H	-2.223683371	4.999881138	-1.359291964
C	1.169053139	5.235406521	1.573683011
C	1.939756394	4.069171391	1.388259836
C	1.374559158	2.878852622	0.652658346
C	3.245571657	4.055015845	1.877052165
C	3.804699783	5.165019904	2.521761741
C	5.206434154	5.098450734	3.081839854
C	3.031529142	6.320610460	2.645949374
C	1.716015243	6.380875211	2.170936020
C	0.921927487	7.661277586	2.263894842
H	1.131767675	3.132779092	-0.386755359
H	0.454998955	2.534947265	1.130041067
H	2.085858749	2.049400039	0.640776653
H	3.844535416	3.157305933	1.741381566
H	5.646711627	6.094487906	3.187931208
H	5.866310231	4.503907136	2.440256773
H	5.210434559	4.630666377	4.074523517
H	3.457863652	7.199335762	3.125642821
H	-0.147337475	7.456440155	2.348143194
H	1.230080701	8.255023542	3.129500273
H	1.078894225	8.279561105	1.370113268
C	-3.459267899	3.903533927	0.872065317
C	-4.470012516	4.524541391	1.626298491
C	-4.238105218	5.873277858	2.259691936
C	-5.695449080	3.865251169	1.752160146
C	-5.941805792	2.631712851	1.139459908
C	-7.249343901	1.909358423	1.363756538
C	-4.939002058	2.073011940	0.341726881
C	-3.697424896	2.699106968	0.184468784
C	-2.656438068	2.126525095	-0.752671341
H	-5.042553092	6.121949195	2.957271306
H	-3.286538180	5.883660245	2.798202758
H	-4.206255182	6.661356529	1.496187443
H	-6.476561718	4.322312492	2.355850398
H	-8.087023783	2.609623641	1.444309211
H	-7.468352695	1.207604023	0.553058863
H	-7.216583274	1.334101316	2.297697891
H	-5.127626433	1.132992323	-0.173463243
H	-1.655619348	2.129424458	-0.313200965
H	-2.907083093	1.103071929	-1.045741827
H	-2.597711784	2.715823330	-1.676353842
S	0.119163605	1.790457432	3.931966020
C	-0.406373725	0.610104461	2.840832412
N	-1.473614457	0.688072993	2.000358891
C	-1.614862450	-0.480110083	1.260155246
C	-0.609853284	-1.307542976	1.629111871
N	0.126067559	-0.639088887	2.610640783
H	-2.089077614	1.492972000	1.995853432
H	-2.409650208	-0.611347620	0.545924363
H	-0.312716499	-2.286080288	1.285874193
C	1.302691586	-1.149736479	3.282544132
H	1.228575715	-2.236366989	3.345473235
H	1.343497204	-0.703201705	4.277298282
S	2.590390425	-3.179641340	1.113760512
C	3.106050352	-1.666314477	1.638267886
N	2.529817172	-0.834772306	2.577610716
C	3.255767337	0.351063218	2.703362804
C	4.309284886	0.259972375	1.858801108
N	4.207739532	-0.971789524	1.221156333
H	2.922726295	1.144758855	3.353926088
H	5.111888356	0.950737319	1.658410873
H	4.834057508	-1.349405285	0.527434280
C	-3.014381793	3.496024813	4.544307488
H	-3.735970336	4.311706269	4.467833136
C	-3.595697783	2.165832695	4.100165201
H	-3.894954183	2.169602269	3.047250040
H	-2.879879468	1.354224032	4.263965349
H	-4.499408577	1.921385610	4.680705437
C	-1.118666411	5.800662069	4.437407866
H	-0.115643538	6.258342208	4.321660894
C	-2.093014703	3.528891457	5.641834884
H	-1.721599028	2.583631828	6.037241276
H	-2.154744068	4.313895367	6.390324171
C	-2.035222383	6.673605318	5.249592617
H	-2.138065804	7.659402929	4.769852181
H	-3.038319164	6.250983685	5.362207852
H	-1.628667413	6.860369446	6.255720174

9e

77

RuC30N6H38S2

Ru	0.781923776	1.809408701	0.850550815
C	-0.168047698	0.977278465	-0.544787425
H	-0.955942845	1.513858319	-1.096013389
C	-0.521611654	3.300963729	0.779981087
N	-0.800812823	4.105598918	-0.305632476
C	-1.678286815	5.242199066	0.012627529
C	-2.309092721	4.791995753	1.329012005
N	-1.349295525	3.775013955	1.786870191
H	-1.081369461	6.158709396	0.132359535
H	-2.412405514	5.415751132	-0.779288651
H	-2.414811217	5.604983555	2.053675596
H	-3.301217289	4.340598950	1.178220179
C	-0.033656916	4.170745938	-1.510889604
C	-0.668005427	3.840210168	-2.723107273
C	-2.119124822	3.420299324	-2.767427321
C	0.062416942	3.924806156	-3.911671343
C	1.393728603	4.344708922	-3.927070070
C	2.185689104	4.385164341	-5.213618524
C	1.982860288	4.712542235	-2.714537416
C	1.293130881	4.641514155	-1.501728938
C	1.993685926	5.055401772	-0.231462520
H	-2.776058041	4.283554868	-2.942164179
H	-2.428857370	2.952714713	-1.830248879
H	-2.296328680	2.711041828	-3.581828365
H	-0.422178095	3.650856761	-4.847169162
H	1.531193338	4.461537024	-6.087745215
H	2.787794321	3.475411042	-5.333206913
H	2.877320393	5.234451567	-5.232547668
H	3.012218742	5.067102327	-2.709849089
H	1.295105062	5.375557291	0.544742636
H	2.553359244	4.207881582	0.178545698
H	2.698099708	5.871074224	-0.427174651
C	-1.582271076	3.146893938	3.047734018
C	-1.179867958	3.827997053	4.216071278
C	-0.486390271	5.165339964	4.129897184
C	-1.438864411	3.249262544	5.460232675
C	-2.098839106	2.022248765	5.574717223
C	-2.370142968	1.424174385	6.935595744
C	-2.516342603	1.383883092	4.403977210
C	-2.286389696	1.930516671	3.134763870
C	-2.772451330	1.218656701	1.899817648
H	0.052774591	5.385543248	5.056731162
H	0.224246955	5.181191101	3.301480384
H	-1.206922912	5.978809104	3.974067063
H	-1.110369647	3.764302823	6.360858393
H	-1.482540158	1.484907606	7.575718027
H	-3.178165268	1.955790742	7.453494855
H	-2.664477300	0.372331892	6.862664455
H	-3.055097773	0.440329227	4.475508986
H	-3.283803834	1.905591480	1.216938336
H	-1.914919654	0.817083703	1.345119525
H	-3.462574099	0.409377339	2.156808710
S	2.098938505	3.043919091	2.833924693
C	1.750975521	2.277621818	4.290317370
N	2.038888410	2.787577441	5.530108344
C	1.608666046	1.944461558	6.542681086
C	1.041541121	0.878946920	5.934259293
N	1.124047572	1.078999790	4.555821087
H	2.489410574	3.682587933	5.636762438
H	1.729241458	2.181993575	7.587058127
H	0.555810371	0.011072425	6.348755519
C	0.684648627	0.108897222	3.570268702
H	0.406525017	0.637691867	2.604961663
H	-0.193301497	-0.399805450	3.971572043
S	2.623114947	0.101204238	0.812533774
C	2.569216424	-0.870014806	2.191750115
N	1.715708362	-0.870865634	3.280935869
C	2.063292738	-1.902905246	4.157542295
C	3.122143542	-2.555909116	3.630027580
N	3.422382882	-1.916674763	2.437680355
H	1.517550322	-2.073954684	5.071026468
H	3.676561864	-3.408667324	3.987228993
H	4.149106002	-2.163125306	1.784175750
C	-0.107673676	-0.430564189	-1.092490170
H	0.642251784	-1.062123340	-0.610931211
H	-1.089903449	-0.922953156	-0.992323504
H	0.108211874	-0.409000570	-2.173802991

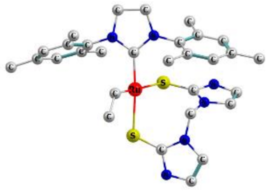

9e’

77

RuC30N6H38S2

Ru	-0.211488459	1.520223715	1.403456960
C	-0.869215942	0.957853756	-0.264851098
H	-1.570299611	1.580341803	-0.840619336
C	-0.900517539	3.330452942	0.988977933
N	-0.766066973	4.070179580	-0.175921900
C	-1.288788337	5.440915295	-0.066858535
C	-2.150348834	5.355386875	1.198362276
N	-1.635273077	4.135274569	1.829920221
H	-0.464848903	6.160765099	0.045469770
H	-1.860305694	5.720597439	-0.958101380
H	-2.035902175	6.225688688	1.852170675
H	-3.219351867	5.244233481	0.964498424
C	0.174745852	3.779452034	-1.212675676
C	-0.293478809	3.409942370	-2.483666801
C	-1.768846289	3.346968802	-2.803200186
C	0.639019937	3.107296986	-3.484720285
C	2.012415474	3.183309646	-3.258496644
C	3.005185917	2.795883322	-4.330267109
C	2.451421072	3.620084825	-2.001867476
C	1.558254530	3.937481271	-0.977781990
C	2.070540004	4.465961256	0.339576007
H	-2.061153071	4.189011915	-3.444300006
H	-2.376218520	3.379522180	-1.897460674
H	-2.012445131	2.426399434	-3.343524190
H	0.275368623	2.798685571	-4.463294144
H	2.584809993	2.918876909	-5.333369106
H	3.303090088	1.743965998	-4.230325917
H	3.918212463	3.398210467	-4.269572695
H	3.519790328	3.735610774	-1.822728699
H	1.711466551	5.485466010	0.526490064
H	1.726804017	3.850949919	1.174382377
H	3.164944751	4.496350805	0.350222520
C	-2.072388084	3.752373217	3.133245152
C	-1.480602075	4.375213884	4.246259929
C	-0.421615557	5.438618341	4.075996203
C	-1.894909406	3.994585405	5.526791352
C	-2.877401182	3.022716701	5.719629508
C	-3.272202929	2.579628829	7.109587492
C	-3.472556886	2.445191671	4.592227637
C	-3.093605726	2.795067774	3.294770786
C	-3.740148167	2.145180172	2.097799107
H	0.225517840	5.487490315	4.956451146
H	0.204513393	5.235179561	3.204665191
H	-0.872564158	6.431445134	3.944432179
H	-1.425922570	4.459992807	6.391773500
H	-2.759143463	1.649178450	7.384334619
H	-3.012378628	3.331614982	7.861395537
H	-4.348317188	2.387272607	7.179816640
H	-4.252078956	1.697746289	4.727004910
H	-4.028468617	2.888394419	1.345587416
H	-3.020822062	1.469017435	1.616627043
H	-4.632268940	1.582939341	2.389851757
S	0.478613947	-0.851051370	2.132040654
C	1.880190454	-1.248048897	1.283714410
N	2.330439123	-2.518666313	1.030123629
C	3.504040271	-2.508543254	0.291503986
C	3.810512511	-1.208332959	0.078374579
N	2.815954957	-0.432873589	0.676482847
H	1.808325129	-3.328241843	1.326850010
H	4.000725534	-3.412551842	-0.021761771
H	4.625189090	-0.758059482	-0.465969714
C	2.843070796	1.011283090	0.784432043
H	1.768562687	1.397055739	0.745711702
H	3.410867419	1.413715972	-0.055876404
S	1.232797999	2.422926482	3.419047529
C	2.828844694	1.944325924	3.148243573
N	3.463643565	1.431112038	2.034038910
C	4.824658958	1.261009032	2.299045945
C	5.056083415	1.671488251	3.565761953
N	3.833692011	2.084879634	4.069657443
H	5.493913510	0.853950099	1.558924491
H	5.965281678	1.707934020	4.143824064
H	3.648490569	2.444401154	4.993047553
C	-0.508967045	-0.227141559	-1.133547458
H	0.136506608	-0.964219382	-0.650813208
H	-1.415327647	-0.751742195	-1.478842843
H	0.001988604	0.128716430	-2.044376452

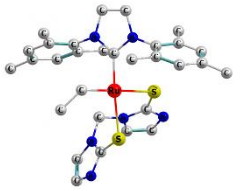

10e

86
RuC33N6H44S2

Ru	0.978624600	1.959625701	0.803597128
C	-0.252727000	0.645671602	0.130237413
H	-1.269152753	0.903163672	-0.212439272
C	-0.598069125	3.162815698	0.734073442
N	-1.157539637	3.813279465	-0.349942146
C	-2.492820476	4.381012966	-0.100039521
C	-2.603411346	4.296960732	1.419654883
N	-1.513324603	3.368618863	1.756836131
H	-2.553368880	5.406804991	-0.476458793
H	-3.264417866	3.786607112	-0.607393714
H	-2.451492851	5.271577796	1.904615862
H	-3.564028993	3.897274169	1.760633844
C	-0.474834844	4.268631860	-1.517261395
C	-0.694703653	3.640085316	-2.752387399
C	-1.548878643	2.402021885	-2.841565462
C	-0.030436588	4.138826421	-3.876846126
C	0.839413812	5.230194016	-3.795163134
C	1.601705721	5.705307718	-5.010095861
C	1.018045314	5.849163313	-2.554234686
C	0.363691490	5.392589594	-1.408391220
C	0.575543063	6.062621749	-0.072644535
H	-1.631878899	2.054187694	-3.875076602
H	-2.561074571	2.570033364	-2.457526686
H	-1.105672821	1.606577858	-2.234001333
H	-0.178190200	3.646934146	-4.836185951
H	1.046666495	5.515505122	-5.934364715
H	2.562953387	5.182972465	-5.093171195
H	1.818136777	6.777148927	-4.955851825
H	1.688546969	6.702478106	-2.475423937
H	1.026781098	5.364939740	0.639742713
H	1.231293846	6.932342115	-0.167529233
H	-0.372477387	6.398776133	0.364801243
C	-1.468599108	2.944758736	3.120670425
C	-0.973801495	3.825619573	4.103781951
C	-0.408803991	5.178662745	3.744279982
C	-1.053899220	3.449085812	5.447594275
C	-1.647372481	2.248818357	5.845153671
C	-1.681301163	1.849004183	7.301603504
C	-2.190227674	1.424030079	4.856003257
C	-2.118090359	1.752956496	3.497429437
C	-2.708308286	0.839539640	2.458619635
H	0.424958880	5.437795336	4.402045733
H	-0.041924052	5.200725717	2.718873900
H	-1.175471773	5.957497472	3.856926661
H	-0.647157791	4.118006802	6.204075605
H	-0.729704315	1.388542145	7.595936765
H	-1.838282256	2.715122339	7.953212690
H	-2.477495704	1.125402360	7.503405889
H	-2.695085722	0.503946639	5.147051864
H	-3.379859486	1.380173130	1.782838325
H	-1.908805418	0.432181021	1.826945897
H	-3.269417291	0.021650303	2.920921894
S	2.029376697	2.999049961	2.969414589
C	1.885558126	2.029687858	4.336826348
N	2.408636134	2.357438236	5.564274782
C	2.184982742	1.363666665	6.504461379
C	1.494858932	0.389261512	5.872552602
N	1.307419315	0.791328674	4.548467635
H	2.919305343	3.215402329	5.700330931
H	2.528291387	1.439523577	7.523448956
H	1.118028670	-0.554685245	6.231558949
C	0.575985416	-0.001387077	3.582750621
H	0.376623206	0.612088980	2.685325536
H	-0.369201788	-0.313882802	4.026236312
S	3.037541564	0.041046735	1.408765228
C	2.333372467	-1.217372262	2.274387532
N	1.295660260	-1.195086725	3.179822025
C	1.038403936	-2.479371801	3.655998705
C	1.913118852	-3.320377946	3.056998582
N	2.696811950	-2.538203225	2.222159255
H	0.244867996	-2.671845496	4.360609322
H	2.039914486	-4.387334378	3.143718439
H	3.426352855	-2.855440342	1.602759622
C	0.022601357	-0.783430944	-0.303483928
H	1.051394612	-1.103260740	-0.124050187
H	-0.647567363	-1.491126943	0.216043075
H	-0.177168177	-0.919269306	-1.380235040
C	2.087394881	2.448802006	-1.049860891
C	2.825794463	3.088654396	-0.031476855
H	2.751577430	4.164086762	0.102343788
H	1.468709162	3.079246901	-1.668672361
H	3.742765104	2.644574136	0.352010282
C	2.597288957	1.240834485	-1.811020151
H	3.207420034	0.578375850	-1.192725770
H	1.769817879	0.649941885	-2.217712919
H	3.218121860	1.573976012	-2.657251740

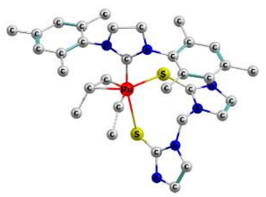

11e‡ (179.07*i* cm⁻¹)

86
RuC33N6H44S2

Ru	0.289503882	1.817083063	0.377578018
C	-0.660881178	0.267437234	-0.210662212
H	-1.124665063	0.132788274	-1.202038491
C	-0.929601645	3.340827992	0.786721551
N	-1.091167106	4.525522691	0.108592715
C	-2.323540381	5.245667879	0.459044456
C	-2.713241088	4.587275172	1.788101313
N	-1.940102253	3.338697710	1.740123533
H	-2.139396315	6.321268051	0.549536984
H	-3.094112160	5.098194012	-0.310844121
H	-2.416539857	5.203380994	2.648638293
H	-3.787846220	4.389487256	1.869176832
C	-0.379724796	4.891707415	-1.075870097
C	-0.770155437	4.379942924	-2.324549865
C	-1.905513676	3.390893593	-2.428939152
C	-0.060159117	4.785285538	-3.457774817
C	1.008774508	5.683518357	-3.376083193
C	1.794114596	6.066783352	-4.609195290
C	1.352771642	6.196511450	-2.122538308
C	0.669506960	5.818171275	-0.963551867
C	1.052885743	6.383023104	0.382218327
H	-2.000130811	3.011374840	-3.450435214
H	-2.865600402	3.844706013	-2.153690757
H	-1.730337445	2.549793813	-1.749676371
H	-0.343800216	4.382693390	-4.428369638
H	1.162261696	6.075525451	-5.503443777
H	2.606716159	5.352394088	-4.792057152
H	2.249288883	7.057090719	-4.504451763
H	2.174283561	6.906023218	-2.043654042
H	1.396774587	5.585102395	1.047200042
H	1.855613925	7.119707458	0.282906668
H	0.202730254	6.872157360	0.871384584
C	-1.897025677	2.487724219	2.882938390
C	-1.098600921	2.825614396	3.995412316
C	-0.226067868	4.058022215	3.983040111
C	-1.117486706	1.981910491	5.110340041
C	-1.894887448	0.817218171	5.141184180
C	-1.902728543	-0.063031802	6.371025986
C	-2.665808785	0.501176369	4.017633051
C	-2.690436689	1.328684399	2.888011378
C	-3.549449348	1.007872657	1.691427197
H	0.566177190	3.977745791	4.732854088
H	0.237364602	4.198724599	3.003383585
H	-0.805823520	4.960794006	4.212658260
H	-0.497798883	2.232669596	5.969338541
H	-0.886006137	-0.257790455	6.731812658
H	-2.449818033	0.412860639	7.193819849
H	-2.380432238	-1.027680867	6.173310993
H	-3.273065542	-0.401862509	4.022344058
H	-4.351485339	1.747382416	1.578869544
H	-2.947310477	1.040170685	0.779082439
H	-4.008979577	0.020052096	1.786335088
S	2.451855402	3.051030628	1.252602619
C	2.623291712	2.665603800	2.889939806
N	3.346764405	3.386615266	3.798602321
C	3.253674682	2.840925790	5.069936067
C	2.451344470	1.756713072	4.963116865
N	2.072385039	1.648706075	3.629164002
H	3.860495838	4.210347029	3.527350761
H	3.766687683	3.259547520	5.920138568
H	2.140860688	1.030972236	5.695770463
C	1.313901201	0.534792854	3.078186089
H	1.474400995	0.534243185	1.990260367
H	0.251997281	0.673386978	3.276916292
S	4.340896116	-0.690740944	2.857446138
C	2.957436185	-1.299328994	3.573544501
N	1.703501754	-0.722531635	3.677005064
C	0.822262471	-1.570632890	4.350526961
C	1.514337335	-2.683295696	4.686889670
N	2.808933192	-2.503195940	4.219049465
H	-0.209929108	-1.297677277	4.498368036
H	1.203780237	-3.575110745	5.206579122
H	3.578236300	-3.150026130	4.295316350
C	-0.857738419	-0.987860457	0.619615563
H	-0.509770226	-0.893313027	1.653479418
H	-1.923537057	-1.255944554	0.659232054
H	-0.333636196	-1.846702034	0.172911904
C	1.327999689	0.402942585	-1.194985444
C	1.184157059	1.799354422	-1.549425174
H	0.525328019	2.034264719	-2.384680608
H	0.803381842	-0.310006516	-1.827366237
H	2.080474053	2.420948836	-1.534092001
C	2.597489874	-0.185820010	-0.613229720
H	3.175324264	0.545418430	-0.044072363
H	2.383003336	-1.025866812	0.058855606
H	3.243122130	-0.579045470	-1.414132179

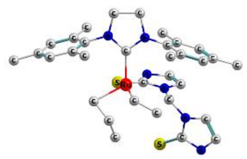

12e

86

RuC33N6H44S2

Ru	0.616271524	1.994094826	0.635437807
C	-0.333392063	0.699621921	-0.738218078
H	-1.415779263	0.815578128	-0.885891975
C	-0.844807689	3.204992710	0.489682432
N	-1.317079593	4.105741180	-0.445402141
C	-2.497314847	4.845595354	0.031056433
C	-3.005919741	3.951094977	1.169914971
N	-1.786437129	3.222191027	1.512301129
H	-2.204983518	5.843099796	0.392993715
H	-3.230670102	4.975481560	-0.769777425
H	-3.388178820	4.520194638	2.023914570
H	-3.799124886	3.269789616	0.826326703
C	-0.577656019	4.628814059	-1.547744730
C	-1.042971823	4.375855891	-2.850825030
C	-2.293773788	3.561976971	-3.088444727
C	-0.318524547	4.883695859	-3.933032270
C	0.842760307	5.637758906	-3.750086606
C	1.651061542	6.123046227	-4.931118114
C	1.258560673	5.906993483	-2.442734228
C	0.565798559	5.420906393	-1.331472935
C	1.066332700	5.715074030	0.059985047
H	-2.274363611	3.096830444	-4.078338250
H	-3.194771925	4.188431379	-3.040607649
H	-2.400163915	2.774530805	-2.337553862
H	-0.662404942	4.669885845	-4.943108998
H	1.033580353	6.223456492	-5.829206170
H	2.458978649	5.418398131	-5.166359532
H	2.117104017	7.093042697	-4.728087110
H	2.151260744	6.508500611	-2.281898096
H	1.615901810	4.852810924	0.453164431
H	1.729901642	6.585473578	0.059407971
H	0.241510812	5.908499566	0.753171719
C	-1.620011132	2.438439861	2.692525688
C	-0.882169152	2.999899619	3.756625967
C	-0.345882415	4.404282084	3.622313265
C	-0.690625935	2.249791260	4.915908848
C	-1.237724536	0.966477233	5.054397224
C	-1.038338318	0.190720085	6.334622844
C	-1.981907163	0.445095724	3.995237438
C	-2.185931007	1.157968895	2.806369292
C	-3.018458677	0.576689134	1.691983365
H	0.131199549	4.732051646	4.550334195
H	0.373015632	4.461637326	2.797649590
H	-1.148811994	5.111609229	3.387909703
H	-0.113205464	2.672464801	5.735144764
H	0.023859334	0.037038950	6.555183593
H	-1.472101854	0.725427771	7.188115986
H	-1.515121189	-0.792920012	6.285816457
H	-2.421470813	-0.545888259	4.092447798
H	-4.045398402	0.963079044	1.723976226
H	-2.594432284	0.828012249	0.718869338
H	-3.076736239	-0.513021699	1.770823476
S	2.875542646	2.518065585	1.702898173
C	2.907956968	2.513557073	3.411207238
N	2.708256842	3.588151569	4.220687300
C	2.808090831	3.242653762	5.561151998
C	3.095586886	1.920006882	5.599512205
N	3.165821510	1.481175418	4.277601319
H	2.480048073	4.495034868	3.844385850
H	2.665088489	3.957669716	6.354741620
H	3.226923788	1.228432322	6.417147141
C	3.472672184	0.120547575	3.866222045
H	4.207093913	-0.293321683	4.559105104
H	3.870852946	0.165421963	2.851997313
S	2.583214659	-1.516848209	6.518457908
C	1.891214400	-1.449304999	4.981983496
N	2.311849419	-0.739269109	3.877961514
C	1.453066049	-0.947140347	2.798289459
C	0.492917891	-1.801752673	3.215220333
N	0.773798141	-2.099818868	4.544125998
H	1.588891126	-0.436604113	1.860732477
H	-0.359638510	-2.202820195	2.694161876
H	0.245042857	-2.702845214	5.154409805
C	-0.044517190	-0.761341435	-0.381904938
H	1.026738739	-0.948604932	-0.220351957
H	-0.575179410	-1.055224209	0.532094357
H	-0.353812292	-1.459076119	-1.176810441
C	0.451679952	1.267232362	-1.953747407
C	1.539510124	2.058676524	-1.224291834
H	1.778609980	3.034768992	-1.655116871
H	-0.198116145	1.982818381	-2.464275848
H	2.475778778	1.480480085	-1.117886064
C	0.945988749	0.265449075	-3.007060830
H	1.641112506	-0.463490344	-2.573327318
H	0.116171926	-0.294011973	-3.459003081
H	1.478897514	0.786140097	-3.812176781

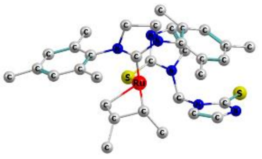

13e‡ (266.06*i* cm^{-1})

86

RuC33N6H44S2

Ru	0.525355184	1.696568532	0.120706261
C	-0.559418357	0.092185034	-0.731335557
H	-1.376517078	0.400453859	-1.392246994
C	-0.908449843	2.905074136	0.772663918
N	-1.256045314	4.085916270	0.126131070
C	-2.380348101	4.798785638	0.733976317
C	-3.030961605	3.691912875	1.561199931
N	-1.917390847	2.738219289	1.723432216
H	-2.031940967	5.635580996	1.360728099
H	-3.049110658	5.207536530	-0.030426150
H	-3.394348983	4.034046294	2.534402971
H	-3.869881014	3.232556030	1.020681791
C	-0.510706224	4.718448289	-0.908654303
C	-0.914164281	4.542378146	-2.240478568
C	-2.074325301	3.632224691	-2.557385950
C	-0.183703073	5.175444054	-3.250277223
C	0.929089848	5.970542581	-2.961230069
C	1.747485028	6.597238665	-4.066371950
C	1.296709903	6.142142441	-1.621961908
C	0.590518400	5.527971312	-0.586810198
C	1.011288861	5.680270386	0.853096335
H	-2.173555084	3.478376202	-3.635615151
H	-3.023486568	4.038598485	-2.186992191
H	-1.929752924	2.661615024	-2.072216278
H	-0.478160592	5.027718718	-4.287428985
H	1.177002358	6.674097975	-4.996843680
H	2.641886477	5.997482661	-4.277802378
H	2.089618819	7.601420987	-3.793782411
H	2.159905840	6.760693230	-1.382073209
H	1.171547145	4.692113306	1.295903454
H	1.926106018	6.279218665	0.931508690
H	0.242634399	6.177646362	1.456629247
C	-1.885533925	1.925024334	2.888512499
C	-0.958863724	2.203229817	3.919354429
C	0.109422082	3.251434928	3.745044167
C	-1.067582665	1.511966574	5.127025075
C	-2.061000216	0.551613085	5.343476475
C	-2.179350819	-0.149523814	6.678684538
C	-2.946234493	0.270393285	4.300516977
C	-2.883253498	0.947573850	3.076251680
C	-3.894923127	0.646555689	1.994399743
H	0.654375964	3.419032000	4.677492955
H	0.819715539	2.951110868	2.969066200
H	-0.320588081	4.202983768	3.411771762
H	-0.354229953	1.727059646	5.919914930
H	-1.233112349	-0.132960863	7.231157191
H	-2.926106696	0.339662029	7.316481646
H	-2.497387172	-1.192199777	6.563210574
H	-3.716870683	-0.485773482	4.442498289
H	-4.786027608	1.279834301	2.094654886
H	-3.476286483	0.822844608	1.000356111
H	-4.229636943	-0.393882358	2.050858177
S	3.213647495	1.951785072	0.670126701
C	3.436807454	2.603834790	2.219253015
N	3.456133223	3.922324590	2.573017550
C	3.613386458	4.080637247	3.943719162
C	3.716394270	2.837734926	4.470712310
N	3.622595545	1.937589029	3.410179246
H	3.308146451	4.651840546	1.893067657
H	3.629415765	5.050066886	4.414594648
H	3.813240175	2.486124178	5.486466879
C	3.686207205	0.492102415	3.534654567
H	4.353735008	0.243680077	4.361348479
H	4.062522663	0.096916917	2.590244910
S	2.680291909	-0.099955785	6.564990229
C	1.944569460	-0.405937885	5.078847091
N	2.399080150	-0.103174518	3.812824851
C	1.445988012	-0.446288074	2.849427978
C	0.401403099	-0.996487180	3.509182822
N	0.733598696	-0.993152306	4.856505763
H	1.602623478	-0.214080318	1.806796227
H	-0.556757329	-1.335109759	3.154668944
H	0.125378464	-1.251122348	5.616970249
C	-1.014552449	-0.964737304	0.268005371
H	-0.176979235	-1.407908301	0.813128175
H	-1.706394323	-0.551495835	1.006183173
H	-1.528824758	-1.795278352	-0.238655345
C	0.727864742	-0.069989160	-1.412517669
C	1.020602866	2.111241813	-1.632857174
H	0.378122139	2.110359836	-2.519395649
H	0.699880630	0.057186196	-2.492333345
H	2.024238046	2.504541178	-1.848590660
C	1.791646466	-1.082881716	-1.032762691
H	1.827749020	-1.298076144	0.037423922
H	1.627835538	-2.038973650	-1.555257471
H	2.785502903	-0.721781052	-1.315518224

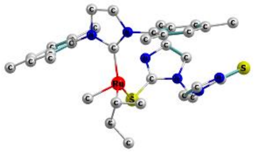

14e

86
RuC33N6H44S2

Ru	0.272299633	1.457353135	0.029417623
C	-0.904069128	0.235888033	-1.364294491
H	-0.528480296	0.486026627	-2.357659078
C	-1.074153495	2.886593727	0.466796838
N	-1.255909152	4.188953390	0.059132768
C	-2.461668846	4.824543220	0.611108779
C	-2.764454515	3.922876539	1.802440025
N	-2.077445911	2.683563948	1.412938466
H	-2.266999613	5.863997235	0.891907651
H	-3.274398267	4.818523487	-0.130408011
H	-2.340808205	4.321331795	2.736861298
H	-3.833701188	3.752184688	1.955934555
C	-0.533154977	4.892653409	-0.946735401
C	-0.832325434	4.704110433	-2.305295291
C	-1.825056324	3.652571946	-2.727421974
C	-0.135823114	5.463086326	-3.249523720
C	0.842954456	6.389502074	-2.875116652
C	1.611323923	7.165717411	-3.919422403
C	1.117015167	6.555028948	-1.514414158
C	0.439177355	5.820056117	-0.538182232
C	0.739314044	6.018268344	0.928951752
H	-2.011663609	3.691766470	-3.804036613
H	-2.783831454	3.761959482	-2.208982507
H	-1.435363416	2.663671411	-2.469088254
H	-0.356474540	5.320112295	-4.305472561
H	1.004965913	7.345501866	-4.812888484
H	2.504419463	6.613741779	-4.237931281
H	1.946717906	8.134354462	-3.535315718
H	1.876226336	7.271614864	-1.206776000
H	0.790124779	5.061715143	1.456689768
H	1.684450304	6.552914820	1.066791930
H	-0.041642605	6.609169240	1.425208096
C	-2.139951002	1.570662306	2.306619191
C	-1.154913389	1.381192325	3.300730243
C	-0.079281336	2.409964221	3.561272440
C	-1.251935852	0.258156480	4.125545047
C	-2.313658729	-0.645696889	4.028755195
C	-2.359519211	-1.879039902	4.899436303
C	-3.325654012	-0.378264109	3.104962192
C	-3.265722055	0.725471237	2.248326728
C	-4.435761923	1.031430341	1.342377607
H	0.715902451	1.993733072	4.185028485
H	0.366901272	2.767369132	2.633246483
H	-0.503213181	3.272645327	4.091972610
H	-0.483968649	0.102894463	4.878455599
H	-1.894601227	-1.699989687	5.875199166
H	-3.387085186	-2.214478232	5.071378712
H	-1.819412657	-2.709122184	4.427376800
H	-4.184809011	-1.043428390	3.045005174
H	-5.204937171	1.587922601	1.894352115
H	-4.133937106	1.635527500	0.486461504
H	-4.903365689	0.114124805	0.972618043
S	2.277534742	0.453218080	1.433000408
C	3.034120865	1.836324130	2.072571611
N	2.844405085	3.116776420	1.672948006
C	3.576113386	3.995268825	2.456639290
C	4.254882926	3.251677069	3.364332315
N	3.928800016	1.915969611	3.112369370
H	2.187615096	3.303351485	0.899872940
H	3.543169065	5.061083914	2.301362709
H	4.908786554	3.516507826	4.180479344
C	4.393801661	0.771545766	3.869637458
H	5.399382083	0.977802835	4.238851782
H	4.390597269	-0.094725178	3.206418508
S	5.052059307	1.976504456	6.802574117
C	3.798611964	0.982137593	6.283783083
N	3.557484468	0.483700857	5.018636169
C	2.389820226	-0.282141794	5.001200146
C	1.903746715	-0.288967097	6.264276746
N	2.770883591	0.478944997	7.033038137
H	2.015464494	-0.707497514	4.082987278
H	1.032009890	-0.768256228	6.679039439
H	2.690378108	0.682734632	8.016965897
C	-2.414308134	0.177279728	-1.289574541
H	-2.753962306	-0.115319932	-0.295944576
H	-2.876924532	1.138840523	-1.532654848
H	-2.807928744	-0.566275124	-2.000033154
C	-0.070320853	-0.621022400	-0.585860844
C	1.392333888	2.447091487	-1.055842593
H	1.120459654	3.108326051	-1.883977497
H	0.862254698	-0.951260798	-1.043049029
H	2.459129082	2.169582746	-1.048882495
C	-0.579033879	-1.528544890	0.519081879
H	-1.296564861	-1.030999033	1.179555275
H	-1.078583092	-2.423060401	0.116125239
H	0.251045772	-1.868359968	1.147277453

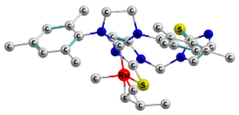

Supplement: Supplementary file 1 — jp1c09336_si_001.pdf [file jp1c09336_si_001.pdf]
